# Supplementary material for: Sampling bias and uneven data coverage shape observed biodiversity patterns in a megadiverse island archipelago hotspot
Source: PLoS One. 2026 Jul 17;21(7):e0353393. doi: 10.1371/journal.pone.0353393 (PMC13379091; doi:10.1371/journal.pone.0353393)
Supplement: S1 Table — All models included PAIC as a random intercept and were fitted using a negative binomial error distribution. Predictor variables were scaled prior to analysis. “Area” refers to log-transformed PCA area, “Occurrence density” refers to log-transformed occurrence records per 100 km², and “Topographic relief” refers to square-root transformed topographic relief. (DOCX) [file pone.0353393.s001.docx]

**Table S1. Top-supported generalized linear mixed-effects models explaining observed herpetofaunal species richness across Philippine conservation-relevant areas (PCAs).** All models included PAIC as a random intercept and were fitted using a negative binomial error distribution. Predictor variables were scaled prior to analysis. “Area” refers to log-transformed PCA area, “Occurrence density” refers to log-transformed occurrence records per 100 km², and “Topographic relief” refers to square-root transformed topographic relief.

| Model | Fixed effects structure | df | logLik | AICc | ΔAICc | Weight |
| --- | --- | --- | --- | --- | --- | --- |
| 1 | PCA type + Area + Occurrence density + Topographic relief + Area × Occurrence density + Area × PCA type + Area × Topographic relief + Occurrence density × PCA type + Occurrence density × Topographic relief + PCA type × Topographic relief | 13 | -662.77 | 1352.75 | 0.00 | 0.400 |
| 2 | PCA type + Area + Occurrence density + Topographic relief + Area × PCA type + Area × Topographic relief + Occurrence density × PCA type + Occurrence density × Topographic relief + PCA type × Topographic relief | 12 | -664.56 | 1354.15 | 1.40 | 0.199 |
| 3 | PCA type + Area + Occurrence density + Topographic relief + Area × PCA type + Area × Topographic relief + Occurrence density × PCA type + PCA type × Topographic relief | 11 | -665.84 | 1354.55 | 1.80 | 0.163 |
| 4 | PCA type + Area + Occurrence density + Topographic relief + Area × Occurrence density + Area × PCA type + Area × Topographic relief + Occurrence density × PCA type + Occurrence density × Topographic relief | 12 | -664.83 | 1354.69 | 1.94 | 0.151 |
| 5 | PCA type + Area + Occurrence density + Topographic relief + Area × PCA type + Area × Topographic relief + Occurrence density × PCA type | 10 | -667.54 | 1355.80 | 3.06 | 0.087 |
