# Supplementary figures and images for: Sampling bias and uneven data coverage shape observed biodiversity patterns in a megadiverse island archipelago hotspot
Source: PLoS One. 2026 Jul 17;21(7):e0353393. doi: 10.1371/journal.pone.0353393 (PMC13379091; doi:10.1371/journal.pone.0353393)

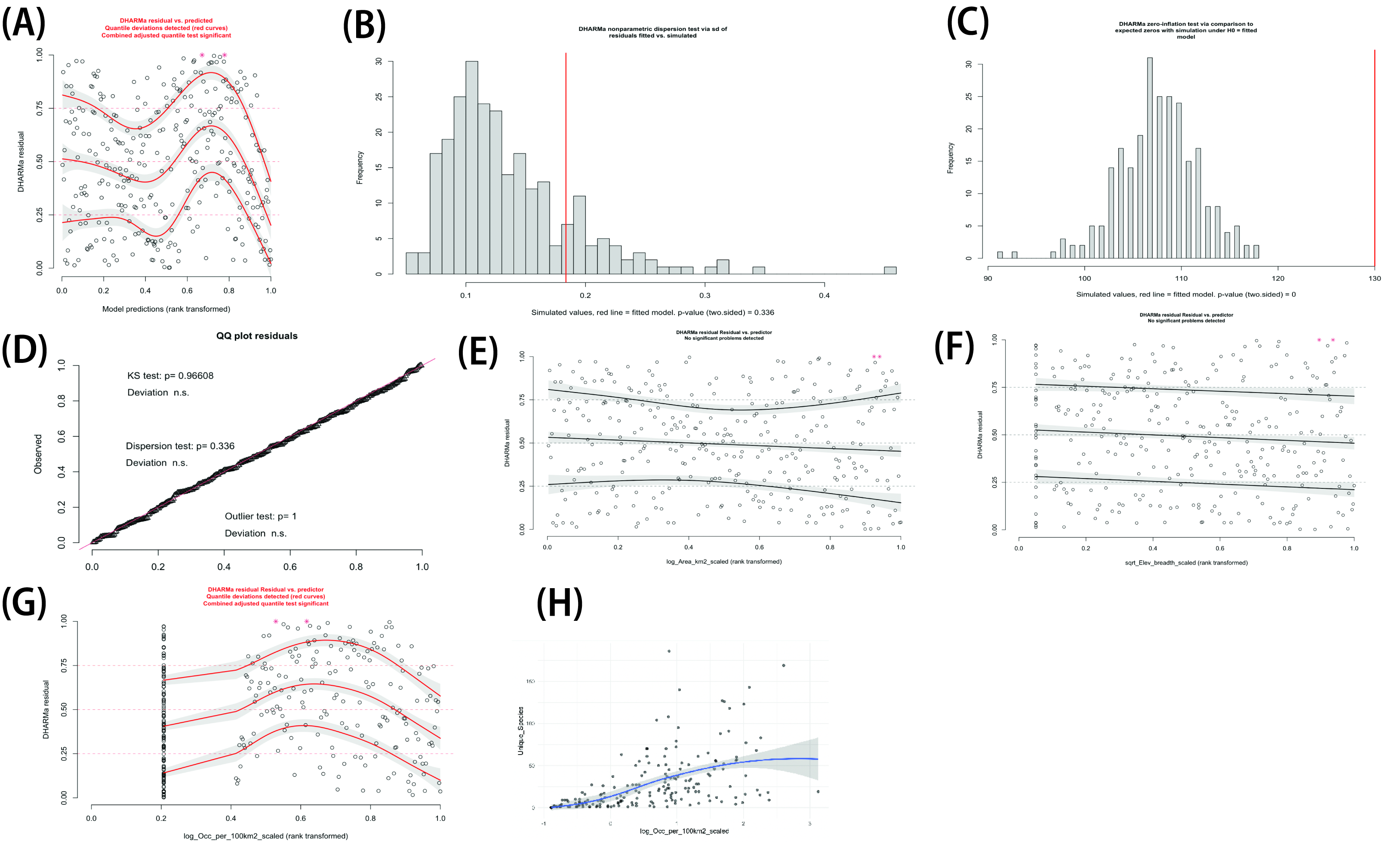

Supplement: S1 Fig — (A) Simulated residuals from the fitted model, showing detected quantile deviations. (B) Nonparametric dispersion test. (C) Zero-inflation test. (D) Q–Q plot used to assess deviations from the expected residual distribution, together with tests for uniformity (Kolmogorov–Smirnov), dispersion, and outliers. (E–F) Scaled residuals plotted against predicted values and against each model predictor. Simulation outliers—observations falling outside the range of simulated values—are highlighted as red stars, while red trend lines indicate statistically significant deviations from model expectations. Deviations were primarily associated with the predictor occurrence density, which likely reflects zero inflation arising from undersampled sites rather than true model misspecification. (G) Bivariate relationship between species richness and occurrence density. (TIF) [file pone.0353393.s002.tif]

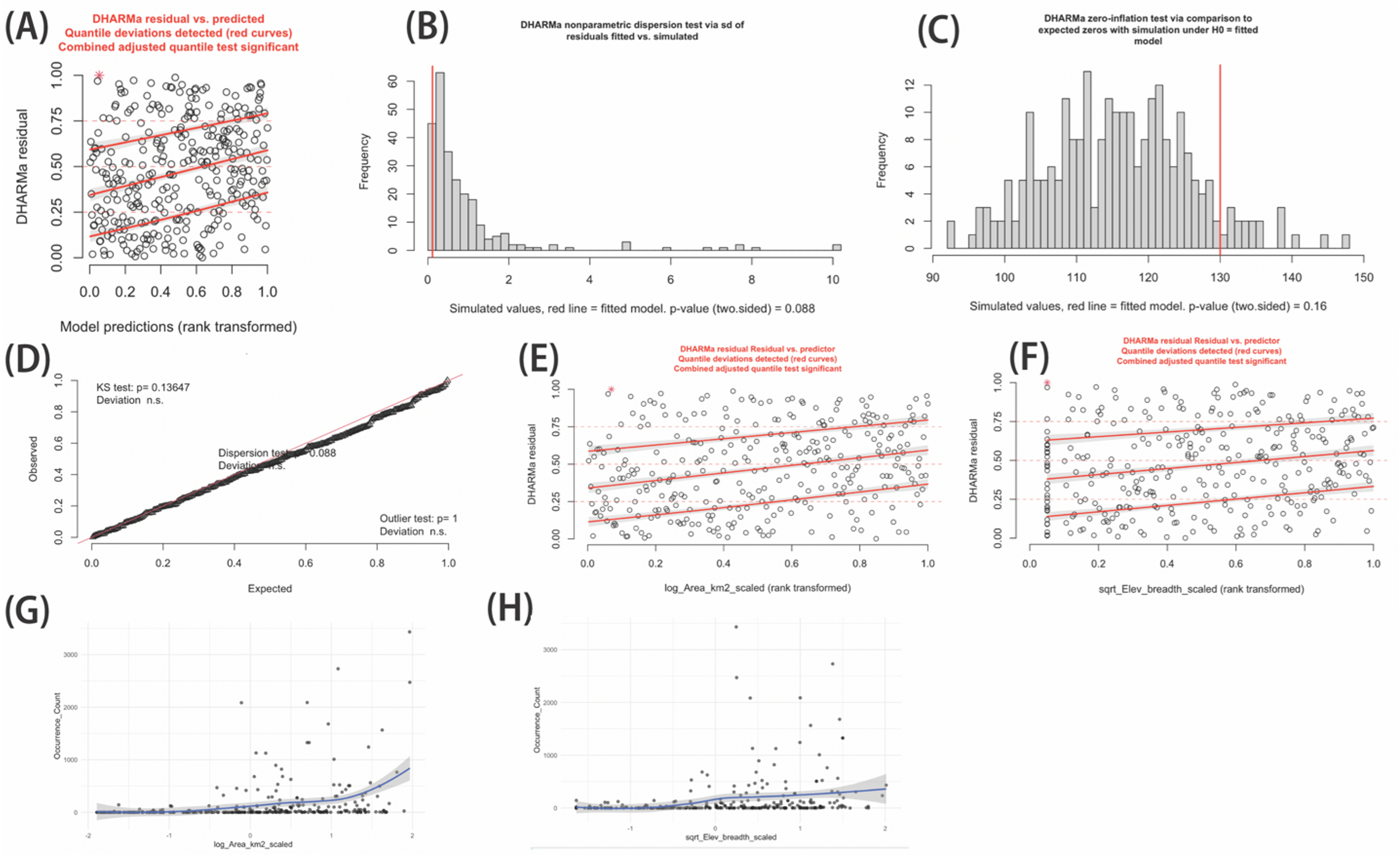

Supplement: S2 Fig — (A) Simulated residuals from the fitted model, showing detected quantile deviations. (B) Nonparametric dispersion test. (C) Zero-inflation test. (D) Q–Q plot used to assess deviations from the expected residual distribution, together with tests for uniformity (Kolmogorov–Smirnov), dispersion, and outliers. (E–F) Scaled residuals plotted against predicted values and against each model predictor. Simulation outliers—observations falling outside the range of simulated values—are highlighted as red stars, while red trend lines indicate statistically significant deviations from model expectations. (G–H) Bivariate relationship between species richness and each model predictor. Deviations were primarily associated with each predictor, likely reflecting increased variance at large PCAs where fewer observations exist. (PNG) [file pone.0353393.s003.png]

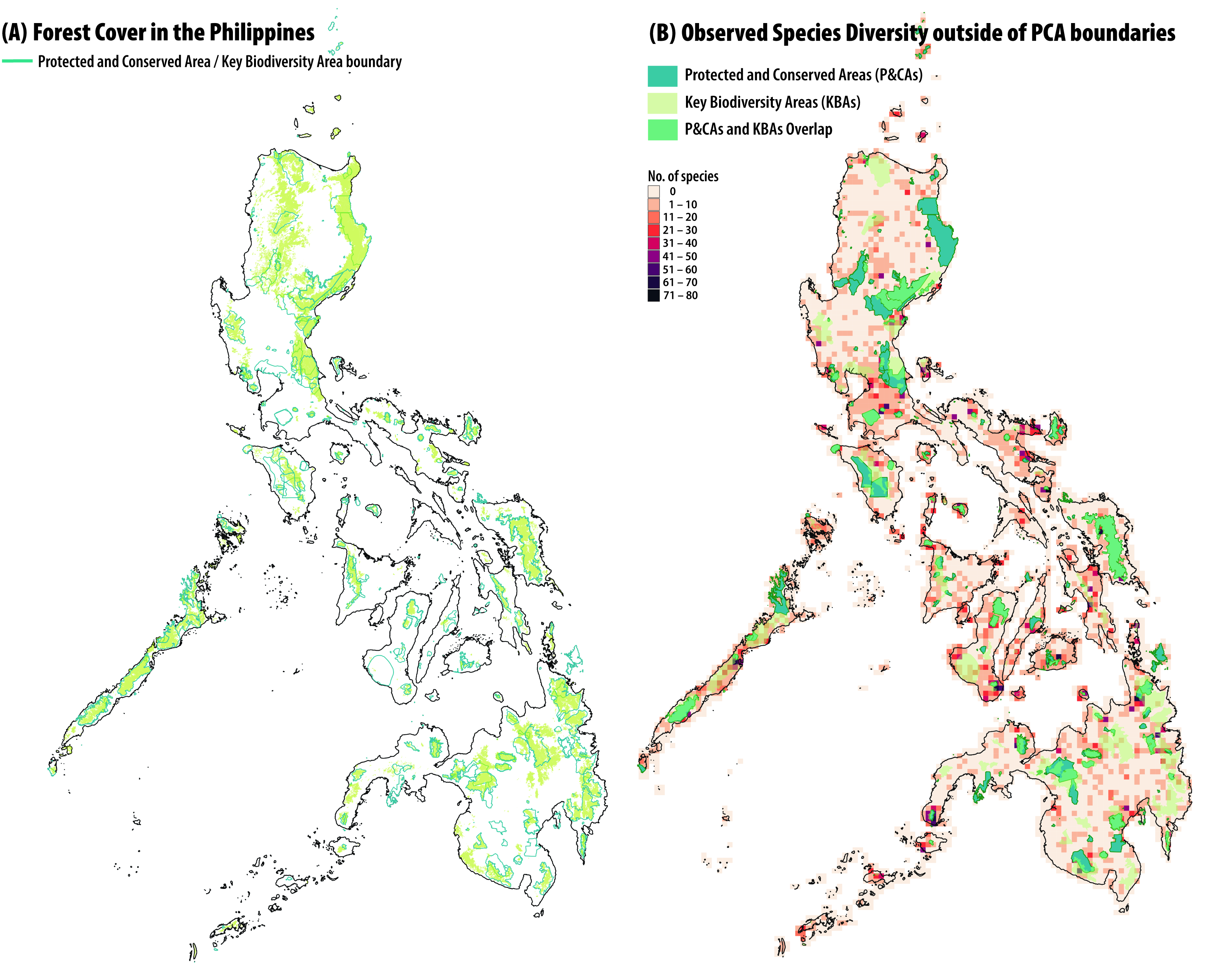

Supplement: S3 Fig — Administrative boundary and biodiversity area shapefiles were sourced from NAMRIA and the DENR-BMB via Geoportal Philippines (https://www.geoportal.gov.ph/). (TIF) [file pone.0353393.s004.tif]
