# Supplementary material for: Sampling bias and uneven data coverage shape observed biodiversity patterns in a megadiverse island archipelago hotspot
Source: PLoS One. 2026 Jul 17;21(7):e0353393. doi: 10.1371/journal.pone.0353393 (PMC13379091; doi:10.1371/journal.pone.0353393)
Supplement: Attachment — Submitted filename: PONE-D-25-55790.pdf [file pone.0353393.s005.pdf]

PLOS ONE

Sampling bias obscures biodiversity patterns, reveals data gaps in priority conservation areas: a call for improved documentation

--Manuscript Draft--

**Manuscript Number:**	PONE-D-25-55790
**Article Type:**	Research Article
**Full Title:**	Sampling bias obscures biodiversity patterns, reveals data gaps in priority conservation areas: a call for improved documentation
**Short Title:**	Biodiversity sampling gaps and biases in a tropical biodiversity hotspot
**Corresponding Author:**	Kier Mitchel E. Pitogo<br>The University of Kansas<br>Lawrence, Kansas UNITED STATES OF AMERICA
**Keywords:**	Philippines, biodiversity data, knowledge shortfalls, protected areas, mountains, macroecology, conservation
**Abstract:**	Where and how species are sampled can shape biodiversity knowledge, spatial patterns, and data-driven conservation. In many Global South biodiversity hotspots, sampling remains uneven, and available data often lack the synthesis needed to assess region-wide gaps for effective conservation planning and priority-setting. This shortfall is common within conserved areas and key biodiversity areas (hereafter 'priority conservation areas' or PCAs). We demonstrate this case in the Philippines, one of the most biodiverse countries in the world, where longstanding biodiversity research and growing policy momentum support efforts to expand coverage of conserved areas. Drawing on over a century of species occurrence records made digitally accessible, we compiled and manually curated these data to assemble and analyze information on Philippine amphibians and squamate reptiles from multiple sources, assessing the spatial distribution of observed diversity in relation to PCAs. Results reveal strong spatial biases, with preserved specimens comprising the majority of records and largely shaping observed diversity patterns. Citizen-science data complement already well-sampled regions, while records from peer-reviewed literature contribute valuable documentation in poorly sampled areas. PCAs are proportionally well-sampled, although gaps and biases remain. Sampling effort and observed diversity were higher in larger PCAs, but this positive area effect diminishes with increasing topographic relief, highlighting large mountain ranges as persistent blind spots in biodiversity documentation. Notably, some areas of higher diversity occur outside established PCAs. We discuss implications of these biases and propose enabling mechanisms to improve primary biodiversity data collection. This study affirms the importance of integrating digitally accessible biodiversity data from multiple sources in revealing sampling gaps and biases, guiding future studies towards poorly sampled areas and informing conservation priorities.
**Order of Authors:**	Kier Mitchel E. Pitogo
	Camila G. Meneses
	Syrus Cesar P. Decena
	Christian E. Supsup
	Hannah E. Som
	Justin M. Bernstein
	Kin Onn Chan
	Mark William Herr
	Rafe Brown
**Additional Information:**	
**Question**	**Response**

Powered by Editorial Manager® and ProduXion Manager® from Aries Systems Corporation

**Financial Disclosure**<br><br>Enter a financial disclosure statement that describes the sources of funding for the work included in this submission. Review the submission guidelines for detailed requirements. View published research articles from *PLOS ONE* for specific examples.<br><br>This statement is required for submission and **will appear in the published article** if the submission is accepted. Please make sure it is accurate.<br><br>**Funded studies**<br>Enter a statement with the following details:<br>• Initials of the authors who received each award<br>• Grant numbers awarded to each author<br>• The full name of each funder<br>• URL of each funder website<br>• Did the sponsors or funders play any role in the study design, data collection and analysis, decision to publish, or preparation of the manuscript?<br><br>Did you receive funding for this work?	The author(s) received no specific funding for this work.
**Competing Interests**<br><br>Use the instructions below to enter a competing interest statement for this submission. On behalf of all authors, disclose any competing interests that could be perceived to bias this work—acknowledging all financial support and any other relevant financial or non-financial competing interests.<br><br>This statement is required for submission and **will appear in the published article** if the submission is accepted. Please make sure it is accurate and that any funding sources listed in your Funding Information later in the submission form are also declared in your Financial Disclosure statement.	The authors have declared that no competing interests exist.

View published research articles from *PLOS ONE* for specific examples.<br><br>**NO authors have competing interests**<br><br>Enter: *The authors have declared that no competing interests exist.*<br><br>**Authors with competing interests**<br><br>Enter competing interest details beginning with this statement:<br><br>*I have read the journal's policy and the authors of this manuscript have the following competing interests: [insert competing interests here]*<br><br>* typeset	
**Ethics Statement**<br><br>Enter an ethics statement for this submission. This statement is required if the study involved:<br><br>• Human participants<br>• Human specimens or tissue<br>• Vertebrate animals or cephalopods<br>• Vertebrate embryos or tissues<br>• Field research<br><br>Write "N/A" if the submission does not require an ethics statement.<br><br>General guidance is provided below. Consult the submission guidelines for detailed instructions. **Make sure that all information entered here is included in the Methods section of the manuscript.**	N/A

**Format for specific study types**<br><br>**Human Subject Research (involving human participants and/or tissue)**<br>• Give the name of the institutional review board or ethics committee that approved the study<br>• Include the approval number and/or a statement indicating approval of this research<br>• Indicate the form of consent obtained (written/oral) or the reason that consent was not obtained (e.g. the data were analyzed anonymously)<br><br>**Animal Research (involving vertebrate animals, embryos or tissues)**<br>• Provide the name of the Institutional Animal Care and Use Committee (IACUC) or other relevant ethics board that reviewed the study protocol, and indicate whether they approved this research or granted a formal waiver of ethical approval<br>• Include an approval number if one was obtained<br>• If the study involved *non-human primates*, add *additional details* about animal welfare and steps taken to ameliorate suffering<br>• If anesthesia, euthanasia, or any kind of animal sacrifice is part of the study, include briefly which substances and/or methods were applied<br><br>**Field Research**<br><br>Include the following details if this study involves the collection of plant, animal, or other materials from a natural setting:<br>• Field permit number<br>• Name of the institution or relevant body that granted permission	
**Data Availability**<br><br>Authors are required to make all data underlying the findings described fully available, without restriction, and from the time of publication. PLOS allows rare exceptions to address legal and ethical concerns. See the PLOS Data Policy and FAQ for detailed information.	Yes - all data are fully available without restriction

A Data Availability Statement describing where the data can be found is required at submission. Your answers to this question constitute the Data Availability Statement and **will be published in the article**, if accepted.<br><br>**Important:** Stating 'data available on request from the author' is not sufficient. If your data are only available upon request, select 'No' for the first question and explain your exceptional situation in the text box.<br><br>Do the authors confirm that all data underlying the findings described in their manuscript are fully available without restriction?	
**Describe where the data may be found in full sentences. If you are copying our sample text, replace any instances of XXX with the appropriate details.**<br><br>• If the data are **held or will be held in a public repository**, include URLs, accession numbers or DOIs. If this information will only be available after acceptance, indicate this by ticking the box below. For example: *All XXX files are available from the XXX database (accession number(s) XXX, XXX.)*.<br>• If the data are all contained **within the manuscript and/or Supporting Information files**, enter the following: *All relevant data are within the manuscript and its Supporting Information files.*<br>• If neither of these applies but you are able to provide **details of access elsewhere**, with or without limitations, please do so. For example:<br><br>*Data cannot be shared publicly because of [XXX]. Data are available from the XXX Institutional Data Access / Ethics Committee (contact via XXX) for researchers who meet the criteria for access to confidential data.*<br><br>*The data underlying the results presented in the study are available from (include the name of the third party*	All data to replicate our analyses are publicly available at https://zenodo.org/records/17344465

and contact information or URL).<br>• This text is appropriate if the data are owned by a third party and authors do not have permission to share the data.<br><br>* typeset	
Additional data availability information:	

Powered by Editorial Manager® and ProduXion Manager® from Aries Systems Corporation

Manuscript

Click here to access/download;Manuscript;081025 Biodiversity Data Manuscript.docx

Sampling bias obscures biodiversity patterns, reveals data gaps in priority conservation areas: a call for improved documentation

Kier Mitchel E. Pitogo[1*], Camila G. Meneses[1], Syrus Cesar P. Decena[1,2], Christian E. Supsup[1], Hannah E. Som[1], Justin M. Bernstein[3], Kin Onn Chan[1,#a], Mark William Herr[1], Rafe M. Brown[1]

[1]*Department of Ecology and Evolutionary Biology & Biodiversity Institute, University of Kansas, Lawrence, KS, USA*

[2]*College of Agricultural and Environmental Sciences, Visayas State University-Alangalang, Leyte, Philippines*

[3]*Department of Mammalogy, Division of Vertebrate Zoology, American Museum of Natural History, New York, NY, USA*

[#a]*Current Address: Department of Integrative Biology & MSU Museum, Michigan State University, East Lansing, MI, USA*

*Corresponding author: Kier Mitchel E. Pitogo

E-mail: pitogo.kiermitchel@gmail.com

Abstract

Where and how species are sampled can shape biodiversity knowledge, spatial patterns, and data-driven conservation. In many Global South biodiversity hotspots, sampling remains uneven, and available data often lack the synthesis needed to assess region-wide gaps for effective conservation planning and priority-setting. This shortfall is common within conserved areas and key biodiversity areas (hereafter 'priority conservation areas' or PCAs). We demonstrate this case in the Philippines, one of the most biodiverse countries in the world, where longstanding biodiversity research and growing policy momentum support efforts to expand coverage of conserved areas. Drawing on over a century of species occurrence records made digitally accessible, we compiled and manually curated these data to assemble and analyze information on Philippine amphibians and squamate reptiles from multiple sources, assessing the spatial distribution of observed diversity in relation to PCAs. Results reveal strong spatial biases, with preserved specimens comprising the majority of records and largely shaping observed diversity patterns. Citizen-science data complement already well-sampled regions, while records from peer-reviewed literature contribute valuable documentation in poorly sampled areas. PCAs are proportionally well-sampled, although gaps and biases remain. Sampling effort and observed diversity were higher in larger PCAs, but this positive area effect diminishes with increasing topographic relief, highlighting large mountain ranges as persistent blind spots in biodiversity documentation. Notably, some areas of higher diversity occur outside established PCAs. We discuss implications of these biases and propose enabling mechanisms to improve primary biodiversity data collection. This study affirms the importance of integrating digitally accessible

biodiversity data from multiple sources in revealing sampling gaps and biases, guiding future studies towards poorly sampled areas and informing conservation priorities.

Introduction

Biodiversity data remain unevenly distributed across taxonomic groups and geographic regions, with pronounced biases against global biodiversity hotspots [1–5]. Several factors contribute to these biases, including limited research capacity [6,7], preferential focus on certain taxonomic groups [3,4,8] and charismatic taxa [9,10], ease of site accessibility [11–13], and prevailing tendency to conduct research in areas of special interest [14,15]. As a result, many regions and their wildlife remain poorly sampled—and where data exist, they are often not digitally accessible or fall into "biodiversity blind spots", obscuring biodiversity patterns [5]. These sampling biases can hinder robust macroecological analyses and obstruct evidence-based conservation planning [17–20], particularly at national scales where most conservation policies and management actions are implemented [21–23]. Therefore, identifying where biodiversity data exist, for which taxa, and to what extent is essential for proactively addressing these biases and strengthening the knowledge base needed for effective conservation.

Challenges in biodiversity data availability are persistent even within global biodiversity hotspots, like the Philippines, where gaps and biases in data coverage skew biodiversity knowledge toward certain regions and taxonomic groups [24–26]. As a megadiverse and biogeographically unique country threatened by habitat loss [27,28], the Philippines urgently requires robust and data-informed conservation strategies. However, limited availability and

discoverability of biodiversity data, and a lack of information on how these data are spatially distributed, constrains efforts to evaluate sampling completeness and to guide national biodiversity strategies and research priorities [23,29]. The need for synthesized biodiversity data to inform conservation planning is especially urgent, as biodiversity loss and the global momentum under the Kunming-Montreal Global Biodiversity Framework [30,31] have prompted policy responses to expand protected areas (PAs) and recognition of other effective area-based conservation measures (OECMs), now covering approximately 15% of the Philippines' land area [32]. This expansion not only demands effective management grounded in ecologically representative, well-connected, and equitably governed networks of PAs and OECMs [30] but also must deliver measurable, positive outcomes for biodiversity [33]. A critical first step towards achieving such outcomes is to strengthen biodiversity data by improving sampling coverage and accessibility [21,23,29,34,35–37].

A key step toward ensuring that biodiversity knowledge of a particular area is sufficient to support conservation strategies is to assess where biodiversity data are spatially distributed and how these patterns align with conserved areas (PAs and OECMs) and key biodiversity areas (collectively referred to herein as 'priority conservation areas' or PCAs). Such an approach remains lacking for the Philippines despite long-standing efforts to document wildlife in this megadiverse, global biodiversity hotspot [24–26,38–47]. While data deficiencies affect many taxonomic groups, some are better represented due to sustained research and collection efforts. Herpetofauna offer a compelling case for such analysis in the Philippines: the group has benefited from over a century of active and sustained research effort [25,38]. In fact, recent years have seen an acceleration of engagement in amphibian and reptile studies, with increasing

numbers of researchers, broader types of investigations, and expanding publication output [25], all suggesting that herpetofauna are a particularly timely and relevant group for such work and that data availability will likely continue to grow. Endemic herpetofauna are also relatively well-sampled genetically, with voucher specimens housed in museum collections [26]; and curated occurrence records are available in substantial volume across peer-reviewed literature [48,49]. Moreover, reptiles have been shown to serve as effective surrogates for broader vertebrate biodiversity patterns in KBAs across the country [50]. Herpetofauna thus provide a valuable lens to evaluate spatial patterns in biodiversity data for improving country-wide biodiversity documentation and ensuring that conservation efforts are grounded in the best available evidence.

Leveraging species data on Philippine herpetofauna spanning ~125 years (1900s–2025) that are made digitally accessible, we compiled, manually curated, and analyzed occurrence records to conduct country-wide spatial assessments of observed diversity of amphibians and squamate reptiles using multiple data sources. Specifically, we ask: (1) How is observed herpetofaunal diversity spatially distributed across the Philippines? (2) How do these patterns differ across datasets derived from museum collections, citizen-science platforms, and peer-reviewed literature? (3) How well do observed diversity patterns coincide with PCAs? And (4) Which PCAs, and which types, are more thoroughly sampled? Our findings offer novel, long-overdue insights into spatial distribution of biodiversity data in the Philippines, particularly in relation to PCAs. Drawing from our results, we propose and discuss enabling mechanisms to improve primary biodiversity data collection, strengthening the knowledge base necessary in assessing management effectiveness for area-based conservation measures needed in the country

[51,52]. By identifying key data shortfalls and sampling biases, this study contributes to the growing body of empirical evidence underscoring the critical role of integrating digitally accessible biodiversity data from multiple sources in revealing sampling gaps and biases, guiding future studies towards poorly sampled areas and informing conservation priorities.

Materials & methods

Data assembly and curation

All georeferenced occurrence records for amphibians and squamate reptiles from the Philippines were downloaded from the Global Biodiversity Information Facility (GBIF) on 31 January 2025 (amphibians) and 07 February 2025 (squamate reptiles). To reduce data duplication, we initially excluded records categorized as material citations and retained only human-observation records from iNaturalist and HerpWatch Pilipinas. These were then merged with expert-curated species occurrence data from the literature: amphibians from the Amphibians of the Philippines, part I: checklist of the species [48] and snakes from the Synopsis of snakes of the Philippines [49]. To avoid overlap between GBIF records and these curated datasets, only GBIF records dated post-publication—2015 onwards for amphibians and 2018 onwards for snakes—were retained. For lizards, all GBIF records were included. Additionally, duplicate field numbers from Sam Noble Oklahoma Museum of Natural History and Father Saturnino Urios University-Biodiversity Informatics and Research Center that corresponded to catalogued specimens from the University of Kansas Natural History Museum (KU) were excluded, with only KU records retained. Finally, we incorporated verifiable occurrence records of non-museum-catalogued specimens from peer-reviewed literature compiled by Meneses et al [25],

along with a few additional entries. For consistency in categorization, all museum records were treated as "preserved specimen" records; iNaturalist and HerpWatch Pilipinas as "citizen science" records; and data from peer-reviewed literature as "material citation" records.

After initial clean-up, we recovered 471 nominal and candidate species of terrestrial amphibians and squamate reptiles that have digitally accessible occurrence records. Occurrence records for each species were then manually curated using currently accepted taxonomic treatments and synonyms. Each species' occurrence records were mapped in QGIS 3.4 Madeira [53] to assess spatial accuracy of each point. Records falling outside the known geographic range of a species and lacking verifiable documentation were excluded. In cases where a formerly widespread species had been taxonomically split, historical records were reassigned to currently accepted species name based on updated diagnostic and distributional information in taxonomic studies. For species with unclear taxonomic or geographic boundaries, only records supported by verifiable data (e.g., curated vouchered specimens, occurrences within known distribution from peer-reviewed literature) were retained. Since expert-curated distributional literature for Philippine lizards is lacking, we adopted an additional measure by comparing our preliminary range estimates with the Global Assessment of Reptile Distributions [54–55]. This conservative step was taken to minimize potential overestimation of species diversity metrics.

Records not identified to the species level were excluded unless a species name or a placeholder name (e.g., sp. + island name, sp. + number) could be confidently assigned without the risk of double-counting (e.g., when only a single species from a genus is known to occur in the area). Given that the frog genus *Platymantis* remains taxonomically unresolved [56–58], a

conservative approach that accounts for this uncertainty was adopted by including records of candidate species occurring in areas with no known range overlap with closely related congeners. This strategy minimizes the risk of double-counting should these populations ultimately represent a single species, while also retaining valuable data that would otherwise be discarded. This decision was informed by phylogenetic evidence, following Brown et al [56].

All records lacking locality information were discarded. Additionally, records with coordinates at 16.45°N, 120.55°E and 13°N, 122°E from GBIF were excluded, as the associated locality descriptions did not match the expected geographic locations. These coordinates appear to be generalized placeholders rather than accurate site data.

Statistical analyses

Mapping herpetofaunal diversity across the Philippines

Occurrence records for amphibians and squamate reptiles were used to generate a presence–absence matrix (PAM) at a 10-km grid resolution across the Philippines, using prepare_base_pam function from the R package biosurvey [59]. Separate PAMs were created for (1) all data types, (2) preserved-specimen records, (3) citizen-science records, and (4) material-citation records. For each PAM, alpha diversity (species richness) was calculated per 10-km grid cell, and the results were visualized as spatial maps using QGIS 3.4 Madeira [53]. The resulting maps of herpetofaunal diversity were overlaid with shapefiles of PCAs to assess spatial overlap between species richness and coverage area. Shapefiles for CAs were obtained from the World

Database on Protected Areas (www.protectedplanet.net) and KBAs [60] from Geoportal Philippines (https://www.geoportal.gov.ph/).

To assess whether species richness classes were evenly represented across grid cells, a chi-square goodness-of-fit test was conducted using the chisq.test function in R version 4.3.1 [61]. Richness values were grouped into nine discrete classes, and the observed frequency of cells in each class was compared to a uniform distribution, under the assumption that all classes were equally likely. Expected counts were computed by dividing the total number of grid cells by the number of richness classes. All expected frequencies exceeded 5, meeting assumptions of the chi-square test.

Predictors of herpetofaunal diversity across priority conservation areas

To quantify species occurrence records and diversity within PCAs, we spatially intersected occurrence points with PCA boundaries using "Join Attributes by Location" tool in QGIS 3.4 Madeira [53] (input: occurrence points; join layer: PCA polygons; predicate: intersects). The resulting dataset was exported as a CSV file and processed in R statistical software [61], where records without PCA matches were excluded. Data were grouped by PCA to calculate the number of occurrence records and unique species per PCA. PCAs without intersecting occurrences were retained with zero counts. Polygon area (in km^2) was calculated using expanse function in the R package terra [62]. Only terrestrial PCAs were included in the analysis, comprising 207 CAs and 109 KBAs.

Topographic relief for each PCA was extracted using a Digital Elevation Model (DEM) of the Philippines downloaded from the CGIAR Consortium for Spatial Information (https://srtm.csi.cgiar.org/). This DEM, provided in Arc/Info Grid format, was clipped to the spatial extents of each PCA. Clipping was performed using QGIS' 'Clip Raster by Mask Layer' tool, with DEM as the input raster and PCA polygon shapefiles as the mask layer. The resulting clipped DEM layers contained elevation data exclusively within each PCA boundary. Subsequently, minimum and maximum elevation values were calculated for each PCA polygon using the 'Zonal Statistics' tool in QGIS. The difference between these values was used to quantify topographic relief (in meters) of each PCA.

To investigate potential drivers of species diversity across PCAs, we fitted generalized linear models with a negative binomial error distribution to account for overdispersion (dispersion parameter < 1, compared to >5 in the Poisson models) and improve model fit (AIC $>$ 1000 for Poisson, but $<$ 1000 for negative binomial models). The response variable was the total number of species recorded per PCA. Fixed predictors included total area (km^2), occurrence density (number of occurrence records per km^2), and topographic relief (m). Total area and topographic relief served as proxies for landscape features, based on hypotheses that larger areas and those spanning broader elevational gradients —by virtue of having heterogenous habitat— support higher biodiversity [63,64]. Occurrence density was used as proxy for sampling effort. Predictor variables were transformed based on their distribution to improve model fit: area and occurrence density were $\log_{10}$-transformed due to strong right skew, and topographic relief was square-root-transformed due to moderate right skew. All predictors were then mean-centered and scaled to facilitate model convergence and interpretation of coefficients.

Because PCAs differ in their underlying species pools due to historical biogeographic structuring or other evolutionary processes, we initially included the Pleistocene Aggregate Island Complex (PAIC; see [27]) as a random effect for potential non-independence in species diversity. However, PAIC explained less than 1% of the variance and did not improve model performance, so it was excluded from the final model. We then fitted a fully factorial model with negative binomial error and performed stepwise model selection based on Akaike Information Criterion (AIC), using the stepAIC function in the R package MASS [65] (Table S1–S2). Residual diagnostics using DHARMa indicated quantile deviations for the occurrence density predictor, likely attributable to an excess of zero values reflecting undersampling, rather than model misspecification. Tests of overdispersion and residual uniformity did not indicate violations of model assumptions.

In addition to modeling species diversity, total occurrence records per PCA were modeled as a function of total area, topographic relief, and PAIC as fixed predictors, using a negative binomial error distribution. A two-way interaction between total area and topographic relief improved model fit and was retained in the final model, as supported by model selection based on AIC (Table S3–S4). DHARMa diagnostics indicated no major violations of model assumptions, although quantile deviations were observed for the total area predictor. These deviations likely reflect an exponential increase in predicted values and a widening spread of residuals at higher area values, where fewer PCAs occur and variability is greater. This sampling effort model aimed to determine whether species-poor areas are genuinely less diverse or simply poorly sampled.

Generalized linear models were fitted using the R package glmmTMB package [66]. Overdispersion was initially assessed using a custom R function that calculates the ratio of the sum of squared Pearson residuals to residual degrees of freedom, with values >1.5 considered indicative of overdispersion requiring correction [67]. Model diagnostics for final models were conducted using the DHARMa package [68]. Simulated residuals were generated with the simulateResiduals function (plot = TRUE), and goodness-of-fit tests on scaled residuals were done using testZeroInflation, testUniformity, and testDispersion functions. Residuals were also plotted against each fixed predictor using plotResiduals to check for non-random patterns. Variance inflation factors (VIFs) for all predictors, calculated using the R package performance [69], were generally within acceptable limits (all VIFs < 5). However, moderate collinearity was observed in KBA species diversity model for the interaction between occurrence density and area (VIF = 6.42), as well as for topographic relief (VIF = 5.34). All diagnostic outputs and residual plots are provided in S1–S4 Figs.

As a complement to model-based inference, bivariate relationships between the response variable and continuous predictors were visually examined. These relationships were plotted using locally estimated scatterplot smoothing (LOESS) with 95% confidence intervals, implemented via the R package ggplot2 [70]. Predictor variables were transformed to match model specifications, and point colors were used to reflect the values of interacting variables with significant effects, unless otherwise stated, aiding interpretation of interaction patterns. For descriptive purposes, Pearson correlation coefficients were calculated for each plot, with

significance assessed at α = 0.05 using the cor.test function. All statistical analyses were conducted in R version 4.3.1 [61] and documented in Supplementary Material.

Results

Spatial distribution of herpetofaunal diversity in the Philippines

Spatial distribution of observed herpetofaunal diversity in the Philippines is non-uniform and fragmented, with unequal national-scale coverage that varies considerably across islands (Fig 1A). The distribution of species richness classes significantly deviated from a uniform expectation ($\chi^2(8) = 15{,}843$, $p < 0.001$), indicating uneven observed species richness across the archipelago. Some richness classes were overrepresented, while others were underrepresented relative to expectations if all classes were equally likely. Nationally, only ~2% (n = 87) of the 10-km² grids have more than 41 observed species—roughly 50% of maximum richness recorded in any grid—while 31% (n = 1,370) have between 1 and 40 species, and 66.7% (n = 2,914) have zero recorded species.

Fig 1. Spatial distribution of observed amphibian and squamate reptile diversity for (A) all data types; (B) preserved-specimen records; (C) citizen-science records; and (D) material-citation records. Species richness is calculated per 10-km² grid cell across the Philippines. Inset bar graph shows frequency distribution of grid cells across species richness classes for each dataset. Dashed red line indicates expected frequency for each class under a uniform distribution, as used in chi-square goodness-of-fit tests.

Different data sources provide complementary insights into herpetofaunal diversity. The majority of occurrence records came from preserved specimens (n = 70,112), followed by citizen science contributions (n = 3,735) and material citations (n = 2,344), while 37 points were unclassified. Both preserved-specimen and citizen-science data represent individual occurrences, while material citations typically reflect species-level records, as published species inventory studies usually report presence by species rather than by individuals. All three data types significantly deviated from a uniform expectation ($\chi^2(8) = 20{,}186–30{,}610$, $p < 0.001$). Because preserved specimens account for 92% of all records, spatial richness patterns largely mirror those derived from the combined dataset (Fig 1B). Citizen science data generally contribute 1–10 species per grid, with a few grids reaching up to 30 species, and tend to complement regions already covered by specimen-based surveys (Fig 1C). Material citation data are concentrated in fewer areas, consistent with the targeted, site-specific nature of biotic inventories (Fig 1D).

Priority conservation areas were proportionally well-sampled, although distribution of herpetofaunal diversity is uneven and concentrated in few areas (Fig 2). Of the 76,228 curated occurrence records, 21,210 (27.8%) fell within CAs and 23,058 (30.2%) within KBAs—proportions that are disproportionately high relative to the Philippine land area occupied by these designations (12–15% for CAs and 6.72% for KBAs). Overlaying species richness with PCA boundaries shows that whereas some high-richness grid cells lie within PCAs, many large sites include cells with sparse or no records. Notably, 52.7% of 207 CAs and 20.2% of 109 KBAs contain grid cells with zero species records, and an additional 22.4% of CAs and 29.4% of KBAs contain only up to 10 recorded species. At the same time, several well-sampled areas with high

observed diversity (>40 recorded species) occur outside established CAs but within forested areas, many of which partially overlap with KBAs (S5 Fig).

Fig 2. **Relative locations of well-sampled (≥40 species) conserved areas and key biodiversity areas across the Philippines**. Total area, occurrence point density, and number of species are included for each area. Base map shows spatial distribution of observed amphibian and squamate reptile diversity for all data types. Inset histogram shows frequency distribution of priority conservation areas by species richness and number of occurrence records.

Well-surveyed sites, those with >40 recorded species, make up 8.8% of CAs and 18.3% of KBAs (Fig 2). Particularly species-rich locations (>60 species) include Samar Island Natural Park, Pasonanca Natural Park, Mt. Malindang Natural Park, Mt. Makiling Forest Reserve, and PP1636 (unnamed wildlife sanctuary in southcentral Luzon). These sites have been extensively surveyed, with data available in public repositories like GBIF, though many records remain absent from, or not reported in, peer-reviewed literature (but see [71,72]). Other well-documented areas combine strong specimen representation in collections with published data: Cuernos de Negros [73,74], Aurora Memorial National Park [75,76], Mt. Busa–Kimba [58,77], Mt. Guiting-Guiting [78,79], Mt. Hilong-hilong [57,80], Pantabangan-Caranglan Watershed Reservation [81], and Victoria-Anepahan Ranges. The 40-species cut-off, however, is used here solely as a reference point for discussion and should not be interpreted as a true threshold of diversity, since observed values remain influenced by sampling limitations and potential biases.

Predictors of species diversity across conservation areas

Species diversity in PCAs was best explained by models that included total area, occurrence density, and their interactions with topographic relief, although the structure of these relationships varied. For CAs, the top-ranked model included total area, occurrence density, topographic relief, and an interaction between area and topographic relief (AIC = 875.8; Table S1). Both total area (1.9556 ± 0.1894 SE, $z = 10.326$, $p < 0.01$) and occurrence density (1.4503 ± 0.1496 SE, $z = 9.696$, $p < 0.01$) were strong positive predictors of species diversity (Table 1).

Table 1. Coefficient estimates from generalized linear models (negative binomial) relating species diversity to total area, occurrence density, and topographic relief for priority conservation areas in the Philippines.

Predictors	Estimate ± SE	z-value	p-value
Conserved Areas			
Intercept	0.6405 ± 0.1423	4.502	<0.01*
Total Area	1.9556 ± 0.1894	10.326	<0.01*
Occurrence Density	1.4503 ± 0.1496	9.696	<0.01*
Topographic Relief	0.0021 ± 0.1645	0.013	0.989
Area * Topographic Relief	-0.2322 ± 0.1430	-1.624	0.104
Key Biodiversity Areas			
Intercept	2.8210 ± 0.1279	22.049	<0.01*
Total Area	0.8974 ± 0.1553	5.780	<0.01*
Occurrence Density	1.3127 ± 0.1868	7.027	<0.01*

Topographic relief	0.2019 ± 0.1435	1.407	0.1593
Area * Occurrence Density	0.5501 ± 0.2188	2.514	0.0119*
Area * Topographic Relief	-0.4243 ± 0.1439	-2.948	0.0032*
Occurrence Density * Topographic Relief	-0.5392 ± 0.2362	-2.283	0.0225*

Note: Total area and occurrence density were log-transformed, and topographic relief was square-root-transformed; all predictors were scaled and mean-centered. Significance assessed at $\alpha = 0.05$(*).

Results from model-based inference aligned with patterns observed in scatterplots, which showed moderate positive correlations between species diversity and total area (Pearson's $r = 0.51$, $p < 0.05$; Fig 3A), and a steep but variable trend for occurrence density ($r = 0.53$, $p < 0.05$), particularly at higher values (Fig 3B). A slightly weaker positive correlation was found between species diversity and topographic relief, with an initial increase that flattened at broader elevational ranges ($r = 0.41$, $p < 0.05$; Fig 3C).

Fig 3. Scatterplots showing species diversity in relation to total area, occurrence density, and topographic relief for conserved areas (top panels) and key biodiversity areas (bottom panels). Dashed line represents LOESS-smoothed trend, with grey shaded area indicating 95% confidence interval. Each point represents a priority conservation area; colors denote interacting variables with significant effects or inclusion in the best-fit generalized linear models. No interactions were significant in the top panels, although area × topographic relief interaction was retained in the best model. Y-axis is fixed at a lower limit of 0 for better visualization. Pearson correlation coefficients (r) and p-values are shown; significance is based on $\alpha = 0.05$.

For KBAs, the best-supported model additionally included interactions between topographic relief and both total area and occurrence density (AIC = 779.2; S2 Table). Occurrence density (1.3127 ± 0.1868 SE, $z = 7.027$, $p < 0.01$) was a stronger predictor than total area (0.8974 ± 0.1553 SE, $z = 5.780$, $p < 0.01$), and their interaction was positive (0.5501 ± 0.2188 SE, $z = 2.514$, $p = 0.0119$) (Table 1). Topographic relief had no significant main effect on species diversity, but its interactions with total area (–0.4243 ± 0.1439 SE, $z = –2.948$, $p = 0.0032$) and occurrence density (–0.5392 ± 0.2362 SE, $z = –2.283$, $p = 0.0225$) were both significant and negative. These results suggest that topographic relief may modulate influence of area and sampling effort on observed species diversity in KBAs.

Similar patterns emerged in the scatterplots: species diversity generally increased with total area, though the relationship flattened at intermediate values before rising again ($r = 0.31$, $p < 0.05$; Fig 3D). A steep initial increase was also observed with occurrence density, followed by greater variability at higher densities ($r = 0.46$, $p < 0.05$; Fig 3E). The association between topographic relief and species diversity was also weak but positive ($r = 0.30$, $p < 0.05$; Fig 3F), resembling the pattern observed in CAs.

Predictors of sampling effort across conservation areas

Sampling effort in PCAs was best explained by models that included total area, topographic relief, their interaction, and biogeographic subregion (PAIC) as a categorical predictor (AIC = 1348.4 for CAs; AIC = 1172.9 for KBAs; S3–S4 Tables). In CAs, both total

area (1.1765 ± 0.3709 SE, z = 3.172, p = 0.0015) and topographic relief (1.2525 ± 0.3938 SE, z = 3.181, p = 0.0015) were strong positive predictors of sampling effort (Table 2). However, their interaction had a significant negative effect (–0.6413 ± 0.2683 SE, z = –2.390, p = 0.0168), suggesting that the combined influence of total area and topographic relief on sampling density may diminish at high values. Sampling effort was highest in Luzon, the reference level, among all PAICs. No other PAIC differed significantly from Luzon, except West Visayas, which exhibited comparably higher sampling effort (3.3097 ± 0.8625 SE, z = 3.827, p < 0.01).

Table 2. Coefficient estimates from generalized linear models (negative binomial) relating number of occurrence records to total area and topographic relief for priority conservation areas in the Philippines.

Predictors	Estimate ± SE	z-value	p-value
Conserved Areas			
Intercept	3.3381 ± 0.3142	10.62	< 0.01 *
Total Area	1.1765 ± 0.3709	3.172	0.0015 *
Topographic Relief	1.2525 ± 0.3938	3.181	0.0015 *
PAIC: Mindanao	0.1343 ± 0.4606	0.292	0.7706
PAIC: Mindoro	0.5381 ± 1.5006	0.359	0.72
PAIC: Palawan	0.4310 ± 1.0143	0.425	0.6709
PAIC: Romblon Island Group	1.9644 ± 1.9589	1.003	0.3160
PAIC: Sulu Island Group	0.4890 ± 2.8422	0.172	0.8634
PAIC: West Visayas	3.3097 ± 0.8625	3.837	0.0001 *
Area * Topographic Relief	-0.6413 ± 0.2683	-2.390	0.017 *

Key Biodiversity Areas			
Intercept	4.9874 ± 0.3419	14.59	< 0.01 *
Total Area	0.3027 ± 0.2802	1.080	0.28
Topographic Relief	0.4453 ± 0.2790	1.596	0.1105
PAIC: Mindanao	0.3802 ± 0.4864	0.782	0.4345
PAIC: Mindoro	-0.7675 ± 0.7252	-1.058	0.29
PAIC: Palawan	0.4971 ± 0.8467	0.587	0.5571
PAIC: Romblon Island Group	0.5533 ± 1.1229	0.493	0.6222
PAIC: Sulu Island Group	-4.7948 ± 1.5603	-3.073	0.0021 *
PAIC: West Visayas	1.1147 ± 0.5995	1.859	0.0630
Area * Topographic Relief	-0.3679 ± 0.1937	-1.899	0.0576

Note: Total area was log-transformed and topographic relief was square-root-transformed; continuous predictors were scaled and mean-centered. Significance assessed at $\alpha = 0.05$(*). PAIC = Pleistocene Aggregate Island Complexes. Reference level for PAIC is Luzon.

In KBAs, the same model structure identified a strong positive effect of the intercept (Luzon) and weak individual effects of total area and topographic relief (Table 2). Although total area (0.3027 ± 0.2802 SE, $z = 1.080$, $p = 0.2801$) and topographic relief (0.4453 ± 0.2790 SE, $z = 1.596$, $p = 0.1105$) were not individually significant, the interaction term showed a marginally significant negative effect (–0.3679 ± 0.1937 SE, $z = –1.899$, $p = 0.0576$). Luzon, the reference level or intercept, had the highest sampling effort among PAICs. Only the Sulu Island Group had significantly lower sampling effort compared to Luzon (–4.7948 ± 1.5603 SE, $z = –3.073$, $p = 0.0021$), possibly reflecting logistical challenges or reduced field access in this region.

These model-based findings were broadly consistent with scatterplot analyses. Among CAs, weak positive correlations were observed between sampling effort and both total area ($r = 0.32$, $p < 0.05$; Fig 4A) and topographic relief ($r = 0.27$, $p < 0.05$; Fig 4B). For KBAs, correlations were weaker ($r = 0.24$ for total area, Fig 4C; and $r = 0.19$ for topographic relief, Fig 4D; both $p < 0.05$), likely reflecting influence of zero values or sampling gaps across several sites.

Fig 4. Scatterplots showing number of occurrence points in relation to total area and topographic relief for conserved areas (top panels) and key biodiversity areas (bottom panels). Dashed line represents LOESS-smoothed trend, with grey shaded area indicating 95% confidence interval. Each point represents a priority conservation area; colors indicate interacting variables with significant effects. Y-axis is fixed at a lower limit of 0 for better visualization. Pearson correlation coefficients (r) and p-values are shown; significance at $\alpha = 0.05$.

Discussion

Uneven spatial diversity pattern reflects sampling biases

Sustained biodiversity documentation across the Philippines has enabled spatial mapping of observed species diversity of amphibians and squamate reptiles, revealing stark patterns of sampling biases. More than two-thirds of grid cells contain either zero or only 1–10 documented

species, while comparatively fewer cells (~2%) exhibit higher species diversity levels. This pronounced disparity reflects persistent spatial biases in biodiversity knowledge. Observed species diversity patterns likely indicate where sampling has been more intensive, rather than accurately representing underlying ecological or biogeographic factors—a pattern that is persistent across many biodiversity-rich areas globally [17,20,21,83]. Addressing these biases is critical not only for improving knowledge of Philippine biodiversity but also for ensuring that conservation policy, priority-setting, and management decisions rest on a more complete and representative knowledge base.

A recent comprehensive review of Philippine herpetology revealed substantial geographic gaps in field sampling across the archipelago (see [25] for a detailed discussion). Our findings broadly align with these observations but offer additional fine-scale information by integrating curated species point data and grid-based species richness estimates. This approach not only identifies where sampling has occurred but also quantifies its intensity, demonstrating that higher diversity values are associated with well-sampled sites. Notably, we found that a disproportionate share of documented herpetofaunal diversity is concentrated in a few intensively surveyed areas—mostly in Luzon, the largest island, and in West Visayas, comprising most central islands—where collection efforts in recent decades have been more intense [25,38]. High-resolution data of this kind are critical for accurately representing spatial patterns of sampling effort, thereby guiding resources and research efforts towards poorly explored regions. Furthermore, by incorporating species point data from different sources, we recovered diversity data in areas that would otherwise appear as knowledge gaps, such as the Sulu Archipelago in the south [25]; although sampling effort is still disproportionately lower in

this region. As such, given that biodiversity data in the Philippines are fragmented, reliance on selective or incomplete datasets for *any* synthesis studies can introduce substantial bias [84].

Priority conservation areas (PCAs) are relatively well-sampled in proportion to their total area, yet herpetofaunal knowledge across them remains limited and uneven. Despite these gains, many PCAs still lack species records altogether or may have data that are not accessible in digital form. Where species data are lacking in PCAs, they are however available outside delineated boundaries, which may suggest persistent issues associated with sampling within PCAs, including permitting and other bureaucratic processes (particularly for legislated protected areas), logistical access, and security constraints [25,57,85] (see Fig 5). Although well-surveyed sites include some of the country's known PCAs, vast portions of other mountain ranges and many smaller islands remain poorly sampled or lack species data that are readily accessible for broader scientific and conservation use. Expanding targeted field-based surveys beyond established PCAs—and ensuring that resulting data are made publicly and digitally available for mobilization—remains urgently needed to support conservation initiatives grounded in accurate, comprehensive biodiversity knowledge [21,86].

Fig 5. Constraints in field-based biodiversity data collection and corresponding enabling mechanisms to improve primary biodiversity data coverage. Each mechanism primarily addresses one or more constraint types.

Sampling biases skew knowledge toward large conservation areas

Documenting biodiversity is critical in areas where such information can directly inform protection and management decisions. Conserved areas remain central to efforts aimed at safeguarding biodiversity across genetic, species, and ecosystem levels [87,88]. Complementing these, KBAs, though lacking formal protection or management, are identified as priority sites for future conservation efforts [89]. Thus, biodiversity data support monitoring and adaptive management within CAs and guiding prioritization and investment in KBAs. However, digitally accessible records for Philippine herpetofauna show strong uneven documentation: some sites are well-surveyed, others poorly sampled, and many contain no (digitally accessible) records. These disparities mask true biodiversity patterns and pose significant challenges for efficient, evidence-based conservation planning [90].

Observed species diversity and sampling effort for herpetofauna were generally higher in larger PCAs. However, this relationship weakens with increasing topographic relief. Our results suggest that although larger areas tend to receive more sampling effort (and have higher observed diversity), this effect weakens in high mountainous areas. The significant negative interaction between topographic relief and total area on observed diversity contrasts with hypothesis that topographic complexity, by virtue of hosting diverse habitat types, should support higher species diversity [17,63,64,91], an inconsistency that likely reflects sampling limitations. Mountainous areas in the Philippines, despite their potential for high species richness, are often poorly sampled likely due to logistical constraints, along with other factors [92] (see Fig 5). This hypothesis is especially supported by our results, which recovered significant negative interaction effects between topographic relief and sampling effort. These challenges systematically bias biodiversity knowledge away from topographically complex

areas, which are some of the most ecologically critical and conservation-relevant regions in the country [38,93].

Data limitations are not confined to large mountain ranges. Smaller conservation areas, particularly those in island environments, also suffer from substantial biodiversity knowledge gaps [50]. Although this pattern was not statistically prominent in our model-based inference, it was evident in our exploratory data analyses and visualizations (e.g., small-sized, low-topographic-relief PCAs in Figs 3–4). Many small island conservation areas in the Philippines remain poorly sampled despite yielding newly discovered species and harboring unique, range-restricted taxa [78,94–102]. These systems face disproportionate threats from climate-driven sea-level rise, extreme weather events, invasive species, and habitat loss [103], yet lack the baseline biodiversity data for adaptive management. Targeted surveys are urgently needed in these areas to document species presence and build taxonomic and ecological knowledge base required for effective conservation in islands [104]. Without action, island ecosystems risk becoming critical blind spots in the country's conservation efforts.

Data gaps and biases persist, but diverse sources can address them

The majority of digitally accessible knowledge on Philippine herpetofauna comes from natural history collections. These collections represent over a century of fieldwork that has shaped taxonomic and systematics knowledge, increasing herpetofaunal diversity estimates in the country [25,38]. As such, specimen-associated data serve as a vital resource for improving knowledge on biodiversity patterns [5,105]. They also support large-scale ecological studies,

which depend on spatially referenced records to examine broad biodiversity trends and guide conservation planning [16,31,106]. Although many regions remain underrepresented in biodiversity data, it is noteworthy that areas with limited contemporary surveys—as mentioned earlier, Sulu Archipelago, where fieldwork has been limited [25]—still hold valuable records preserved through historical collections.

In areas where specimens are lacking, citizen science provides a valuable complementary data stream to help address distributional biases. Online biodiversity platforms [107–109] and social media [110–112] have become particularly useful where museum records are absent or limited. Although still emerging in Philippine herpetology (but see [113,114]), citizen-science contributions are already well established in other taxonomic groups. For example, active birding communities regularly contribute to platforms like eBird [115] and collaborate with researchers and biodiversity managers to inform site-level conservation efforts in the Philippines [116]. Another notable initiative is Co's Digital Flora of the Philippines [117], where citizen scientists and taxonomists work together to maintain a real-time overview of Philippine flora. At least 54% of the country's vascular plant species have been photo-documented, many with associated geographic coordinates [40]. These examples show that citizen-science contributions to observed diversity may be more pronounced in taxonomic groups with active, organized communities driving such efforts. Despite challenges related to data quality and metadata completeness, carefully curated citizen-science records can enhance biodiversity knowledge, particularly in remote or poorly sampled regions [107,118].

Another important yet often overlooked source of biodiversity data comes from formal surveys that do not involve specimen collection and eventual deposition in natural history museums [58,81,119–121]. And if there are such records, they are often not digitized and published in publicly accessible domains, especially when deposited in university-based museums. Many in-country scientists conduct fieldwork that reports valuable records published in peer-reviewed journals, yet these data are rarely archived in open-access databases such as GBIF [122]. Such studies frequently document species in poorly sampled areas in the Philippines, providing crucial complementary information [25]. Although valuable for expanding species distribution knowledge, many of these studies lack specific geographic coordinates of areas sampled, limiting their use for geospatial research; thus, submission of spatial occurrences to online databases like GBIF is highly encouraged [36]. In addition, a wealth of biodiversity data remains locked in grey literature—government reports, university theses, and project documents—that are not digitally accessible but are often used in site-level management. Incorporating these sources into public repositories and ensuring they are properly curated would improve national biodiversity coverage [123].

Disparate biodiversity data types underscores the need for standardized archiving practices [124–126] and greater adherence to best-practice guidelines in dealing with big data [20,127]. Although these varied data streams help fill distributional gaps, they often differ in quality, accessibility, and curation. These differences are especially true for non-specimen-based data, which typically lack the standardized metadata associated with museum specimens. To maximize their scientific utility, we recommend that non-specimen-based records be accompanied by metadata, including GPS coordinates (including uncertainty), observation date

and time, natural history notes, among others. Robust metadata not only enhances credibility and utility of individual records but also facilitates their integration into broader ecological, biogeographic, and conservation research [16].

Scaling biodiversity documentation to meet conservation targets

The Kunming-Montreal Global Biodiversity Framework, adopted in 2022, sets an ambitious goal: to protect 30% of the world's terrestrial and marine ecosystems by 2030, building on the earlier Aichi Target 11 [128]. Achieving this target requires more than simply expanding protected area coverage; it also demands effective management that delivers measurable benefits for biodiversity [30]. These outcomes depend on robust, accurate biodiversity data, particularly within PCAs [34,129,130]. However, our results show that persistent data gaps and biases, especially in large mountainous and island conservation areas, continue to shape observed biodiversity data patterns. Although many PCAs with apparent data gaps may in fact hold biodiversity data, their lack of digital accessibility limits inclusion in national-scale assessments of sampling effort. This limitation hinders resource allocation for improved documentation, reduces opportunities for external vetting and expert curation of data, and impedes integration into broader datasets that inform national conservation planning [21,31,125].

Addressing the challenge of insufficient biodiversity documentation requires developing and implementing enabling mechanisms to overcome constraints in site-level primary data collection, which comprises the majority—if not all—of available biodiversity data (Fig 5). This

includes prioritizing biodiversity documentation within national conservation policies and planning frameworks (e.g., Philippine Biodiversity Strategy and Action Plan, PBSAP 2024–2040) to strengthen institutional support for field-based activities, especially within PCAs [23]. For example, streamlining research permitting processes, fully aligned with Indigenous Peoples' rights and national access and benefit-sharing policies, can reduce bureaucratic barriers to ethical and standardized data collection [36,131]. Engaging Indigenous Peoples and local communities as collaborators, and supporting culturally appropriate awareness and communication initiatives, can further foster equitable partnerships and mutual knowledge exchange [132]. Financial mechanisms within PAs, such as the Integrated Protected Area Fund, can be leveraged to support student-led and locally collaborative field research. Local or subnational government policies and support mechanisms are also critical for ensuring logistical coordination, safe access, and community involvement in fieldwork.

Beyond site-level interventions, targeted national funding for species discovery and taxonomic capacity-building can incentivize early-career researchers to pursue field-based work [131,133]. These efforts should be complemented by investments in biodiversity science within university curricula [134], ensuring students are trained in both theory and practice. Addressing the limited data management capacity among local researchers is also critical for promoting effective data-sharing, enhancing the verifiability and utility of biodiversity studies. Comprehensive training programs and standardized data systems can help overcome this barrier, ensuring the production of high-quality, accessible datasets. In parallel, the increasing diversity of data contributors underscores the need for a streamlined and curated national biodiversity data infrastructure [135]. Such a platform should ensure that biodiversity data, especially those

generated from PCAs, are digitally and publicly accessible, enabling diverse stakeholders to collectively contribute to and benefit from biodiversity knowledge. Ultimately, these in-country initiatives must be paired with stronger institutional mechanisms, such as equitable collaboration standards and data-sharing agreements, to ensure that non-monetary benefits from biological resource use are meaningfully shared with local collaborators and inform national policy and conservation planning [136].

Finally, scaling biodiversity documentation beyond existing PCAs can guide the identification of new priority sites for protected area establishment. Our findings show that several well-sampled, high-diversity areas remain outside current PCAs, many overlapping with only partially protected KBAs, underscoring opportunities to align future CA expansion with empirically documented biodiversity patterns. The updated national biodiversity strategy and action plan (PBSAP 2024–2040) targets protecting 24% of terrestrial areas through CAs [137], yet only ~15% are currently protected. Prioritizing well-sampled, high-diversity areas for protection will be critical to achieving this goal. Our results, however, only focus on herpetofauna and similar data gaps and biases likely affect many taxonomic groups in the Philippines [24,26] reinforcing the need for broader, inclusive biodiversity documentation. Building a robust and representative biodiversity knowledge base—well-curated and aligned with FAIR (findable, accessible, interoperable, reusable) and CARE (collective benefit, authority to control, responsibility, ethics) principles—will require sustained collaboration among scientists, conservation practitioners, institutions, and critically, with Indigenous Peoples and local communities [16,138,139]. Strengthening such collective and inclusive efforts is essential

for transforming biodiversity knowledge into tangible conservation outcomes and ensuring the Philippines meets its national and global biodiversity commitments.

Acknowledgements

During the preparation of this manuscript, KMEP was partially supported by a Graduate Research Assistantship from the University of Kansas Department of Ecology and Evolutionary Biology; and RMB was supported by a Fulbright International Scholar Award administered by the U.S. Department of State, the Philippine-American Education Foundation (PAEF) and the Philippine Council for International Exchange of Scholars. Support for fieldwork that generated many specimen-associated records was provided by the U.S. National Science Foundation (DEB 073199, 0743491, 1418895, 0910341, 0804115, 1654388, and 0334952 and EF-0334952 to RMB and KU-based students), the KU College of Liberal Arts and Sciences Graduate Assistantship in Support of Faculty Research Award, a KU Biodiversity Institute Research and Exploration Fund grant, the South, Southeast, and East Asia Fund of the KU Office of International Affairs, and the General Research Fund of the University of Kansas. We also thank Marites Bonachita Sanguila, Michael Clores, Michael Cuesta, Aljohn Jay Saavedra, and Tristan Luap Senarillos for numerous discussions and their valuable insights on the challenges of primary biodiversity data collection in the Philippines.

References

1. Hortal J, De Bello F, Diniz-Filho JAF, Lewinsohn TM, Lobo JM, & Ladle RJ. Seven shortfalls that beset large-scale knowledge of biodiversity. Annual Review of Ecology, Evolution, and Systematics. 2015; 46(1): 523–549.
2. Hughes AC, Orr MC, Ma K, Costello MJ, Waller J, Provoost P, et al. Sampling biases shape our view of the natural world. Ecography. 2021; 44(9): 1259–1269.
3. Moura MR, Jetz W. Shortfalls and opportunities in terrestrial vertebrate species discovery. Nature Ecology & Evolution. 2021; 5(5): 631–639.
4. Ondo I, Dhanjal-Adams KL, Pironon S, Silvestro D, Colli-Silva M, Deklerck V, et al. Plant diversity darkspots for global collection priorities. New Phytologist. 2024; 244(2): 719–733.
5. Ball L, Rodríguez-Machado S, Paredes-Burneo D, Rutledge S, Boyd DA, Vander Pluym D, et al. What 'unexplored' means: mapping regions with digitized natural history records to look for 'biodiversity blindspots'. PeerJ. 2025; 13: e18511.
6. Barber PH, Ablan-Lagman MCA, Berlinck RG, Cahyani D, Crandall ED, Ravago-Gotanco R, et al. Advancing biodiversity research in developing countries: the need for changing paradigms. Bulletin of Marine Science. 2014; 90(1): 187–210.
7. Zhang L, Yang L, Chapman CA, Peres CA, Lee TM, Fan PF. Growing disparity in global conservation research capacity and its impact on biodiversity conservation. One Earth. 2023; 6(2): 147–157.
8. Titley MA, Snaddon JL, Turner EC. Scientific research on animal biodiversity is systematically biased towards vertebrates and temperate regions. PloS one. 2017; 12(12): e0189577.

9. Troudet J, Grandcolas P, Blin A, Vignes-Lebbe R, Legendre F. Taxonomic bias in biodiversity data and societal preferences. Scientific Reports. 2017; 7(1): 9132.
10. Guénard B, Hughes AC, Lainé C, Cannicci S, Russell BD, Williams GA. Limited and biased global conservation funding means most threatened species remain unsupported. Proceedings of the National Academy of Sciences. 2025; 122(9): e2412479122.
11. Oliveira U, Paglia AP, Brescovit AD, de Carvalho CJ, Silva DP, Rezende DT, et al. The strong influence of collection bias on biodiversity knowledge shortfalls of Brazilian terrestrial biodiversity. Diversity and Distributions. 2016; 22(12): 1232–1244.
12. Mandeville CP, Nilsen EB, Finstad AG. Spatial distribution of biodiversity citizen science in a natural area depends on area accessibility and differs from other recreational area use. Ecological Solutions and Evidence. 2022; 3(4): e12185.
13. Penhacek M, Castro-Souza RA, Tessarolo G, Diniz-Filho JA, Sobral-Souza T, de Jesus Rodrigues D. Biases in amphibian sampling in the Amazon: Using infrastructure and accessibility data to identify sampling gaps. Biotropica. 2025; 57(5): e70079.
14. Sastre P, Lobo JM. Taxonomist survey biases and the unveiling of biodiversity patterns. Biological Conservation. 2009; 142(2): 462–467.
15. Mentges A, Blowes SA, Hodapp D, Hillebrand H, Chase JM. Effects of site-selection bias on estimates of biodiversity change. Conservation Biology. 2021; 35(2): 688–698.
16. Jetz W, McPherson JM, Guralnick RP. Integrating biodiversity distribution knowledge: toward a global map of life. Trends in Ecology & Evolution. 2012; 27(3): 151–159.
17. Engemann K, Enquist BJ, Sandel B, Boyle B, Jørgensen PM, Morueta-Holme N, et al. Limited sampling hampers “big data” estimation of species richness in a tropical biodiversity hotspot. Ecology and Evolution. 2015; 5(3): 807–820.

18. Santini L, Antão LH, Jung M, Benítez-López A, Rapacciuolo G, Di Marco M, et al. The interface between macroecology and conservation: existing links and untapped opportunities. Frontiers of Biogeography. 2021; 13(4): e53025.
19. Diniz-Filho JAF, Jardim L, Guedes JJ, Meyer L, Stropp J, Frateles LEF, et al. Macroecological links between the Linnean, Wallacean, and Darwinian shortfalls. Frontiers of Biogeography, 2023; 15(2): e59566.
20. Hughes AC, Dorey JB, Bossert S, Qiao H, Orr MC. Big data, big problems? How to circumvent problems in biodiversity mapping and ensure meaningful results. Ecography. 2024; 2024(8): e07115.
21. Grattarola F, Martínez-Lanfranco JA, Botto G, Naya DE, Maneyro R, Mai P, et al. Multiple forms of hotspots of tetrapod biodiversity and the challenges of open-access data scarcity. Scientific Reports. 2020; 10(1): 22045.
22. Perino A, Pereira HM, Felipe-Lucia M, Kim H, Kühl HS, Marselle MR, et al. Biodiversity post-2020: Closing the gap between global targets and national-level implementation. Conservation Letters. 2021; 15(2): e12848.
23. Soberón J. Biodiversity informatics for public policy. The case of CONABIO in Mexico. Biodiversity Informatics. 2022; 17.
24. Berba CMP, Matias AMA. State of biodiversity documentation in the Philippines: Metadata gaps, taxonomic biases, and spatial biases in the DNA barcode data of animal and plant taxa in the context of species occurrence data. PeerJ. 2022; 10: e13146.
25. Meneses CG, Pitogo KME, Supsup CE, Brown RM. Philippine herpetology (Amphibia, Reptilia), 20 years on: two decades of progress towards an increasingly collaborative,

equitable, and inclusive approach to the study of the archipelago's amphibians and reptiles. ZooKeys. 2024; 1190, 213–257.

26. Pitogo KME. Gaps and biases in vertebrate wildlife genetics from a global biodiversity hotspot. Environmental Conservation. 2025; 52(3): 127–138.

27. Brown RM, Siler CD, Oliveros CH, Esselstyn JA, Diesmos AC, Hosner PA, et al. Evolutionary processes of diversification in a model island archipelago. Annual Review of Ecology, Evolution, and Systematics. 2013; 44(1): 411–435.

28. Huais PY, Osorio-Olvera L, Cordier JM, Tomba AN, Soberón J, Loyola R, Nori J. Rethinking global hotspots for threatened terrestrial vertebrates. Global Ecology and Biogeography. 2025; 34(1): e13942.

29. Soberón J, Peterson AT. Monitoring biodiversity loss with primary species-occurrence data: toward national-level indicators for the 2010 target of the convention on biological diversity. AMBIO: A Journal of the Human Environment. 2009; 38(1): 29–34.

30. CBD [Convention on Biological Diversity] [cited 01 August 2025]. Decision adopted by the Conference of the Parties to the Convention on Biological Diversity CBD/COP/DEC/15/4 Kunming-Montreal Global Biodiversity Framework. 2022; CBD/COP/DEC/15/4. Available from: https://www.cbd.int/doc/decisions/cop-15/cop-15-dec-04-en.pdf

31. Orr MC, Hughes AC, Costello MJ, Qiao H. Biodiversity data synthesis is critical for realizing a functional post-2020 framework. Biological Conservation. 2022; 274: 109735.

32. ASEAN Centre for Biodiversity. ASEAN Biodiversity Outlook 3. Philippines, pp. 392; 2023.

33. Maxwell SL, Cazalis V, Dudley N, Hoffmann M, Rodrigues AS, Stolton S, et al. Area-based conservation in the twenty-first century. Nature. 2020; 586(7828): 217–227.
34. Mallari NAD, Collar NJ, McGowan PJ, Marsden SJ. Science-driven management of protected areas: a Philippine case study. Environmental Management. 2013; 51(6): 1236–1246.
35. Jetz W, McGeoch MA, Guralnick R, Ferrier S, Beck J, Costello MJ, et al. Essential biodiversity variables for mapping and monitoring species populations. Nature Ecology & Evolution. 2019; 3(4): 539–551.
36. Hochkirch A, Samways MJ, Gerlach J, Böhm M, Williams P, Cardoso P, et al. A strategy for the next decade to address data deficiency in neglected biodiversity. Conservation Biology. 2021; 35(2): 502–509.
37. Urbano F, Viterbi R, Pedrotti L, Vettorazzo E, Movalli C, Corlatti L. Enhancing biodiversity conservation and monitoring in protected areas through efficient data management. Environmental Monitoring and Assessment. 2024; 196(1): 12.
38. Brown RM, Diesmos AC, Alcala AC. The state of Philippine herpetology and the challenges for the next decade. Silliman Journal. 2001; 42(1): 18–87.
39. Heaney LR. A decade of research on Philippine mammals: progress and challenges. Silliman Journal. 2001; 42(1): 88–107.
40. Pelser PB, Barcelona JF, Nickrent DL . Co's Digital Flora of the Philippines. Available from: www. philippineplants.org.
41. Heaney LR, Balete DS, Rickart EA. The mammals of Luzon Island: biogeography and natural history of a Philippine fauna. JHU Press; 2016.

42. Tanalgo KC, Hughes AC. Bats of the Philippine Islands—A review of research directions and relevance to national-level priorities and targets. Mammalian Biology. 2018; 91(1): 46–56.

43. Allen D. Birds of the Philippines. Lynx and Birdlife International Field Guides, Lynx Edicions, Barcelona, Spain; 2020.

44. Gamalo LE, Sabanal B, Ang A. Three decades of Philippine nonhuman primate studies: research gaps and opportunities for Philippine primatology. Primates. 2021; 62(1): 233–239.

45. delos Angeles MD, Radbouchoom S, Ngarega BK, Lapuz RS, Schneider H. Spatial diversity and distribution of fern and lycophyte species in karst and non-karst landscapes towards conservation needs. Frontiers in Plant Science. 2025; 16: 1495796.

46. Fernandez DAP, Do Linh San E, Forget PM, Veron G. Carnivorans of the Philippines: current knowledge and research gaps. Journal of Mammalogy. 2025; gyaf020.

47. Balisco RAT, Liao TY. An annotated checklist of fish species described from the Philippines. ZooKeys. 2025; 1246: 1–287.

48. Diesmos AC, Watters JL, Huron NA, Davis DR, Alcala AC, Crombie RI, et al. Amphibians of the Philippines, part I: checklist of the species. Proceedings of the California Academy of Sciences. 2015; 62(20): 457–539.

49. Leviton AE, Siler CD, Weinell JL, Brown RM. Synopsis of the snakes of the Philippines. Proceedings of the California Academy of Sciences. 2018; 64(14): 399–568.

50. Fidelino JS, Constantino RRP, Duya MRM. Cross-taxon congruence of terrestrial vertebrates across Philippine Key Biodiversity Areas. Journal for Nature Conservation. 2025; 86: 126907.

51. Mallari NAD, Collar NJ, McGowan PJ, Marsden SJ. Philippine protected areas are not meeting the biodiversity coverage and management effectiveness requirements of Aichi Target 11. Ambio. 2016; 45(3): 313–322.

52. Struebig MJ, Lee JS, Deere NJ, Gevaña DT, Ingram DJ, Lwin N. Drivers and solutions to Southeast Asia's biodiversity crisis. Nature Reviews Biodiversity. 2025;1: 497–514.

53. QGIS Development Team. QGIS Geographic Information System. Open Source Geospatial Foundation Project; 2018. Available from: http://qgis.osgeo.org.

54. Roll U, Feldman A, Novosolov M, Allison A, Bauer AM, Bernard R, et al. The global distribution of tetrapods reveals a need for targeted reptile conservation. Nature Ecology & Evolution. 2017; 1(11): 1677–1682.

55. Caetano GHDO, Chapple DG, Grenyer R, Raz T, Rosenblatt J, Tingley R, et al. Automated assessment reveals that the extinction risk of reptiles is widely underestimated across space and phylogeny. PLoS Biology. 2022; 20(5): e3001544.

56. Brown RM, Siler CD, Richards SJ, Diesmos AC, Cannatella DC. Multilocus phylogeny and a new classification for Southeast Asian and Melanesian forest frogs (family Ceratobatrachidae). Zoological Journal of the Linnean Society. 2015; 174(1): 130–168.

57. Sanguila MB, Cobb KA, Siler CD, Diesmos AC, Alcala AC, Brown RM. The amphibians and reptiles of Mindanao Island, southern Philippines, II: the herpetofauna of northeast Mindanao and adjacent islands. ZooKeys. 2016; (624), 1–132.

58. Pitogo KME, Saavedra AJL, Afuang LE. Amphibians and reptiles of Mount Busa, Sarangani Province: a glimpse of the herpetological community of southern Mindanao, Philippines. Philippine Journal of Science. 2021; 150(5): 1279–1306.

59. Nuñez-Penichet C, Cobos ME, Soberón J, Gueta T, Barve N, Barve V, Navarro-Sigüenza AG, Peterson AT. Selection of sampling sites for biodiversity inventory: Effects of environmental and geographical considerations. Methods in Ecology and Evolution. 2022; 00:1–13.

60. Ong PS, Afuang LE, Rosell-Ambal RG. Philippine Biodiversity Conservation Priorities: A second iteration of the National Biodiversity Strategy and Action Plan. Department of Environment and Natural Resources-Protected Areas and Wildlife Bureau, Conservation International Philippines, Biodiversity Conservation Program-University of the Philippines Center for Integrative and Development Studies, and Foundation for the Philippine Environment. Quezon City, Philippines; 2002.

61. R Core Team (2022). R: A language and environment for statistical computing. R Foundation for Statistical Computing, Vienna, Austria. Available from: https://www.R-project.org/.

62. Hijmans R._terra: Spatial Data Analysis_. R package version 1.7-78; 2025. Available from: https://CRAN.R-project.org/package=terra.

63. Ricklefs RE, Lovette IJ. The roles of island area per se and habitat diversity in the species–area relationships of four Lesser Antillean faunal groups. Journal of Animal Ecology. 1999; 68(6): 1142–1160.

64. Gotelli NJ. A Primer of Ecology, 4th ed. Sunderland, MA: Sinauer Associates; 2008.

65. Venables WN, Ripley BD. Modern Applied Statistics with S. Fourth Edition. Springer, New York. ISBN 0-387-95457-0; 2002.

66. Brooks ME, Kristensen K, Van Benthem KJ, Magnusson A, Berg CW, Nielsen A, et al. glmmTMB balances speed and flexibility among packages for zero-inflated generalized linear mixed modeling. The R Journal. 2017; 9(2): 378–400.
67. Zuur AF, Ieno EN, Walker N, Saveliev AA, Smith GM, Zuur AF, et al. GLM and GAM for count data. Mixed effects models and extensions in ecology with R, 209–243; 2009.
68. Hartig F. _DHARMa: Residual Diagnostics for Hierarchical (Multi-Level / Mixed) Regression Models_. R package version 0.4.7; 2024. Available from: https://CRAN.R-project.org/package=DHARMa.
69. Lüdecke D, Ben-Shachar MS, Patil I, Waggoner P, Makowski D. performance: An R Package for Assessment, Comparison and Testing of Statistical Models. Journal of Open Source Software. 2021; 6(60): 3139.
70. H. Wickham. ggplot2: Elegant Graphics for Data Analysis. Springer-Verlag New York; 2016.
71. Nuñeza OM, Ates FB, Alicante AA. Distribution of endemic and threatened herpetofauna in Mt. Malindang, Mindanao, Philippines. Biodiversity and Conservation. 2010; 19, 503–518.
72. Gonzalez JCT, de Guia APO, Dimalibot JC, Pantua KV, Gustilo WO, Bantayan NC. Understorey to canopy vertebrate fauna of a lowland evergreen forest in Mt. Makiling Forest Reserve, Philippines. Biodiversity Data Journal. 72; 8: e56999.
73. Brown WC, Alcala AC. Populations of amphibians and reptiles in the submontane and montane forests of Cuernos de Negros, Philippine Islands. Ecology. 1961; 628–636.
74. Brown WC, Alcala AC. The zoogeography of the Philippine Islands, a fringing archipelago. Proceedings of the California Academy of Sciences. 1970; 38: 105–130.

75. Brown RM, McGuire JA, Ferner JW, Icarangal Jr N, Kennedy RS. Amphibians and reptiles of Luzon Island, II: preliminary report on the herpetofauna of Aurora Memorial National Park, Philippines. Hamadryad. 2000; 25:175–195

76. Siler CD, Welton LJ, Siler JM, Brown J, Bucol A, Diesmos AC, Brown RM. Amphibians and Reptiles, Luzon Island, Aurora Province and Aurora Memorial National Park, Northern Philippines: New island distribution records. Check List. 2011; 7(2): 182–195.

77. Brown RM. A new species of stream frog of the genus *Hylarana* from the mountains of southern Mindanao Island, Philippines. Herpetologica. 2015; 71(3): 223–233.

78. Siler CD, Welton LJ, Diesmos AC, Siler JM, Bucol A, Brown J, Brown RM. Amphibians and reptiles, Romblon Island Group, central Philippines: comprehensive herpetofaunal inventory. Check List. 2012; 8(3): 443–462.

79. Meneses CG, Siler CD, Alviola PA, Gonzalez JCT, Balatibat JB, Natividad CA, Brown RM. Amphibian and reptile diversity along a ridge-to-reef elevational gradient on a small isolated oceanic island of the central Philippines. Check List. 2012; 18(5): 941–948.

80. Plaza JL, Sanguila MB. Preliminary report on the anurans of Mount Hilong-hilong, Agusan del Norte, eastern Mindanao, Philippines. Asian Herpetological Research. 2015; 6(1): 18–33.

81. Gojo-Cruz PHP, Afuang LE, Gonzalez JCT, Gruezo WS. Amphibians and reptiles of Luzon Island, Philippines: the herpetofauna of Pantabangan-Carranglan watershed, Nueva Ecija Province, Caraballo Mountain range. Asian Herpetological Research. 2018; 9(4): 201–223.

82. Supsup CE, Asis AA, Carestia Jr UV, Diesmos AC, Mallari NAD, Brown RM. Variation in species richness, composition and herpetological community structure across a tropical habitat gradient of Palawan Island, Philippines. Herpetozoa. 2020; 33: 95–111.

83. Azovsky AI. Species–area and species–sampling effort relationships: disentangling the effects. Ecography. 2011; 34(1): 18–30.

84. Pitogo KME, Meneses CG, Flores ABA, Saavedra AJL, Amarga AKS, Angeles MD, et al. Caught in statistical noise: pitfalls of a unidimensional approach to understanding biodiversity-conflict relationships. npj Biodiversity. 2025; 4(1): 17.

85. Brown RM, Meneses CG, Wood Jr PL, Fernandez JB, Cuesta MA, Clores MA, et al. Unexpected discovery of another new species of Philippine false gecko (Gekkonidae; *Pseudogekko*) from the Bicol Peninsula of Luzon Island. Herpetologica. 2020; 76(3): 315–329.

86. Di Minin E, Toivonen T. Global protected area expansion: creating more than paper parks. BioScience. 2015; 65(7): 637–638.

87. Watson JE, Dudley N, Segan DB, Hockings M. The performance and potential of protected areas. Nature. 2014; 515(7525): 67–73.

88. Jonas HD, Ahmadia GN, Bingham HC, Briggs J, Butchart SHM, Carino J, Chassot O, Chaudhary S, et al. Equitable and effective areas-based conservation: Towards the conserved area paradigm. *PARKS*. 2021; 21: 71–84.

89. Eken G, Bennun L, Brooks TM, Darwall W, Fishpool LD, Foster M, et al. Key biodiversity areas as site conservation targets. BioScience. 2004; 54(12): 1110–1118.

90. Hoffmann S. Challenges and opportunities of area-based conservation in reaching biodiversity and sustainability goals. Biodiversity and Conservation. 2022; 31(2): 325–352.

91. Tenorio EA, Montoya P, Norden N, Rodríguez-Buriticá S, Salgado-Negret B, Gonzalez MA. Mountains exhibit a stronger latitudinal diversity gradient than lowland regions. Journal of Biogeography. 2023; 50(6): 1026–1036.

92. Pitogo KME, Saavedra AJ. Conservation assessment of the recently rediscovered Guttman's Stream Frog, *Pulchrana guttmani* (Brown, 2015), reveals the need for its uplisting into a higher threat category. Herpetology Notes. 2023; 16: 717–721.

93. Heaney LR. Conservation biogeography in oceanic archipelagoes. Frontiers of Biogeography, New Directions in the Geography of Nature. Sinauer Associates, Sunderland, MA, 345–360; 2004.

94. McGuire JA, Alcala AC. A taxonomic revision of the flying lizards (Iguania: Agamidae: *Draco*) of the Philippine Islands, with a description of a new species. Herpetological Monographs. 2000; 14: 81–138.

95. Allen D, Oliveros C, Española C, Broad G, Gonzalez JCT. A new species of *Galliralus* from Calayan Island, Philippines. Forktail. 2004; 20: 1–7.

96. Heaney LR, Tabaranza BR, Balete DS, Rigertas N. Synopsis and biogeography of the mammals of Camiguin Island, Philippines. Fieldiana Zoology. 2006; 2006(106): 28–48.

97. Brown RM, Siler CD, Oliveros CH, Diesmos AC, Alcala AC. A new *Gekko* from Sibuyan Island, central Philippines. Herpetologica. 2011; 67(4), 460–476.

98. Oliveros CH, Ota H, Crombie RI, Brown RM. The herpetofauna of the Babuyan group of islands, northern Philippines. Scientific Publications of the Natural History Museum of the University of Kansas. 2011; 43: 1–20.

99. Reyes MC, Ingicco T, Piper PJ, AmanoN, Pawlik AF. First fossil evidence of the extinct Philippine cloud rat *Crateromys paulus* (Muridae: Murinae: Phloeomyini) from Ilin Island, Mindoro, and insights into its Holocene abundance. Proceedings of the Biological Society of Washington. 2017; 130(1): 84–97.

100. Barley AJ, Diesmos AC, Siler CD, Martinez CM, Brown RM. Taxonomic revision of Philippine Sun Skinks (Reptilia: Squamata: Scincidae: *Eutropis*), and descriptions of eight new species. Herpetological Monographs. 2020; 34(1): 39–70.

101. Clores MA, Bautista JB, Fernandez JB, Cuesta MA, Brown RM. Diversity and distribution of amphibians and reptiles in the Caramoan Island Group, Maqueda Channel, southern Luzon, Philippines. Journal of Asia-Pacific Biodiversity. 2021; 14(1): 1–14.

102. Supsup CE, Asis AA, Carestia UV, Puna NM, Avanceña JB. Geographic distribution, population and conservation status of *Tragulus nigricans*, an endangered mouse deer endemic to western Philippines. Pacific Conservation Biology. 2021; 28(1): 90–96.

103. Russell JC, Kueffer C. Island biodiversity in the Anthropocene. Annual Review of Environment and Resources. 2019; 44(1): 31–60.

104. Borges PA, Cardoso P, Gabriel R, Ah-Peng C, Emerson BC. Challenges, advances and perspectives in Island Biogeography. Frontiers of Biogeography. 2016; 8(2).

105. Blades B, Ronquillo C, Hortal J. Mobilisation of data from natural history collections can increase the quality and coverage of biodiversity information. Ecology and Evolution. 2025; 15(4): e71139.

106. Heberling JM, Miller JT, Noesgaard D, Weingart SB, Schigel D. Data integration enables global biodiversity synthesis. Proceedings of the National Academy of Sciences. 2021; 118(6): e2018093118.

107. Amano T, Lamming JD, Sutherland WJ. Spatial gaps in global biodiversity information and the role of citizen science. Bioscience. 2016; 66(5): 393–400.

108. Della Rocca F, Musiani M, Galaverni M, Milanesi P. Improving online citizen science platforms for biodiversity monitoring. Journal of Biogeography. 108; 51(12): 2412–2423.

109. Mason BM, Mesaglio T, Barratt Heitmann J, Chandler M, Chowdhury S, Gorta SBZ, et al. iNaturalist accelerates biodiversity research. BioScience. 2025; biaf104.

110. Barve V. Discovering and developing primary biodiversity data from social networking sites: A novel approach. Ecological Informatics. 2014; 24: 194–199.

111.Chowdhury S, Aich U, Rokonuzzaman M, Alam S, Das P, Siddika A, et al. Increasing biodiversity knowledge through social media: A case study from tropical Bangladesh. BioScience. 2023; 73(6): 453–459.

112. Tabeta MSG, Bejar SGF. Improving our understanding of a cryptic primate, the Philippine Tarsier (*Carlito syrichta*), through social media. International Journal of Primatology. 2025; 1–26.

113. Madera BS. Population survey of marine turtles in Boracay Island, Philippines using a citizen science approach. Journal of Management and Development Studies. 2019; 8(2): 1–15.

114. Acuña D, Isagani N, Pitogo KME. Predation on a McClung’s Philippine false coralsnake, *Hemibungarus mcclungi* (Weigmann 1835), by a giant spiny centipede,

Scolopendra spinosissima Kraepelin 1903, on Luzon Island, The Philippines. Reptiles & Amphibians. 2021; 28(3): 417–419.

115. Sullivan BL, Aycrigg JL, Barry JH, Bonney RE, Bruns N, Cooper CB, et al. The eBird enterprise: An integrated approach to development and application of citizen science. Biological Conservation. 2014; 169: 31–40.

116. Pitogo KME, Jarvis F, Saavedra AJL, Senarillos TL, Concepcion CB, Simpson PM. Avian fauna of Mount Melibengoy, southern Mindanao, Philippines: conservation implications on a partially protected key biodiversity area under threat. Philippine Journal of Systematic Biology Online ISSN. 2024; 17: 29–40.

117. Barcelona JF, Nickrent DL, LaFrankie JV, Callado JRC, Pelser PB. Co's digital flora of the Philippines: Plant identification and conservation through cybertaxonomy. Philippine Journal of Science. 2013; 142: 57–67.

118. Pernat N, Canavan S, Golivets M, Hillaert J, Itescu Y, Jarić I, et al. Overcoming biodiversity blindness: Secondary data in primary citizen science observations. Ecological Solutions and Evidence. 2024; 5(1): e12295.

119. Binaday JWB, Amarga AKS, Barrameda ES, Bonagua BJM. Amphibians and reptiles in the vicinity of Bulusan lake, Bulusan Volcano natural park, Sorsogon, Philippines. Philippine Journal of Science. 2017; 146(3): 339–351.

120. Decena SCP, Macasait Jr DR, Arguelles MS. Species Accounts, assemblage, and microhabitats of amphibians and reptiles of Northeastern Leyte, Philippines. Philippine Journal of Science. 2023; 152(1).

121. Maglangit EPT, Nuñeza OM, Coritico FP, Medecilo-Guiang MMP, Mohagan AB, Patano Jr RR, Amoroso VB. Richness and distribution of reptiles and amphibians in the

tropical lowland habitats of Mt. Agad-Agad, Iligan City, Southern Philippines. Reptiles & Amphibians. 2022; 29(1): 413–425.

122. Beck J, Ballesteros-Mejia L, Nagel P, Kitching IJ. Online solutions and the 'Wallacean shortfall': what does GBIF contribute to our knowledge of species' ranges?. Diversity and Distributions. 2013; 19(8): 1043–1050.

123. Cadotte MW, Beyene MS, Bowell M, Dalrymple SE, Dechoum MS, Dooner P, et al. Grey matters: Ensuring management information is a part of the permanent evidence base by creating open grey literature principles. Ecological Solutions and Evidence. 2025; 6(2).

124. Wieczorek J, Bloom D, Guralnick R, Blum S, Döring M, Giovanni R, et al. Darwin Core: an evolving community-developed biodiversity data standard. PloS one. 2012; 7(1): e29715.

125. Ball-Damerow JE, Brenskelle L, Barve N, Soltis PS, Sierwald P, Bieler R, et al. Research applications of primary biodiversity databases in the digital age. PloS one. 2019; 14(9): e0215794.

126. Marques N, de Melo Soares CD, de Melo Casali D, Guimarães EC, Fava FG, da Silva Abreu JM, et al. Retrieving biodiversity data from multiple sources: making secondary data standardised and accessible. Biodiversity Data Journal. 2024; 12: e133775.

127. Costello MJ, Wieczorek J. Best practice for biodiversity data management and publication. Biological Conservation. 2014; 173: 68–73.

128. Robinson JG, LaBruna D, O'Brien T, Clyne PJ, Dudley N, Andelman SJ, et al. Scaling up area-based conservation to implement the Global Biodiversity Framework's 30x30 target: The role of Nature's Strongholds. PLoS Biology. 2024; 22(5): e3002613.

129. Buckland ST, Johnston A. Monitoring the biodiversity of regions: Key principles and possible pitfalls. Biological Conservation. 2917; 214: 23–34.

130. Wenk E, Mesaglio T, Keith D, Cornwell W. Curating protected area-level species lists in an era of diverse and dynamic data sources. Ecological Informatics. 2024; 84: 102921.

131. Britz R, Hundsdörfer A, Fritz U. Funding, training, permits—the three big challenges of taxonomy. Megataxa. 2020; 1(1): 49–52.

132. Dawson NM, Coolsaet B, Bhardwaj A, Booker F, Brown D, Lliso B, et al. Is it just conservation? A typology of Indigenous peoples’ and local communities’ roles in conserving biodiversity. One Earth. 2024; 7(6): 1007–1021.

133. Abrahamse T, Andrade-Correa MG, Arida C, Galsim R, Häuser C, Price M, Sommerwerk N. The Global Taxonomy Initiative in Support of the Post-2020 Global Biodiversity Framework. CBD Technical Series No. 96. Secretariat of the Convention on Biological Diversity, Montreal, 103 pages; 2021.

134. Soberón J, Peterson AT. Biodiversity informatics: managing and applying primary biodiversity data. Philosophical Transactions of the Royal Society of London. Series B: Biological Sciences. 2004; 359(1444): 689–698.

135. Güntsch A, Overmann J, Ebert B, Bonn A, Le Bras Y, Engel T, et al. National biodiversity data infrastructures: ten essential functions for science, policy, and practice. BioScience. 2025; 75(2): 139–151.

136. Colella JP, Silvestri L, Súzan G, Weksler M, Cook JA, Lessa EP. Engaging with the Nagoya Protocol on Access and Benefit-Sharing: recommendations for noncommercial biodiversity researchers. Journal of Mammalogy. 2023; 104(3): 430–443.

137. DENR-BMB [Department of Environment and Natural Resources - Biodiversity Management Bureau]. Philippine Biodiversity Strategy and Action Plan (Abridged). Department of Environment and Natural Resources - Biodiversity Management Bureau, Quezon City, Metro Manila, 26 pp; 2025.

138. Schmeller DS, Böhm M, Arvanitidis C, Barber-Meyer S, Brummitt N, Chandler M, et al. Building capacity in biodiversity monitoring at the global scale. Biodiversity and Conservation. 2017; 26: 2765–2790.Carroll, S. R., Herczog, E., Hudson, M., Russell, K., & Stall, S. (2021). Operationalizing the CARE and FAIR Principles for Indigenous data futures. *Scientific Data*, 8(1): 108.

139. Carroll SR, Herczog E, Hudson M, Russell K, Stall S. Operationalizing the CARE and FAIR Principles for Indigenous data futures. Scientific Data. 2021; 8(1): 108.

Supporting Information

S1 Fig. Model diagnostic results from DHARMa for the best-fit species diversity model for conserved areas (Species Diversity ~ log_Area + log_Occurrence + sqrt_Topo_Relief + log_Area:sqrt_Topo_Relief). Upper-right panel shows a Q–Q plot used to detect deviations from the expected distribution, along with tests for distribution (Kolmogorov–Smirnov), dispersion, and outliers. Additional panels show scaled residuals plotted against predicted values and each predictor. Simulation outliers, data points falling outside the range of simulated values, are highlighted as red stars. Red line indicates statistically significant deviations from model expectations. DHARMa zero-inflation test is also included.

S2 Fig. Model diagnostic results from DHARMa for the best-fit species diversity model for key biodiversity areas (Species Diversity ~ log_Area + log_Occurrence + sqrt_Topo_Relief + log_Area:log_Occurrence + log_Area:sqrt_Topo_Relief + log_Occurrence:sqrt_Topo_Relief). Upper-right panel shows a Q–Q plot used to detect deviations from the expected distribution, along with tests for distribution (Kolmogorov–Smirnov), dispersion, and outliers. Additional panels show scaled residuals plotted against predicted values and each predictor. Simulation outliers, data points falling outside the range of simulated values, are highlighted as red stars. Red line indicates statistically significant deviations from model expectations. DHARMa zero-inflation test is also included.

S3 Fig. Model diagnostic results from DHARMa for the best-fit sampling effort model for conserved areas (No. of Occurrence Records ~ log_Area × sqrt_Topo_Relief + PAIC). Upper-right panel shows a Q–Q plot used to detect deviations from the expected distribution, along with tests for distribution (Kolmogorov–Smirnov), dispersion, and outliers. Additional panels show scaled residuals plotted against predicted values and each predictor. Simulation outliers, data points falling outside the range of simulated values, are highlighted as red stars. Red line indicates statistically significant deviations from model expectations. DHARMa zero-inflation test is also included.

S4 Fig. Model diagnostic results from DHARMa for the best-fit sampling effort model for key biodiversity areas (No. of Occurrence Records ~ log_Area × sqrt_Topo_Relief + PAIC). Upper-right panel shows a Q–Q plot used to detect deviations from the expected

distribution, along with tests for distribution (Kolmogorov–Smirnov), dispersion, and outliers. Additional panels show scaled residuals plotted against predicted values and each predictor. Simulation outliers, data points falling outside the range of simulated values, are highlighted as red stars. Red line indicates statistically significant deviations from model expectations. DHARMa zero-inflation test is also included.

S5 Fig. Map of the Philippines showing (A) overlap between forest cover, priority conservation areas (PCAs), and key biodiversity areas (KBAs), and (B) distribution of observed amphibian and squamate reptile diversity across all data types outside PCAs and KBAs, highlighting that many well-sampled sites with higher observed diversity lie beyond established conservation areas.

S1 Table. Stepwise model selection using AIC for species richness as a function of area, occurrence density, and topographic relief (species diversity model for conserved areas). The starting model included all main effects and their three-way interaction. The final model retained only two main effects and one two-way interaction.

S2 Table. Stepwise model selection using AIC for species richness as a function of area, occurrence density, and topographic relief (species diversity model for key biodiversity areas). The starting model included all main effects and their three-way interaction. The final model retained all main effects and two-way interactions, excluding the three-way interaction.

S3 Table. Stepwise model selection using AIC for occurrence count as a function of area, topographic relief, and biogeographic subregion or PAIC (sampling effort model for conserved areas). The starting model included a two-way interaction between area and relief, and an additive effect of PAIC level.

S4 Table. Stepwise model selection using AIC for occurrence count as a function of area, topographic relief, and biogeographic subregion or PAIC (sampling effort model for key biodiversity areas). The starting model included a two-way interaction between area and relief, and an additive effect of PAIC level.

Figure 1 Click here to access/download;Figure;Fig 1.tif

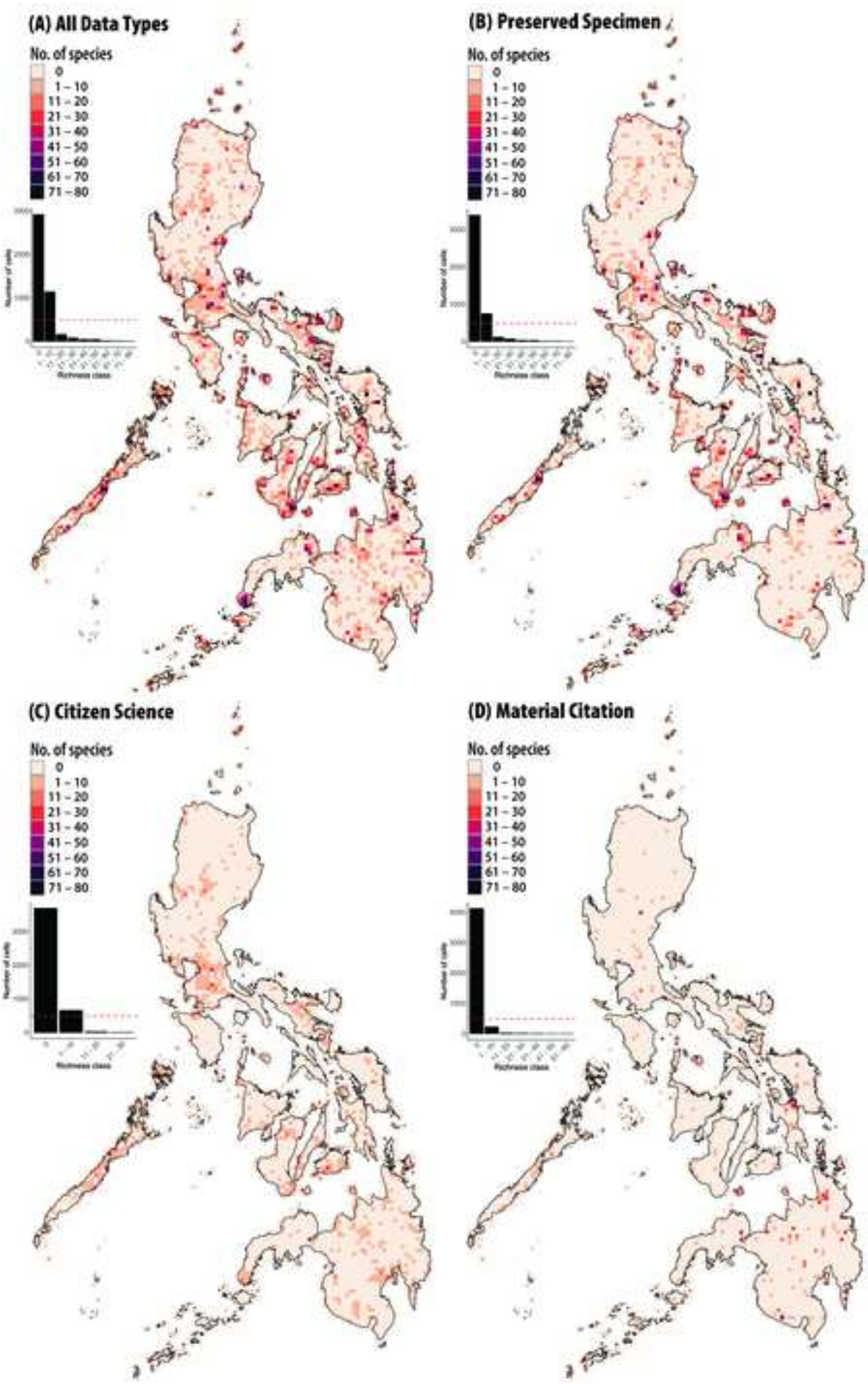

Figure 2

Click here to access/download;Figure;Fig 2.tif

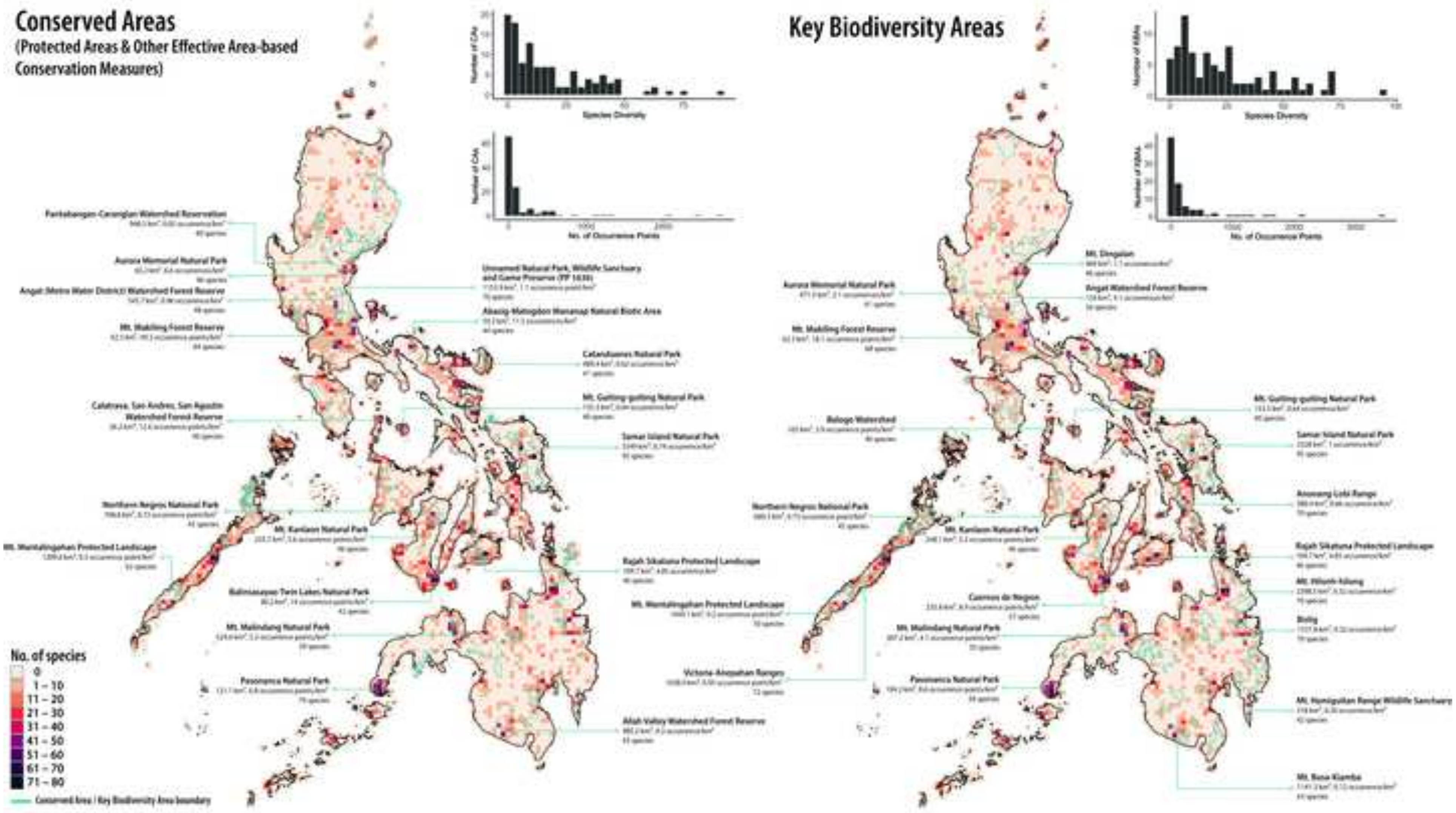

Figure 3

Click here to access/download;Figure;Fig 3.tif

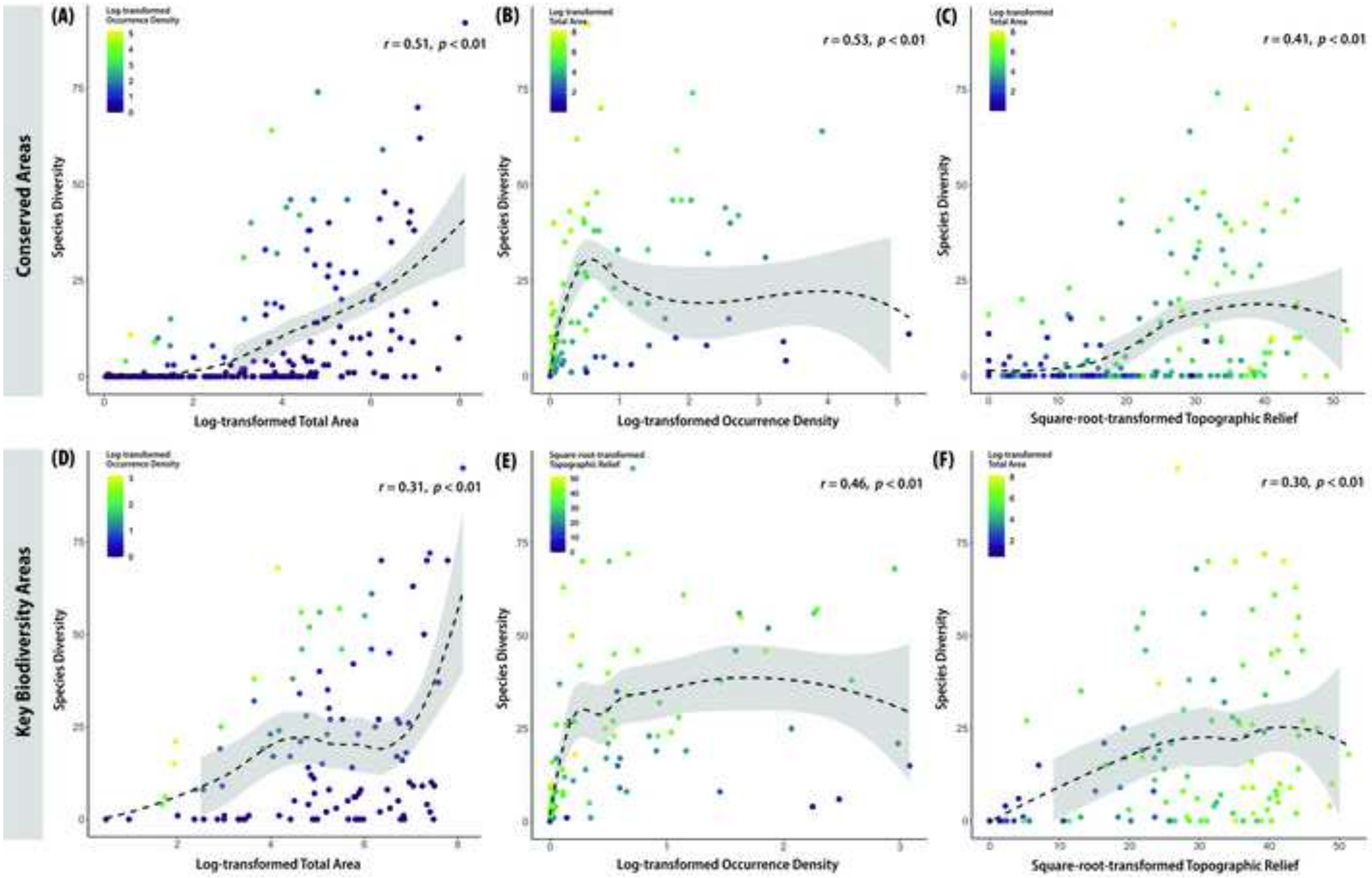

Figure 4

Click here to access/download;Figure;Fig 4.tif

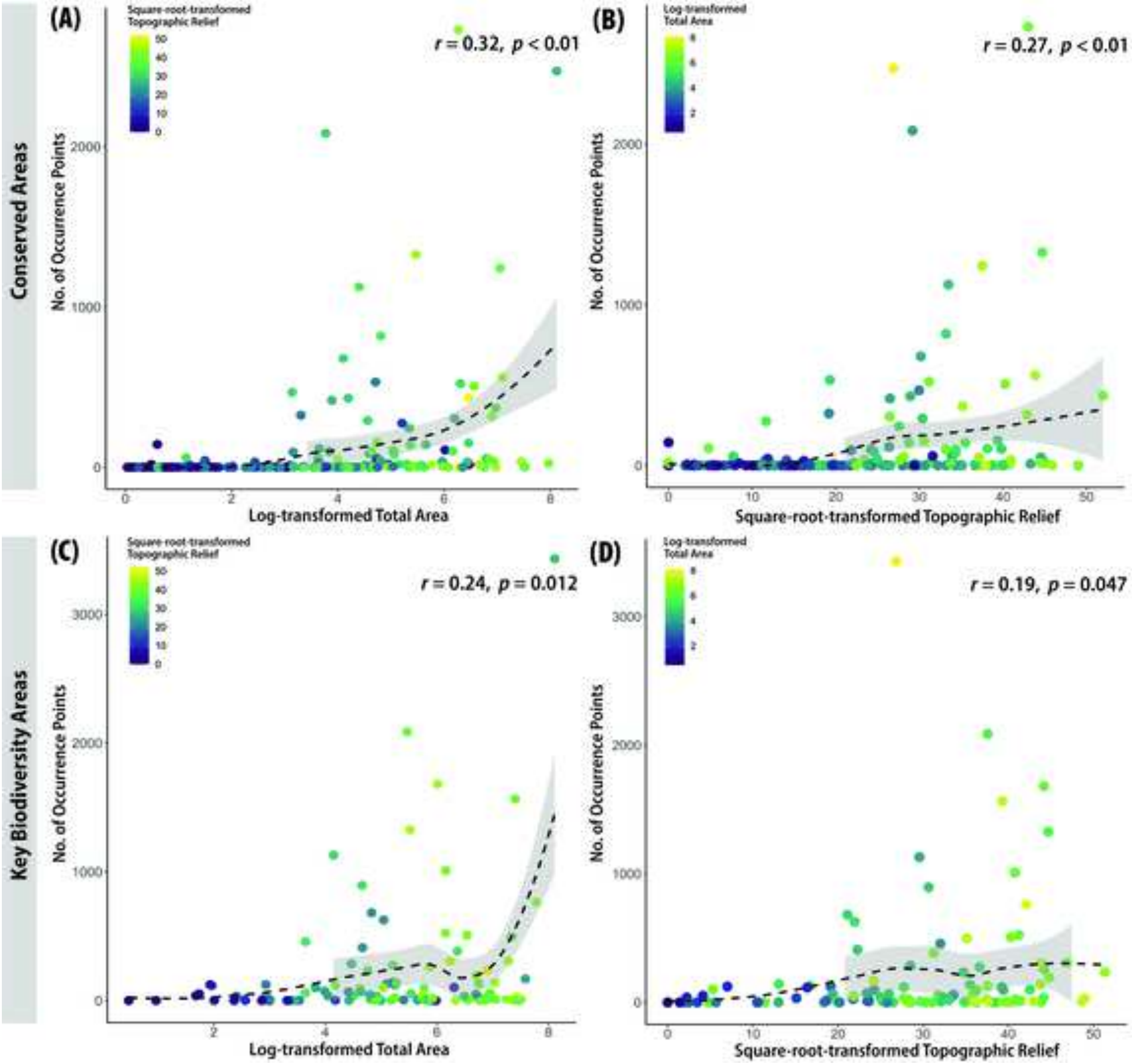

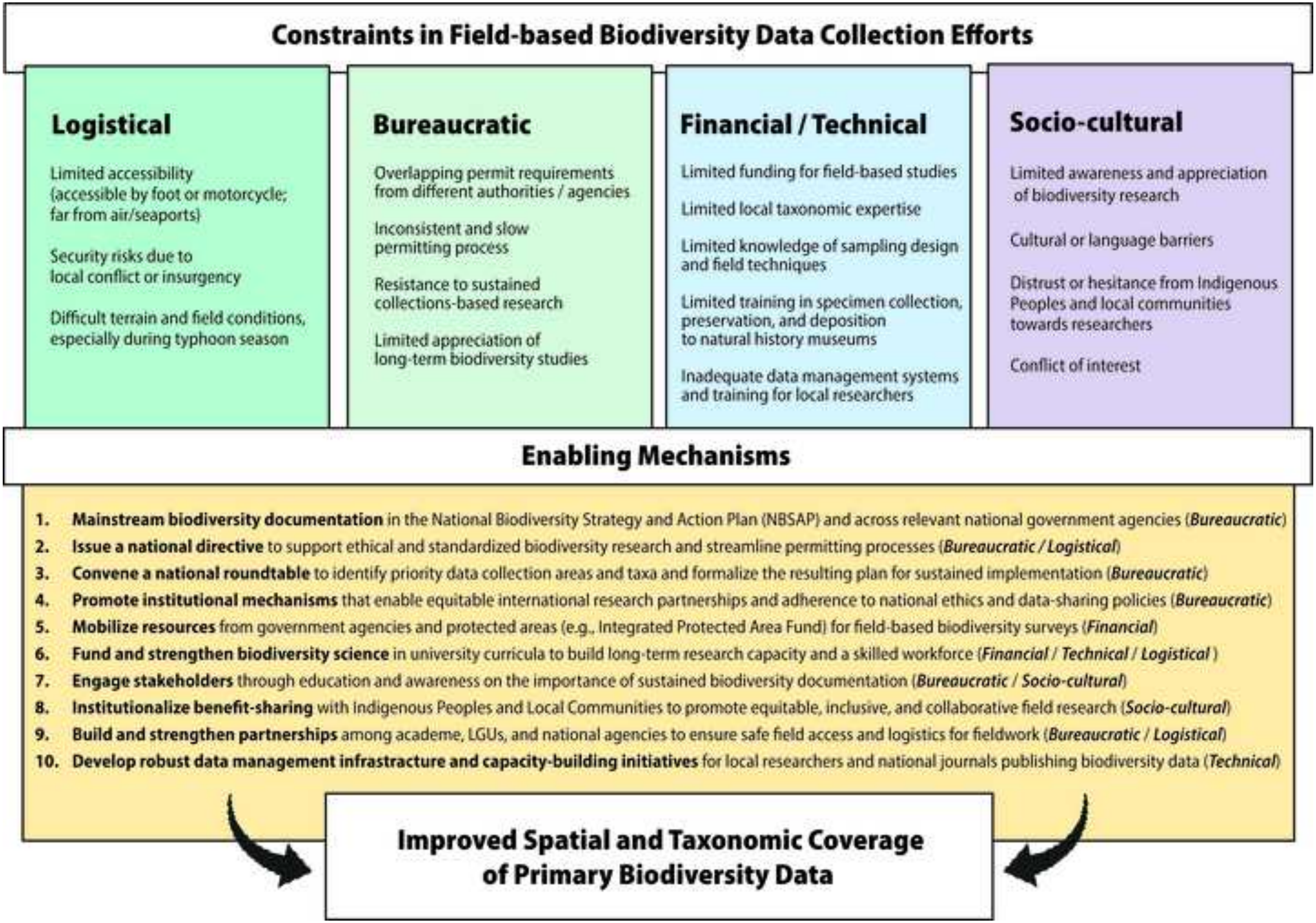
Constraints in Field-based Biodiversity Data Collection Efforts
Logistical
Limited accessibility (accessible by foot or motorcycle; far from air/seaports)
Security risks due to local conflict or insurgency
Difficult terrain and field conditions, especially during typhoon season
Bureaucratic
Overlapping permit requirements from different authorities / agencies
Inconsistent and slow permitting process
Resistance to sustained collections-based research
Limited appreciation of long-term biodiversity studies
Financial / Technical
Limited funding for field-based studies
Limited local taxonomic expertise
Limited knowledge of sampling design and field techniques
Limited training in specimen collection, preservation, and deposition to natural history museums
Inadequate data management systems and training for local researchers
Socio-cultural
Limited awareness and appreciation of biodiversity research
Cultural or language barriers
Distrust or hesitance from Indigenous Peoples and local communities towards researchers
Conflict of interest
Enabling Mechanisms
1. Mainstream biodiversity documentation in the National Biodiversity Strategy and Action Plan (NBSAP) and across relevant national government agencies (Bureaucratic)
2. Issue a national directive to support ethical and standardized biodiversity research and streamline permitting processes (Bureaucratic / Logistical)
3. Convene a national roundtable to identify priority data collection areas and taxa and formalize the resulting plan for sustained implementation (Bureaucratic)
4. Promote institutional mechanisms that enable equitable international research partnerships and adherence to national ethics and data-sharing policies (Bureaucratic)
5. Mobilize resources from government agencies and protected areas (e.g., Integrated Protected Area Fund) for field-based biodiversity surveys (Financial)
6. Fund and strengthen biodiversity science in university curricula to build long-term research capacity and a skilled workforce (Financial / Technical / Logistical)
7. Engage stakeholders through education and awareness on the importance of sustained biodiversity documentation (Bureaucratic / Socio-cultural)
8. Institutionalize benefit-sharing with Indigenous Peoples and Local Communities to promote equitable, inclusive, and collaborative field research (Socio-cultural)
9. Build and strengthen partnerships among academe, LGUs, and national agencies to ensure safe field access and logistics for fieldwork (Bureaucratic / Logistical)
10. Develop robust data management infrastructure and capacity-building initiatives for local researchers and national journals publishing biodiversity data (Technical)
Improved Spatial and Taxonomic Coverage of Primary Biodiversity Data

Click here to access/download
Supporting Information
Supplementary Files.docx

Data Review URL

Click here to download Data Review URL
http://zenodo.org/records/17344465
