# Supplementary material for: Sampling bias and uneven data coverage shape observed biodiversity patterns in a megadiverse island archipelago hotspot
Source: PLoS One. 2026 Jul 17;21(7):e0353393. doi: 10.1371/journal.pone.0353393 (PMC13379091; doi:10.1371/journal.pone.0353393)
Supplement: Attachment — Submitted filename: PONE-D-25-55790_R1.pdf [file pone.0353393.s007.pdf]

PLOS One

Sampling bias obscures biodiversity patterns, reveals data gaps in a megadiverse island archipelago hotspot

--Manuscript Draft--

**Manuscript Number:**	PONE-D-25-55790R1
**Article Type:**	Research Article
**Full Title:**	Sampling bias obscures biodiversity patterns, reveals data gaps in a megadiverse island archipelago hotspot
**Short Title:**	Biodiversity sampling gaps and biases in a tropical biodiversity hotspot
**Corresponding Author:**	Kier Mitchel E. Pitogo<br>The University of Kansas<br>Lawrence, Kansas UNITED STATES OF AMERICA
**Keywords:**	Philippines, biodiversity data, knowledge shortfalls, protected areas, mountains, macroecology, conservation
**Abstract:**	Where and how species are sampled can shape biodiversity knowledge, spatial patterns, and data-driven conservation. In many Global South megadiverse, biodiversity hotspots, sampling remains uneven, and available data often lack the synthesis needed to assess region-wide gaps for effective conservation planning and priority-setting. This shortfall is common within areas considered important for biodiversity. We demonstrate this case in the Philippines, one of the world's most biodiverse countries, where longstanding research and growing policy momentum support expansion of these conservation-relevant areas (hereafter "Philippine conservation-relevant areas" or PCAs). Using over a century of species occurrence records made digitally accessible, which we compiled and manually curated from multiple sources, we analyzed country-wide spatial distribution of observed amphibian and squamate reptile species richness. We also tested whether species richness across PCAs is explained by area, topographic relief, and sampling effort, and whether effects vary between PCA type: protected and conserved areas (with management or governance schemes) and key biodiversity areas (unmanaged, but priority areas for conservation). Results reveal strong spatial biases, with preserved specimens comprising 92% of all records and largely shaping species richness patterns. Citizen-science data reinforce already well-sampled regions, whereas peer-reviewed literature provides valuable documentation in undersampled areas. PCAs are proportionally well-sampled, with sampling effort and species richness increasing with area. However, this positive area effect weakens with increasing topographic relief, which may suggest that large mountain ranges remain documentation blind spots. The species–area relationship and the influence of sampling effort are stronger in protected and conserved areas, whereas topographic relief exerts a stronger effect in key biodiversity areas. Notably, several high-richness areas occur outside established protected areas. Our findings highlight sampling gaps and biases and underscore the value of integrating multiple data sources. We outline enabling mechanisms to strengthen primary biodiversity data collection, guiding future surveys toward undersampled regions.
**Order of Authors:**	Kier Mitchel E. Pitogo
	Camila G. Meneses
	Syrus Cesar P. Decena
	Christian E. Supsup
	Hannah E. Som
	Justin M. Bernstein
	Kin Onn Chan
	Mark William Herr
	Rafe Brown

Powered by Editorial Manager® and ProduXion Manager® from Aries Systems Corporation

Response to Reviewers:	EDITOR<br><br>Editor: A first overarching issue, raised primarily by Reviewer 1, concerns the alignment between research questions and analytical choices. Although the research questions are clearly stated, the links between each question, the statistical models used, and the corresponding results are not always explicit. I strongly suggest that the revised manuscript make these connections clear and systematic. Each main question should be directly associated with the analysis used to address it and with the relevant results, as this alignment is essential for transparency and interpretability.<br><br>Response: We have revised the phrasing of Questions 3 and 4 to better align with the analyses conducted. In the Methods section, we now state each specific objective before describing the corresponding analytical approach to clarify the connection between the questions and the analyses. The Results section also follow the same structure.<br><br>Editor: A major methodological concern, discussed in detail by Reviewer 1 and reinforced by Reviewer 2, relates to the framework used to compare different categories of conservation areas. Much of the manuscript's inference depends on comparing sampling effort and diversity patterns across conservation area types, yet the current analytical approach does not allow formal statistical testing of differences among these categories. I agree with both reviewers that this represents a substantive limitation. For the revision to be satisfactory, these comparisons should be conducted within a unified modeling framework that allows direct statistical evaluation of differences in effects across conservation area types.<br><br>Response: We reanalyzed the dataset using a joint modeling framework in which species richness was modeled as a function of area, topographic relief, and occurrence density, including all pairwise interactions among these predictors and their interactions with PCA type (protected and conserved areas vs. key biodiversity areas). Biogeographic subregion was included as a random intercept to account for non-independence among PCAs due to shared biogeographic structure. Results were broadly consistent with our previous approach. However, we retain this unified model as it more directly addresses our specific questions, as recommended by the reviewers as well.<br><br>Editor: Reviewer 1 emphasizes the importance of retaining model components that are justified by the study design, particularly grouping or hierarchical variables included to account for non-independence among observations.<br><br>Response: In our new models, we retained the biogeographic subregion (Pleistocene Aggregate Island Complexes or PAIC) as a random effect. Variance explained for the random effect are also reported in the results section, alongside the coefficient estimates of the fixed predictors.<br><br>Editor: The spatial properties of the dataset constitute another key issue raised by both reviewers from complementary perspectives. While reviewer 1 focuses on spatial precision, including how coordinate uncertainty was handled and whether the chosen grid resolution is appropriate given the characteristics of the occurrence records, the reviewer 2 emphasizes the need to explicitly address spatial dependence among observations. Given the spatially explicit nature of the analyses, I agree that these issues cannot be treated as secondary. The revised manuscript should clearly justify the analytical scale, address positional uncertainty, and either incorporate spatial structure into the models or provide a clear and defensible rationale for not doing so.<br>Response: We thank both Reviewers for highlighting the inherent challenges of spatially explicit analyses. In response to Reviewer 1, we have clarified in the revised manuscript our rationale for the selected 10-km grid resolution (lines 200–204) and our treatment of coordinate uncertainty (lines 184–190). The choice of a 10-km grid resolution was deliberate and intended to provide an appropriate balance for visualizing broad-scale spatial patterns of observed species richness patterns across the Philippines.<br>Regarding spatial uncertainty, we retained coordinates that were consistent with general (provincial-level) locality information, as these adequately reflect broad geographic distribution patterns (country-wide assessment). Our sampling is broad

enough and has a level of certainty where broad patterns will be discovered. Although minor spatial imprecision may influence species richness estimates within individual PCAs used in the modeling, any resulting bias is expected to be minimal because most PCAs are supported by numerous occurrence records, species are represented by multiple points within PCAs due to the integration of multiple data sources, and the overall sample size is large (n = 316 PCAs).
Editor: Both reviewers also identify limitations in the biological interpretation of the results, particularly regarding the joint treatment of amphibians and reptiles. These groups differ in ecology, detectability, sampling intensity, and conservation context. I suggest that the authors either explicitly assess whether these taxa exhibit similar or contrasting patterns, or clearly justify their combined analysis. Without this clarification, the ecological interpretation of the results remains incomplete.

Response: We acknowledge that amphibians and reptiles differ in their ecology and diversity patterns. However, as stated in the Introduction, these groups were used in this study because, relative to other taxonomic groups in the Philippines, they are comparatively well-documented and thus provide a robust basis for assessing observed biodiversity patterns and sampling distribution across areas. Our objective was not to model amphibian and reptile diversity separately or to compare their biological drivers (e.g., fine-scale environmental variables), but rather to evaluate how observed species richness patterns relate to area, sampling effort, and topographic relief in the context of biodiversity documentation or conservation planning. Accordingly, our interpretation focuses on spatial patterns of documented diversity rather than taxon-specific ecological differences. In addition, amphibians and reptiles have historically and contemporarily been surveyed and analyzed jointly in the Philippines (see Meneses et al. 2024), making their combined treatment, at least within the context of this paper, consistent with established research practice.

Editor: Further clarification is also required regarding the conceptual framing of conservation areas. Reviewer 2 raises questions about how different conservation categories are defined and compared, while Reviewer 1 highlights the difficulty of interpreting area-based comparisons under strong and uneven sampling biases. I agree with both assessments and recommend that the revised manuscript explicitly state the assumptions underlying these comparisons and clearly articulate how results should be interpreted in light of known data limitations.

Response: We recognize that the conceptual framing and terminology of conservation areas required consistency in the original submission. Following the recommendation of the Reviewer, we adopted the lexicon proposed by Salafsky et al. (2024), distinguishing between protected and conserved areas (i.e., areas with formal management or governance) and key biodiversity areas (i.e., spatial prioritization tools without inherent governance or protection status). In the revised manuscript, we refer to both collectively as Philippine conservation-relevant areas (PCAs) to clarify the scope of our comparisons and to avoid confusion with the previous use of “priority conservation areas.” We limit acronym usage to PCA, while “protected and conserved areas” and “key biodiversity areas” are written out in full throughout the text for clarity and consistency.

Regarding concerns about interpretation under uneven sampling effort, we model observed species richness as a function of area, topographic relief, and occurrence density (as a proxy for sampling effort), with biogeographic subregion included as a random intercept to account for geographic clustering. This framework allows us to assess whether differences in observed richness among PCA types persist after accounting for variation in sampling intensity (and other predictors) and shared biogeographic structure. In addition, we modeled occurrence points as a function of area and topographic relief, again including biogeographic subregion as a random effect, to evaluate whether sampling effort itself varies systematically between PCA types (which we found no sufficient evidence). Finally, we clarify in the Discussion that our results reflect documented (observed) diversity patterns and should be interpreted in light of known data limitations.

Editor: Both reviewers note that several figures (particularly those illustrating spatial patterns) are difficult to interpret due to low resolution and small text. This may be related to how figures are displayed in the submission system; however, I recommend

Powered by Editorial Manager® and ProduXion Manager® from Aries Systems Corporation

you include all figures in the revised version at the highest resolution possible to facilitate careful evaluation by the reviewers.

Response: We will upload the highest-resolution version permitted by the submission system. The original figures were generated at high resolution; however, the conversion to PDF within the system may have reduced their clarity.

REVIEWER 1

Reviewer: I would suggest that the authors more explicitly connect each research question to the specific model(s) used. Clarifying which models and results correspond to which of the four main questions would enhance the transparency and coherence of the study design.

Response: We thank the Reviewer for raising this clarification. In the revised methods section, we now state each specific objective before describing the corresponding analytical approach to clarify the connection between the questions and the analyses. The Results section also follow the same structure.

Reviewer: Thank you for the detailed description of the data curation procedures. All the steps were important to guarantee the quality of the input data. However, I would like to raise a question regarding the spatial precision of the occurrence records used.

While the manuscript mentions the exclusion of records outside known distributions and the removal of generic coordinates, I did not find any reference to the use of the “coordinate uncertainty in meters” field (available in GBIF records) or a similar assessment of the dataset's spatial accuracy. Given that the study employs a 10-km grid resolution, I wonder whether this scale is appropriate considering the potential variability in coordinate precision, especially for historical museum records which can lack fine-scale georeferencing.

Could the authors clarify whether any filtering based on coordinate uncertainty was applied? If not, do the authors consider the 10-km resolution robust to such imprecision, and how might this potentially affect the spatial allocation of records and the resulting diversity patterns?

Response: We thank the reviewer for highlighting the issue of spatial precision. Because relatively few occurrence records include explicit uncertainty, we did not filter based on this metric. Instead, we retained records with geographic coordinates that correspond to general geographic location (provincial level). All records broadly reflect geographic patterns of each species distribution, and any minor imprecision is expected to have minimal impact on broad-scale, country-wide patterns. As stated above, although minor spatial imprecision may influence species richness estimates within individual PCAs used in the modeling, any resulting bias is expected to be minimal because most PCAs are supported by numerous occurrence records, species are represented by multiple points within PCAs due to the integration of multiple data sources, and the overall sample size is large (n = 316 PCAs).

The 10-km grid resolution was also deliberately chosen to visualize broad-scale distribution patterns across the Philippines. This scale provides an appropriate balance: smaller grids (e.g., <10 km) would be too fragmented on larger islands, while larger grids (e.g., 15–20 km) could average out meaningful spatial variation, particularly on smaller islands. We have included this justification in the revised manuscript (Lines 200–204).

Reviewer: The current analysis utilizes Negative Binomial GLMs fitted separately for Protected Areas (PAs) and Key Biodiversity Areas (KBAs). The methodological rationale for this decision is not fully clear in the manuscript. If this separation was a deliberate choice to address, for example, severe variance heterogeneity, it would be very helpful for the reader if this justification were made explicit.

My main concern with this approach is that a central objective of the study appears to be the comparison of predictor effects (e.g., area, topographic relief), and the comparison of richness and effort between PAs and KBAs. Adjusting separate models, while legitimate for describing the relationships within each group, impedes a formal

statistical comparison of the coefficient estimates.

To formally test the hypothesis that a predictor's effect differs between PAs and KBAs, the standard and more robust statistical approach would be to analyze both groups within a single, unified model. This is typically achieved by including the group affiliation (PA vs. KBA) as a categorical predictor and, crucially, testing interaction terms between this group variable and the predictors of interest (e.g., PCA_Type * Area). The p-value for the interaction term provides the formal statistical test of the null hypothesis that there is no difference in the slopes between the groups.

It is plausible that adopting this unified model approach would yield substantive results similar to those already presented. However, its key advantage would be to make the comparative claims—which are central to the study's objectives—statistically explicit and defensible, thereby increasing the analysis's robustness and interpretive clarity.

Response: We thank the Reviewer for this thoughtful and constructive feedback. In the revised manuscript, we reanalyzed the dataset using a joint modeling framework in which species richness was modeled as a function of area, topographic relief, and occurrence density, including all pairwise interactions among these predictors and their interactions with PCA type. Biogeographic subregion was included as a random intercept to account for non-independence among PCAs due to shared biogeographic structure. Although the results are broadly consistent with our previous approach, as the Reviewer noted, this framework allows for a more direct comparison between PCA types and highlights additional patterns for discussion. Hence, we kept this unified modelling framework. We have revised the Methods (lines 228–317), Results (lines 401–465), and Discussion (lines 522–578) sections accordingly.

Reviewer: My final methodological point relates to the model selection for control effects. The authors state that the random effect variable "PAIC" was removed from one of the models because it "explained less than 1% of the variance and did not improve model performance." (Line 234).

A low number of levels (seven, in this case) can lead to imprecise variance estimates. However, the decision to include or exclude a structural random effect should be based on the study design, not on its statistical performance (such as variance explained or AIC).

As the 'PAIC' variable represents a grouping variable (e.g., island/region) dictated by the sampling design, its function in the model is not primarily to "explain variance" (as in a predictive model) , but rather to control for the non-independence of the data (i.e., pseudoreplication). Observations from the same PAIC group are likely to be more similar to each other, violating the assumption of independence. The literature strongly advocates for retaining the random effects structure justified by the design. Removing this structural term, even if its variance is low, can lead to "overoptimistic" results, such as artificially reduced standard errors and an inflated Type I error rate. (Barr, D. J., Levy, R., Scheepers, C., & Tily, H. J. (2013). Random effects structure for confirmatory hypothesis testing: Keep it maximal. Journal of Memory and Language, 68(3), 255-278. https://doi.org/10.1016/j.jml.2012.11.001)

I would, therefore, recommend that the authors retain the 'PAIC' random effect in the final models to ensure that potential non-independence in the data is appropriately controlled for, thereby strengthening the validity of the model's inferences.

Response: We thank the Reviewer for this critical point. In our new models, we retained the biogeographic subregion (Pleistocene Aggregate Island Complexes or PAIC) as a random effect. Variance explained for the random effect are also reported in the results section, alongside the coefficient estimates of the fixed predictors.

Reviewer: Regarding the presentation of these results, I found many of the figures, both in the supplementary material and - more significantly - in the main manuscript, difficult to interpret: the resolution appears to be low, and the font size for labels and legends is very small. I would recommend the authors provide higher-resolution figures with more legible text to ensure readers can fully interpret the study’s important spatial results.

Powered by Editorial Manager® and ProduXion Manager® from Aries Systems Corporation

Response: We will upload the highest-resolution version permitted by the submission system. The original figures were generated at high resolution; however, the conversion to PDF within the system may have reduced their clarity.

REVIEWER 2

Our responses address the specific comments provided in the annotated manuscript, which correspond closely to the summarized remarks. Accordingly, we have responded directly to those in-text comments.

Reviewer: Lines 84–85: You are mixing terms and categories. Key Biodiversity Areas (KBAs) are referred to in the manuscript as Priority Conservation Areas (PCAs), yet the models and figures still use the term “KBAs.” This inconsistency creates confusion for the reader.

Furthermore, it is not clear which criteria were used to spatially define the Priority Conservation Areas. Based on the description, it appears that these areas correspond to the officially recognized KBAs in the Philippines. If that is the case, then the PCAs in your study are essentially KBAs, and this should be explicitly stated to avoid misinterpretation.

Response: We thank the Reviewer for raising this point. We recognize that the conceptual framing and terminology of conservation areas required greater precision or consistency in the original submission. Following the recommendation, we adopted the lexicon proposed by Salafsky et al. (2024), distinguishing between protected and conserved areas (i.e., areas with formal management or governance) and key biodiversity areas (i.e., spatial prioritization tools without inherent governance or protection status). In the revised manuscript, we refer to both collectively as Philippine conservation-relevant areas (PCAs) to clarify the scope of our comparisons and to avoid confusion with the previous use of “priority conservation areas.” We limit acronym usage to PCA, while “protected and conserved areas” and “key biodiversity areas” are written out in full throughout the text for clarity and consistency.

Reviewer: Lines 130–132: Why the criteria used to avoid duplication was time and not space? Did you drop data from that period indiscriminately without assessing the spatial distribution?

Response: The curated datasets used in Diesmos et al. (2015) and Leviton et al. (2018) were also sourced from GBIF. As a result, many records collected prior to 2015 and 2018 had already undergone rigorous curation, which allowed us to use our time efficiently during the manual data-cleaning process. The text was revised to read:

“To avoid overlap between GBIF records and these curated datasets (also sourced from GBIF), only GBIF records dated post-publication—2015 onwards for amphibians and 2018 onwards for snakes—were retained, since GBIF records for these groups collected before 2015 (for amphibains) and 2018 (for snakes) had already undergone rigorous curation. For lizards, all GBIF records were included since no curated datasets are available.”

Reviewer: From Line 139, It is not clear how you warrant the quality of the citizen science data. For example, many records of iNaturalist are from enthusiast observers and not from specialists, leading in some cases to overestimation of records. Careful review of the records will be desired to discard those dubious observations. Please, explain the steps to follow to ensure data quality.

Response: We thank the Reviewer for raising this point. Only research-grade observations were included in the dataset. Although we did not manually curate each individual record, our species-by-species data curation process was designed to flag potentially dubious occurrences. This process relies on genetically confirmed species boundaries reported in the literature, expert-curated species ranges (e.g., GARD), and our own expertise in Philippine herpetofauna systematics. In addition, curated specimens from natural history museums constitute the majority of the dataset (92%).

Reviewer: From Lines 144–145, Please specify which source did you follow to define the current taxonomy nomenclature.

Response: We revised the text to read: “Occurrence records for each species were then manually curated using currently accepted taxonomic treatments and synonyms, following the AmphibiaWeb (AmphibiaWeb, 2025) for amphibians and The Reptile Database (Uetz et al. 2025) for reptiles”

Reviewer: From Line 146: How did you define the geographic range of the species? red list expert maps, national assessment, other?

Response: We have revised the text to read: “Records falling outside the known geographic range of a species were excluded, following genetically confirmed species boundaries from published literature summarized in Meneses et al. 2024 (recent Philippine herpetology review), and excluding records lacking verifiable documentation (e.g., curated vouchered specimens or occurrences within the known distribution reported in peer-reviewed literature).”

In addition, we compared our preliminary range estimates for lizards with the Global Assessment of Reptile Distributions (GARD), which is an expert-curated database. We avoided using IUCN Red List maps, as they can be broad and may not always accurately reflect species boundaries.

Reviewer: From Line 183, Definition related to terms like CAs and PCAs is needed. you previously refer to OEMCs and KBA, but this are one of many figures of area based conservation. Review Nick Salafsky lexicon https://conbio.onlinelibrary.wiley.com/doi/10.1111/cobi.14269.

Response: We thank the reviewer for sharing the reference. We followed the definition proposed by Salafsky et al. (2024), distinguishing between protected and conserved areas (i.e., areas with formal management or governance) and key biodiversity areas (i.e., spatial prioritization tools without inherent governance or protection status). In the revised manuscript, we refer to both collectively as Philippine conservation-relevant areas (PCAs) to clarify the scope of our comparisons and to avoid confusion with the previous use of “priority conservation areas.” We limit acronym usage to PCA, while “protected and conserved areas” and “key biodiversity areas” are written out in full throughout the text for clarity and consistency.

Reviewer: From lines 217–218, I do not think this is the most appropriate approach. Species diversity is the outcome of multiple environmental and evolutionary factors, which you are not measuring here. Moreover, the manuscript itself argues that there are several sampling biases, which affect the observed patterns of species diversity.

I think that if you want to explore the relationship between species diversity and sampling effort, extrapolation and interpolation methods should be used. These approaches can indicate how well the current knowledge captures species diversity relative to the expected.

Response: We thank the Reviewer for this important clarification. We agree that species diversity arises from multiple environmental and evolutionary processes; for this reason, our focus is on the observed patterns rather than the underlying drivers. To avoid potential mischaracterization, we have used the term species diversity sparingly and consistently refer to counts of species as “observed species richness” throughout the manuscript. We also add a paragraph in the section “Predictors of observed species richness across PCAs”:

“Species richness is shaped by a complex interplay of environmental and evolutionary processes. However, the richness values analyzed here reflect observed species counts derived from available occurrence records. Accordingly, our analysis focuses on variation in observed species richness across PCAs and the influence of spatial attributes, rather than attempting to infer the underlying processes that generate biodiversity per se. Specifically, we examined whether area, topographic relief, and sampling effort help account for variation in observed richness, and whether these relationships differ among PCA types. As a complementary analysis, we also tested

	whether sampling intensity is predicted by these same spatial attributes of PCAs, and whether these relationships vary across PCA types. Calculations for each variable are outlined below, followed by a description of our modeling framework."<br><br>Reviewer: From Lines 217–220: Does the model account for the non-independence of the records? Spatial data and regression analysis must deal with spatial patterns and positive autocorrelation in the records. I suggest you review the GLM and evaluate the possibility of doing a Spatial GLM. An example: https://cran.r-project.org/web/packages/glmmfields/vignettes/spatial-glms.html<br><br>Response: We thank the Reviewer for this comment. In our modeling framework, we accounted for the non-independence of samples (306 Philippine conservation-relevant areas) by including biogeographic subregion (PAIC) as a random intercept, as PCAs may share similarity due to shared biogeographic structure. We included the variance explained by PAIC in our results section (which is negligible). We did not use individual occurrence points as model samples; instead, we used summary metrics (specifically, occurrence counts per km²) as a predictor to serve as a proxy for sampling effort.<br><br>Reviewer: From Line 282, Even though amphibians and reptiles share certain traits that allow them to be grouped together, their spatial patterns and global research efforts differ considerably. I suggest splitting the dataset to examine each group separately. If they show different tendencies, you should present the analyses independently; otherwise, they can be grouped, at least for this section.<br><br>Response: We acknowledge that amphibians and reptiles differ in their ecology and diversity patterns. However, as stated in the Introduction, these groups were used in this study because, relative to other taxonomic groups in the Philippines, they are comparatively well-documented and thus provide a robust basis for assessing observed biodiversity patterns and sampling distribution across areas. Our objective was not to model amphibian and reptile diversity separately or to compare their biological drivers, but rather to evaluate how observed species richness patterns (across well-sampled vertebrate taxa) relate to area, sampling effort, and topographic relief in the context of biodiversity documentation or conservation planning. Accordingly, our interpretation focuses on spatial patterns of documented diversity rather than taxon-specific ecological differences. In addition, amphibians and reptiles have historically and contemporarily been surveyed and analyzed jointly in the Philippines (see Meneses et al. 2024), making their combined treatment, at least within the context of this paper, consistent with established research practice.<br><br>Reviewer: From Lines 312 –313: According to this figure, Conservation Areas (CAs) include both Protected Areas and OECMs. However, these categories differ substantially in their capacity, governance, and origin. Protected Areas are generally designated based on ecosystem representativeness and specialist criteria, whereas OECMs—which are highly diverse in governance and conservation contributions—may include Indigenous territories, private reserves, and other land designations that contribute to conservation objectives such as ecosystem service provision and species protection. Therefore, grouping all CAs together may be misleading, as one cannot assume comparable levels of sampling effort or management capacity across these distinct types.Moreover, the manuscript does not justify why KBAs are analyzed separately from OECMs. An OECM may also qualify as a KBA, and vice versa, so the rationale for treating them as mutually exclusive categories needs clarification. It is necessary to clearly define and categorize the CAs, and to structure the analyses according to these categories.<br><br>Response: We thank the Reviewer for this comment. As noted in our earlier response, we adopted the framework proposed by Salafsky et al. (2024), distinguishing between protected and conserved areas (i.e., areas with formal management or governance) and key biodiversity areas (i.e., spatial prioritization tools without formal management or protection). In the revised manuscript, we refer to both collectively as Philippine conservation-relevant areas (PCAs) to clarify the scope of our comparisons and avoid confusion with the previous use of "priority conservation areas."<br>Within our dataset, protected and conserved areas include both PAs and OECMs, as both have some form of management or governance, though the mechanisms vary (even among PAs, at least in the Philippines). This distinction is meaningful relative to

KBAs, which generally lack formal management and are primarily spatial planning tools. Some KBAs have subsequently been established as protected or conserved areas, although boundaries do not always coincide, due to land-use and socio-political considerations, which we discuss in the manuscript to support interpretation of our results (see Lines 538–552). In essence, our split distinguishes managed versus unmanaged conservation-relevant areas, while retaining formal terminology commonly used in policy and planning discussions (particularly in the Philippines)
Reviewer: From Line 343, Consider changing this title, as it may lead to misunderstandings. You are not attempting to predict diversity, but rather sampling effort success in terms of species richness.
Response: We revised the phrasing to refer to predictors of observed species richness across conservation areas to maintain consistency with the terminology used throughout the manuscript.
Reviewer: From Line 458, The relationship between sampling completeness and diversity cannot be reliably assessed using the proposed models. I recommend considering interpolation and extrapolation diversity estimators, such as Jackknife or Chao, which are more appropriate for evaluating diversity completeness.
Response: We note that our focus is not on assessing sampling completeness per se, but on evaluating the relationship between sampling effort and observed species richness. Our modeling framework addresses this by including occurrence density as a predictor, serving as a proxy for sampling effort. Accordingly, the resulting observed species richness is interpreted in relation to variation in sampling effort, rather than as a measure of completeness.
Reviewer: From Lines 539–540: Why are they so valuable? Because there are so few records, or because the identified species are threatened or/and endemic?
Response: We revised the text for clarity: “Although many regions remain underrepresented in biodiversity data, it is noteworthy that areas with limited contemporary surveys—as mentioned earlier, Sulu Archipelago, where fieldwork has been limited [25]—still hold valuable records preserved through historical collections, most of which are the only verifiable species records for this biogeographic subregion.”
ADDITIONAL JOURNAL REQUIREMENTS
We moved this funding information to the funding statement:

“During the preparation of this manuscript, KMEP was partially supported by a Graduate Research Assistantship from the University of Kansas Department of Ecology and Evolutionary Biology; and RMB was supported by a Fulbright International Scholar Award administered by the U.S. Department of State, the Philippine-American Education Foundation (PAEF) and the Philippine Council for International Exchange of Scholars. Support for fieldwork that generated many specimen-associated records was provided by the U.S. National Science Foundation (DEB 073199, 0743491, 1418895, 0910341, 0804115, 1654388, and 0334952 and EF-0334952 to RMB and KU-based students), the KU College of Liberal Arts and Sciences Graduate Assistantship in Support of Faculty Research Award, a KU Biodiversity Institute Research and Exploration Fund grant, the South, Southeast, and East Asia Fund of the KU Office of International Affairs, and the General Research Fund of the University of Kansas. The funders had no role in study design, data collection and analysis, decision to publish, or preparation of the manuscript.”

Figures

Regarding the copyright of Figures 1, 2, and S5, we confirm that these maps were independently generated by the authors using open-access geospatial data. The foundational shapefiles for protected and conserved areas and key biodiversity areas were sourced from the National Integrated Protected Area System (NIPAS) dataset, while the national and administrative boundary shapefiles were provided by the National Mapping and Resource Information Auth ority (NAMRIA).

All layers were accessed via the Geoportal Philippines. According to the official Data Sharing Agreement managed by NAMRIA, the originating agencies (including DENR-BMB) authorize the public to 'freely download, reuse and share' this geospatial data (Clause f). Furthermore, the datasets' metadata indicates there are no access or use constraints. As the authors have used this unrestricted data to create original map visualizations, these figures are compatible with the CC BY 4.0 license and include

Powered by Editorial Manager® and ProduXion Manager® from Aries Systems Corporation

	proper attribution to the DENR-BMB and NAMRIA as the primary data sources.<br><br>(https://www.geoportal.gov.ph/resources/DataSharingAgreement.docx)
Additional Information:	
Question	Response
**Financial Disclosure**<br><br>Enter a financial disclosure statement that describes the sources of funding for the work included in this submission. Review the submission guidelines for detailed requirements. View published research articles from *PLOS ONE* for specific examples.<br><br>This statement is required for submission and **will appear in the published article** if the submission is accepted. Please make sure it is accurate.<br><br>**Funded studies**<br>Enter a statement with the following details:<br>• Initials of the authors who received each award<br>• Grant numbers awarded to each author<br>• The full name of each funder<br>• URL of each funder website<br>• Did the sponsors or funders play any role in the study design, data collection and analysis, decision to publish, or preparation of the manuscript?<br><br>Did you receive funding for this work?	The author(s) received no specific funding for this work.
**Competing Interests**<br><br>Use the instructions below to enter a competing interest statement for this submission. On behalf of all authors, disclose any competing interests that could be perceived to bias this work—acknowledging all financial support and any other relevant financial or non-financial competing interests.<br><br>This statement is required for submission and **will appear in the published article** if	The authors have declared that no competing interests exist.

the submission is accepted. Please make sure it is accurate and that any funding sources listed in your Funding Information later in the submission form are also declared in your Financial Disclosure statement.<br><br>View published research articles from *PLOS ONE* for specific examples.<br><br>**NO authors have competing interests**<br><br>Enter: *The authors have declared that no competing interests exist.*<br><br>**Authors with competing interests**<br><br>Enter competing interest details beginning with this statement:<br><br>*I have read the journal's policy and the authors of this manuscript have the following competing interests: [insert competing interests here]*<br><br>* typeset	
**Ethics Statement**<br><br>Enter an ethics statement for this submission. This statement is required if the study involved:<br><br>• Human participants<br>• Human specimens or tissue<br>• Vertebrate animals or cephalopods<br>• Vertebrate embryos or tissues<br>• Field research<br><br>Write "N/A" if the submission does not require an ethics statement.<br><br>General guidance is provided below. Consult the submission guidelines for detailed instructions. **Make sure that all information entered here is included in the Methods section of the manuscript.**	N/A

**Format for specific study types**<br><br>**Human Subject Research (involving human participants and/or tissue)**<br>• Give the name of the institutional review board or ethics committee that approved the study<br>• Include the approval number and/or a statement indicating approval of this research<br>• Indicate the form of consent obtained (written/oral) or the reason that consent was not obtained (e.g. the data were analyzed anonymously)<br><br>**Animal Research (involving vertebrate animals, embryos or tissues)**<br>• Provide the name of the Institutional Animal Care and Use Committee (IACUC) or other relevant ethics board that reviewed the study protocol, and indicate whether they approved this research or granted a formal waiver of ethical approval<br>• Include an approval number if one was obtained<br>• If the study involved *non-human primates*, add *additional details* about animal welfare and steps taken to ameliorate suffering<br>• If anesthesia, euthanasia, or any kind of animal sacrifice is part of the study, include briefly which substances and/or methods were applied<br><br>**Field Research**<br><br>Include the following details if this study involves the collection of plant, animal, or other materials from a natural setting:<br>• Field permit number<br>• Name of the institution or relevant body that granted permission	
**Data Availability**<br><br>Authors are required to make all data underlying the findings described fully available, without restriction, and from the time of publication. PLOS allows rare exceptions to address legal and ethical concerns. See the PLOS Data Policy and FAQ for detailed information.	Yes - all data are fully available without restriction

A Data Availability Statement describing where the data can be found is required at submission. Your answers to this question constitute the Data Availability Statement and **will be published in the article**, if accepted.<br><br>**Important:** Stating 'data available on request from the author' is not sufficient. If your data are only available upon request, select 'No' for the first question and explain your exceptional situation in the text box.<br><br>Do the authors confirm that all data underlying the findings described in their manuscript are fully available without restriction?	
**Describe where the data may be found in full sentences. If you are copying our sample text, replace any instances of XXX with the appropriate details.**<br><br>• If the data are **held or will be held in a public repository**, include URLs, accession numbers or DOIs. If this information will only be available after acceptance, indicate this by ticking the box below. For example: *All XXX files are available from the XXX database (accession number(s) XXX, XXX.*).<br>• If the data are all contained **within the manuscript and/or Supporting Information files**, enter the following: *All relevant data are within the manuscript and its Supporting Information files.*<br>• If neither of these applies but you are able to provide **details of access elsewhere**, with or without limitations, please do so. For example:<br><br>*Data cannot be shared publicly because of [XXX]. Data are available from the XXX Institutional Data Access / Ethics Committee (contact via XXX) for researchers who meet the criteria for access to confidential data.*<br><br>*The data underlying the results presented in the study are available from (include the name of the third party*	All data to replicate our analyses are publicly available at https://zenodo.org/records/17344465

and contact information or URL).<br>• This text is appropriate if the data are owned by a third party and authors do not have permission to share the data.<br><br>* typeset	
Additional data availability information:	

Powered by Editorial Manager® and ProduXion Manager® from Aries Systems Corporation

Revised Manuscript
Click here to access/download;Manuscript;180326 Biodiversity Data Manuscript.docx

Sampling bias obscures biodiversity patterns, reveals data gaps in a megadiverse island archipelago hotspot

Kier Mitchel E. Pitogo[1*], Camila G. Meneses[1], Syrus Cesar P. Decena[1,2], Christian E. Supsup[1], Hannah E. Som[1], Justin M. Bernstein[3], Kin Onn Chan[1,#a], Mark William Herr[1], Rafe M. Brown[1]

[1]*Department of Ecology and Evolutionary Biology & Biodiversity Institute, University of Kansas, Lawrence, KS, USA*

[2]*College of Agricultural and Environmental Sciences, Visayas State University-Alangalang, Leyte, Philippines*

[3]*Department of Mammalogy, Division of Vertebrate Zoology, American Museum of Natural History, New York, NY, USA*

[#a]*Current Address: Department of Integrative Biology & MSU Museum, Michigan State University, East Lansing, MI, USA*

*Corresponding author: Kier Mitchel E. Pitogo

E-mail: pitogo.kiermitchel@gmail.com

Abstract

Where and how species are sampled can shape biodiversity knowledge, spatial patterns, and data-driven conservation. In many Global South megadiverse, biodiversity hotspots, sampling remains uneven, and available data often lack the synthesis needed to assess region-wide gaps for effective conservation planning and priority-setting. This shortfall is common within areas considered important for biodiversity. We demonstrate this case in the Philippines, one of the world's most biodiverse countries, where longstanding research and growing policy momentum support expansion of these conservation-relevant areas (hereafter "Philippine conservation-relevant areas" or PCAs). Using over a century of species occurrence records made digitally accessible, which we compiled and manually curated from multiple sources, we analyzed country-wide spatial distribution of observed amphibian and squamate reptile species richness. We also tested whether species richness across PCAs is explained by area, topographic relief, and sampling effort, and whether effects vary between PCA type: protected and conserved areas (with management or governance schemes) and key biodiversity areas (unmanaged, but priority areas for conservation). Results reveal strong spatial biases, with preserved specimens comprising 92% of all records and largely shaping species richness patterns. Citizen-science data reinforce already well-sampled regions, whereas peer-reviewed literature provides valuable documentation in undersampled areas. PCAs are proportionally well-sampled, with sampling effort and species richness increasing with area. However, this positive area effect weakens with increasing topographic relief, which may suggest that large mountain ranges remain documentation blind spots. The species–area relationship and the influence of sampling effort are stronger in protected and conserved areas, whereas topographic relief exerts a stronger effect in

key biodiversity areas. Notably, several high-richness areas occur outside established protected areas. Our findings highlight sampling gaps and biases and underscore the value of integrating multiple data sources. We outline enabling mechanisms to strengthen primary biodiversity data collection, guiding future surveys toward undersampled regions.

Introduction

Biodiversity occurrence data remain unevenly distributed across taxonomic groups and geographic regions, with pronounced biases against global biodiversity hotspots [1–5]. Several factors contribute to these biases, including limited research capacity [6,7], preferential focus on certain taxonomic groups [3,4,8] and charismatic taxa [9,10], ease of site accessibility [11–13], and prevailing tendency to conduct research in areas of special interest [14,15]. As a result, many regions and their wildlife remain poorly sampled—and where data exist, they are often not digitally accessible or fall into "biodiversity blind spots", obscuring biodiversity patterns [5]. These sampling biases can hinder robust macroecological analyses and obstruct evidence-based conservation planning [17–20], particularly at national scales where most conservation policies and management actions are implemented [21–23]. Therefore, identifying where biodiversity data exist, for which taxa, and to what extent is essential for proactively addressing these biases and strengthening the knowledge base needed for effective conservation.

Challenges in biodiversity data availability are persistent even within global biodiversity hotspots and megadiverse countries, like the Philippines, where gaps and biases in data coverage skew biodiversity knowledge toward certain regions and taxonomic groups [24–26]. As a megadiverse and biogeographically unique country threatened by habitat loss [27,28], the

Philippines urgently needs robust and data-informed conservation strategies. However, limited availability and inequitable discoverability of biodiversity data, and a lack of information on how these data are spatially distributed, constrains efforts to evaluate sampling completeness and to guide national biodiversity strategies and research priorities [23,29]. The need for synthesized biodiversity data to inform conservation planning is especially urgent, given ongoing biodiversity loss and the global momentum under the Kunming–Montreal Global Biodiversity Framework [30,31]. These factors have prompted policy responses to expand protected areas and recognize other effective area-based conservation measures, which now cover approximately 15% of the Philippines' land area [32]. This expansion not only demands effective management grounded in ecologically representative, well-connected, and equitably governed networks of protected and conserved areas [30] but also must deliver measurable, positive outcomes for biodiversity [33]. A critical first step towards achieving such outcomes is to strengthen biodiversity data by improving sampling coverage and accessibility [21,23,29,34,35–37].

A key step toward ensuring that biodiversity knowledge is sufficient to support conservation strategies is to assess where biodiversity data are spatially distributed and how these patterns align with areas considered important for biodiversity (hereafter 'Philippine conservation-relevant areas' or PCAs), including protected and conserved areas (with governance and management schemes) and key biodiversity areas (priority areas for conservation, with no management or governance schemes), following standard terminologies [38]. Such an approach remains lacking for the Philippines despite long-standing efforts to document wildlife in this country [24–26,39–48]. Although data deficiencies affect many taxonomic groups, some are better represented due to sustained research and collection efforts.

Amphibians and squamate reptiles (herpetofauna) offer a compelling case for such analysis in the Philippines. The group has benefited from over a century of active and sustained research effort [25,39]; in fact, recent years have seen an acceleration of engagement in amphibian and reptile studies, with increasing numbers of researchers, broader types of investigations, and expanding publication output [25], all suggesting that herpetofauna are a particularly timely and relevant group for such work and that data availability will likely continue to grow. Endemic herpetofauna are also relatively well-sampled genetically, with voucher specimens housed in museum collections [26]; and curated occurrence records are available in substantial volume across peer-reviewed literature [49,50]. Moreover, amphibians and reptiles have been shown to serve as effective surrogates for broader vertebrate biodiversity patterns in KBAs across the country [51]. Herpetofauna thus provide a valuable lens to evaluate spatial patterns in biodiversity data for improving country-wide biodiversity documentation and ensuring that conservation efforts are grounded in the best available evidence.

Leveraging species occurrence data on Philippine herpetofauna spanning ~125 years (1900s–2025) that are made digitally accessible, we compiled, manually curated, and analyzed occurrence records to conduct country-wide spatial assessments of observed species richness of amphibians and squamate reptiles using data from multiple sources. Specifically, we ask: (1) How is observed herpetofaunal species richness spatially distributed across the Philippines? (2) How do these patterns differ across datasets derived from museum collections, citizen-science platforms, and peer-reviewed literature? (3) How does area, topographic relief, and sampling intensity shape variations in observed species richness across PCAs? And (4) do these variations in observed species richnness differ between protected and conserved areas and key biodiversity

areas? Our findings offer novel, long-overdue insights into spatial distribution of biodiversity data in the Philippines, particularly in relation to PCAs. Drawing from our results, we propose and discuss enabling mechanisms to improve primary biodiversity data collection, strengthening the knowledge base necessary in assessing management effectiveness for area-based conservation measures needed in the country [52,53]. By identifying key data shortfalls and sampling biases, this study contributes to the growing body of empirical evidence underscoring the critical role of integrating digitally accessible biodiversity data from multiple sources in revealing sampling gaps and biases, guiding future studies towards poorly sampled areas and informing conservation priorities.

Materials & methods

Data assembly and curation

All georeferenced occurrence records for amphibians and squamate reptiles from the Philippines were downloaded from the Global Biodiversity Information Facility (GBIF) on 31 January 2025 (amphibians) and 07 February 2025 (squamate reptiles). To reduce data duplication, we initially excluded records categorized as material citations and retained only human-observation records from iNaturalist (research grade) and HerpWatch Pilipinas, a non-government organization from the Philippines that documents herpetofaunal diversity. These data were then merged with expert-curated species occurrence data from the literature: amphibians from the Amphibians of the Philippines, part I: checklist of the species [49] and snakes from the Synopsis of snakes of the Philippines [50]. To avoid overlap between GBIF

records and these curated datasets (also sourced from GBIF), only GBIF records dated post-publication—2015 onwards for amphibians and 2018 onwards for snakes—were retained, since GBIF records for these groups collected before 2015 (for amphibains) and 2018 (for snakes) had already undergone rigorous curation. For lizards, all GBIF records were included since no curated datasets are available. Duplicate specimen records across institutions were identified using shared field numbers. When duplicates were detected, only records from the University of Kansas Natural History Museum (KU) were retained. Finally, we incorporated verifiable occurrence records of non-museum-catalogued specimens from peer-reviewed literature compiled by a recent review on Philippine herpetology over the last 20 years [25], along with a few additional entries. For consistency in categorization, all museum records were treated as "preserved specimen" records; iNaturalist and HerpWatch Pilipinas as "citizen science" records; and data from peer-reviewed literature as "material citation" records.

After initial clean-up, we recovered 471 nominal and candidate species (i.e., populations supported by evidence from peer-reviewed studies but not yet formally described) of terrestrial amphibians and squamate reptiles that have digitally accessible occurrence records. Occurrence records for each species were then manually curated using currently accepted taxonomic treatments and synonyms, following the AmphibiaWeb [54] for amphibians and The Reptile Database [55] for reptiles . Each species' occurrence records were mapped in QGIS 3.4 Madeira [56] to assess spatial accuracy of each point. Records falling outside the known geographic range of a species were excluded, following genetically confirmed species boundaries from published literature summarized in the recent Philippine herpetology review paper [25], and those lacking verifiable documentation (e.g., curated vouchered specimens, occurrences within known

distribution from peer-reviewed literature). In cases where a formerly widespread species had been taxonomically split, historical records were reassigned to currently accepted species name based on updated diagnostic and distributional information in taxonomic studies. For species with unclear taxonomic or geographic boundaries, only records supported by verifiable documentation were retained. Since expert-curated distributional literature for Philippine lizards is lacking, we adopted an additional measure by comparing our preliminary range estimates with the Global Assessment of Reptile Distributions [57–58]. This conservative step was taken to minimize potential overestimation of species richness.

Records not identified to the species level were excluded unless a species name or a placeholder name (e.g., sp. + island name, sp. + placeholder number) could be confidently assigned without the risk of double-counting (e.g., when only a single species from a genus is known to occur in the area). For example, the frog genus *Platymantis* remains taxonomically unresolved with many potentially undescribed species [59–61]. We accounted for this uncertainty by adopting a conservative approach by including records of candidate species occurring in areas with no known range overlap with closely related congeners. This strategy minimizes the risk of double-counting should these populations ultimately represent a single species, while also retaining valuable data that would otherwise be discarded. This decision was informed by phylogenetic evidence, following published phylogeny of Ceratobatrachidae [59].

All records lacking locality information were discarded. Additionally, records with coordinates at 16.45°N, 120.55°E and 13°N, 122°E from GBIF were excluded, as the associated locality descriptions did not match the projected geographic locations. These coordinates appear

to be generalized placeholders rather than accurate site data. In the final dataset, we retained records whose geographic coordinates were consistent with their general (provincial-level) locality. Although minor spatial imprecision is unlikely to affect broad, country-scale patterns, it may influence species richness estimates within individual PCAs used in the modeling. However, any resulting bias is expected to be minimal because most PCAs are supported by numerous occurrence records, species are represented by multiple points within PCAs due to the integration of multiple data sources, and the overall sample size is large (n = 316 PCAs).

Statistical analyses

Mapping herpetofaunal diversity across the Philippines

To assess the spatial distribution of observed herpetofaunal species richness patterns, we aggregated occurrence records within a spatial grid and mapped resulting species richness values. Occurrence records for amphibians and squamate reptiles were used to generate a presence–absence matrix (PAM) at a 10-km grid resolution across the Philippines, using prepare_base_pam function from the R package biosurvey [62]. Based on the overall geographic template of the Philippines, we selected a 10-km grid resolution for this visual assessment because it better captures coarse-scale patterns across islands of varying sizes. Smaller grids (e.g., 5 km) would be too fragmented in larger islands, while larger grids (e.g., 20 km) risked averaging out important spatial variation, especially in smaller islands.

Separate PAMs were generated for (1) combined dataset, (2) preserved-specimen records, (3) citizen-science records, and (4) material-citation records to determine if observed species richness patterns differ among datasets. For each dataset, the unique number of species was calculated per 10-km grid cell, and the results were visualized as spatial maps using QGIS 3.4 Madeira [56]. The resulting maps of herpetofaunal species richness were overlaid with shapefiles of PCAs to assess spatial overlap between species richness and PCA coverage area. Geospatial data for protected and conserved areas, as well as key biodiversity areas [63], were sourced from the National Integrated Protected Area System dataset provided by the Department of Environment and Natural Resources – Biodiversity Management Bureau (DENR-BMB). National boundary shapefile was obtained from the National Mapping and Resource Information Authority (NAMRIA). All data were accessed via the Geoportal Philippines, the central repository for official Philippine geospatial information (https://www.geoportal.gov.ph/).

To assess whether species richness classes were evenly represented across grid cells, a chi-square goodness-of-fit test was conducted using the chisq.test function in R version 4.3.1 [64]. Richness values were grouped into nine discrete classes based on the observed range, with 90 as the maximum per grid cell (i.e., 0, 1–10, 11–20, …, 81–90). The observed frequency of cells in each class was compared to a uniform distribution, under the assumption that all classes were equally likely. Expected counts were computed by dividing the total number of grid cells by the number of richness classes. All expected frequencies exceeded 5, meeting assumptions of the chi-square test.

Predictors of observed species richness across PCAs

Species richness is shaped by a complex interplay of environmental and evolutionary processes. However, the richness values analyzed here reflect observed species counts derived from available occurrence records. Accordingly, our analysis focuses on variation in observed species richness across PCAs and the influence of spatial attributes, rather than attempting to infer the underlying processes that generate biodiversity per se. Specifically, we examined whether area, topographic relief, and sampling effort help account for variation in observed richness, and whether these relationships differ among PCA types. As a complementary analysis, we also tested whether sampling effort is predicted by these same spatial attributes of PCAs, and whether these relationships vary across PCA types. Calculations for each variable are outlined below, followed by a description of our modeling framework.

To quantify species occurrence records and observed species richness within PCAs, we spatially intersected occurrence points with PCA boundaries using “Join Attributes by Location” tool in QGIS 3.4 Madeira [56] (input: occurrence points; join layer: PCA polygons; predicate: intersects). The resulting dataset was exported as a CSV file and processed in R statistical software [64], where occurrence records without PCA matches were excluded. Data were grouped by PCA to calculate the number of occurrence records and unique species per PCA. PCAs without intersecting occurrences were retained with zero counts. Polygon area (in km^2) was calculated using expanse function in the R package terra [65]. Only terrestrial PCAs were included in the analysis, comprising 207 protected and conserved areas and 109 key biodiversity areas.

Topographic relief for each PCA was extracted using a Digital Elevation Model (DEM) of the Philippines downloaded from the CGIAR Consortium for Spatial Information (https://srtm.csi.cgiar.org/). This DEM, provided in Arc/Info Grid format, was clipped to the spatial extents of each PCA. Clipping was performed using QGIS' 'Clip Raster by Mask Layer' tool, with DEM as the input raster and PCA polygon shapefiles as the mask layer. The resulting clipped DEM layers contained elevation data exclusively within each PCA boundary. Subsequently, minimum and maximum elevation values were calculated for each PCA polygon using the 'Zonal Statistics' tool in QGIS. The difference between these values was used to quantify topographic relief (in meters) of each PCA.

To test whether area, topographic relief, and sampling intensity explain variations in observed species richness across PCAs, we fitted generalized linear mixed-effects models (GLMMs) with a negative binomial error distribution to account for overdispersion (dispersion parameter = 0.7552; AIC = 1354.7), which improved model fit relative to a Poisson model (dispersion parameter = 2.1871; AIC = 1602.8). The response variable was the total number of species recorded per PCA. Fixed effects included total area (km^2), occurrence density (number of records per km^2), topographic relief (m), and all pairwise interactions among these predictors. Area and topographic relief were used as proxies for landscape features, based on the hypothesis that larger PCAs and those spanning broader elevational gradients — thus encompassing more heterogeneous habitats — support higher species richness [66,67]. Occurrence density served as a proxy for sampling effort. Predictor variables were transformed to improve model fit: area and occurrence density were $\log_{10}$-transformed due to strong right skew, and topographic relief was

square-root-transformed due to moderate skew. All predictors were then mean-centered and scaled to facilitate convergence and allow direct comparisons of effect sizes.

To determine whether observed species richness patterns differ between protected and conserved areas (managed) and key biodiversity areas (unmanaged), PCA type was included as a fixed effect interacting with all main predictors and their pairwise interactions. This unified modeling framework allows direct comparison of effect sizes between designation types, and the interaction terms formally test whether both individual predictors and their interactions vary between PCA types. To account for potential non-independence among PCAs within biogeographic regions (i.e., PCAs share more similarity in species composition by virtue of historical biogeographic structuring), we included the Pleistocene Aggregate Island Complex (PAIC; see [27]) as a random intercept in all models. Residual diagnostics indicated minor deviations for occurrence density (S1 Fig), likely reflecting zero inflation from undersampled sites rather than model misspecification. Tests of overdispersion and residual uniformity indicated no violations of model assumptions (S1 Fig).

To further facilitate interpretation, we explicitly modeled the number of occurrence points to assess the extent to which variation in sampling effort may influence observed species richness patterns, with occurrence counts incorporated as a proxy measure of sampling intensity. Total occurrence records per PCA were modeled as a function of PCA type and its interaction with area, topographic relief, and the area × topographic relief interaction, with PAIC again included as a random effect. A negative binomial error distribution was used to account for overdispersion (dispersion parameter = 1.574; AIC = 2561.9), which substantially improved

model fit relative to a Poisson model (dispersion = 660.4; AIC = 102443). Residual diagnostics indicated no major violations of model assumptions, although some quantile deviations were observed for each predictor, likely reflecting increased variance at large PCAs where fewer observations exist (S2 Fig). This sampling-effort model allow us to test whether PCAs with lower observed species richness are undersampled.

Generalized linear models were fitted using the R package glmmTMB [68]. Overdispersion was initially assessed using a custom R function that calculates the ratio of the sum of squared Pearson residuals to residual degrees of freedom, with values >1.5 considered indicative of overdispersion requiring correction [69]. Model diagnostics for final models were conducted using the DHARMa package [70]. Simulated residuals were generated with the simulateResiduals function, and goodness-of-fit tests on scaled residuals were done using testZeroInflation, testUniformity, and testDispersion functions. Residuals were also plotted against each fixed predictor using plotResiduals to check for non-random patterns. Variance inflation factors (VIFs) were calculated using the R package performance [71]. In the full interaction model, some terms exhibited elevated VIF values (> 5), which are expected due to the inclusion of higher-order interaction terms and do not indicate problematic collinearity among primary predictors. To validate this assumption, we modeled the main effects only, with species richness as the response variable; all predictors showed low multicollinearity (VIFs < 5). All sampling effort models, with occurrence points as the response variable, showed low VIF values (< 5.2). All diagnostic outputs and residual plots are provided in S1–S2 Figs.

As a complement to model-based inference, bivariate relationships were visualized using negative binomial generalized linear models (log link) with 95% confidence intervals, fitted separately by PCA type using the geom_smooth function in the R package ggplot2 [72]. Predictor variables were transformed to match model specifications, and point colors were used to reflect the values of interacting variables with significant effects, unless otherwise stated, aiding interpretation of interaction patterns. All statistical analyses were conducted in R version 4.3.1 [64] and documented in Supplementary Material.

Results

Spatial distribution of herpetofaunal diversity in the Philippines

Spatial distribution of observed herpetofaunal species richness in the Philippines is non-uniform and fragmented, with unequal national-scale coverage that varies considerably across islands (Fig 1A). The distribution of species richness classes significantly deviated from a uniform expectation ($\chi^2(8) = 15{,}843$, $p < 0.001$), indicating uneven observed species richness across the archipelago. Some richness classes were overrepresented, while others were underrepresented relative to expectations if all classes were equally likely. Nationally, only ~2% (n = 87) of the 10-km² grids have more than 41 observed species—roughly 50% of maximum richness recorded in any grid—while 31% (n = 1,370) have between 1 and 40 species, and 66.7% (n = 2,914) have zero recorded species.

Fig 1. Spatial distribution of observed amphibian and squamate reptile species richness for (A) combined datasets; (B) preserved-specimen records; (C) citizen-science records; and (D) material-citation records. Species richness is calculated per 10-km^2 grid cell across the Philippines. Inset bar graph shows frequency distribution of grid cells across species richness classes for each dataset. Dashed red line indicates expected frequency for each class under a uniform distribution, as used in chi-square goodness-of-fit tests. Administrative boundary and biodiversity area shapefiles were sourced from NAMRIA and the DENR-BMB via Geoportal Philippines (https://www.geoportal.gov.ph/).

Different data sources provide complementary insights into observed herpetofaunal species richness patterns. The majority of occurrence records came from preserved specimens (n = 70,112), followed by citizen science contributions (n = 3,735) and material citations (n = 2,344), while 37 points were unclassified. Both preserved-specimen and citizen-science data represent individual occurrences, while material citations typically reflect species-level records, as published species inventory studies mostly report presence by species rather than by individuals. All three types of data sources significantly deviated from a uniform expectation ($\chi^2(8)$ = 20,186–30,610, $p < 0.001$). Because preserved specimens account for 92% of all records, spatial richness patterns largely mirror those derived from the combined dataset (Fig 1B). Citizen science data generally contribute 1–10 species per grid, with a few grids reaching up to 30 species, and tend to complement regions already covered by specimen-based surveys (Fig 1C). Material citation data are concentrated in fewer areas, consistent with the targeted, site-specific nature of biotic inventories (Fig 1D).

Philippine conservation-relevant areas (PCAs) were proportionally well-sampled, although distribution of herpetofaunal species richness patterns is uneven and concentrated in few areas (Fig 2). Of the 76,228 curated occurrence records, 21,210 (27.8%) fell within protected and conserved areas and 23,058 (30.2%) within key biodiversity areas—proportions that are disproportionately high relative to the Philippine land area occupied by these designations (12–15% for protected and conserved areas and 6.72% for key biodiversity areas). Overlaying species richness with PCA boundaries shows that whereas some high-richness grid cells lie within their boundaries, many large sites include cells with sparse or no records. Notably, 52.7% of 207 protected and conserved areas and 20.2% of 109 key biodiversity areas contain grid cells with zero species records, and an additional 22.4% of protected and conserved areas and 29.4% of key biodiversity areas contain only up to 10 recorded species. At the same time, several well-sampled areas with high observed species richness (>40 recorded species) occur outside established protected and conserved areas but within forested areas, many of which partially overlap with key biodiversity areas (S3 Fig).

Fig 2. Relative locations of well-sampled (≥40 species) conservation-relevant areas across the Philippines (PCAs). Total area, occurrence point density, and number of species are included for each area. Base map shows spatial distribution of observed amphibian and squamate reptile richness patterns for combined data sources. Inset histogram shows frequency distribution of PCAs by species richness and number of occurrence records. Administrative boundary and biodiversity area shapefiles were sourced from NAMRIA and the DENR-BMB via Geoportal Philippines (https://www.geoportal.gov.ph/).

Well-surveyed sites, those with >40 recorded species, make up 8.8% of protected and conserved areas and 18.3% of key biodiversity areas (Fig 2). Particularly species-rich locations (>60 species) include Samar Island Natural Park, Pasonanca Natural Park, Mt. Malindang Natural Park, Mt. Makiling Forest Reserve, and PP1636 (unnamed wildlife sanctuary in southcentral Luzon). These sites have been extensively surveyed, with data available in public repositories like GBIF, though many records remain absent from, or not reported in, peer-reviewed literature (but see [74,75]). Other well-documented areas combine strong specimen representation in collections with published data: Cuernos de Negros [76,77], Aurora Memorial National Park [78,79], Mt. Busa–Kimba [61,79], Mt. Guiting-Guiting [80,81], Mt. Hilong-hilong [60,82], Pantabangan-Caranglan Watershed Reservation [83], and Victoria-Anepahan Ranges [84]. The 40-species cut-off, however, is used here solely as a reference point for discussion and should not be interpreted as a threshold of "high species diversity," since observed values may be influenced by sampling limitations and environmental factors.

Predictors of observed species richness across PCAs

There was insufficient evidence of a difference in baseline species richness between key biodiversity areas (reference category) and protected and conserved areas (Table 1). In key biodiversity areas, observed species richness increased with total area, topographic relief, and occurrence density. Larger key biodiversity areas supported more species ($\beta\_area = 1.2724 \pm 0.1407$ SE, $z = 9.043$, $p < 0.001$; Fig 3A), richness increased strongly with occurrence density ($\beta\_occurrence\ density = 1.5975 \pm 0.1084$, $z = 14.74$, $p < 0.001$; Fig 3B), and areas encompassing greater topographic relief were richer ($\beta\_relief = 0.4970 \pm 0.1550$, $z = 3.206$, $p = 0.001$; Fig 3C).

Two-way interactions among predictors indicated non-additive effects of spatial attributes. Specifically, the positive effect of area on richness weakened in key biodiversity areas with greater topographic relief (area × topographic relief: β = –0.3587 ± 0.1107, z = –3.241, p = 0.001), suggesting that the species–area relationship is less steep in more topographically heterogeneous landscapes. Likewise, the positive effect of occurrence density on richness decreased slightly as topographic relief increased (occurrence density × topographic relief: β = –0.3477 ± 0.1304, z = –2.665, p = 0.008).

Relative to key biodiversity areas, protected and conserved areas exhibited significantly steeper species–area relationships (PCA type × area: β = 0.3834 ± 0.1322, z = 2.170, p = 0.03; Fig 3A) and stronger positive effects of occurrence density (PCA type × occurrence density: β = 0.3779 ± 0.1007, z = 2.992, p = 0.003; Fig 3b), whereas the effect of topographic relief on species richness was weaker in protected and conserved areas (PCA type × topographic relief: β = –0.4699 ± 0.1880, z = –2.500, p = 0.012; Fig 3C). None of the higher-order interactions involving PCA type were significant, indicating that the overall interaction structure among species richness drivers was broadly similar between PCA types. The random intercept for biogeographic subregion (PAIC) explained negligible variance in richness (variance = 1.04×10^{-9}; SD = 3.23×10^{-5}), suggesting minimal influence of biogeographic clustering on observed richness.

Table 1. Coefficient estimates from a generalized linear mixed-effects model (negative binomial) in which species richness was modeled as a function of area, topographic relief, and occurrence density, including all pairwise interactions among these predictors and

their interactions with Philippine conservation-relevant area (PCA) type (reference category: key biodiversity area). Biogeographic subregion was included as a random intercept.

**Predictors**	**Estimate ± SE**	**z-value**	**p-value**
Conserved Areas			
Intercept	1.0397 ± 0.14382	7.229	<0.01*
PCA Type	–0.1913 ± 0.1677	–1.140	0.2542
Area	1.2724 ± 0.1407	9.043	<0.01*
Occurrence Density	1.5975 ± 0.1084	14.74	<0.01*
Topographic Relief	0.4970 ± 0.1550	3.206	0.0013*
Area × Occurrence Density	0.1916 ± 0.1237	1.550	0.1213
Area × Topographic Relief	–0.3587 ± 0.1107	–3.241	0.0012*
Occurrence Density × Topographic Relief	–0.3477 ± 0.1304	–2.665	0.0077*
PCA Type × Area	0.3834 ± 0.1322	2.170	0.0300*
PCA Type × Occurrence Density	0.3779 ± 0.1007	2.992	0.0028*
PCA Type × Topographic Relief	–0.4699 ± 0.1083	–2.500	0.0124*
PCA Type × Area × Occurrence Density	–0.0849 ± 0.1616	–0.526	0.5992
PCA Type × Area × Topographic Relief	0.1943 ± 0.1363	1.425	0.1542
PCA Type × Occurrence Density × Topographic Relief	0.24436 ± 0.1704	1.434	0.1516

Note: Total area and occurrence density were log-transformed, and topographic relief was square-root-transformed; all predictors were scaled and mean-centered. Significance assessed at $\alpha = 0.05$(*).

Fig 3. Bivariate relationships between species richness and total area, occurrence density, and topographic relief; and between sampling effort and area and topographic relief across Philippine conservation-relevant areas (PCAs). Lines represent negative binomial generalized linear model fits (log link) estimated separately by PCA type, with grey bands indicating 95% confidence intervals. Points represent individual PCAs; colors denote interacting predictors. Only occurrence density × topographic relief and area × topographic relief interactions were statistically significant.

To evaluate potential sampling bias, total occurrence records per PCA were modeled as a function of area, topographic relief, and PCA type. There was insufficient evidence of differences in baseline sampling intensity between key biodiversity areas and protected and conserved areas (Table 2). In key biodiversity areas, sampling intensity increased marginally with area ($\beta = 0.7267 \pm 0.3782$, $z = 1.922$, $p = 0.055$; Fig 3D) and topographic relief ($\beta = 0.7181 \pm 0.3854$, $z = 1.863$, $p = 0.062$; Fig 3E), and none of the interactions with PCA type were significant. The PAIC random intercept accounted for moderate variance in sampling (variance = 0.46; SD = 0.68), indicating geographic clustering in sampling effort. Together, these findings suggest that differences between PCA types arise from how area and topographic relief shape species richness, rather than from inherent differences in baseline richness levels. Importantly, these patterns are unlikely to reflect systematic differences in sampling effort.

Table 2. Coefficient estimates from a generalized linear mixed-effects model (negative binomial) in which number of occurrence points was modeled as a function of area,

topographic relief, area × topographic relief, and their interactions with Philippine conservation-relevant area (PCA) type (reference category: key biodiversity area). Biogeographic subregion was included as a random intercept.

Predictors	Estimate ± SE	z-value	p-value
Intercept	5.0046 ± 0.4661	10.737	< 0.01 *
PCA Type	-0.5019 ± 0.4034	-1.244	0.2134
Area	0.7267 ± 0.3782	1.922	0.0547
Topographic Relief	0.7181 ± 0.3854	1.863	0.0624
Area × Topographic Relief	-0.5365 ± 0.3187	-1.683	0.0923
PCA Type × Area	0.1607 ± 0.4746	0.339	0.7350
PCA Type × Topographic Relief	0.3017 ± 0.5100	0.592	0.5541
PCA Type × Area × Topographic Relief	0.2050 ± 0.3862	0.531	0.5956

Note: Total area was log-transformed and topographic relief was square-root-transformed; continuous predictors were scaled and mean-centered. Significance assessed at $\alpha = 0.05$(*).

Discussion

Uneven spatial diversity pattern reflects sampling biases

Our spatial mapping of observed species richness of amphibians and squamate reptiles revealed stark patterns of sampling biases. More than two-thirds of grid cells contain either zero or only 1–10 documented species, while comparatively fewer cells (~2%) exhibit higher species richness levels. This pronounced disparity likely reflects persistent spatial biases in biodiversity knowledge. Observed species richness patterns likely indicate where sampling has been more intensive, rather than accurately representing underlying ecological or biogeographic factors—a pattern that is persistent across many biodiversity-rich areas globally [17,20,21,85]. Addressing these biases is critical not only for improving knowledge of Philippine biodiversity but also for ensuring that conservation policy, priority-setting, and management decisions rest on a more complete and representative knowledge base.

A recent comprehensive review of Philippine herpetology revealed substantial geographic gaps in field sampling across the archipelago (see [25] for a detailed discussion). Our findings broadly align with these observations but offer additional fine-scale information by integrating curated species point data and grid-based species richness estimates. This approach not only identifies where sampling has occurred but also quantifies its intensity, suggesting that higher species richness values are associated with well-sampled sites (i.e., more occurrence points). Notably, we found that a disproportionate share of documented herpetofaunal diversity is concentrated in a few intensively surveyed areas—primarily in Luzon (the largest island) and in the West Visayas, which comprise much of the central islands—where collection effort has historically been high [25,39]. However, many records from the West Visayas date to pre-2000 surveys and warrant resampling [25]. High-resolution data of this kind are critical for accurately

representing spatial patterns of sampling effort, thereby guiding resources and research efforts towards poorly explored regions. Furthermore, by incorporating species point data from different sources, we recovered diversity data in areas that would otherwise appear as knowledge gaps, such as the Sulu Archipelago in the south [25]; although sampling effort is still disproportionately lower in this region. As such, given that biodiversity data in the Philippines are fragmented, reliance on selective or incomplete datasets for *any* synthesis studies can introduce substantial bias [86].

Philippine conservation-relevant areas (PCAs) are relatively well-sampled in proportion to their total area, yet herpetofaunal knowledge across them remains limited and uneven. Despite these gains, many PCAs still lack species records altogether or may have data that are not accessible in digital form. Where species data are lacking in PCAs, they are however available outside delineated boundaries, which may suggest persistent issues associated with sampling within PCAs, including permitting and other bureaucratic processes (particularly for legislated protected areas), logistical access, and security constraints [25,60,87] (see Fig 4). Although well-surveyed sites include some of the country's known PCAs, vast portions of other mountain ranges and many smaller islands remain poorly sampled or lack species data that are readily accessible for broader scientific and conservation use. Expanding targeted field-based surveys beyond established PCAs—and ensuring that resulting data are made publicly and digitally available for mobilization—remains urgently needed to support conservation initiatives grounded in accurate, comprehensive biodiversity knowledge [21,88].

Fig 4. Constraints in field-based biodiversity data collection and corresponding enabling mechanisms to improve primary biodiversity data coverage. Each mechanism primarily addresses one or more constraint types.

Shared drivers scale differently across conservation-relevant areas

Our unified modeling framework provides insufficient statistical evidence that baseline species richness differs between types of conservation-relevant areas in the Philippines (protected and conserved areas vs. key biodiversity areas) after accounting for area, sampling effort, and topographic relief. However, the strength of these predictors varies significantly between designation types. In both PCA types, area, occurrence density, and topographic relief positively influence observed richness. Yet, the species–area relationship and the effect of sampling effort are significantly stronger in protected and conserved areas, whereas the effect of topographic relief is stronger in key biodiversity areas. Importantly, these patterns cannot be attributed to systematic differences in sampling intensity between designation types. Our sampling effort model showed no baseline difference in number of occurrence points between PCA types, nor any interaction between designation type and area or topographic relief. Thus, the stronger richness–area and richness–sampling relationships observed in protected and conserved areas do not appear to arise from uneven survey effort alone.

The stronger elevational effect in key biodiversity areas suggests that mountainous key biodiversity areas may disproportionately capture high-elevation diversity relative to protected and conserved areas. One possible explanation is that key biodiversity areas are planning tools

that target areas of high ecological importance, such as concentrations of endemic or range-restricted taxa, many of which occur in topographically complex regions [89]. As such, their boundary delineations may cover wider topographic relief to cover habitat diversity gradient along elevation. In contrast, protected and conserved area designation involves additional socio-political and land-use considerations that may not always prioritize elevational heterogeneity [90–92]. In the Philippines, nationally legislated protected areas are designated based not only on biodiversity value but also on socio-cultural importance and provision of key ecosystem services, including regional water security, which may be politically influenced [93,94]. In addition, our definition of protected and conserved areas includes other effective area-based conservation measures, such as Indigenous and Community Conserved Areas and locally managed conservation areas, whose establishment is often motivated by social and governance considerations beyond ecological criteria [92].

Between both designation types, the positive effect of topographic relief weakens as area and sampling intensity increase, as indicated by significant negative interaction terms. This shared pattern contrasts with the common expectation that large, topographically complex areas should support disproportionately high diversity due to habitat heterogeneity [17,66,67,95]. One plausible explanation is that elevational heterogeneity may strongly promote species turnover at smaller spatial extents, but as area increases, much of this gradient may already be represented, leading to partial redundancy in species composition (see scale effects on elevational species richness [96]). Similarly, in heavily sampled PCAs, additional survey effort may disproportionately document widespread or low-elevation taxa rather than reveal the full

complement of high-elevation specialists, many of which are rarely documented and taxonomically undescribed [42,60,62,75,81,97].

Sampling constraints likely further contribute to this pattern. Mountainous regions in the Philippines are frequently remote, difficult to access, and logistically expensive to survey [97] (see Fig 4). Even where PCAs encompass wide topographic relief, field effort may be concentrated in accessible foothill zones, leaving high-elevation habitats underrepresented [25]. This undersampling in high-elevation areas, particularly for herpetofauna, has been pointed out in a recent review of Philippine herpetology [25]. As a result, observed richness may underestimate the contribution of topographically complex systems, systematically biasing biodiversity knowledge away from montane environments despite their well-documented ecological importance and high levels of local endemism [38, 98]. Together, these findings suggest that large, mountainous PCAs do not automatically translate into proportionally higher recorded richness — not because they lack diversity, but because the ecological benefits of elevational heterogeneity may be masked by scale-dependent redundancy and uneven sampling coverage.

Biodiversity data limitations are not confined to large mountain ranges. Smaller conservation areas, particularly those in island environments, also suffer from substantial knowledge gaps [51]. Although this pattern was not statistically prominent in our model-based inference, it was evident in our data visualizations (e.g., small-sized, low-topographic-relief PCAs in Fig 3). Many small island conservation areas in the Philippines remain poorly sampled despite yielding newly discovered species and harboring unique, range-restricted taxa [99–107].

These systems face disproportionate threats from climate-driven sea-level rise, extreme weather events, invasive species, and habitat loss [108], yet lack the baseline biodiversity data for adaptive management. Targeted surveys are urgently needed in these areas to document species presence and build taxonomic and ecological knowledge base required for effective conservation in islands [109]. Without action, island ecosystems risk becoming critical blind spots in the country's conservation efforts.

Data gaps and biases persist, but diverse sources can address them

The majority of digitally accessible knowledge of Philippine herpetofauna comes from natural history collections. These collections represent over a century of fieldwork that has shaped taxonomic and systematics knowledge, increasing herpetofaunal diversity estimates in the country [25,39]. As such, specimen-associated data serve as a vital resource for improving knowledge of biodiversity patterns [5,110]. They also support large-scale ecological studies, which depend on spatially referenced records to examine broad biodiversity trends and guide conservation planning [16,31,111]. Although many regions remain underrepresented in biodiversity data, it is noteworthy that areas with limited contemporary surveys—as mentioned earlier, Sulu Archipelago, where fieldwork has been limited [25]—still hold valuable records preserved through historical collections, many of which are the only verifiable species records for this biogeographic subregion.

In areas where specimens are lacking, citizen science provides a valuable complementary data stream to help address distributional biases. Online biodiversity platforms [112–114] and

social media [115–118] have become particularly useful where museum records are absent or limited. Although still emerging in Philippine herpetology (but see [118–120]), citizen-science contributions are already well established in other taxonomic groups. For example, active birding communities regularly contribute to platforms like eBird [121] and collaborate with researchers and biodiversity managers to inform site-level conservation efforts in the Philippines [94]. Another notable initiative is Co's Digital Flora of the Philippines [122], where citizen scientists and taxonomists work together to maintain a real-time overview of Philippine flora. At least 54% of the country's vascular plant species have been photo-documented, many with associated geographic coordinates [41]. These examples show that citizen-science contributions to observed diversity may be more pronounced in taxonomic groups with active, organized communities driving such efforts. Despite challenges related to data quality and metadata completeness, carefully curated citizen-science records can enhance biodiversity knowledge, particularly in remote or poorly sampled regions [112,123].

Another important yet often overlooked source of biodiversity data comes from formal surveys that do not involve specimen collection and eventual deposition in natural history museums [61,83,124–126]. And if there are such records, they are often not digitized and published in publicly accessible domains, especially when deposited in university-based museums. Many in-country scientists conduct fieldwork that reports valuable records published in peer-reviewed journals, yet these data are rarely archived in open-access databases such as GBIF [127]. Such studies frequently document species in poorly sampled areas in the Philippines, providing crucial complementary information [25]. Although valuable for expanding species distribution knowledge, many of these studies lack specific geographic coordinates of

areas sampled, limiting their use for geospatial research; thus, submission of spatial occurrences to online databases like GBIF is highly encouraged [36]. In addition, a wealth of biodiversity data remains locked in grey literature—government reports, university theses, and project documents—that are not digitally accessible but are often used in site-level management. Incorporating these sources into public repositories and ensuring they are properly curated would improve national biodiversity coverage [128].

Disparate biodiversity data from different sources underscore the need for standardized archiving practices [129–131] and greater adherence to best-practice guidelines in dealing with big data [20,132]. Although these varied data streams help fill distributional gaps, they often differ in quality, accessibility, and curation. These differences are especially true for non-specimen-based data, which typically lack the standardized metadata (Darwin Core format) associated with museum specimens. To maximize their scientific utility, we recommend that non-specimen-based records be accompanied by metadata, including GPS coordinates (including uncertainty), observation date and time, natural history notes, among others. Robust metadata not only enhance credibility and utility of individual records but also facilitates their integration into broader ecological, biogeographic, and conservation research [16].

Scaling biodiversity documentation to meet conservation targets

The Kunming-Montreal Global Biodiversity Framework, adopted in 2022, sets an ambitious goal: to protect 30% of the world's terrestrial and marine ecosystems by 2030, building on the earlier Aichi Target 11 [133]. Achieving this target requires more than simply

expanding protected area coverage; it also demands effective management that delivers measurable benefits for biodiversity [30]. These outcomes depend on robust, accurate biodiversity data, particularly within conservation-relevant areas in the Philippines [34,134,135]. However, our results show that persistent data gaps and biases, especially in large mountainous and island conservation areas, continue to shape observed biodiversity data patterns. Although many PCAs with apparent data gaps may in fact hold biodiversity data, their lack of digital accessibility limits inclusion in national-scale assessments of sampling effort. This limitation hinders resource allocation for improved documentation, reduces opportunities for external vetting and expert curation of data, and impedes integration into broader datasets that inform national conservation planning [21,31,130,136].

Addressing the challenge of insufficient biodiversity documentation requires developing and implementing enabling mechanisms to overcome constraints in site-level primary data collection, which comprises the majority—if not all—of available biodiversity data (Fig 4). This includes prioritizing biodiversity documentation within national conservation policies and planning frameworks (e.g., Philippine Biodiversity Strategy and Action Plan, PBSAP 2024–2040) to strengthen institutional support for field-based activities, especially within PCAs [23]. For example, streamlining research permitting processes, fully aligned with Indigenous Peoples' rights and national access and benefit-sharing policies, can reduce bureaucratic barriers to ethical and standardized data collection [36,137]. Engaging Indigenous Peoples and local communities as collaborators, and supporting culturally appropriate awareness and communication initiatives, can further foster equitable partnerships and mutual knowledge exchange [138]. Financial mechanisms within national legislated Protected Areas, such as the Integrated Protected Area

Fund, can be leveraged to support student-led and locally collaborative field research. Local or subnational government policies and support mechanisms are also critical for ensuring logistical coordination, safe access, and community involvement in fieldwork.

Beyond site-level interventions, targeted national funding for species discovery and taxonomic capacity-building can incentivize early-career researchers to pursue field-based work [137,139]. These efforts should be complemented by investments in biodiversity science within university curricula [140], ensuring students are trained in both theory and practice. Addressing the limited data management capacity among local researchers is also critical for promoting effective data-sharing, enhancing the verifiability and utility of biodiversity studies. Comprehensive training programs and standardized data systems can help overcome this barrier, ensuring the production of high-quality, accessible datasets. In parallel, the increasing diversity of data contributors underscores the need for a streamlined and curated national biodiversity data infrastructure [141]. Such a platform should ensure that biodiversity data, especially those generated from PCAs, are digitally and publicly accessible, enabling diverse stakeholders to collectively contribute to and benefit from biodiversity knowledge. Ultimately, these in-country initiatives must be paired with stronger institutional mechanisms, such as equitable collaboration standards and data-sharing agreements, to ensure that non-monetary benefits from biological resource use are meaningfully shared with local collaborators and inform national policy and conservation planning [142, 143].

Finally, scaling biodiversity documentation beyond existing PCAs can guide the identification of new priority sites for protected area establishment. Our findings show that

several well-sampled, high-diversity areas remain outside current PCAs (neither protected/conserved nor delineated as priority areas for conservation), many overlapping with only partially protected key biodiversity areas. This mismatch underscores opportunities to align future protected and conserved area expansion (i.e., protected area, other effective area-based conservation measures) with empirically documented biodiversity patterns, particularly in remaining forested regions. The updated national biodiversity strategy and action plan (PBSAP 2024–2040) targets protecting 24% of terrestrial areas through protected and conserved areas [144], yet only ~15% are currently designated. Prioritizing well-sampled, high-diversity areas for protection will be critical to achieving this goal. Our results, however, only focus on herpetofauna and similar data gaps and biases likely affect many taxonomic groups in the Philippines [24,26] reinforcing the need for broader, inclusive biodiversity documentation. Building a robust and representative biodiversity knowledge base—well-curated and aligned with FAIR (findable, accessible, interoperable, reusable) and CARE (collective benefit, authority to control, responsibility, ethics) principles—will require sustained collaboration among scientists, conservation practitioners, institutions, and critically, with Indigenous Peoples and local communities [16, 143–146]. Strengthening such collective and inclusive efforts is essential for transforming biodiversity knowledge into tangible conservation outcomes and ensuring the Philippines meets its national and global biodiversity commitments.

Acknowledgements

We thank Marites Bonachita Sanguila, Michael Clores, Michael Cuesta, Aljohn Jay Saavedra, and Tristan Luap Senarillos for numerous discussions and their valuable insights on the challenges of primary biodiversity data collection in the Philippines. We would also

like to thank the Editor and two reviewers for their constructive feedback, which greatly improved the manuscript.

References

1. Hortal J, De Bello F, Diniz-Filho JAF, Lewinsohn TM, Lobo JM, & Ladle RJ. Seven shortfalls that beset large-scale knowledge of biodiversity. Annual Review of Ecology, Evolution, and Systematics. 2015; 46(1): 523–549.
2. Hughes AC, Orr MC, Ma K, Costello MJ, Waller J, Provoost P, et al. Sampling biases shape our view of the natural world. Ecography. 2021; 44(9): 1259–1269.
3. Moura MR, Jetz W. Shortfalls and opportunities in terrestrial vertebrate species discovery. Nature Ecology & Evolution. 2021; 5(5): 631–639.
4. Ondo I, Dhanjal-Adams KL, Pironon S, Silvestro D, Colli-Silva M, Deklerck V, et al. Plant diversity darkspots for global collection priorities. New Phytologist. 2024; 244(2): 719–733.
5. Ball L, Rodríguez-Machado S, Paredes-Burneo D, Rutledge S, Boyd DA, Vander Pluym D, et al. What 'unexplored' means: mapping regions with digitized natural history records to look for 'biodiversity blindspots'. PeerJ. 2025; 13: e18511.
6. Barber PH, Ablan-Lagman MCA, Berlinck RG, Cahyani D, Crandall ED, Ravago-Gotanco R, et al. Advancing biodiversity research in developing countries: the need for changing paradigms. Bulletin of Marine Science. 2014; 90(1): 187–210.
7. Zhang L, Yang L, Chapman CA, Peres CA, Lee TM, Fan PF. Growing disparity in global conservation research capacity and its impact on biodiversity conservation. One Earth. 2023; 6(2): 147–157.

8. Titley MA, Snaddon JL, Turner EC. Scientific research on animal biodiversity is systematically biased towards vertebrates and temperate regions. PloS one. 2017; 12(12): e0189577.
9. Troudet J, Grandcolas P, Blin A, Vignes-Lebbe R, Legendre F. Taxonomic bias in biodiversity data and societal preferences. Scientific Reports. 2017; 7(1): 9132.
10. Guénard B, Hughes AC, Lainé C, Cannicci S, Russell BD, Williams GA. Limited and biased global conservation funding means most threatened species remain unsupported. Proceedings of the National Academy of Sciences. 2025; 122(9): e2412479122.
11. Oliveira U, Paglia AP, Brescovit AD, de Carvalho CJ, Silva DP, Rezende DT, et al. The strong influence of collection bias on biodiversity knowledge shortfalls of Brazilian terrestrial biodiversity. Diversity and Distributions. 2016; 22(12): 1232–1244.
12. Mandeville CP, Nilsen EB, Finstad AG. Spatial distribution of biodiversity citizen science in a natural area depends on area accessibility and differs from other recreational area use. Ecological Solutions and Evidence. 2022; 3(4): e12185.
13. Penhacek M, Castro-Souza RA, Tessarolo G, Diniz-Filho JA, Sobral-Souza T, de Jesus Rodrigues D. Biases in amphibian sampling in the Amazon: Using infrastructure and accessibility data to identify sampling gaps. Biotropica. 2025; 57(5): e70079.
14. Sastre P, Lobo JM. Taxonomist survey biases and the unveiling of biodiversity patterns. Biological Conservation. 2009; 142(2): 462–467.
15. Mentges A, Blowes SA, Hodapp D, Hillebrand H, Chase JM. Effects of site-selection bias on estimates of biodiversity change. Conservation Biology. 2021; 35(2): 688–698.
16. Jetz W, McPherson JM, Guralnick RP. Integrating biodiversity distribution knowledge: toward a global map of life. Trends in Ecology & Evolution. 2012; 27(3): 151–159.

17. Engemann K, Enquist BJ, Sandel B, Boyle B, Jørgensen PM, Morueta-Holme N, et al. Limited sampling hampers “big data” estimation of species richness in a tropical biodiversity hotspot. Ecology and Evolution. 2015; 5(3): 807–820.

18. Santini L, Antão LH, Jung M, Benítez-López A, Rapacciuolo G, Di Marco M, et al. The interface between macroecology and conservation: existing links and untapped opportunities. Frontiers of Biogeography. 2021; 13(4): e53025.

19. Diniz-Filho JAF, Jardim L, Guedes JJ, Meyer L, Stropp J, Frateles LEF, et al. Macroecological links between the Linnean, Wallacean, and Darwinian shortfalls. Frontiers of Biogeography, 2023; 15(2): e59566.

20. Hughes AC, Dorey JB, Bossert S, Qiao H, Orr MC. Big data, big problems? How to circumvent problems in biodiversity mapping and ensure meaningful results. Ecography. 2024; 2024(8): e07115.

21. Grattarola F, Martínez-Lanfranco JA, Botto G, Naya DE, Maneyro R, Mai P, et al. Multiple forms of hotspots of tetrapod biodiversity and the challenges of open-access data scarcity. Scientific Reports. 2020; 10(1): 22045.

22. Perino A, Pereira HM, Felipe-Lucia M, Kim H, Kühl HS, Marselle MR, et al. Biodiversity post-2020: Closing the gap between global targets and national-level implementation. Conservation Letters. 2021; 15(2): e12848.

23. Soberón J. Biodiversity informatics for public policy. The case of CONABIO in Mexico. Biodiversity Informatics. 2022; 17.

24. Berba CMP, Matias AMA. State of biodiversity documentation in the Philippines: Metadata gaps, taxonomic biases, and spatial biases in the DNA barcode data of animal and plant taxa in the context of species occurrence data. PeerJ. 2022; 10: e13146.

25. Meneses CG, Pitogo KME, Supsup CE, Brown RM. Philippine herpetology (Amphibia, Reptilia), 20 years on: two decades of progress towards an increasingly collaborative, equitable, and inclusive approach to the study of the archipelago's amphibians and reptiles. ZooKeys. 2024; 1190, 213–257.

26. Pitogo KME. Gaps and biases in vertebrate wildlife genetics from a global biodiversity hotspot. Environmental Conservation. 2025; 52(3): 127–138.

27. Brown RM, Siler CD, Oliveros CH, Esselstyn JA, Diesmos AC, Hosner PA, et al. Evolutionary processes of diversification in a model island archipelago. Annual Review of Ecology, Evolution, and Systematics. 2013; 44(1): 411–435.

28. Huais PY, Osorio-Olvera L, Cordier JM, Tomba AN, Soberón J, Loyola R, Nori J. Rethinking global hotspots for threatened terrestrial vertebrates. Global Ecology and Biogeography. 2025; 34(1): e13942.

29. Soberón J, Peterson AT. Monitoring biodiversity loss with primary species-occurrence data: toward national-level indicators for the 2010 target of the convention on biological diversity. AMBIO: A Journal of the Human Environment. 2009; 38(1): 29–34.

30. CBD [Convention on Biological Diversity] [cited 01 August 2025]. Decision adopted by the Conference of the Parties to the Convention on Biological Diversity CBD/COP/DEC/15/4 Kunming-Montreal Global Biodiversity Framework. 2022; CBD/COP/DEC/15/4. Available from: https://www.cbd.int/doc/decisions/cop-15/cop-15-dec-04-en.pdf

31. Orr MC, Hughes AC, Costello MJ, Qiao H. Biodiversity data synthesis is critical for realizing a functional post-2020 framework. Biological Conservation. 2022; 274: 109735.

32. ASEAN Centre for Biodiversity. ASEAN Biodiversity Outlook 3. Philippines, pp. 392; 2023.
33. Maxwell SL, Cazalis V, Dudley N, Hoffmann M, Rodrigues AS, Stolton S, et al. Area-based conservation in the twenty-first century. Nature. 2020; 586(7828): 217–227.
34. Mallari NAD, Collar NJ, McGowan PJ, Marsden SJ. Science-driven management of protected areas: a Philippine case study. Environmental Management. 2013; 51(6): 1236–1246.
35. Jetz W, McGeoch MA, Guralnick R, Ferrier S, Beck J, Costello MJ, et al. Essential biodiversity variables for mapping and monitoring species populations. Nature Ecology & Evolution. 2019; 3(4): 539–551.
36. Hochkirch A, Samways MJ, Gerlach J, Böhm M, Williams P, Cardoso P, et al. A strategy for the next decade to address data deficiency in neglected biodiversity. Conservation Biology. 2021; 35(2): 502–509.
37. Urbano F, Viterbi R, Pedrotti L, Vettorazzo E, Movalli C, Corlatti L. Enhancing biodiversity conservation and monitoring in protected areas through efficient data management. Environmental Monitoring and Assessment. 2024; 196(1): 12.
38. Salafsky N, Mejía Cortez P, de Meyer K, Dudley N, Klimmek H, Lewis A, et al. A standard lexicon of terms for area-based conservation version 1.0. Conservation Biology. 2024; 38(4): e14269.
39. Brown RM, Diesmos AC, Alcala AC. The state of Philippine herpetology and the challenges for the next decade. Silliman Journal. 2001; 42(1): 18–87.
40. Heaney LR. A decade of research on Philippine mammals: progress and challenges. Silliman Journal. 2001; 42(1): 88–107.

41. Pelser PB, Barcelona JF, Nickrent DL . Co's Digital Flora of the Philippines. Available from: www. philippineplants.org.
42. Heaney LR, Balete DS, Rickart EA. The mammals of Luzon Island: biogeography and natural history of a Philippine fauna. JHU Press; 2016.
43. Tanalgo KC, Hughes AC. Bats of the Philippine Islands—A review of research directions and relevance to national-level priorities and targets. Mammalian Biology. 2018; 91(1): 46–56.
44. Allen D. Birds of the Philippines. Lynx and Birdlife International Field Guides, Lynx Edicions, Barcelona, Spain; 2020.
45. Gamalo LE, Sabanal B, Ang A. Three decades of Philippine nonhuman primate studies: research gaps and opportunities for Philippine primatology. Primates. 2021; 62(1): 233–239.
46. delos Angeles MD, Radbouchoom S, Ngarega BK, Lapuz RS, Schneider H. Spatial diversity and distribution of fern and lycophyte species in karst and non-karst landscapes towards conservation needs. Frontiers in Plant Science. 2025; 16: 1495796.
47. Fernandez DAP, Do Linh San E, Forget PM, Veron G. Carnivorans of the Philippines: current knowledge and research gaps. Journal of Mammalogy. 2025; gyaf020.
48. Balisco RAT, Liao TY. An annotated checklist of fish species described from the Philippines. ZooKeys. 2025; 1246: 1–287.
49. Diesmos AC, Watters JL, Huron NA, Davis DR, Alcala AC, Crombie RI, et al. Amphibians of the Philippines, part I: checklist of the species. Proceedings of the California Academy of Sciences. 2015; 62(20): 457–539.

50. Leviton AE, Siler CD, Weinell JL, Brown RM. Synopsis of the snakes of the Philippines. Proceedings of the California Academy of Sciences. 2018; 64(14): 399–568.

51. Fidelino JS, Constantino RRP, Duya MRM. Cross-taxon congruence of terrestrial vertebrates across Philippine Key Biodiversity Areas. Journal for Nature Conservation. 2025; 86: 126907.

52. Mallari NAD, Collar NJ, McGowan PJ, Marsden SJ. Philippine protected areas are not meeting the biodiversity coverage and management effectiveness requirements of Aichi Target 11. Ambio. 2016; 45(3): 313–322.

53. Struebig MJ, Lee JS, Deere NJ, Gevaña DT, Ingram DJ, Lwin N. Drivers and solutions to Southeast Asia's biodiversity crisis. Nature Reviews Biodiversity. 2025;1: 497–514.

54. AmphibiaWeb. Berkeley, California: University of California; 2025 [cited 2025 Jun 25]. Available from: https://amphibiaweb.org

55. Uetz P, Freed P, Aguilar R, Reyes F, Kudera J, Hošek J, editors. The Reptile Database. 2025 [cited 2025 Jun 14]. Available: http://www.reptile-database.org

56. QGIS Development Team. QGIS Geographic Information System. Open Source Geospatial Foundation Project; 2018. Available from: http://qgis.osgeo.org.

57. Roll U, Feldman A, Novosolov M, Allison A, Bauer AM, Bernard R, et al. The global distribution of tetrapods reveals a need for targeted reptile conservation. Nature Ecology & Evolution. 2017; 1(11): 1677–1682.

58. Caetano GHDO, Chapple DG, Grenyer R, Raz T, Rosenblatt J, Tingley R, et al. Automated assessment reveals that the extinction risk of reptiles is widely underestimated across space and phylogeny. PLoS Biology. 2022; 20(5): e3001544.

59. Brown RM, Siler CD, Richards SJ, Diesmos AC, Cannatella DC. Multilocus phylogeny and a new classification for Southeast Asian and Melanesian forest frogs (family Ceratobatrachidae). Zoological Journal of the Linnean Society. 2015; 174(1): 130–168.
60. Sanguila MB, Cobb KA, Siler CD, Diesmos AC, Alcala AC, Brown RM. The amphibians and reptiles of Mindanao Island, southern Philippines, II: the herpetofauna of northeast Mindanao and adjacent islands. ZooKeys. 2016; (624), 1–132.
61. Pitogo KME, Saavedra AJL, Afuang LE. Amphibians and reptiles of Mount Busa, Sarangani Province: a glimpse of the herpetological community of southern Mindanao, Philippines. Philippine Journal of Science. 2021; 150(5): 1279–1306.
62. Nuñez-Penichet C, Cobos ME, Soberón J, Gueta T, Barve N, Barve V, Navarro-Sigüenza AG, Peterson AT. Selection of sampling sites for biodiversity inventory: Effects of environmental and geographical considerations. Methods in Ecology and Evolution. 2022; 00:1–13.
63. Ong PS, Afuang LE, Rosell-Ambal RG. Philippine Biodiversity Conservation Priorities: A second iteration of the National Biodiversity Strategy and Action Plan. Department of Environment and Natural Resources-Protected Areas and Wildlife Bureau, Conservation International Philippines, Biodiversity Conservation Program-University of the Philippines Center for Integrative and Development Studies, and Foundation for the Philippine Environment. Quezon City, Philippines; 2002.
64. R Core Team (2022). R: A language and environment for statistical computing. R Foundation for Statistical Computing, Vienna, Austria. Available from: https://www.R-project.org/.

65. Hijmans R._terra: Spatial Data Analysis_. R package version 1.7-78; 2025. Available from: https://CRAN.R-project.org/package=terra.

66. Ricklefs RE, Lovette IJ. The roles of island area per se and habitat diversity in the species–area relationships of four Lesser Antillean faunal groups. Journal of Animal Ecology. 1999; 68(6): 1142–1160.

67. Gotelli NJ. A Primer of Ecology, 4th ed. Sunderland, MA: Sinauer Associates; 2008.

68. Brooks ME, Kristensen K, Van Benthem KJ, Magnusson A, Berg CW, Nielsen A, et al. glmmTMB balances speed and flexibility among packages for zero-inflated generalized linear mixed modeling. The R Journal. 2017; 9(2): 378–400.

69. Zuur AF, Ieno EN, Walker N, Saveliev AA, Smith GM, Zuur AF, et al. GLM and GAM for count data. Mixed effects models and extensions in ecology with R, 209–243; 2009.

70. Hartig F. _DHARMa: Residual Diagnostics for Hierarchical (Multi-Level / Mixed) Regression Models_. R package version 0.4.7; 2024. Available from: https://CRAN.R-project.org/package=DHARMa.

71. Lüdecke D, Ben-Shachar MS, Patil I, Waggoner P, Makowski D. performance: An R Package for Assessment, Comparison and Testing of Statistical Models. Journal of Open Source Software. 2021; 6(60): 3139.

72. H. Wickham. ggplot2: Elegant Graphics for Data Analysis. Springer-Verlag New York; 2016.

73. Nuñeza OM, Ates FB, Alicante AA. Distribution of endemic and threatened herpetofauna in Mt. Malindang, Mindanao, Philippines. Biodiversity and Conservation. 2010; 19, 503–518.

74. Gonzalez JCT, de Guia APO, Dimalibot JC, Pantua KV, Gustilo WO, Bantayan NC. Understorey to canopy vertebrate fauna of a lowland evergreen forest in Mt. Makiling Forest Reserve, Philippines. Biodiversity Data Journal. 72; 8: e56999.

75. Brown WC, Alcala AC. Populations of amphibians and reptiles in the submontane and montane forests of Cuernos de Negros, Philippine Islands. Ecology. 1961; 628–636.

76. Brown WC, Alcala AC. The zoogeography of the Philippine Islands, a fringing archipelago. Proceedings of the California Academy of Sciences. 1970; 38: 105–130.

77. Brown RM, McGuire JA, Ferner JW, Icarangal Jr N, Kennedy RS. Amphibians and reptiles of Luzon Island, II: preliminary report on the herpetofauna of Aurora Memorial National Park, Philippines. Hamadryad. 2000; 25:175–195

78. Siler CD, Welton LJ, Siler JM, Brown J, Bucol A, Diesmos AC, Brown RM. Amphibians and Reptiles, Luzon Island, Aurora Province and Aurora Memorial National Park, Northern Philippines: New island distribution records. Check List. 2011; 7(2): 182–195.

79. Brown RM. A new species of stream frog of the genus *Hylarana* from the mountains of southern Mindanao Island, Philippines. Herpetologica. 2015; 71(3): 223–233.

80. Siler CD, Welton LJ, Diesmos AC, Siler JM, Bucol A, Brown J, Brown RM. Amphibians and reptiles, Romblon Island Group, central Philippines: comprehensive herpetofaunal inventory. Check List. 2012; 8(3): 443–462.

81. Meneses CG, Siler CD, Alviola PA, Gonzalez JCT, Balatibat JB, Natividad CA, Brown RM. Amphibian and reptile diversity along a ridge-to-reef elevational gradient on a small isolated oceanic island of the central Philippines. Check List. 2012; 18(5): 941–948.

82. Plaza JL, Sanguila MB. Preliminary report on the anurans of Mount Hilong-hilong, Agusan del Norte, eastern Mindanao, Philippines. Asian Herpetological Research. 2015; 6(1): 18–33.

83. Gojo-Cruz PHP, Afuang LE, Gonzalez JCT, Gruezo WS. Amphibians and reptiles of Luzon Island, Philippines: the herpetofauna of Pantabangan-Carranglan watershed, Nueva Ecija Province, Caraballo Mountain range. Asian Herpetological Research. 2018; 9(4): 201–223.

84. Supsup CE, Asis AA, Carestia Jr UV, Diesmos AC, Mallari NAD, Brown RM. Variation in species richness, composition and herpetological community structure across a tropical habitat gradient of Palawan Island, Philippines. Herpetozoa. 2020; 33: 95–111.

85. Azovsky AI. Species–area and species–sampling effort relationships: disentangling the effects. Ecography. 2011; 34(1): 18–30.

86. Pitogo KME, Meneses CG, Flores ABA, Saavedra AJL, Amarga AKS, Angeles MD, et al. Caught in statistical noise: pitfalls of a unidimensional approach to understanding biodiversity-conflict relationships. npj Biodiversity. 2025; 4(1): 17.

87. Brown RM, Meneses CG, Wood Jr PL, Fernandez JB, Cuesta MA, Clores MA, et al. Unexpected discovery of another new species of Philippine false gecko (Gekkonidae; *Pseudogekko*) from the Bicol Peninsula of Luzon Island. Herpetologica. 2020; 76(3): 315–329.

88. Di Minin E, Toivonen T. Global protected area expansion: creating more than paper parks. BioScience. 2015; 65(7): 637–638.

89. Eken G, Bennun L, Brooks TM, Darwall W, Fishpool LD, Foster M, et al. Key biodiversity areas as site conservation targets. BioScience. 2004; 54(12): 1110–1118.

90. Watson JE, Dudley N, Segan DB, Hockings M. The performance and potential of protected areas. Nature. 2014; 515(7525): 67–73.

91. Jonas HD, Ahmadia GN, Bingham HC, Briggs J, Butchart SHM, Carino J, Chassot O, Chaudhary S, et al. Equitable and effective areas-based conservation: Towards the conserved area paradigm. PARKS. 2021; 21: 71–84.

92. Zhang Y, West P, Thakholi L, Suryawanshi K, Supuma M, Straub D, Sithole SS, Sharma R, et al. Governance and conservation effectiveness in protected areas and indigenous and locally managed areas. Annual Review of Environment and Resources. 2023; 48(1): 559–588.

93. DENR [Department of Environment and Natural Resources]. Clarifying the procedural guidelines in the conduct of Protected Area Suitability Assessment (PASA). DENR Administrative Order No. 2016-04, 11 pp; 2017.

94. Pitogo KME, Jarvis F, Saavedra AJL, Senarillos TL, Concepcion CB, Simpson PM. Avian fauna of Mount Melibengoy, southern Mindanao, Philippines: conservation implications on a partially protected key biodiversity area under threat. Philippine Journal of Systematic Biology Online ISSN. 2024; 17: 29–40.

95. Tenorio EA, Montoya P, Norden N, Rodríguez-Buriticá S, Salgado-Negret B, Gonzalez MA. Mountains exhibit a stronger latitudinal diversity gradient than lowland regions. Journal of Biogeography. 2023; 50(6): 1026–1036.

96. Nogués-Bravo D, Araújo MB, Romdal T, Rahbek C. Scale effects and human impact on the elevational species richness gradients. Nature; 2008; 453(7192): 216–219.

97. Pitogo KME, Saavedra AJ. Conservation assessment of the recently rediscovered Guttman's Stream Frog, *Pulchrana guttmani* (Brown, 2015), reveals the need for its uplisting into a higher threat category. Herpetology Notes. 2023; 16: 717–721.

98. Heaney LR. Conservation biogeography in oceanic archipelagoes. Frontiers of Biogeography, New Directions in the Geography of Nature. Sinauer Associates, Sunderland, MA, 345–360; 2004.

99. McGuire JA, Alcala AC. A taxonomic revision of the flying lizards (Iguania: Agamidae: *Draco*) of the Philippine Islands, with a description of a new species. Herpetological Monographs. 2000; 14: 81–138.

100. Allen D, Oliveros C, Española C, Broad G, Gonzalez JCT. A new species of *Galliralus* from Calayan Island, Philippines. Forktail. 2004; 20: 1–7.

101. Heaney LR, Tabaranza BR, Balete DS, Rigertas N. Synopsis and biogeography of the mammals of Camiguin Island, Philippines. Fieldiana Zoology. 2006; 2006(106): 28–48.

102. Brown RM, Siler CD, Oliveros CH, Diesmos AC, Alcala AC. A new *Gekko* from Sibuyan Island, central Philippines. Herpetologica. 2011; 67(4), 460–476.

103. Oliveros CH, Ota H, Crombie RI, Brown RM. The herpetofauna of the Babuyan group of islands, northern Philippines. Scientific Publications of the Natural History Museum of the University of Kansas. 2011; 43: 1–20.

104. Reyes MC, Ingicco T, Piper PJ, AmanoN, Pawlik AF. First fossil evidence of the extinct Philippine cloud rat *Crateromys paulus* (Muridae: Murinae: Phloeomyini) from Ilin Island, Mindoro, and insights into its Holocene abundance. Proceedings of the Biological Society of Washington. 2017; 130(1): 84–97.

105. Barley AJ, Diesmos AC, Siler CD, Martinez CM, Brown RM. Taxonomic revision of Philippine Sun Skinks (Reptilia: Squamata: Scincidae: *Eutropis*), and descriptions of eight new species. Herpetological Monographs. 2020; 34(1): 39–70.

106. Clores MA, Bautista JB, Fernandez JB, Cuesta MA, Brown RM. Diversity and distribution of amphibians and reptiles in the Caramoan Island Group, Maqueda Channel, southern Luzon, Philippines. Journal of Asia-Pacific Biodiversity. 2021; 14(1): 1–14.

107. Supsup CE, Asis AA, Carestia UV, Puna NM, Avanceña JB. Geographic distribution, population and conservation status of *Tragulus nigricans*, an endangered mouse deer endemic to western Philippines. Pacific Conservation Biology. 2021; 28(1): 90–96.

108. Russell JC, Kueffer C. Island biodiversity in the Anthropocene. Annual Review of Environment and Resources. 2019; 44(1): 31–60.

109. Borges PA, Cardoso P, Gabriel R, Ah-Peng C, Emerson BC. Challenges, advances and perspectives in Island Biogeography. Frontiers of Biogeography. 2016; 8(2).

110. Blades B, Ronquillo C, Hortal J. Mobilisation of data from natural history collections can increase the quality and coverage of biodiversity information. Ecology and Evolution. 2025; 15(4): e71139.

111. Heberling JM, Miller JT, Noesgaard D, Weingart SB, Schigel D. Data integration enables global biodiversity synthesis. Proceedings of the National Academy of Sciences. 2021; 118(6): e2018093118.

112. Amano T, Lamming JD, Sutherland WJ. Spatial gaps in global biodiversity information and the role of citizen science. Bioscience. 2016; 66(5): 393–400.

113. Della Rocca F, Musiani M, Galaverni M, Milanesi P. Improving online citizen science platforms for biodiversity monitoring. Journal of Biogeography. 108; 51(12): 2412–2423.

114. Mason BM, Mesaglio T, Barratt Heitmann J, Chandler M, Chowdhury S, Gorta SBZ, et al. iNaturalist accelerates biodiversity research. BioScience. 2025; biaf104.

115. Barve V. Discovering and developing primary biodiversity data from social networking sites: A novel approach. Ecological Informatics. 2014; 24: 194–199.

116.Chowdhury S, Aich U, Rokonuzzaman M, Alam S, Das P, Siddika A, et al. Increasing biodiversity knowledge through social media: A case study from tropical Bangladesh. BioScience. 2023; 73(6): 453–459.

117. Tabeta MSG, Bejar SGF. Improving our understanding of a cryptic primate, the Philippine Tarsier (*Carlito syrichta*), through social media. International Journal of Primatology. 2025; 1–26.

118.Supan NB, Norcio JCR, Estrella BNV, Elayda DSB, Punzalan JHH, Dagamac NHA. Facebook networks as citizen science tool for snake species monitoring in the Philippines. Journal of Asia-Pacific Biodiversity. In press, 10.1016/j.japb.2025.12.007

119. Madera BS. Population survey of marine turtles in Boracay Island, Philippines using a citizen science approach. Journal of Management and Development Studies. 2019; 8(2): 1–15.

120. Acuña D, Isagani N, Pitogo KME. Predation on a McClung's Philippine false coralsnake, *Hemibungarus mcclungi* (Weigmann 1835), by a giant spiny centipede, *Scolopendra spinosissima* Kraepelin 1903, on Luzon Island, The Philippines. Reptiles & Amphibians. 2021; 28(3): 417–419.

121. Sullivan BL, Aycrigg JL, Barry JH, Bonney RE, Bruns N, Cooper CB, et al. The eBird enterprise: An integrated approach to development and application of citizen science. Biological Conservation. 2014; 169: 31–40.

122. Barcelona JF, Nickrent DL, LaFrankie JV, Callado JRC, Pelser PB. Co's digital flora of the Philippines: Plant identification and conservation through cybertaxonomy. Philippine Journal of Science. 2013; 142: 57–67.

123. Pernat N, Canavan S, Golivets M, Hillaert J, Itescu Y, Jarić I, et al. Overcoming biodiversity blindness: Secondary data in primary citizen science observations. Ecological Solutions and Evidence. 2024; 5(1): e12295.

124. Binaday JWB, Amarga AKS, Barrameda ES, Bonagua BJM. Amphibians and reptiles in the vicinity of Bulusan lake, Bulusan Volcano natural park, Sorsogon, Philippines. Philippine Journal of Science. 2017; 146(3): 339–351.

125. Decena SCP, Macasait Jr DR, Arguelles MS. Species Accounts, assemblage, and microhabitats of amphibians and reptiles of Northeastern Leyte, Philippines. Philippine Journal of Science. 2023; 152(1).

126. Maglangit EPT, Nuñeza OM, Coritico FP, Medecilo-Guiang MMP, Mohagan AB, Patano Jr RR, Amoroso VB. Richness and distribution of reptiles and amphibians in the tropical lowland habitats of Mt. Agad-Agad, Iligan City, Southern Philippines. Reptiles & Amphibians. 2022; 29(1): 413–425.

127. Beck J, Ballesteros-Mejia L, Nagel P, Kitching IJ. Online solutions and the ‘Wallacean shortfall’: what does GBIF contribute to our knowledge of species' ranges?. Diversity and Distributions. 2013; 19(8): 1043–1050.

128. Cadotte MW, Beyene MS, Bowell M, Dalrymple SE, Dechoum MS, Dooner P, et al. Grey matters: Ensuring management information is a part of the permanent evidence base by creating open grey literature principles. Ecological Solutions and Evidence. 2025; 6(2).

129. Wieczorek J, Bloom D, Guralnick R, Blum S, Döring M, Giovanni R, et al. Darwin Core: an evolving community-developed biodiversity data standard. PloS one. 2012; 7(1): e29715.

130. Ball-Damerow JE, Brenskelle L, Barve N, Soltis PS, Sierwald P, Bieler R, et al. Research applications of primary biodiversity databases in the digital age. PloS one. 2019; 14(9): e0215794.

131. Marques N, de Melo Soares CD, de Melo Casali D, Guimarães EC, Fava FG, da Silva Abreu JM, et al. Retrieving biodiversity data from multiple sources: making secondary data standardised and accessible. Biodiversity Data Journal. 2024; 12: e133775.

132. Costello MJ, Wieczorek J. Best practice for biodiversity data management and publication. Biological Conservation. 2014; 173: 68–73.

133. Robinson JG, LaBruna D, O'Brien T, Clyne PJ, Dudley N, Andelman SJ, et al. Scaling up area-based conservation to implement the Global Biodiversity Framework's 30x30 target: The role of Nature's Strongholds. PLoS Biology. 2024; 22(5): e3002613.

134. Buckland ST, Johnston A. Monitoring the biodiversity of regions: Key principles and possible pitfalls. Biological Conservation. 2917; 214: 23–34.

135. Wenk E, Mesaglio T, Keith D, Cornwell W. Curating protected area-level species lists in an era of diverse and dynamic data sources. Ecological Informatics. 2024; 84: 102921.

136. Hoffmann S. Challenges and opportunities of area-based conservation in reaching biodiversity and sustainability goals. Biodiversity and Conservation. 2022; 31(2): 325–352.

137. Britz R, Hundsdörfer A, Fritz U. Funding, training, permits—the three big challenges of taxonomy. Megataxa. 2020; 1(1): 49–52.

138. Dawson NM, Coolsaet B, Bhardwaj A, Booker F, Brown D, Lliso B, et al. Is it just conservation? A typology of Indigenous peoples' and local communities' roles in conserving biodiversity. One Earth. 2024; 7(6): 1007–1021.

139. Abrahamse T, Andrade-Correa MG, Arida C, Galsim R, Häuser C, Price M, Sommerwerk N. The Global Taxonomy Initiative in Support of the Post-2020 Global Biodiversity Framework. CBD Technical Series No. 96. Secretariat of the Convention on Biological Diversity, Montreal, 103 pages; 2021.

140. Soberón J, Peterson AT. Biodiversity informatics: managing and applying primary biodiversity data. Philosophical Transactions of the Royal Society of London. Series B: Biological Sciences. 2004; 359(1444): 689–698.

141. Güntsch A, Overmann J, Ebert B, Bonn A, Le Bras Y, Engel T, et al. National biodiversity data infrastructures: ten essential functions for science, policy, and practice. BioScience. 2025; 75(2): 139–151.

142. Colella JP, Silvestri L, Súzan G, Weksler M, Cook JA, Lessa EP. Engaging with the Nagoya Protocol on Access and Benefit-Sharing: recommendations for noncommercial biodiversity researchers. Journal of Mammalogy. 2023; 104(3): 430–443.

143. Ramírez-Castañeda V, Westeen EP, Frederick J, Amini S, Wait DR, Achmadi AS, et al. A set of principles and practical suggestions for equitable fieldwork in biology. Proceedings of the National Academy of Sciences. 2022, 119(34): e2122667119.

144. DENR-BMB [Department of Environment and Natural Resources - Biodiversity Management Bureau]. Philippine Biodiversity Strategy and Action Plan (Abridged). Department of Environment and Natural Resources - Biodiversity Management Bureau, Quezon City, Metro Manila, 26 pp; 2025.

145. Schmeller DS, Böhm M, Arvanitidis C, Barber-Meyer S, Brummitt N, Chandler M, et al. Building capacity in biodiversity monitoring at the global scale. Biodiversity and Conservation. 2017; 26: 2765–2790.Carroll, S. R., Herczog, E., Hudson, M., Russell, K., & Stall, S. (2021). Operationalizing the CARE and FAIR Principles for Indigenous data futures. *Scientific Data*, 8(1): 108.

146. Carroll SR, Herczog E, Hudson M, Russell K, Stall S. Operationalizing the CARE and FAIR Principles for Indigenous data futures. Scientific Data. 2021; 8(1): 108.

Supporting Information

S1 Fig. Model diagnostic results from DHARMa for the species diversity model of Philippine conservation-relevant areas. (A) Simulated residuals from the fitted model, showing detected quantile deviations. (B) Nonparametric dispersion test. (C) Zero-inflation test. (D) Q–Q plot used to assess deviations from the expected residual distribution, together with tests for uniformity (Kolmogorov–Smirnov), dispersion, and outliers. (E–F) Scaled residuals plotted against predicted values and against each model predictor. Simulation outliers—observations falling outside the range of simulated values—are highlighted as red stars, while red trend lines indicate statistically significant deviations from model expectations. Deviations were primarily associated with the predictor occurrence density, which likely reflects zero inflation arising from undersampled sites rather than true model misspecification. (G) Bivariate relationship between species richness and occurrence density.

S2 Fig. Model diagnostic results from DHARMa for the sampling effort model of Philippine conservation-relevant areas. (A) Simulated residuals from the fitted model, showing detected quantile deviations. (B) Nonparametric dispersion test. (C) Zero-inflation test. (D) Q–Q plot used to assess deviations from the expected residual distribution, together with tests for uniformity (Kolmogorov–Smirnov), dispersion, and outliers. (E–F) Scaled residuals plotted against predicted values and against each model predictor. Simulation outliers—observations falling outside the range of simulated values—are highlighted as red stars, while red trend lines indicate statistically significant deviations from model expectations. (G–H) Bivariate relationship between species richness and each model predictor. Deviations were primarily associated with each predictor, likely reflecting increased variance at large PCAs where fewer observations exist.

S3 Fig. Map of the Philippines, showing (A) overlap between forest cover and Philippine conservation-relevant areas (PCAs); (B) distribution of observed amphibian and squamate reptile diversity across all combined data sources outside PCA boundaries, highlighting that many well-sampled sites with higher observed species richness lie beyond established conservation-relevant areas. Administrative boundary and biodiversity area shapefiles were sourced from NAMRIA and the DENR-BMB via Geoportal Philippines (https://www.geoportal.gov.ph/).

Figure 1

Click here to access/download;Figure;Figure1.tif

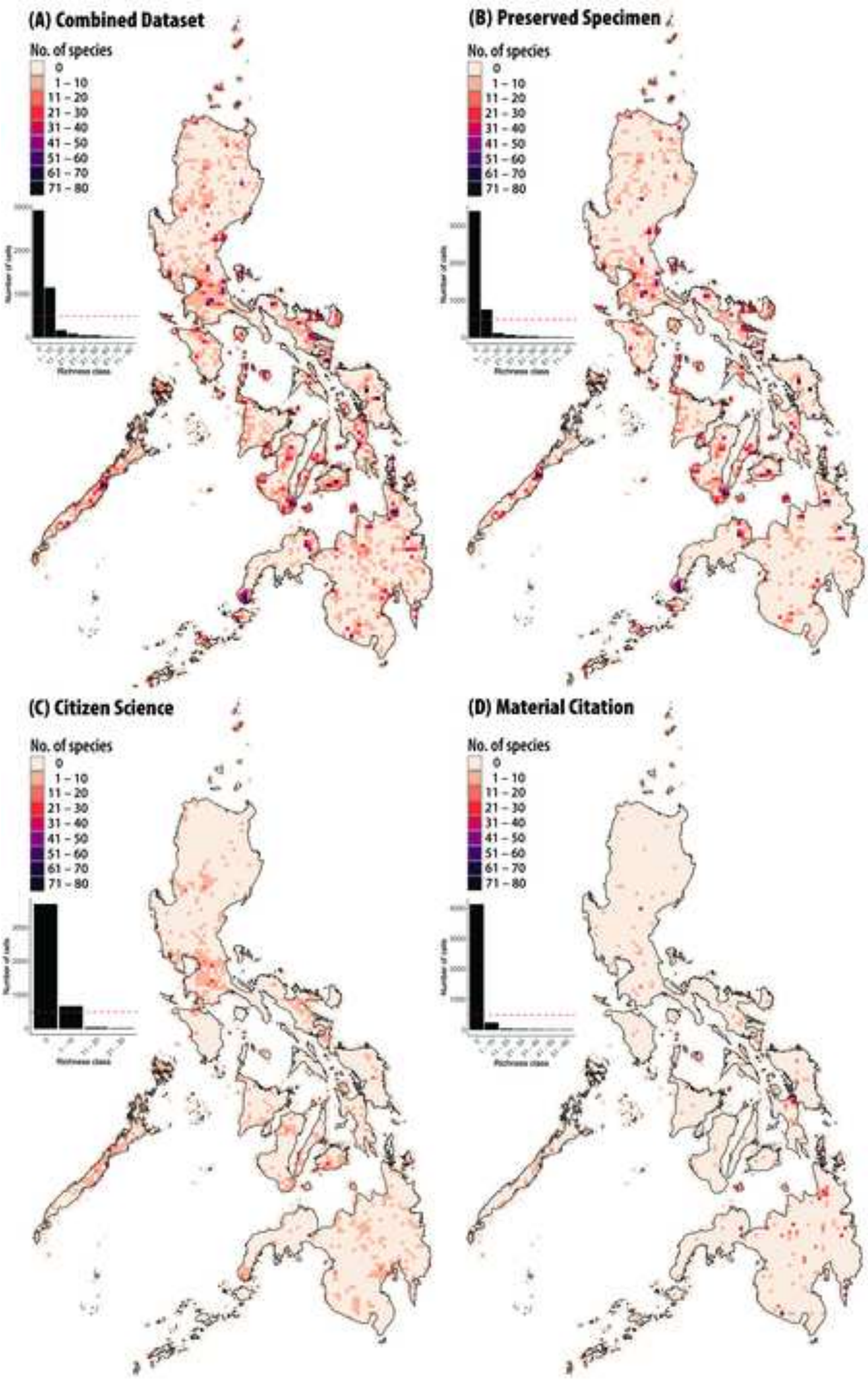

Figure 2

Click here to access/download;Figure;Figure_2.tif

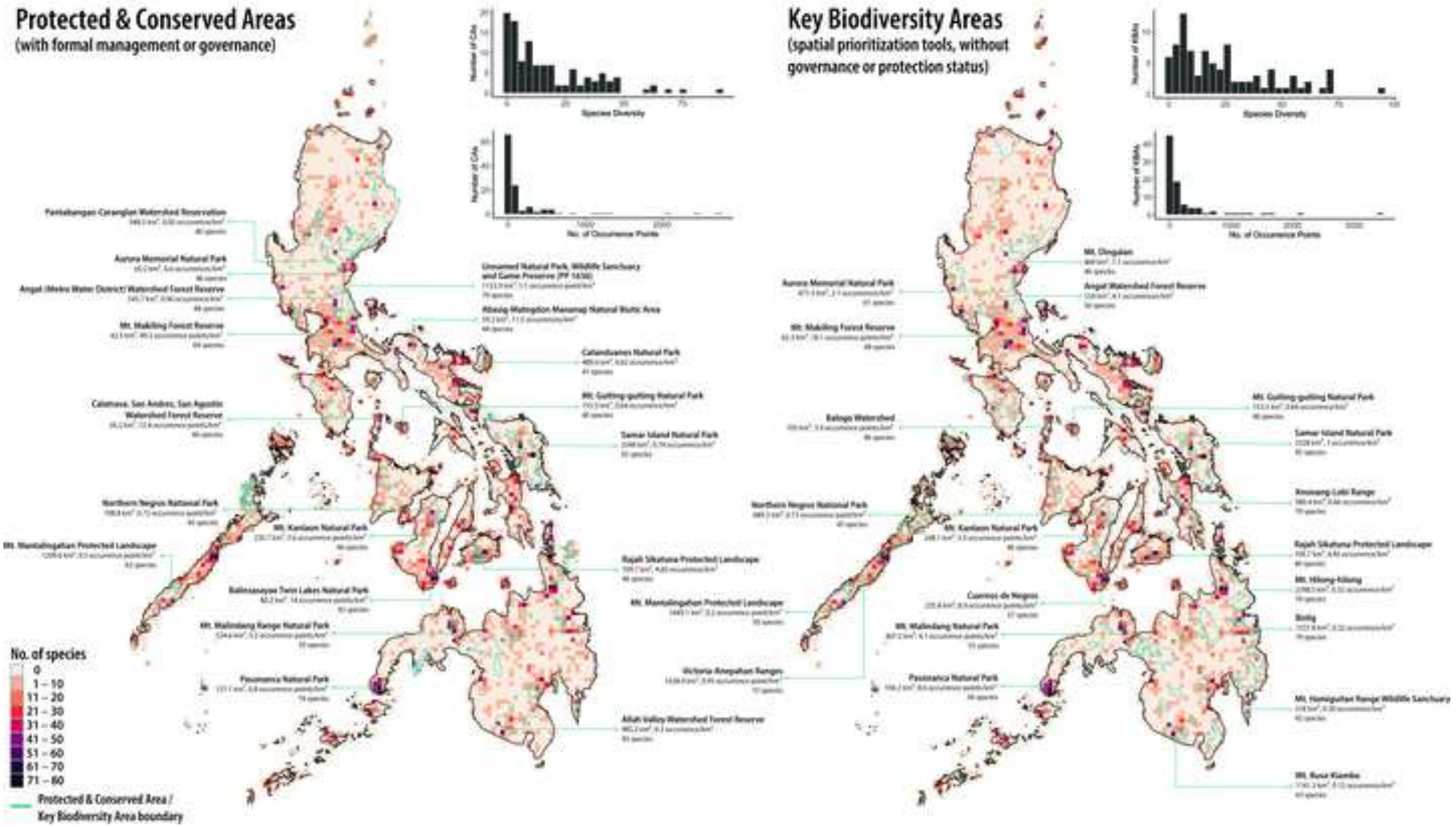

Figure 3

Click here to access/download;Figure;Figure_3.tif

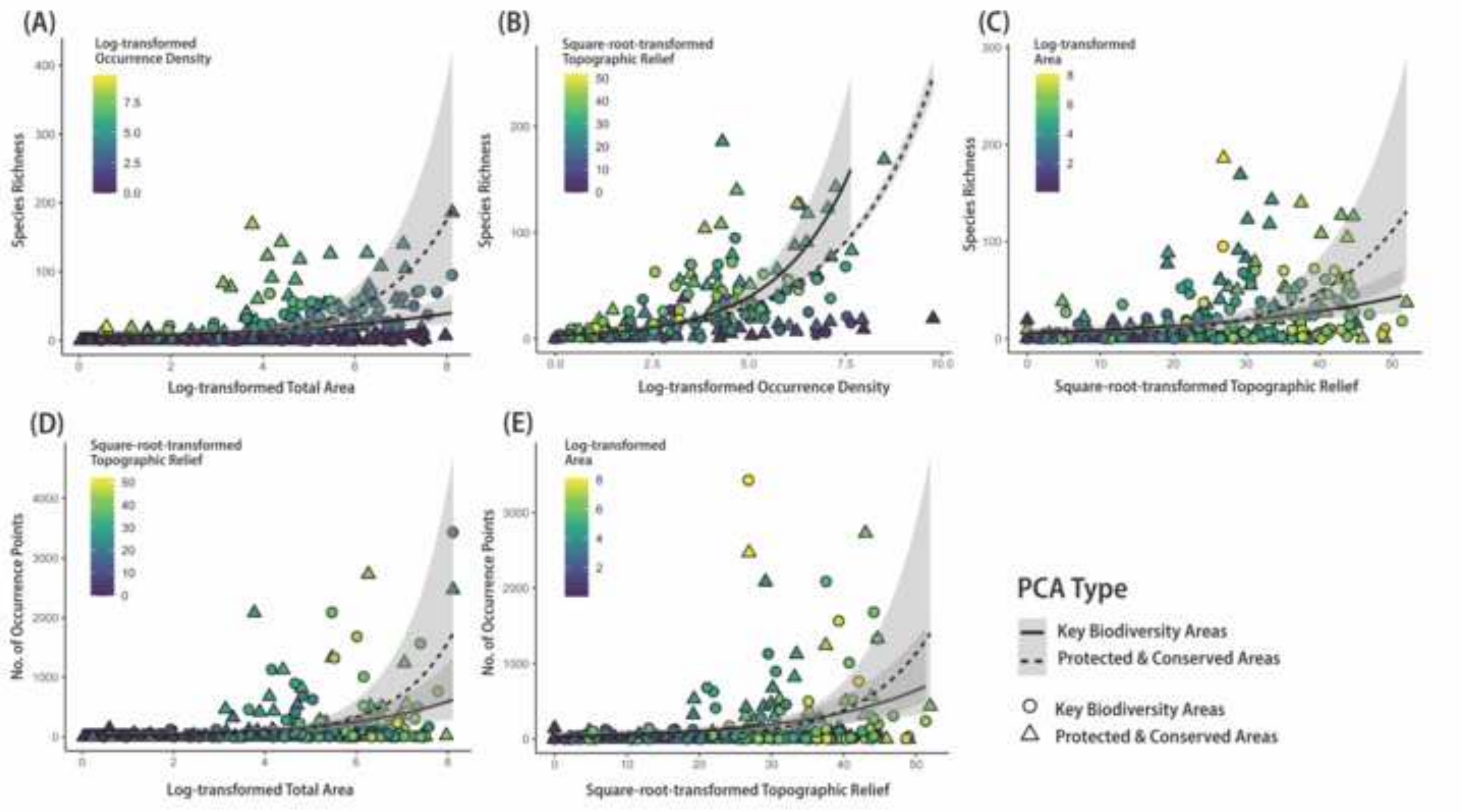

Figure 4

Click here to access/download;Figure;Figure_4.tif

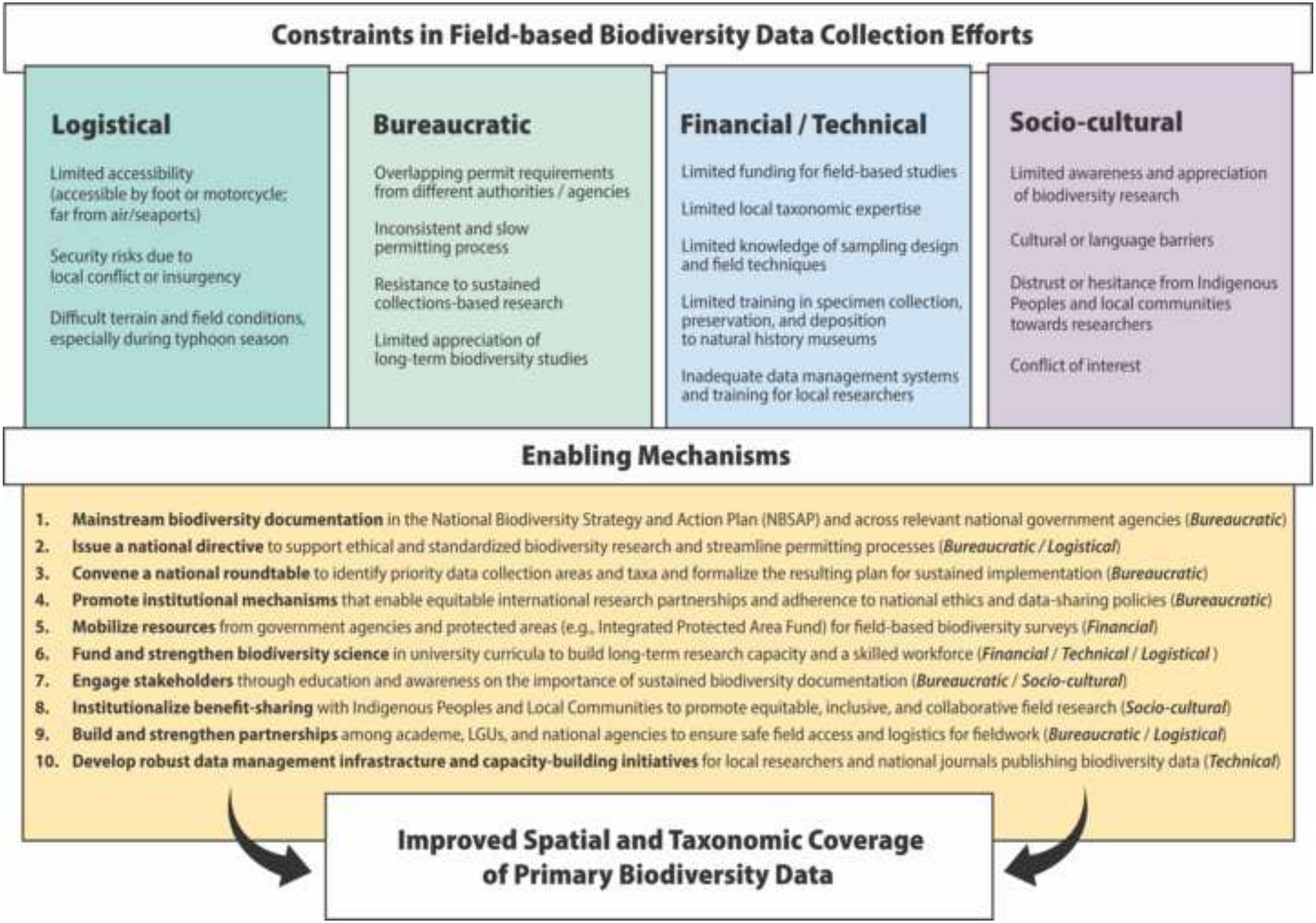

Click here to access/download
Supporting Information
Supporting Information.docx

Sampling bias obscures biodiversity patterns, reveals data gaps in ~~priority~~ <u>a megadiverse island archipelago hotspot</u> ~~conservation areas~~: a call for improved documentation

Kier Mitchel E. Pitogo[1*], Camila G. Meneses[1], Syrus Cesar P. Decena[1,2], Christian E. Supsup[1], Hannah E. Som[1], Justin M. Bernstein[3], Kin Onn Chan[1,#a], Mark William Herr[1], Rafe M. Brown[1]

[1]*Department of Ecology and Evolutionary Biology & Biodiversity Institute, University of Kansas, Lawrence, KS, USA*

[2]*College of Agricultural and Environmental Sciences, Visayas State University-Alangalang, Leyte, Philippines*

[3]*Department of Mammalogy, Division of Vertebrate Zoology, American Museum of Natural History, New York, NY, USA*

[#a]*Current Address: Department of Integrative Biology & MSU Museum, Michigan State University, East Lansing, MI, USA*

*Corresponding author: Kier Mitchel E. Pitogo

E-mail: pitogo.kiermitchel@gmail.com

Abstract

Where and how species are sampled can shape biodiversity knowledge, spatial patterns, and data-driven conservation. In many Global South megadiverse, biodiversity hotspots~~s~~, sampling remains uneven, and available data often lack the synthesis needed to assess region-wide gaps for effective conservation planning and priority-setting. This shortfall is common within areas considered important for biodiversity~~ conserved areas and key biodiversity areas~~. ~~(hereafter 'priority conservation areas' or PCAs).~~ We demonstrate this case in the Philippines, one of the world's most biodiverse countries~~ in the world~~, where longstanding~~ biodiversity~~ research and growing policy momentum support ~~efforts to expand~~expansion ~~coverage~~ of these conservation-relevant areas (hereafter "Philippine conservation-relevant areas" or PCAs) ~~conserved areas~~. ~~Drawing on~~Using over a century of species occurrence records made digitally accessible, which we compiled and manually curated from multiple sources, we ~~these data to assemble and~~ analyzed~~ information on Philippine amphibians and squamate reptiles from multiple sources, assessing~~ country-wide ~~the~~ spatial distribution of observed amphibian and squamate reptile species richness. We also tested whether species richness across PCAs is explained by area, topographic relief, and sampling effort, and whether effects vary between PCA type: ~~diversity in relation to~~ protected and conserved areas (with management or governance schemes) and key biodiversity areas (unmanaged, but priority areas for conservation).~~PCAs.~~ Results reveal strong spatial biases, with preserved specimens comprising ~~the majority of~~~92% of all records and largely shaping species ~~observed diversity~~ richness patterns. Citizen-science data reinforce~~complement~~ already well-sampled regions, ~~while records from~~whereas peer-reviewed literature ~~contribute valuable~~provides valuable documentation in

underpoorly sampled areas. PCAs are proportionally well-sampled, although gaps and biases remain.with -sampling effort and species richness increase with area. However, this positive area effect weakens with increasing topographic relief, which may suggest that large mountain ranges remain documentation blind spots. The species–area relationship and the influence of sampling effort are stronger in protected and conserved areas, whereas topographic relief exerts a stronger effect in key biodiversity areas. Sampling effort and observed diversity were higher in larger PCAs, but this positive area effect diminishes with increasing topographic relief, highlighting large mountain ranges as persistent blind spots in biodiversity documentation. Notably, some areas of higher diversityseveral high-richness areas occur outside established PCAsprotected areas. We discuss implications of these biases and propose enabling mechanisms to improve primary biodiversity data collection. Our findings highlight sampling gaps and biases and underscore the value of integrating multiple data sources. We outline enabling mechanisms to strengthen primary biodiversity data collection, guiding future surveys toward undersampled regions.This study affirms the importance of integrating digitally accessible biodiversity data from multiple sources in revealing sampling gaps and biases, guiding future studies towards poorly sampled areas and informing conservation priorities.

Introduction

Biodiversity data remain unevenly distributed across taxonomic groups and geographic regions, with pronounced biases against global biodiversity hotspots [1–5]. Several factors contribute to these biases, including limited research capacity [6,7], preferential focus on certain taxonomic groups [3,4,8] and charismatic taxa [9,10], ease of site accessibility [11–13], and prevailing

Formatted: Indent: First line: 0"

tendency to conduct research in areas of special interest [14,15]. As a result, many regions and their wildlife remain poorly sampled—and where data exist, they are often not digitally accessible or fall into “biodiversity blind spots”, obscuring biodiversity patterns [5]. These sampling biases can hinder robust macroecological analyses and obstruct evidence-based conservation planning [17–20], particularly at national scales where most conservation policies and management actions are implemented [21–23]. Therefore, identifying where biodiversity data exist, for which taxa, and to what extent is essential for proactively addressing these biases and strengthening the knowledge base needed for effective conservation.

Challenges in biodiversity data availability are persistent even within global biodiversity hotspots, like the Philippines, where gaps and biases in data coverage skew biodiversity knowledge toward certain regions and taxonomic groups [24–26]. As a megadiverse and biogeographically unique country threatened by habitat loss [27,28], the Philippines urgently requires robust and data-informed conservation strategies. However, limited availability and discoverability of biodiversity data, and a lack of information on how these data are spatially distributed, constrains efforts to evaluate sampling completeness and to guide national biodiversity strategies and research priorities [23,29]. The need for synthesized biodiversity data to inform conservation planning is especially urgent, as biodiversity loss and the global momentum under the Kunming-Montreal Global Biodiversity Framework [30,31] have prompted policy responses to expand protected areas ~~(PAs)~~ and recognition of other effective area-based conservation measures, ~~(OECMs),~~ now covering approximately 15% of the Philippines’ land area [32]. This expansion not only demands effective management grounded in ecologically representative, well-connected, and equitably governed networks of ~~PAs and OECMs~~protected

and conserved areas [30] but also must deliver measurable, positive outcomes for biodiversity [33]. A critical first step towards achieving such outcomes is to strengthen biodiversity data by improving sampling coverage and accessibility [21,23,29,34,35–37].

A key step toward ensuring that biodiversity knowledge of a particular area is sufficient to support conservation strategies is to assess where biodiversity data are spatially distributed and how these patterns align with areas considered important for biodiversity (hereafter ‘Philippine conservation-relevant areas’ or PCAs), including conserved areas (PAs and OECMs)protected and conserved areas (with governance and management schemes) and key biodiversity areas (priority areas for conservation, with no management or governance schemes), following standard terminologies [38]. (collectively referred to herein as ‘priority conservation areas’ or PCAs). Such an approach remains lacking for the Philippines despite long-standing efforts to document wildlife in this megadiverse, global biodiversity hotspot [24–26,398–487]. While data deficiencies affect many taxonomic groups, some are better represented due to sustained research and collection efforts. Herpetofauna offer a compelling case for such analysis in the Philippines: the group has benefited from over a century of active and sustained research effort [25,398]. In fact, recent years have seen an acceleration of engagement in amphibian and reptile studies, with increasing numbers of researchers, broader types of investigations, and expanding publication output [25], all suggesting that herpetofauna are a particularly timely and relevant group for such work and that data availability will likely continue to grow. Endemic herpetofauna are also relatively well-sampled genetically, with voucher specimens housed in museum collections [26]; and curated occurrence records are available in substantial volume across peer-reviewed literature [498,5049]. Moreover, amphibians and reptiles have been shown

Commented [PKME1]: Salafsky, N., Mejía Cortez, P., de Meyer, K., Dudley, N., Klimmek, H., Lewis, A., ... & Sharma, M. (2024). A standard lexicon of terms for area-based conservation version 1.0. Conservation Biology, 38(4), e14269. https://doi.org/10.1111/cobi.14269

to serve as effective surrogates for broader vertebrate biodiversity patterns in KBAs across the country [51~~0~~]. Herpetofauna thus provide a valuable lens to evaluate spatial patterns in biodiversity data for improving country-wide biodiversity documentation and ensuring that conservation efforts are grounded in the best available evidence.

Leveraging species data on Philippine herpetofauna spanning ~125 years (1900s–2025) that are made digitally accessible, we compiled, manually curated, and analyzed occurrence records to conduct country-wide spatial assessments of observed ~~diversity~~ species richness of amphibians and squamate reptiles using multiple data sources. Specifically, we ask: (1) How is observed herpetofaunal species richness ~~diversity~~ spatially distributed across the Philippines? (2) How do these patterns differ across datasets derived from museum collections, citizen-science platforms, and peer-reviewed literature? (3) How well do ~~observed diversity patterns coincide with~~area, topographic relief, and sampling intensity shape observed species richness across PCAs? And (4~~) Which PCAs, and which types, are more thoroughly sampled?~~) do these observed richness patterns differ between protected and additional conservation areas (managed) and key biodiversity areas (unmanaged)? Our findings offer novel, long-overdue insights into spatial distribution of biodiversity data in the Philippines, particularly in relation to PCAs. Drawing from our results, we propose and discuss enabling mechanisms to improve primary biodiversity data collection, strengthening the knowledge base necessary in assessing management effectiveness for area-based conservation measures needed in the country [52~~1~~,53~~2~~]. By identifying key data shortfalls and sampling biases, this study contributes to the growing body of empirical evidence underscoring the critical role of integrating digitally

accessible biodiversity data from multiple sources in revealing sampling gaps and biases, guiding future studies towards poorly sampled areas and informing conservation priorities.

Materials & methods

Data assembly and curation

All georeferenced occurrence records for amphibians and squamate reptiles from the Philippines were downloaded from the Global Biodiversity Information Facility (GBIF) on 31 January 2025 (amphibians) and 07 February 2025 (squamate reptiles). To reduce data duplication, we initially excluded records categorized as material citations and retained only human-observation records from iNaturalist (research grade) and HerpWatch Pilipinas. These were then merged with expert-curated species occurrence data from the literature: amphibians from the Amphibians of the Philippines, part I: checklist of the species [49~~8~~] and snakes from the Synopsis of snakes of the Philippines [~~4~~50~~9~~]. To avoid overlap between GBIF records and these curated datasets (also sourced from GBIF), only GBIF records dated post-publication—2015 onwards for amphibians and 2018 onwards for snakes—were retained. For lizards, all GBIF records were included. Additionally, duplicate field numbers from Sam Noble Oklahoma Museum of Natural History and Father Saturnino Urios University-Biodiversity Informatics and Research Center that corresponded to catalogued specimens from the University of Kansas Natural History Museum (KU) were excluded, with only KU records retained. Finally, we incorporated verifiable occurrence records of non-museum-catalogued specimens from peer-reviewed literature compiled by ~~Meneses et a~~a recent review on Philippine herpetology over the last 20 years~~l~~ [25], along with a few additional entries. For consistency in categorization, all

museum records were treated as “preserved specimen” records; iNaturalist and HerpWatch Pilipinas as “citizen science” records; and data from peer-reviewed literature as “material citation” records.

After initial clean-up, we recovered 471 nominal and candidate species of terrestrial amphibians and squamate reptiles that have digitally accessible occurrence records. Occurrence records for each species were then manually curated using currently accepted taxonomic treatments and synonyms, following the AmphibiaWeb [54] for amphibians and The Reptile Database [55] for reptiles . Each species’ occurrence records were mapped in QGIS 3.4 Madeira [56~~3~~] to assess spatial accuracy of each point. Records falling outside the known geographic range of a species were excluded, -following genetically confirmed species boundaries from published literature summarized in the recent Philippine herpetology review paper [25], and those lacking verifiable documentation (e.g., curated vouchered specimens, occurrences within known distribution from peer-reviewed literature)~~were excluded~~. In cases where a formerly widespread species had been taxonomically split, historical records were reassigned to currently accepted species name based on updated diagnostic and distributional information in taxonomic studies. For species with unclear taxonomic or geographic boundaries, only records supported by verifiable data ~~(e.g., curated vouchered specimens, occurrences within known distribution from peer-reviewed literature)~~ were retained. Since expert-curated distributional literature for Philippine lizards is lacking, we adopted an additional measure by comparing our preliminary range estimates with the Global Assessment of Reptile Distributions [57~~4~~–58~~5~~]. This conservative step was taken to minimize potential overestimation of species ~~diversity~~ richness.~~metrics,~~

Formatted: No underline, Font color: Black

Records not identified to the species level were excluded unless a species name or a placeholder name (e.g., sp. + island name, sp. + number) could be confidently assigned without the risk of double-counting (e.g., when only a single species from a genus is known to occur in the area). Given that the frog genus *Platymantis* remains taxonomically unresolved [596–6158], a conservative approach that accounts for this uncertainty was adopted by including records of candidate species occurring in areas with no known range overlap with closely related congeners. This strategy minimizes the risk of double-counting should these populations ultimately represent a single species, while also retaining valuable data that would otherwise be discarded. This decision was informed by phylogenetic evidence, following Brown et al published phylogeny of Ceratobatrachidae [596].

All records lacking locality information were discarded. Additionally, records with coordinates at 16.45°N, 120.55°E and 13°N, 122°E from GBIF were excluded, as the associated locality descriptions did not match the expected geographic locations. -These coordinates appear to be generalized placeholders rather than accurate site data. In the final dataset, we retained records with geographic coordinates consistent with the general (provincial-level) locality information. At this spatial resolution, such records are unlikely to influence country-wide patterns. Although minor imprecision could affect species richness estimates within individual PCAs, any resulting bias is expected to be minimal given the large number of observations per PCA, the integration of multiple data sources, and the overall sample size (n = 316 PCAs) used in the modeling framework.

Statistical analyses

Mapping herpetofaunal diversity across the Philippines

To assess the spatial distribution of observed herpetofaunal species richness, we aggregated occurrence records within a spatial grid and mapped resulting species richness values. Occurrence records for amphibians and squamate reptiles were used to generate a presence–absence matrix (PAM) at a 10-km grid resolution across the Philippines, using prepare_base_pam function from the R package biosurvey [6259]. We selected a 10-km grid resolution for this visual assessment because it better captures coarse-scale patterns across islands of varying sizes: smaller grids (e.g., 5 km) would be too fragmented in larger islands, while larger grids (e.g., 20 km) risked averaging out important spatial variation, especially in smaller islands.

Separate PAMs were created for (1) all data types, (2) preserved-specimen records, (3) citizen-science records, and (4) material-citation records to visually assess if observed species richness patterns differ among datasets used. For each PAM, alpha diversity (the unique number of speciesspecies richness) was calculated per 10-km grid cell, and the results were visualized as spatial maps using QGIS 3.4 Madeira [563]. The resulting maps of herpetofaunal species richness diversity were overlaid with shapefiles of PCAs to assess spatial overlap between species richness and coverage area. Shapefiles for protected and conserved areasCAs were obtained from the World Database on Protected Areas (www.protectedplanet.net) and KBAs key biodiversity areas [630] from Geoportal Philippines (https://www.geoportal.gov.ph/).

To assess whether species richness classes were evenly represented across grid cells, a chi-square goodness-of-fit test was conducted using the chisq.test function in R version 4.3.1 [64~~1~~]. Richness values were grouped into nine discrete classes, and the observed frequency of cells in each class was compared to a uniform distribution, under the assumption that all classes were equally likely. Expected counts were computed by dividing the total number of grid cells by the number of richness classes. All expected frequencies exceeded 5, meeting assumptions of the chi-square test.

Predictors of observed~~herpetofaunal~~ species richness~~diversity~~ across ~~priority~~ conservation areas

Species richness is shaped by a complex interplay of environmental and evolutionary processes. However, the richness values analyzed here reflect observed species counts derived from available occurrence records. Accordingly, our analysis focuses on variation in observed species richness across PCAs, rather than attempting to explain the processes that generate biodiversity per se. Specifically, we examined whether area, topographic relief, and sampling effort help account for variation in observed richness, and whether these relationships differ among PCA types. As a complementary analysis, we also tested whether the number of occurrence records per PCA is predicted by area and topographic relief, and whether these relationships vary across PCA types. Calculations for each variable are outlined below, followed by a description of our modeling framework.

To quantify species occurrence records and observed ~~diversity~~ species richness within PCAs, we spatially intersected occurrence points with PCA boundaries using “Join Attributes by Location” tool in QGIS 3.4 Madeira [56~~3~~] (input: occurrence points; join layer: PCA polygons; predicate: intersects). The resulting dataset was exported as a CSV file and processed in R statistical software [64~~1~~], where records without PCA matches were excluded. Data were grouped by PCA to calculate the number of occurrence records and unique species per PCA. PCAs without intersecting occurrences were retained with zero counts. Polygon area (in km²) was calculated using expanse function in the R package terra [65~~2~~]. Only terrestrial PCAs were included in the analysis, comprising 207 ~~CAs~~ protected and conserved areas and 109 ~~KBAs~~key biodiversity areas.

Topographic relief for each PCA was extracted using a Digital Elevation Model (DEM) of the Philippines downloaded from the CGIAR Consortium for Spatial Information (https://srtm.csi.cgiar.org/). This DEM, provided in Arc/Info Grid format, was clipped to the spatial extents of each PCA. Clipping was performed using QGIS’ ‘Clip Raster by Mask Layer’ tool, with DEM as the input raster and PCA polygon shapefiles as the mask layer. The resulting clipped DEM layers contained elevation data exclusively within each PCA boundary. Subsequently, minimum and maximum elevation values were calculated for each PCA polygon using the ‘Zonal Statistics’ tool in QGIS. The difference between these values was used to quantify topographic relief (in meters) of each PCA.

To test whether area, topographic relief, and sampling intensity explain observed species richness patterns across PCAs, we fitted generalized linear mixed-effects models (GLMMs) with a negative binomial error distribution to account for overdispersion (dispersion parameter = 0.7552; AIC = 1354.7), which improved model fit relative to a Poisson model (dispersion = 2.1871; AIC = 1602.8). The response variable was the total number of species recorded per PCA. Fixed effects included total area (km^2), occurrence density (number of records per km^2), topographic relief (m), and all pairwise interactions among these predictors. Area and topographic relief were used as proxies for landscape features, based on the hypothesis that larger PCAs and those spanning broader elevational gradients — thus encompassing more heterogeneous habitats — support higher species richness [66,67]. Occurrence density served as a proxy for sampling effort. Predictor variables were transformed to improve model fit: area and occurrence density were $\log_{10}$-transformed due to strong right skew, and topographic relief was square-root-transformed due to moderate skew. All predictors were then mean-centered and scaled to facilitate convergence and allow direct comparisons of effect sizes.

Formatted: Space Before: 0 pt, After: 0 pt

Formatted: Font: Not Bold

Formatted: Font: Not Bold

To determine whether observed species richness patterns differ between protected and conserved areas (managed) and key biodiversity areas (unmanaged), PCA type was included as a fixed effect interacting with all main predictors and their pairwise interactions. This unified modeling framework allows direct comparison of effect sizes between designation types, and the interaction terms formally test whether both individual predictors and their interactions vary between PCA types. To account for potential non-independence among PCAs within biogeographic regions (i.e., PCAs share more similarity by virtue of historical biogeographic structuring), we included the Pleistocene Aggregate Island Complex (PAIC; see [27]) as a

Formatted: Font: Not Bold

random intercept in all models. Residual diagnostics using DHARMa indicated minor deviations for occurrence density, likely reflecting zero inflation from undersampled sites rather than model misspecification. Tests of overdispersion and residual uniformity indicated no violations of model assumptions.

Formatted: Indent: First line: 0.5", Space Before: 0 pt, After: 0 pt

To further facilitate interpretation, we explicitly modeled the number of occurrence points to assess the extent to which variation in sampling effort may influence observed species richness patterns, with occurrence counts incorporated as a proxy measure of sampling intensity. Total occurrence records per PCA were modeled as a function of PCA type and its interaction with area, topographic relief, and the area x topographic relief interaction, with PAIC again included as a random effect. A negative binomial error distribution was used to account for overdispersion (dispersion parameter = 1.574; AIC = 2561.9), which substantially improved model fit relative to a Poisson model (dispersion = 660.4; AIC = 102443). DHARMa diagnostics indicated no major violations of model assumptions, although some quantile deviations were observed for the area predictor, likely reflecting increased variance at large PCAs where fewer observations exist. This sampling-effort model allow us to test whether PCAs with lower observed species richness are undersampled.~~To investigate potential drivers of species diversity across PCAs, we fitted generalized linear models with a negative binomial error distribution to account for overdispersion (dispersion parameter < 1, compared to >5 in the Poisson models) and improve model fit (AIC > 1000 for Poisson, but < 1000 for negative binomial models). The response variable was the total number of species recorded per PCA. Fixed predictors included total area (km^2), occurrence density (number of occurrence records per km^2), and topographic relief (m). Total area and topographic relief served as proxies for landscape features, based on~~

Formatted: Normal (Web), Indent: First line: 0.5"

hypotheses that larger areas and those spanning broader elevational gradients — by virtue of having heterogenous habitat – support higher biodiversity [63,64]. Occurrence density was used as proxy for sampling effort. Predictor variables were transformed based on their distribution to improve model fit: area and occurrence density were $\log_{10}$-transformed due to strong right skew, and topographic relief was square-root-transformed due to moderate right skew. All predictors were then mean-centered and scaled to facilitate model convergence and interpretation of coefficients.

Because PCAs differ in their underlying species pools due to historical biogeographic structuring or other evolutionary processes, we initially included the Pleistocene Aggregate Island Complex (PAIC; see [27]) as a random effect for potential non-independence in species diversity. However, PAIC explained less than 1% of the variance and did not improve model performance, so it was excluded from the final model. We then fitted a fully factorial model with negative binomial error and performed stepwise model selection based on Akaike Information Criterion (AIC), using the stepAIC function in the R package MASS [65] (Table S1–S2). Residual diagnostics using DHARMa indicated quantile deviations for the occurrence density predictor, likely attributable to an excess of zero values reflecting undersampling, rather than model misspecification. Tests of overdispersion and residual uniformity did not indicate violations of model assumptions.

In addition to modeling species diversity, total occurrence records per PCA were modeled as a function of total area, topographic relief, and PAIC as fixed predictors, using a negative binomial

Formatted: Indent: First line: 0"

~~error distribution. A two-way interaction between total area and topographic relief improved model fit and was retained in the final model, as supported by model selection based on AIC (Table S3–S4). DHARMa diagnostics indicated no major violations of model assumptions, although quantile deviations were observed for the total area predictor. These deviations likely reflect an exponential increase in predicted values and a widening spread of residuals at higher area values, where fewer PCAs occur and variability is greater. This sampling effort model aimed to determine whether species-poor areas are genuinely less diverse or simply poorly sampled.~~

Generalized linear models were fitted using the R package glmmTMB ~~package~~ [68~~6~~]. Overdispersion was initially assessed using a custom R function that calculates the ratio of the sum of squared Pearson residuals to residual degrees of freedom, with values >1.5 considered indicative of overdispersion requiring correction [69~~7~~]. Model diagnostics for final models were conducted using the DHARMa package [70~~68~~]. Simulated residuals were generated with the simulateResiduals function (plot = TRUE), and goodness-of-fit tests on scaled residuals were done using testZeroInflation, testUniformity, and testDispersion functions. Residuals were also plotted against each fixed predictor using plotResiduals to check for non-random patterns. Variance inflation factors (VIFs) were calculated using the R package performance [71]. In the full interaction model, some terms exhibited elevated VIF values (> 5), which are expected due to the inclusion of higher-order interaction terms and do not indicate problematic collinearity among primary predictors. To validate this, we modeled the main effects only, with species richness as the response variable; all predictors showed low multicollinearity (VIFs < 5). All sampling effort models, with occurrence points as the response variable, showed low VIF values

(< 5.2). All diagnostic outputs and residual plots are provided in S1–S4 Figs. Variance inflation factors (VIFs) for all predictors, calculated using the R package performance [69], were generally within acceptable limits (all VIFs < 5). However, moderate collinearity was observed in KBA species diversity model for the interaction between occurrence density and area (VIF = 6.42), as well as for topographic relief (VIF = 5.34). All diagnostic outputs and residual plots are provided in S1–S4 Figs.

Formatted: Highlight

Formatted: Font color: Red

As a complement to model-based inference, bivariate relationships were visualized using negative binomial generalized linear models (log link) with 95% confidence intervals, fitted separately by PCA type using the geom_smooth function in the R package ggplot2 [72]. bivariate relationships between the response variable and continuous predictors were visually examined. These relationships were plotted using locally estimated scatterplot smoothing (LOESS) with 95% confidence intervals, implemented via the R package ggplot2 [70]. Predictor variables were transformed to match model specifications, and point colors were used to reflect the values of interacting variables with significant effects, unless otherwise stated, aiding interpretation of interaction patterns. For descriptive purposes, Pearson correlation coefficients were calculated for each plot, with significance assessed at α = 0.05 using the cor.test function. All statistical analyses were conducted in R version 4.3.1 [641] and documented in Supplementary Material.

Results

Spatial distribution of herpetofaunal diversity in the Philippines

Spatial distribution of observed herpetofaunal species richness~~diversity~~ in the Philippines is non-uniform and fragmented, with unequal national-scale coverage that varies considerably across islands (Fig 1A). The distribution of species richness classes significantly deviated from a uniform expectation ($\chi^2(8) = 15{,}843$, $p < 0.001$), indicating uneven observed species richness across the archipelago. Some richness classes were overrepresented, while others were underrepresented relative to expectations if all classes were equally likely. Nationally, only ~2% (n = 87) of the 10-km² grids have more than 41 observed species—roughly 50% of maximum richness recorded in any grid—while 31% (n = 1,370) have between 1 and 40 species, and 66.7% (n = 2,914) have zero recorded species.

Formatted: Highlight

Fig 1. Spatial distribution of observed amphibian and squamate reptile ~~diversity~~ species richness for (A) all data types; (B) preserved-specimen records; (C) citizen-science records; and (D) material-citation records. Species richness is calculated per 10-km² grid cell across the Philippines. Inset bar graph shows frequency distribution of grid cells across species richness classes for each dataset. Dashed red line indicates expected frequency for each class under a uniform distribution, as used in chi-square goodness-of-fit tests.

Different data sources provide complementary insights into observed herpetofaunal ~~diversity~~species richness patterns. The majority of occurrence records came from preserved specimens (n = 70,112), followed by citizen science contributions (n = 3,735) and material citations (n = 2,344), while 37 points were unclassified. Both preserved-specimen and citizen-science data represent individual occurrences, while material citations typically reflect species-

level records, as published species inventory studies ~~usually~~ mostly report presence by species rather than by individuals. All three data types significantly deviated from a uniform expectation ($\chi^2(8) = 20{,}186$–$30{,}610$, $p < 0.001$). Because preserved specimens account for 92% of all records, spatial richness patterns largely mirror those derived from the combined dataset (Fig 1B). Citizen science data generally contribute 1–10 species per grid, with a few grids reaching up to 30 species, and tend to complement regions already covered by specimen-based surveys (Fig 1C). Material citation data are concentrated in fewer areas, consistent with the targeted, site-specific nature of biotic inventories (Fig 1D).

~~Priority~~ Philippine conservation-relevant areas (PCAs) ~~-~~were proportionally well-sampled, although distribution of herpetofaunal ~~diversity~~ species richness patterns is uneven and concentrated in few areas (Fig 2). Of the 76,228 curated occurrence records, 21,210 (27.8%) fell within ~~CAs~~ protected and conserved areas and 23,058 (30.2%) within key biodiversity areas~~KBAs~~ —proportions that are disproportionately high relative to the Philippine land area occupied by these designations (12–15% for protected and conserved areas~~CAs~~ and 6.72% for key biodiversity areas~~KBAs~~). Overlaying species richness with ~~PCA~~PCA boundaries ~~boundaries~~ shows that whereas some high-richness grid cells lie within their boundaries~~PCAs~~, many large sites include cells with sparse or no records. Notably, 52.7% of 207 protected and conserved areas~~CAs~~ and 20.2% of 109 key biodiversity areas~~KBAs~~ contain grid cells with zero species records, and an additional 22.4% of protected and conserved areas ~~CAs~~ and 29.4% of ~~KBAs~~ key biodiversity areas contain only up to 10 recorded species. At the same time, several well-sampled areas with high observed ~~diversity~~ species richness (>40 recorded species) occur outside

Formatted: Highlight

Formatted: Highlight

Formatted: Highlight

Formatted: Highlight

established protected and conserved areas~~CAs~~ but within forested areas, many of which partially overlap with key biodiversity areas~~KBAs~~ (S5 Fig).

Fig 2. Relative locations of well-sampled (≥40 species) conserved areas and key biodiversity areas across the Philippines. Total area, occurrence point density, and number of species are included for each area. Base map shows spatial distribution of observed amphibian and squamate reptile diversity for all data types. Inset histogram shows frequency distribution of priority conservation areas by species richness and number of occurrence records

~~**Fig 2. Relative locations of well-sampled (≥40 species) conserved areas and key biodiversity areas across the Philippines**. Total area, occurrence point density, and number of species are included for each area. Base map shows spatial distribution of observed amphibian and squamate reptile diversity for all data types. Inset histogram shows frequency distribution of priority conservation areas by species richness and number of occurrence records.~~

Well-surveyed sites, those with >40 recorded species, make up 8.8% of protected and conserved areas~~CAs~~ and 18.3% of ~~KBAs~~ key biodiversity areas (Fig 2). Particularly species-rich locations (>60 species) include Samar Island Natural Park, Pasonanca Natural Park, Mt. Malindang Natural Park, Mt. Makiling Forest Reserve, and PP1636 (unnamed wildlife sanctuary in southcentral Luzon). These sites have been extensively surveyed, with data available in public repositories like GBIF, though many records remain absent from, or not reported in, peer-reviewed literature (but see [74~~1~~,75~~2~~]). Other well-documented areas combine strong specimen representation in collections with published data: Cuernos de Negros [76~~3~~,77~~4~~], Aurora

Formatted: Highlight

Formatted: Font: Bold

Formatted: Indent: First line: 0"

Formatted: Highlight

Memorial National Park [78~~5~~,79~~6~~], Mt. Busa–Kimba [61~~58~~,79~~77~~], Mt. Guiting-Guiting [80~~78~~,81~~79~~], Mt. Hilong-hilong [60~~57~~,82~~0~~], Pantabangan-Caranglan Watershed Reservation [83~~1~~], and Victoria-Anepahan Ranges [84]. The 40-species cut-off, however, is used here solely as a reference point for discussion and should not be interpreted as a ~~true~~ threshold of "high species diversity", since observed values remain influenced by sampling limitations and potential biases.

Predictors of observed species ~~diversity~~ richness across conservation areas

There was insufficient evidence of a difference in baseline species richness between key biodiversity areas (reference category) and protected and conserved areas (Table 1). In key biodiversity areas, observed species richness increased with total area, topographic relief, and occurrence density. Larger key biodiversity areas supported more species (β_area = 1.2724 ± 0.1407 SE, z = 9.043, p < 0.001; Fig 3A), richness increased strongly with occurrence density (β_occurrence density = 1.5975 ± 0.1084, z = 14.74, p < 0.001; Fig 3B), and areas encompassing greater topographic relief were richer (β_relief = 0.4970 ± 0.1550, z = 3.206, p = 0.001; Fig 3C). Two-way interactions among predictors indicated non-additive effects of landscape structure. Specifically, the positive effect of area on richness weakened in key biodiversity areas with greater topographic relief (area × topographic relief: β = –0.3587 ± 0.1107, z = –3.241, p = 0.001), suggesting that the species–area relationship is less steep in more topographically

Formatted: Space Before: 0 pt, After: 0 pt
Formatted: Highlight
Formatted: Font: Italic
Formatted: Font: Italic
Formatted: Highlight
Formatted: Font: Italic
Formatted: Font: Italic
Formatted: Highlight
Formatted: Highlight
Formatted: Font: Italic
Formatted: Font: Italic

heterogeneous landscapes. Likewise, the positive effect of occurrence density on richness decreased slightly as topographic relief increased (occurrence density × topographic relief: $\beta = -0.3477 \pm 0.1304$, $z = -2.665$, $p = 0.008$).

Formatted: Font: Italic

Formatted: Font: Italic

Formatted: Indent: First line: 0.5", Space Before: 0 pt, After: 0 pt, Line spacing: Double

Relative to key biodiversity areas, protected and conserved areas exhibited significantly steeper species–area relationships (PCA type × area: $\beta = 0.3834 \pm 0.1322$, $z = 2.170$, $p = 0.03$; Fig 3A) and stronger positive effects of occurrence density (PCA type × occurrence density: $\beta = 0.3779 \pm 0.1007$, $z = 2.992$, $p = 0.003$; Fig 3b), whereas the effect of topographic relief on species richness was weaker in protected and conserved areas (PCA type × topographic relief: $\beta = -0.4699 \pm 0.1880$, $z = -2.500$, $p = 0.012$; Fig 3C). None of the higher-order interactions involving PCA type were significant, indicating that the overall interaction structure among species richness drivers was broadly similar between PCA types. The random intercept for biogeographic subregion (PAIC) explained negligible variance in richness (variance = 1.04×10^{-9}; SD = 3.23×10^{-5}), suggesting minimal influence of biogeographic clustering on observed richness.

Formatted: Font: Italic

Formatted: Font: Italic

Formatted: Highlight

Formatted: Font: Italic

Formatted: Font: Italic

Formatted: Highlight

Formatted: Font: Italic

Formatted: Highlight

~~Species diversity in PCAs was best explained by models that included total area, occurrence density, and their interactions with topographic relief, although the structure of these relationships varied. For CAs, the top-ranked model included total area, occurrence density, topographic relief, and an interaction between area and topographic relief (AIC = 875.8; Table S1). Both total area (1.9556 ± 0.1894 SE, z = 10.326, p < 0.01) and occurrence density (1.4503 ± 0.1496 SE, z = 9.696, p < 0.01) were strong positive predictors of species diversity (Table 1).~~

Table 1. Coefficient estimates from a generalized linear mixed-effects model (negative binomial) in which species richness was modeled as a function of area, topographic relief, and occurrence density, including all pairwise interactions among these predictors and their interactions with Philippine conservation-relevant area (PCA) type. Biogeographic subregion was included as a random intercept.Table 1. Coefficient estimates from generalized linear models (negative binomial) relating species diversity to total area, occurrence density, and topographic relief for priority conservation areas in the Philippines.

Formatted: Highlight

Formatted: Font color: Black, Not Highlight

**Predictors**	**Estimate ± SE**	**z-value**	**p-value**
Conserved Areas			
Intercept	1.03970.6405 ± 0.143821423	7.2294.502	<0.01*
Total AreaPCA Type	–0.19131.9556 ± 0.1677894	–1.14010.326	0.2542<0.01*
**Area**	1.2724 ± 0.1407	9.043	<0.01*
Occurrence Density	1.59754503 ± 0.10841496	14.749.696	<0.01*
Topographic Relief	0.49700021 ± 0.15501645	3.2060.013	0.0013*0.989
**Area × Occurrence Density**	0.1916 ± 0.1237	1.550	0.1213
**Area** × Topographic Relief	–0.3587 ± 0.1107	–3.241	0.0012*

Formatted: Font: Not Bold

Formatted: Font: Not Bold

Formatted: Font: Not Bold

**Occurrence Density** × Topographic Relief	–0.3477 ± 0.1304	–2.665	0.0077*
Area PCA Type ×* Topographic ReliefArea	0.3834 0.2322 ± 0.1322430	2.170- 1.624	0.0300*0.104
**PCA Type × Occurrence Density**	0.3779 ± 0.1007	2.992	0.0028*
**PCA Type × Topographic Relief**	–0.4699 ± 0.1083	–2.500	0.0124*
PCA Type × **Area** × **Occurrence Density**	–0.0849 ± 0.1616	–0.526	0.5992
PCA Type × Area × Topographic Relief	0.1943 ± 0.1363	1.425	0.1542
PCA Type × Occurrence Density × Topographic Relief	0.24436 ± 0.1704	1.434	0.1516
Key Biodiversity Areas			
Intercept	2.8210 ± 0.1279	22.049	<0.01*
Total Area	0.8974 ± 0.1553	5.780	<0.01*
Occurrence Density	1.3127 ± 0.1868	7.027	<0.01*
Topographic relief	0.2019 ± 0.1435	1.407	0.1593
Area * Occurrence Density	0.5501 ± 0.2188	2.514	0.0119*
Area * Topographic Relief	-0.4243 ± 0.1439	-2.948	0.0032*
Occurrence Density * Topographic Relief	-0.5392 ± 0.2362	-2.283	0.0225*

Note: Total area and occurrence density were log-transformed, and topographic relief was square-root-transformed; all predictors were scaled and mean-centered. Significance assessed at α = 0.05(*).

Results from model-based inference aligned with patterns observed in scatterplots, which showed moderate positive correlations between species diversity and total area (Pearson's r =

~~0.51, $p < 0.05$; Fig 3A), and a steep but variable trend for occurrence density ($r = 0.53$, $p < 0.05$), particularly at higher values (Fig 3B). A slightly weaker positive correlation was found between species diversity and topographic relief, with an initial increase that flattened at broader elevational ranges ($r = 0.41$, $p < 0.05$; Fig 3C).~~

Fig 3. Bivariate relationships between species richness and total area, occurrence density, and topographic relief, and between sampling effort and area and topographic relief across Philippine conservation-relevant areas (PCAs). Lines represent negative binomial generalized linear model fits (log link) estimated separately by PCA type, with grey bands indicating 95% confidence intervals. Points represent individual PCAs; colors denote interacting predictors. Only occurrence density × topographic relief and area × topographic relief interactions were statistically significant.~~Scatterplots showing species diversity in relation to total area, occurrence density, and topographic relief for conserved areas (top panels) and key biodiversity areas (bottom panels). Dashed line represents LOESS-smoothed trend, with grey shaded area indicating 95% confidence interval. Each point represents a priority conservation area; colors denote interacting variables with significant effects or inclusion in the best-fit generalized linear models. No interactions were significant in the top panels, although area × topographic relief interaction was retained in the best model. Y-axis is fixed at a lower limit of 0 for better visualization. Pearson correlation coefficients (r) and p-values are shown; significance is based on α = 0.05.~~

Formatted: Highlight

Formatted: Font: Not Bold

Formatted: Font: Not Bold

Formatted: Indent: First line: 0.5"

For KBAs, the best-supported model additionally included interactions between topographic relief and both total area and occurrence density (AIC = 779.2; S2 Table). Occurrence density (1.3127 ± 0.1868 SE, $z = 7.027$, $p < 0.01$) was a stronger predictor than total area (0.8974 ± 0.1553 SE, $z = 5.780$, $p < 0.01$), and their interaction was positive (0.5501 ± 0.2188 SE, $z = 2.514$, $p = 0.0119$) (Table 1). Topographic relief had no significant main effect on species diversity, but its interactions with total area (−0.4243 ± 0.1439 SE, $z = -2.948$, $p = 0.0032$) and occurrence density (−0.5392 ± 0.2362 SE, $z = -2.283$, $p = 0.0225$) were both significant and negative. These results suggest that topographic relief may modulate influence of area and sampling effort on observed species diversity in KBAs.

Similar patterns emerged in the scatterplots: species diversity generally increased with total area, though the relationship flattened at intermediate values before rising again ($r = 0.31$, $p < 0.05$; Fig 3D). A steep initial increase was also observed with occurrence density, followed by greater variability at higher densities ($r = 0.46$, $p < 0.05$; Fig 3E). The association between topographic relief and species diversity was also weak but positive ($r = 0.30$, $p < 0.05$; Fig 3F), resembling the pattern observed in CAs.

Predictors of sampling effort across conservation areas

To evaluate potential sampling bias, total occurrence records per PCA were modeled as a function of area, topographic relief, and PCA type. There was insufficient evidence of differences in baseline sampling intensity between key biodiversity areas and protected and

Formatted: Normal, Line spacing: Double

conserved areas (Table 2). In key biodiversity areas, sampling intensity increased marginally with area (β = 0.7267 ± 0.3782, *z* = 1.922, *p* = 0.055; Fig 3D) and topographic relief (β = 0.7181 ± 0.3854, *z* = 1.863, *p* = 0.062; Fig 3E), and none of the interactions with PCA type were significant. The PAIC random intercept accounted for moderate variance in sampling (variance = 0.46; SD = 0.68), indicating geographic clustering in sampling intensity. Together, these findings suggest that differences between PCA types arise from how area and topographic relief shape species richness, rather than from inherent differences in baseline richness levels. Importantly, these patterns are unlikely to reflect systematic differences in sampling intensity.

~~Sampling effort in PCAs was best explained by models that included total area, topographic relief, their interaction, and biogeographic subregion (PAIC) as a categorical predictor (AIC = 1348.4 for CAs; AIC = 1172.9 for KBAs; S3–S4 Tables). In CAs, both total area (1.1765 ± 0.3709 SE, z = 3.172, p = 0.0015) and topographic relief (1.2525 ± 0.3938 SE, z = 3.181, p = 0.0015) were strong positive predictors of sampling effort (Table 2). However, their interaction had a significant negative effect (−0.6413 ± 0.2683 SE, z = −2.390, p = 0.0168), suggesting that the combined influence of total area and topographic relief on sampling density may diminish at high values. Sampling effort was highest in Luzon, the reference level, among all PAICs. No other PAIC differed significantly from Luzon, except West Visayas, which exhibited comparably higher sampling effort (3.3097 ± 0.8625 SE, z = 3.827, p < 0.01).~~

Table 2. Coefficient estimates from a generalized linear mixed-effects model (negative binomial) in which number of occurrence points was modeled as a function of area, topographic relief, area × topographic relief, and their interactions with Philippine

Formatted: Highlight

Formatted: Highlight

Formatted: Highlight

Formatted: Indent: First line: 0"

Formatted: Font: Bold

conservation-relevant area (PCA) type. Biogeographic subregion was included as a random intercept.

~~Coefficient estimates from generalized linear models (negative binomial) relating number of occurrence records to total area and topographic relief for priority~~ conservation-relevant -areas (PCA), with biogeographic subregion as a random effect. ~~in the Philippines.~~

**Predictors**	**Estimate ± SE**	**z-value**	**p-value**
~~Conserved Areas~~			
Intercept	5.00463.3381 ± 0.46613142	10.73762	< 0.01 *
~~Total Area~~PCA Type	-0.50191.1765 ± 0.40343709	-1.2443.172	0.21340015 *
~~Topographic Relief~~Area	0.72671.2525 ± 0.3782938	1.9223.181	0.0547015 *
~~PAIC: Mindanao~~Topographic Relief	0.7181 1343 ± 0.38544606	1.8630.292	0.06247706
~~PAIC: Mindoro~~Area × Topographic Relief	-0.53650.5381 ± 0.31871.5006	-1.6830.359	0.0923.72
~~PAIC: Palawan~~PCA Type × Area	0.16074310 ± 0.47461.0143	0.339425	0.73506709
~~PAIC: Romblon Island Group~~PCA Type × Topographic Relief	0.30171.9644 ± 0.51001.9589	0.5921.003	0.55413160
**PCA Type × Area × Topographic Relief**	0.2050 ± 0.3862	0.531	0.5956

Formatted: Font: Not Bold

PAIC: Sulu Island Group	0.4890 ± 2.8422	0.172	0.8634
PAIC: West Visayas	3.3097 ± 0.8625	3.837	0.0001 *
Area * Topographic Relief	-0.6413 ± 0.2683	-2.390	0.017 *
Key Biodiversity Areas			
Intercept	4.9874 ± 0.3419	14.59	< 0.01 *
Total Area	0.3027 ± 0.2802	1.080	0.28
Topographic Relief	0.4453 ± 0.2790	1.596	0.1105
PAIC: Mindanao	-0.3802 ± 0.4864	0.782	0.4345
PAIC: Mindoro	-0.7675 ± 0.7252	-1.058	0.29
PAIC: Palawan	0.4971 ± 0.8467	0.587	0.5571
PAIC: Romblon Island Group	0.5533 ± 1.1229	0.493	0.6222
PAIC: Sulu Island Group	-4.7948 ± 1.5603	-3.073	0.0021 *
PAIC: West Visayas	1.1147 ± 0.5995	1.859	0.0630
Area * Topographic Relief	-0.3679 ± 0.1937	-1.899	0.0576

Note: Total area was log-transformed and topographic relief was square-root-transformed; continuous predictors were scaled and mean-centered. Significance assessed at $\alpha = 0.05$(*). PAIC = Pleistocene Aggregate Island Complexes. Reference level for PAIC is Luzon.

In KBAs, the same model structure identified a strong positive effect of the intercept (Luzon) and weak individual effects of total area and topographic relief (Table 2). Although total area (0.3027 ± 0.2802 SE, z = 1.080, p = 0.2801) and topographic relief (0.4453 ± 0.2790 SE, z = 1.596, p = 0.1105) were not individually significant, the interaction term showed a marginally significant negative effect (-0.3679 ± 0.1937 SE, z = -1.899, p = 0.0576). Luzon, the reference

level or intercept, had the highest sampling effort among PAICs. Only the Sulu Island Group had significantly lower sampling effort compared to Luzon (–4.7948 ± 1.5603 SE, $z = -3.073$, $p = 0.0021$), possibly reflecting logistical challenges or reduced field access in this region.

These model-based findings were broadly consistent with scatterplot analyses. Among CAs, weak positive correlations were observed between sampling effort and both total area ($r = 0.32$, $p < 0.05$; Fig 4A) and topographic relief ($r = 0.27$, $p < 0.05$; Fig 4B). For KBAs, correlations were weaker ($r = 0.24$ for total area, Fig 4C; and $r = 0.19$ for topographic relief, Fig 4D; both $p < 0.05$), likely reflecting influence of zero values or sampling gaps across several sites.

Formatted: Font color: Red, Highlight

Formatted: Highlight

Fig 4. Scatterplots showing number of occurrence points in relation to total area and topographic relief for conserved areas (top panels) and key biodiversity areas (bottom panels). Dashed line represents LOESS-smoothed trend, with grey shaded area indicating 95% confidence interval. Each point represents a priority conservation area; colors indicate interacting variables with significant effects. Y-axis is fixed at a lower limit of 0 for better visualization. Pearson correlation coefficients (r) and p-values are shown; significance at $\alpha = 0.05$.

Discussion

Uneven spatial diversity pattern reflects sampling biases

Sustained biodiversity documentation across the Philippines has enabled spatial mapping of observed species ~~diversity~~ richness of amphibians and squamate reptiles, revealing stark patterns of sampling biases. More than two-thirds of grid cells contain either zero or only 1–10 documented species, while comparatively fewer cells (~2%) exhibit higher species ~~diversity~~ richness levels. This pronounced disparity reflects persistent spatial biases in biodiversity knowledge. Observed species ~~diversity~~ richness patterns likely indicate where sampling has been more intensive, rather than accurately representing underlying ecological or biogeographic factors—a pattern that is persistent across many biodiversity-rich areas globally [17,20,21,85~~3~~]. Addressing these biases is critical not only for improving knowledge of Philippine biodiversity but also for ensuring that conservation policy, priority-setting, and management decisions rest on a more complete and representative knowledge base.

A recent comprehensive review of Philippine herpetology revealed substantial geographic gaps in field sampling across the archipelago (see [25] for a detailed discussion). Our findings broadly align with these observations but offer additional fine-scale information by integrating curated species point data and grid-based species richness estimates. This approach not only identifies where sampling has occurred but also quantifies its intensity, ~~demonstrating~~ suggesting that higher ~~diversity~~ species richness values are ~~-~~associated with well-sampled sites (i.e., more occurrence points). Notably, we found that a disproportionate share of documented herpetofaunal diversity is concentrated in a few intensively surveyed areas—mostly in Luzon, the largest island, and in West Visayas, comprising most central islands—where collection efforts in recent decades have been more intense [25,39~~8~~]. High-resolution data of this kind are critical for accurately representing spatial patterns of sampling effort, thereby guiding resources

and research efforts towards poorly explored regions. Furthermore, by incorporating species point data from different sources, we recovered diversity data in areas that would otherwise appear as knowledge gaps, such as the Sulu Archipelago in the south [25]; although sampling effort is still disproportionately lower in this region. As such, given that biodiversity data in the Philippines are fragmented, reliance on selective or incomplete datasets for *any* synthesis studies can introduce substantial bias [86~~4~~].

~~Priority~~ Philippine conservation-relevant areas (PCAs) are relatively well-sampled in proportion to their total area, yet herpetofaunal knowledge across them remains limited and uneven. Despite these gains, many PCAs still lack species records altogether or may have data that are not accessible in digital form. Where species data are lacking in PCAs, they are however available outside delineated boundaries, which may suggest persistent issues associated with sampling within PCAs, including permitting and other bureaucratic processes (particularly for legislated protected areas), logistical access, and security constraints [25,60~~57~~,87~~5~~] (see Fig 5). Although well-surveyed sites include some of the country's known PCAs, vast portions of other mountain ranges and many smaller islands remain poorly sampled or lack species data that are readily accessible for broader scientific and conservation use. Expanding targeted field-based surveys beyond established PCAs—and ensuring that resulting data are made publicly and digitally available for mobilization—remains urgently needed to support conservation initiatives grounded in accurate, comprehensive biodiversity knowledge [21,88~~6~~].

Fig 5. Constraints in field-based biodiversity data collection and corresponding enabling mechanisms to improve primary biodiversity data coverage. Each mechanism primarily addresses one or more constraint types.

~~Sampling biases skew knowledge toward large conservation areas~~

Shared drivers but scale differently across conservation-relevant areas

~~Documenting biodiversity is critical in areas where such information can directly inform protection and management decisions. Conserved areas remain central to efforts aimed at safeguarding biodiversity across genetic, species, and ecosystem levels [87,88]. Complementing these, KBAs, though lacking formal protection or management, are identified as priority sites for future conservation efforts [89]. Thus, biodiversity data support monitoring and adaptive management within CAs and guiding prioritization and investment in KBAs. However, digitally accessible records for Philippine herpetofauna show strong uneven documentation: some sites are well-surveyed, others poorly sampled, and many contain no (digitally accessible) records. These disparities mask true biodiversity patterns and pose significant challenges for efficient, evidence-based conservation planning [90].~~

Our unified modeling framework provides insufficient evidence that baseline species richness differs between types of conservation-relevant areas in the Philippines (protected and conserved areas vs. key biodiversity areas) after accounting for area, sampling effort, and

Formatted: Highlight

Formatted: Indent: First line: 0"

topographic relief. However, the strength of these predictors varies significantly between designation types. In both PCA types, area, occurrence density, and topographic relief positively influence observed richness. Yet, the species–area relationship and the effect of sampling effort are significantly stronger in protected and conserved areas, whereas the effect of topographic relief is stronger in key biodiversity areas. Importantly, these patterns cannot be attributed to systematic differences in sampling intensity between designation types. Our sampling effort model showed no baseline difference in number of occurrence points between PCA types, nor any interaction between designation type and area or topographic relief. Thus, the stronger richness–area and richness–sampling relationships observed in protected and conserved areas do not appear to arise from uneven survey effort alone.

The stronger elevational effect in key biodiversity areas suggests that mountainous areas may disproportionately capture high-elevation diversity relative to protected and conserved areas. One possible explanation is that key biodiversity areas are planning tools that target areas of high ecological importance, such as concentrations of endemic or range-restricted taxa, many of which occur in topographically complex regions [89]. As such, their boundary delineations may cover wider topographic relief to cover habitat diversity gradient along elevation. In contrast, protected and conserved area designation involves additional socio-political and land-use considerations that may not always prioritize elevational heterogeneity [90–92]. In the Philippines, nationally legislated protected areas are designated based not only on biodiversity value but also on socio-cultural importance and provision of key ecosystem services, including regional water security, which may be politically influenced [93,94]. In addition, our definition of protected and conserved areas includes other effective area-based conservation measures, such

as Indigenous and Community Conserved Areas and locally managed conservation areas, whose establishment is often motivated by social and governance considerations beyond ecological criteria [92].

Between both designation types, the positive effect of topographic relief weakens as area and sampling intensity increase, as indicated by significant negative interaction terms. This shared pattern contrasts with the common expectation that large, topographically complex areas should support disproportionately high diversity due to habitat heterogeneity [17,66,67,95]. One plausible explanation is that elevational heterogeneity may strongly promote species turnover at smaller spatial extents, but as area increases, much of this gradient may already be represented, leading to partial redundancy in species composition (see scale effects on elevational species richness [96]) . Similarly, in heavily sampled PCAs, additional survey effort may disproportionately document widespread or low-elevation taxa rather than reveal the full complement of high-elevation specialists, many of which are rarely documented and taxonomically undescribed [42,60,62,75,81,97].

Sampling constraints likely further contribute to this pattern. Mountainous regions in the Philippines are frequently remote, difficult to access, and logistically expensive to survey [97] (see Fig 5). Even where PCAs encompass wide topographic relief, field effort may be concentrated in accessible foothill zones, leaving high-elevation habitats underrepresented. This undersampling in high-elevation areas, particularly for herpetofauna, has been pointed out in a recent review of Philippine herpetology [25]. As a result, observed richness may underestimate the contribution of topographically complex systems, systematically biasing biodiversity

Formatted: Indent: First line: 0.5", Space Before: 0 pt, After: 0 pt, Don't add space between paragraphs of the same style

Formatted: Space Before: 0 pt, After: 0 pt, Don't add space between paragraphs of the same style

knowledge away from montane environments despite their well-documented ecological importance and high levels of endemism [38, 98]. Together, these findings suggest that large, mountainous PCAs do not automatically translate into proportionally higher recorded richness — not because they lack diversity, but because the ecological benefits of elevational heterogeneity may be masked by scale-dependent redundancy and uneven sampling coverage.Observed species diversity and sampling effort for herpetofauna were generally higher in larger PCAs. However, this relationship weakens with increasing topographic relief. Our results suggest that although larger areas tend to receive more sampling effort (and have higher observed diversity), this effect weakens in high mountainous areas. The significant negative interaction between topographic relief and total area on observed diversity contrasts with hypothesis that topographic complexity, by virtue of hosting diverse habitat types, should support higher species diversity [17,63,64,91], an inconsistency that likely reflects sampling limitations. Mountainous areas in the Philippines, despite their potential for high species richness, are often poorly sampled likely due to logistical constraints, along with other factors [92] (see Fig 5). This hypothesis is especially supported by our results, which recovered significant negative interaction effects between topographic relief and sampling effort. These challenges systematically bias biodiversity knowledge away from topographically complex areas, which are some of the most ecologically critical and conservation-relevant regions in the country [38,93].

Biodiversity dData limitations are not confined to e not confined to large mountain ranges. . SSmaller conservation areas, particularly those in island environments, also suffer from

Formatted: Font color: Auto

Formatted: Normal (Web), Don't add space between paragraphs of the same style, Border: Top: (No border), Bottom: (No border), Left: (No border), Right: (No border), Between : (No border)

Formatted: Space After: 0 pt

Formatted: Normal (Web), Indent: First line: 0.5", Don't add space between paragraphs of the same style, Border: Top: (No border), Bottom: (No border), Left: (No border), Right: (No border), Between : (No border)

Formatted: Don't add space between paragraphs of the same style

substantial ~~biodiversity~~ knowledge gaps [51~~0~~]. Although this pattern was not statistically prominent in our model-based inference, it was evident in our ~~exploratory data analyses and~~data visualizations (e.g., small-sized, low-topographic-relief PCAs in Fig~~s~~ 3~~–4~~). Many small island conservation areas in the Philippines remain poorly sampled despite yielding newly discovered species and harboring unique, range-restricted taxa [~~78,~~99~~4–~~107~~2~~]. These systems face disproportionate threats from climate-driven sea-level rise, extreme weather events, invasive species, and habitat loss [108~~3~~], yet lack the baseline biodiversity data for adaptive management. Targeted surveys are urgently needed in these areas to document species presence and build taxonomic and ecological knowledge base required for effective conservation in islands [109~~4~~]. Without action, island ecosystems risk becoming critical blind spots in the country's conservation efforts.

Formatted: Highlight

Data gaps and biases persist, but diverse sources can address them

The majority of digitally accessible knowledge on Philippine herpetofauna comes from natural history collections. These collections represent over a century of fieldwork that has shaped taxonomic and systematics knowledge, increasing herpetofaunal diversity estimates in the country [25,39~~8~~]. As such, specimen-associated data serve as a vital resource for improving knowledge on biodiversity patterns [5,110~~05~~]. They also support large-scale ecological studies, which depend on spatially referenced records to examine broad biodiversity trends and guide conservation planning [16,31,111~~06~~]. Although many regions remain underrepresented in biodiversity data, it is noteworthy that areas with limited contemporary surveys—as mentioned earlier, Sulu Archipelago, where fieldwork has been limited ~~-~~[25]—still hold valuable records

preserved through historical collections, many of which are the only verifiable species records for this biogeographic subregion.

In areas where specimens are lacking, citizen science provides a valuable complementary data stream to help address distributional biases. Online biodiversity platforms [11207–11409] and social media [1150–1182] have become particularly useful where museum records are absent or limited. Although still emerging in Philippine herpetology (but see [118–1203,114]), citizen-science contributions are already well established in other taxonomic groups. For example, active birding communities regularly contribute to platforms like eBird [12115] and collaborate with researchers and biodiversity managers to inform site-level conservation efforts in the Philippines [94116]. Another notable initiative is Co's Digital Flora of the Philippines [12217], where citizen scientists and taxonomists work together to maintain a real-time overview of Philippine flora. At least 54% of the country's vascular plant species have been photo-documented, many with associated geographic coordinates [410]. These examples show that citizen-science contributions to observed diversity may be more pronounced in taxonomic groups with active, organized communities driving such efforts. Despite challenges related to data quality and metadata completeness, carefully curated citizen-science records can enhance biodiversity knowledge, particularly in remote or poorly sampled regions [11207,12318].

Another important yet often overlooked source of biodiversity data comes from formal surveys that do not involve specimen collection and eventual deposition in natural history museums [6158,831,12419–1261]. And if there are such records, they are often not digitized and published in publicly accessible domains, especially when deposited in university-based

museums. Many in-country scientists conduct fieldwork that reports valuable records published in peer-reviewed journals, yet these data are rarely archived in open-access databases such as GBIF [127~~2~~]. Such studies frequently document species in poorly sampled areas in the Philippines, providing crucial complementary information [25]. Although valuable for expanding species distribution knowledge, many of these studies lack specific geographic coordinates of areas sampled, limiting their use for geospatial research; thus, submission of spatial occurrences to online databases like GBIF is highly encouraged [36]. In addition, a wealth of biodiversity data remains locked in grey literature—government reports, university theses, and project documents—that are not digitally accessible but are often used in site-level management. Incorporating these sources into public repositories and ensuring they are properly curated would improve national biodiversity coverage [128~~3~~].

Disparate biodiversity data types underscores the need for standardized archiving practices [129~~4~~–131~~26~~] and greater adherence to best-practice guidelines in dealing with big data [20,132~~27~~]. Although these varied data streams help fill distributional gaps, they often differ in quality, accessibility, and curation. These differences are especially true for non-specimen-based data, which typically lack the standardized metadata associated with museum specimens. To maximize their scientific utility, we recommend that non-specimen-based records be accompanied by metadata, including GPS coordinates (including uncertainty), observation date and time, natural history notes, among others. Robust metadata not only enhances credibility and utility of individual records but also facilitates their integration into broader ecological, biogeographic, and conservation research [16].

Scaling biodiversity documentation to meet conservation targets

The Kunming-Montreal Global Biodiversity Framework, adopted in 2022, sets an ambitious goal: to protect 30% of the world's terrestrial and marine ecosystems by 2030, building on the earlier Aichi Target 11 [133~~28~~]. Achieving this target requires more than simply expanding protected area coverage; it also demands effective management that delivers measurable benefits for biodiversity [30]. These outcomes depend on robust, accurate biodiversity data, particularly within ~~PCA~~conservation-relevant areas~~s~~ in the Philippines [34,134~~29~~,135~~0~~]. However, our results show that persistent data gaps and biases, especially in large mountainous and island conservation areas, continue to shape observed biodiversity data patterns. Although many PCAs with apparent data gaps may in fact hold biodiversity data, their lack of digital accessibility limits inclusion in national-scale assessments of sampling effort. This limitation hinders resource allocation for improved documentation, reduces opportunities for external vetting and expert curation of data, and impedes integration into broader datasets that inform national conservation planning [21,31,130,136~~25~~].

Addressing the challenge of insufficient biodiversity documentation requires developing and implementing enabling mechanisms to overcome constraints in site-level primary data collection, which comprises the majority—if not all—of available biodiversity data (Fig 5). This includes prioritizing biodiversity documentation within national conservation policies and planning frameworks (e.g., Philippine Biodiversity Strategy and Action Plan, PBSAP 2024–2040) to strengthen institutional support for field-based activities, especially within PCAs [23]. For example, streamlining research permitting processes, fully aligned with Indigenous Peoples'

Formatted: Highlight

rights and national access and benefit-sharing policies, can reduce bureaucratic barriers to ethical and standardized data collection [36,137~~1~~]. Engaging Indigenous Peoples and local communities as collaborators, and supporting culturally appropriate awareness and communication initiatives, can further foster equitable partnerships and mutual knowledge exchange [138~~2~~]. Financial mechanisms within national legislated Protected Areas~~PAs~~, such as the Integrated Protected Area Fund, can be leveraged to support student-led and locally collaborative field research. Local or subnational government policies and support mechanisms are also critical for ensuring logistical coordination, safe access, and community involvement in fieldwork.

Beyond site-level interventions, targeted national funding for species discovery and taxonomic capacity-building can incentivize early-career researchers to pursue field-based work [137~~1~~,139~~3~~]. These efforts should be complemented by investments in biodiversity science within university curricula [1~~3~~40], ensuring students are trained in both theory and practice. Addressing the limited data management capacity among local researchers is also critical for promoting effective data-sharing, enhancing the verifiability and utility of biodiversity studies. Comprehensive training programs and standardized data systems can help overcome this barrier, ensuring the production of high-quality, accessible datasets. In parallel, the increasing diversity of data contributors underscores the need for a streamlined and curated national biodiversity data infrastructure [141~~35~~]. Such a platform should ensure that biodiversity data, especially those generated from PCAs, are digitally and publicly accessible, enabling diverse stakeholders to collectively contribute to and benefit from biodiversity knowledge. Ultimately, these in-country initiatives must be paired with stronger institutional mechanisms, such as equitable collaboration standards and data-sharing agreements, to ensure that non-monetary benefits from biological

resource use are meaningfully shared with local collaborators and inform national policy and conservation planning [142~~36~~].

Finally, scaling biodiversity documentation beyond existing PCAs can guide the identification of new priority sites for protected area establishment. Our findings show that several well-sampled, high-diversity areas remain outside current PCAs (neither protected/conserved nor delineated as priority areas for conservation), many overlapping with only partially protected key biodiversity areas~~KBAs~~. This mismatch~~,~~ underscores~~ing~~ opportunities to align future protected and conserved area~~CA~~ expansion (i.e., protected area, other effective area-based conservation measures) ~~with empirically documented biodiversity patterns~~with empirically documented biodiversity patterns, particularly in remaining forested regions. ~~.~~The updated national biodiversity strategy and action plan (PBSAP 2024–2040) targets protecting 24% of terrestrial areas through protected and conserved areas~~CAs~~ [143~~37~~], yet only ~15% are currently designated~~are currently protected~~. Prioritizing well-sampled, high-diversity areas for protection will be critical to achieving this goal. Our results, however, only focus on herpetofauna and similar data gaps and biases likely affect many taxonomic groups in the Philippines [24,26] reinforcing the need for broader, inclusive biodiversity documentation. Building a robust and representative biodiversity knowledge base—well-curated and aligned with FAIR (findable, accessible, interoperable, reusable) and CARE (collective benefit, authority to control, responsibility, ethics) principles—will require sustained collaboration among scientists, conservation practitioners, institutions, and critically, with Indigenous Peoples and local communities [16,144~~38~~,145~~39~~]. Strengthening such collective and inclusive efforts is

essential for transforming biodiversity knowledge into tangible conservation outcomes and ensuring the Philippines meets its national and global biodiversity commitments.

Acknowledgements

During the preparation of this manuscript, KMEP was partially supported by a Graduate Research Assistantship from the University of Kansas Department of Ecology and Evolutionary Biology; and RMB was supported by a Fulbright International Scholar Award administered by the U.S. Department of State, the Philippine-American Education Foundation (PAEF) and the Philippine Council for International Exchange of Scholars. Support for fieldwork that generated many specimen-associated records was provided by the U.S. National Science Foundation (DEB 073199, 0743491, 1418895, 0910341, 0804115, 1654388, and 0334952 and EF-0334952 to RMB and KU-based students), the KU College of Liberal Arts and Sciences Graduate Assistantship in Support of Faculty Research Award, a KU Biodiversity Institute Research and Exploration Fund grant, the South, Southeast, and East Asia Fund of the KU Office of International Affairs, and the General Research Fund of the University of Kansas. We also thank Marites Bonachita Sanguila, Michael Clores, Michael Cuesta, Aljohn Jay Saavedra, and Tristan Luap Senarillos for numerous discussions and their valuable insights on the challenges of primary biodiversity data collection in the Philippines. Finally, we would like to thank the Editor and two reviewers for their constructive feedback, which greatly improved the manuscript.

References

Formatted: Line spacing: Double

Formatted: Font: 18 pt, Bold

1. Hortal J, De Bello F, Diniz-Filho JAF, Lewinsohn TM, Lobo JM, & Ladle RJ. Seven shortfalls that beset large-scale knowledge of biodiversity. Annual Review of Ecology, Evolution, and Systematics. 2015; 46(1): 523–549.
2. Hughes AC, Orr MC, Ma K, Costello MJ, Waller J, Provoost P, et al. Sampling biases shape our view of the natural world. Ecography. 2021; 44(9): 1259–1269.
3. Moura MR, Jetz W. Shortfalls and opportunities in terrestrial vertebrate species discovery. Nature Ecology & Evolution. 2021; 5(5): 631–639.
4. Ondo I, Dhanjal-Adams KL, Pironon S, Silvestro D, Colli-Silva M, Deklerck V, et al. Plant diversity darkspots for global collection priorities. New Phytologist. 2024; 244(2): 719–733.
5. Ball L, Rodríguez-Machado S, Paredes-Burneo D, Rutledge S, Boyd DA, Vander Pluym D, et al. What 'unexplored' means: mapping regions with digitized natural history records to look for 'biodiversity blindspots'. PeerJ. 2025; 13: e18511.
6. Barber PH, Ablan-Lagman MCA, Berlinck RG, Cahyani D, Crandall ED, Ravago-Gotanco R, et al. Advancing biodiversity research in developing countries: the need for changing paradigms. Bulletin of Marine Science. 2014; 90(1): 187–210.
7. Zhang L, Yang L, Chapman CA, Peres CA, Lee TM, Fan PF. Growing disparity in global conservation research capacity and its impact on biodiversity conservation. One Earth. 2023; 6(2): 147–157.
8. Titley MA, Snaddon JL, Turner EC. Scientific research on animal biodiversity is systematically biased towards vertebrates and temperate regions. PloS one. 2017; 12(12): e0189577.

9. Troudet J, Grandcolas P, Blin A, Vignes-Lebbe R, Legendre F. Taxonomic bias in biodiversity data and societal preferences. Scientific Reports. 2017; 7(1): 9132.

10. Guénard B, Hughes AC, Lainé C, Cannicci S, Russell BD, Williams GA. Limited and biased global conservation funding means most threatened species remain unsupported. Proceedings of the National Academy of Sciences. 2025; 122(9): e2412479122.

11. Oliveira U, Paglia AP, Brescovit AD, de Carvalho CJ, Silva DP, Rezende DT, et al. The strong influence of collection bias on biodiversity knowledge shortfalls of Brazilian terrestrial biodiversity. Diversity and Distributions. 2016; 22(12): 1232–1244.

12. Mandeville CP, Nilsen EB, Finstad AG. Spatial distribution of biodiversity citizen science in a natural area depends on area accessibility and differs from other recreational area use. Ecological Solutions and Evidence. 2022; 3(4): e12185.

13. Penhacek M, Castro-Souza RA, Tessarolo G, Diniz-Filho JA, Sobral-Souza T, de Jesus Rodrigues D. Biases in amphibian sampling in the Amazon: Using infrastructure and accessibility data to identify sampling gaps. Biotropica. 2025; 57(5): e70079.

14. Sastre P, Lobo JM. Taxonomist survey biases and the unveiling of biodiversity patterns. Biological Conservation. 2009; 142(2): 462–467.

15. Mentges A, Blowes SA, Hodapp D, Hillebrand H, Chase JM. Effects of site-selection bias on estimates of biodiversity change. Conservation Biology. 2021; 35(2): 688–698.

16. Jetz W, McPherson JM, Guralnick RP. Integrating biodiversity distribution knowledge: toward a global map of life. Trends in Ecology & Evolution. 2012; 27(3): 151–159.

17. Engemann K, Enquist BJ, Sandel B, Boyle B, Jørgensen PM, Morueta-Holme N, et al. Limited sampling hampers "big data" estimation of species richness in a tropical biodiversity hotspot. Ecology and Evolution. 2015; 5(3): 807–820.

18. Santini L, Antão LH, Jung M, Benítez-López A, Rapacciuolo G, Di Marco M, et al. The interface between macroecology and conservation: existing links and untapped opportunities. Frontiers of Biogeography. 2021; 13(4): e53025.

19. Diniz-Filho JAF, Jardim L, Guedes JJ, Meyer L, Stropp J, Frateles LEF, et al. Macroecological links between the Linnean, Wallacean, and Darwinian shortfalls. Frontiers of Biogeography, 2023; 15(2): e59566.

20. Hughes AC, Dorey JB, Bossert S, Qiao H, Orr MC. Big data, big problems? How to circumvent problems in biodiversity mapping and ensure meaningful results. Ecography. 2024; 2024(8): e07115.

21. Grattarola F, Martínez-Lanfranco JA, Botto G, Naya DE, Maneyro R, Mai P, et al. Multiple forms of hotspots of tetrapod biodiversity and the challenges of open-access data scarcity. Scientific Reports. 2020; 10(1): 22045.

22. Perino A, Pereira HM, Felipe-Lucia M, Kim H, Kühl HS, Marselle MR, et al. Biodiversity post-2020: Closing the gap between global targets and national-level implementation. Conservation Letters. 2021; 15(2): e12848.

23. Soberón J. Biodiversity informatics for public policy. The case of CONABIO in Mexico. Biodiversity Informatics. 2022; 17.

24. Berba CMP, Matias AMA. State of biodiversity documentation in the Philippines: Metadata gaps, taxonomic biases, and spatial biases in the DNA barcode data of animal and plant taxa in the context of species occurrence data. PeerJ. 2022; 10: e13146.

25. Meneses CG, Pitogo KME, Supsup CE, Brown RM. Philippine herpetology (Amphibia, Reptilia), 20 years on: two decades of progress towards an increasingly collaborative,

equitable, and inclusive approach to the study of the archipelago's amphibians and reptiles. ZooKeys. 2024; 1190, 213–257.

26. Pitogo KME. Gaps and biases in vertebrate wildlife genetics from a global biodiversity hotspot. Environmental Conservation. 2025; 52(3): 127–138.

27. Brown RM, Siler CD, Oliveros CH, Esselstyn JA, Diesmos AC, Hosner PA, et al. Evolutionary processes of diversification in a model island archipelago. Annual Review of Ecology, Evolution, and Systematics. 2013; 44(1): 411–435.

28. Huais PY, Osorio-Olvera L, Cordier JM, Tomba AN, Soberón J, Loyola R, Nori J. Rethinking global hotspots for threatened terrestrial vertebrates. Global Ecology and Biogeography. 2025; 34(1): e13942.

29. Soberón J, Peterson AT. Monitoring biodiversity loss with primary species-occurrence data: toward national-level indicators for the 2010 target of the convention on biological diversity. AMBIO: A Journal of the Human Environment. 2009; 38(1): 29–34.

30. CBD [Convention on Biological Diversity] [cited 01 August 2025]. Decision adopted by the Conference of the Parties to the Convention on Biological Diversity CBD/COP/DEC/15/4 Kunming-Montreal Global Biodiversity Framework. 2022; CBD/COP/DEC/15/4. Available from: https://www.cbd.int/doc/decisions/cop-15/cop-15-dec-04-en.pdf

31. Orr MC, Hughes AC, Costello MJ, Qiao H. Biodiversity data synthesis is critical for realizing a functional post-2020 framework. Biological Conservation. 2022; 274: 109735.

32. ASEAN Centre for Biodiversity. ASEAN Biodiversity Outlook 3. Philippines, pp. 392; 2023.

33. Maxwell SL, Cazalis V, Dudley N, Hoffmann M, Rodrigues AS, Stolton S, et al. Area-based conservation in the twenty-first century. Nature. 2020; 586(7828): 217–227.

34. Mallari NAD, Collar NJ, McGowan PJ, Marsden SJ. Science-driven management of protected areas: a Philippine case study. Environmental Management. 2013; 51(6): 1236–1246.

35. Jetz W, McGeoch MA, Guralnick R, Ferrier S, Beck J, Costello MJ, et al. Essential biodiversity variables for mapping and monitoring species populations. Nature Ecology & Evolution. 2019; 3(4): 539–551.

36. Hochkirch A, Samways MJ, Gerlach J, Böhm M, Williams P, Cardoso P, et al. A strategy for the next decade to address data deficiency in neglected biodiversity. Conservation Biology. 2021; 35(2): 502–509.

37. Urbano F, Viterbi R, Pedrotti L, Vettorazzo E, Movalli C, Corlatti L. Enhancing biodiversity conservation and monitoring in protected areas through efficient data management. Environmental Monitoring and Assessment. 2024; 196(1): 12.

38. Salafsky N, Mejía Cortez P, de Meyer K, Dudley N, Klimmek H, Lewis A, et al. A standard lexicon of terms for area-based conservation version 1.0. Conservation Biology. 2024; 38(4): e14269.

39. Brown RM, Diesmos AC, Alcala AC. The state of Philippine herpetology and the challenges for the next decade. Silliman Journal. 2001; 42(1): 18–87.

40. Heaney LR. A decade of research on Philippine mammals: progress and challenges. Silliman Journal. 2001; 42(1): 88–107.

41. Pelser PB, Barcelona JF, Nickrent DL . Co's Digital Flora of the Philippines. Available from: www. philippineplants.org.

42. Heaney LR, Balete DS, Rickart EA. The mammals of Luzon Island: biogeography and natural history of a Philippine fauna. JHU Press; 2016.

43. Tanalgo KC, Hughes AC. Bats of the Philippine Islands—A review of research directions and relevance to national-level priorities and targets. Mammalian Biology. 2018; 91(1): 46–56.

44. Allen D. Birds of the Philippines. Lynx and Birdlife International Field Guides, Lynx Edicions, Barcelona, Spain; 2020.

45. Gamalo LE, Sabanal B, Ang A. Three decades of Philippine nonhuman primate studies: research gaps and opportunities for Philippine primatology. Primates. 2021; 62(1): 233–239.

46. delos Angeles MD, Radbouchoom S, Ngarega BK, Lapuz RS, Schneider H. Spatial diversity and distribution of fern and lycophyte species in karst and non-karst landscapes towards conservation needs. Frontiers in Plant Science. 2025; 16: 1495796.

47. Fernandez DAP, Do Linh San E, Forget PM, Veron G. Carnivorans of the Philippines: current knowledge and research gaps. Journal of Mammalogy. 2025; gyaf020.

48. Balisco RAT, Liao TY. An annotated checklist of fish species described from the Philippines. ZooKeys. 2025; 1246: 1–287.

49. Diesmos AC, Watters JL, Huron NA, Davis DR, Alcala AC, Crombie RI, et al. Amphibians of the Philippines, part I: checklist of the species. Proceedings of the California Academy of Sciences. 2015; 62(20): 457–539.

50. Leviton AE, Siler CD, Weinell JL, Brown RM. Synopsis of the snakes of the Philippines. Proceedings of the California Academy of Sciences. 2018; 64(14): 399–568.

51. Fidelino JS, Constantino RRP, Duya MRM. Cross-taxon congruence of terrestrial vertebrates across Philippine Key Biodiversity Areas. Journal for Nature Conservation. 2025; 86: 126907.

52. Mallari NAD, Collar NJ, McGowan PJ, Marsden SJ. Philippine protected areas are not meeting the biodiversity coverage and management effectiveness requirements of Aichi Target 11. Ambio. 2016; 45(3): 313–322.

53. Struebig MJ, Lee JS, Deere NJ, Gevaña DT, Ingram DJ, Lwin N. Drivers and solutions to Southeast Asia's biodiversity crisis. Nature Reviews Biodiversity. 2025;1: 497–514.

54. AmphibiaWeb. Berkeley, California: University of California; 2025 [cited 2025 Jun 25]. Available from: https://amphibiaweb.org

55. Uetz P, Freed P, Aguilar R, Reyes F, Kudera J, Hošek J, editors. The Reptile Database. 2025 [cited 2025 Jun 14]. Available: http://www.reptile-database.org

56. QGIS Development Team. QGIS Geographic Information System. Open Source Geospatial Foundation Project; 2018. Available from: http://qgis.osgeo.org.

57. Roll U, Feldman A, Novosolov M, Allison A, Bauer AM, Bernard R, et al. The global distribution of tetrapods reveals a need for targeted reptile conservation. Nature Ecology & Evolution. 2017; 1(11): 1677–1682.

58. Caetano GHDO, Chapple DG, Grenyer R, Raz T, Rosenblatt J, Tingley R, et al. Automated assessment reveals that the extinction risk of reptiles is widely underestimated across space and phylogeny. PLoS Biology. 2022; 20(5): e3001544.

59. Brown RM, Siler CD, Richards SJ, Diesmos AC, Cannatella DC. Multilocus phylogeny and a new classification for Southeast Asian and Melanesian forest frogs (family Ceratobatrachidae). Zoological Journal of the Linnean Society. 2015; 174(1): 130–168.

60. Sanguila MB, Cobb KA, Siler CD, Diesmos AC, Alcala AC, Brown RM. The amphibians and reptiles of Mindanao Island, southern Philippines, II: the herpetofauna of northeast Mindanao and adjacent islands. ZooKeys. 2016; (624), 1–132.

61. Pitogo KME, Saavedra AJL, Afuang LE. Amphibians and reptiles of Mount Busa, Sarangani Province: a glimpse of the herpetological community of southern Mindanao, Philippines. Philippine Journal of Science. 2021; 150(5): 1279–1306.

62. Nuñez-Penichet C, Cobos ME, Soberón J, Gueta T, Barve N, Barve V, Navarro-Sigüenza AG, Peterson AT. Selection of sampling sites for biodiversity inventory: Effects of environmental and geographical considerations. Methods in Ecology and Evolution. 2022; 00:1–13.

63. Ong PS, Afuang LE, Rosell-Ambal RG. Philippine Biodiversity Conservation Priorities: A second iteration of the National Biodiversity Strategy and Action Plan. Department of Environment and Natural Resources-Protected Areas and Wildlife Bureau, Conservation International Philippines, Biodiversity Conservation Program-University of the Philippines Center for Integrative and Development Studies, and Foundation for the Philippine Environment. Quezon City, Philippines; 2002.

64. R Core Team (2022). R: A language and environment for statistical computing. R Foundation for Statistical Computing, Vienna, Austria. Available from: https://www.R-project.org/.

65. Hijmans R._terra: Spatial Data Analysis_. R package version 1.7-78; 2025. Available from: https://CRAN.R-project.org/package=terra.

66. Ricklefs RE, Lovette IJ. The roles of island area per se and habitat diversity in the species–area relationships of four Lesser Antillean faunal groups. Journal of Animal Ecology. 1999; 68(6): 1142–1160.

67. Gotelli NJ. A Primer of Ecology, 4th ed. Sunderland, MA: Sinauer Associates; 2008.

68. Brooks ME, Kristensen K, Van Benthem KJ, Magnusson A, Berg CW, Nielsen A, et al. glmmTMB balances speed and flexibility among packages for zero-inflated generalized linear mixed modeling. The R Journal. 2017; 9(2): 378–400.

69. Zuur AF, Ieno EN, Walker N, Saveliev AA, Smith GM, Zuur AF, et al. GLM and GAM for count data. Mixed effects models and extensions in ecology with R, 209–243; 2009.

70. Hartig F. _DHARMa: Residual Diagnostics for Hierarchical (Multi-Level / Mixed) Regression Models_. R package version 0.4.7; 2024. Available from: https://CRAN.R-project.org/package=DHARMa.

71. Lüdecke D, Ben-Shachar MS, Patil I, Waggoner P, Makowski D. performance: An R Package for Assessment, Comparison and Testing of Statistical Models. Journal of Open Source Software. 2021; 6(60): 3139.

72. H. Wickham. ggplot2: Elegant Graphics for Data Analysis. Springer-Verlag New York; 2016.

73. Nuñeza OM, Ates FB, Alicante AA. Distribution of endemic and threatened herpetofauna in Mt. Malindang, Mindanao, Philippines. Biodiversity and Conservation. 2010; 19, 503–518.

74. Gonzalez JCT, de Guia APO, Dimalibot JC, Pantua KV, Gustilo WO, Bantayan NC. Understorey to canopy vertebrate fauna of a lowland evergreen forest in Mt. Makiling Forest Reserve, Philippines. Biodiversity Data Journal. 72; 8: e56999.

75. Brown WC, Alcala AC. Populations of amphibians and reptiles in the submontane and montane forests of Cuernos de Negros, Philippine Islands. Ecology. 1961; 628–636.

76. Brown WC, Alcala AC. The zoogeography of the Philippine Islands, a fringing archipelago. Proceedings of the California Academy of Sciences. 1970; 38: 105–130.

77. Brown RM, McGuire JA, Ferner JW, Icarangal Jr N, Kennedy RS. Amphibians and reptiles of Luzon Island, II: preliminary report on the herpetofauna of Aurora Memorial National Park, Philippines. Hamadryad. 2000; 25:175–195

78. Siler CD, Welton LJ, Siler JM, Brown J, Bucol A, Diesmos AC, Brown RM. Amphibians and Reptiles, Luzon Island, Aurora Province and Aurora Memorial National Park, Northern Philippines: New island distribution records. Check List. 2011; 7(2): 182–195.

79. Brown RM. A new species of stream frog of the genus *Hylarana* from the mountains of southern Mindanao Island, Philippines. Herpetologica. 2015; 71(3): 223–233.

80. Siler CD, Welton LJ, Diesmos AC, Siler JM, Bucol A, Brown J, Brown RM. Amphibians and reptiles, Romblon Island Group, central Philippines: comprehensive herpetofaunal inventory. Check List. 2012; 8(3): 443–462.

81. Meneses CG, Siler CD, Alviola PA, Gonzalez JCT, Balatibat JB, Natividad CA, Brown RM. Amphibian and reptile diversity along a ridge-to-reef elevational gradient on a small isolated oceanic island of the central Philippines. Check List. 2012; 18(5): 941–948.

82. Plaza JL, Sanguila MB. Preliminary report on the anurans of Mount Hilong-hilong, Agusan del Norte, eastern Mindanao, Philippines. Asian Herpetological Research. 2015; 6(1): 18–33.

83. Gojo-Cruz PHP, Afuang LE, Gonzalez JCT, Gruezo WS. Amphibians and reptiles of Luzon Island, Philippines: the herpetofauna of Pantabangan-Carranglan watershed,

Nueva Ecija Province, Caraballo Mountain range. Asian Herpetological Research. 2018; 9(4): 201–223.

84. Supsup CE, Asis AA, Carestia Jr UV, Diesmos AC, Mallari NAD, Brown RM. Variation in species richness, composition and herpetological community structure across a tropical habitat gradient of Palawan Island, Philippines. Herpetozoa. 2020; 33: 95–111.

85. Azovsky AI. Species–area and species–sampling effort relationships: disentangling the effects. Ecography. 2011; 34(1): 18–30.

86. Pitogo KME, Meneses CG, Flores ABA, Saavedra AJL, Amarga AKS, Angeles MD, et al. Caught in statistical noise: pitfalls of a unidimensional approach to understanding biodiversity-conflict relationships. npj Biodiversity. 2025; 4(1): 17.

87. Brown RM, Meneses CG, Wood Jr PL, Fernandez JB, Cuesta MA, Clores MA, et al. Unexpected discovery of another new species of Philippine false gecko (Gekkonidae; *Pseudogekko*) from the Bicol Peninsula of Luzon Island. Herpetologica. 2020; 76(3): 315–329.

88. Di Minin E, Toivonen T. Global protected area expansion: creating more than paper parks. BioScience. 2015; 65(7): 637–638.

89. Eken G, Bennun L, Brooks TM, Darwall W, Fishpool LD, Foster M, et al. Key biodiversity areas as site conservation targets. BioScience. 2004; 54(12): 1110–1118.

90. Watson JE, Dudley N, Segan DB, Hockings M. The performance and potential of protected areas. Nature. 2014; 515(7525): 67–73.

91. Jonas HD, Ahmadia GN, Bingham HC, Briggs J, Butchart SHM, Carino J, Chassot O, Chaudhary S, et al. Equitable and effective areas-based conservation: Towards the conserved area paradigm. PARKS. 2021; 21: 71–84.

92. Zhang Y, West P, Thakholi L, Suryawanshi K, Supuma M, Straub D, Sithole SS, Sharma R, et al. Governance and conservation effectiveness in protected areas and indigenous and locally managed areas. Annual Review of Environment and Resources. 2023; 48(1): 559–588.

93. DENR [Department of Environment and Natural Resources]. Clarifying the procedural guidelines in the conduct of Protected Area Suitability Assessment (PASA). DENR Administrative Order No. 2016-04, 11 pp; 2017.

94. Pitogo KME, Jarvis F, Saavedra AJL, Senarillos TL, Concepcion CB, Simpson PM. Avian fauna of Mount Melibengoy, southern Mindanao, Philippines: conservation implications on a partially protected key biodiversity area under threat. Philippine Journal of Systematic Biology Online ISSN. 2024; 17: 29–40.

95. Tenorio EA, Montoya P, Norden N, Rodríguez-Buriticá S, Salgado-Negret B, Gonzalez MA. Mountains exhibit a stronger latitudinal diversity gradient than lowland regions. Journal of Biogeography. 2023; 50(6): 1026–1036.

96. Nogués-Bravo D, Araújo MB, Romdal T, Rahbek C. Scale effects and human impact on the elevational species richness gradients. Nature; 2008; 453(7192): 216–219.

97. Pitogo KME, Saavedra AJ. Conservation assessment of the recently rediscovered Guttman's Stream Frog, *Pulchrana guttmani* (Brown, 2015), reveals the need for its uplisting into a higher threat category. Herpetology Notes. 2023; 16: 717–721.

98. Heaney LR. Conservation biogeography in oceanic archipelagoes. Frontiers of Biogeography, New Directions in the Geography of Nature. Sinauer Associates, Sunderland, MA, 345–360; 2004.

99. McGuire JA, Alcala AC. A taxonomic revision of the flying lizards (Iguania: Agamidae: *Draco*) of the Philippine Islands, with a description of a new species. Herpetological Monographs. 2000; 14: 81–138.

100. Allen D, Oliveros C, Española C, Broad G, Gonzalez JCT. A new species of *Galliralus* from Calayan Island, Philippines. Forktail. 2004; 20: 1–7.

101. Heaney LR, Tabaranza BR, Balete DS, Rigertas N. Synopsis and biogeography of the mammals of Camiguin Island, Philippines. Fieldiana Zoology. 2006; 2006(106): 28–48.

102. Brown RM, Siler CD, Oliveros CH, Diesmos AC, Alcala AC. A new *Gekko* from Sibuyan Island, central Philippines. Herpetologica. 2011; 67(4), 460–476.

103. Oliveros CH, Ota H, Crombie RI, Brown RM. The herpetofauna of the Babuyan group of islands, northern Philippines. Scientific Publications of the Natural History Museum of the University of Kansas. 2011; 43: 1–20.

104. Reyes MC, Ingicco T, Piper PJ, AmanoN, Pawlik AF. First fossil evidence of the extinct Philippine cloud rat *Crateromys paulus* (Muridae: Murinae: Phloeomyini) from Ilin Island, Mindoro, and insights into its Holocene abundance. Proceedings of the Biological Society of Washington. 2017; 130(1): 84–97.

105. Barley AJ, Diesmos AC, Siler CD, Martinez CM, Brown RM. Taxonomic revision of Philippine Sun Skinks (Reptilia: Squamata: Scincidae: *Eutropis*), and descriptions of eight new species. Herpetological Monographs. 2020; 34(1): 39–70.

106. Clores MA, Bautista JB, Fernandez JB, Cuesta MA, Brown RM. Diversity and distribution of amphibians and reptiles in the Caramoan Island Group, Maqueda Channel, southern Luzon, Philippines. Journal of Asia-Pacific Biodiversity. 2021; 14(1): 1–14.

107. Supsup CE, Asis AA, Carestia UV, Puna NM, Avanceña JB. Geographic distribution, population and conservation status of *Tragulus nigricans*, an endangered mouse deer endemic to western Philippines. Pacific Conservation Biology. 2021; 28(1): 90–96.

108. Russell JC, Kueffer C. Island biodiversity in the Anthropocene. Annual Review of Environment and Resources. 2019; 44(1): 31–60.

109. Borges PA, Cardoso P, Gabriel R, Ah-Peng C, Emerson BC. Challenges, advances and perspectives in Island Biogeography. Frontiers of Biogeography. 2016; 8(2).

110. Blades B, Ronquillo C, Hortal J. Mobilisation of data from natural history collections can increase the quality and coverage of biodiversity information. Ecology and Evolution. 2025; 15(4): e71139.

111. Heberling JM, Miller JT, Noesgaard D, Weingart SB, Schigel D. Data integration enables global biodiversity synthesis. Proceedings of the National Academy of Sciences. 2021; 118(6): e2018093118.

112. Amano T, Lamming JD, Sutherland WJ. Spatial gaps in global biodiversity information and the role of citizen science. Bioscience. 2016; 66(5): 393–400.

113. Della Rocca F, Musiani M, Galaverni M, Milanesi P. Improving online citizen science platforms for biodiversity monitoring. Journal of Biogeography. 108; 51(12): 2412–2423.

114. Mason BM, Mesaglio T, Barratt Heitmann J, Chandler M, Chowdhury S, Gorta SBZ, et al. iNaturalist accelerates biodiversity research. BioScience. 2025; biaf104.

115. Barve V. Discovering and developing primary biodiversity data from social networking sites: A novel approach. Ecological Informatics. 2014; 24: 194–199.

116. Chowdhury S, Aich U, Rokonuzzaman M, Alam S, Das P, Siddika A, et al. Increasing biodiversity knowledge through social media: A case study from tropical Bangladesh. BioScience. 2023; 73(6): 453–459.

117. Tabeta MSG, Bejar SGF. Improving our understanding of a cryptic primate, the Philippine Tarsier (*Carlito syrichta*), through social media. International Journal of Primatology. 2025; 1–26.

118. Supan NB, Norcio JCR, Estrella BNV, Elayda DSB, Punzalan JHH, Dagamac NHA. Facebook networks as citizen science tool for snake species monitoring in the Philippines. Journal of Asia-Pacific Biodiversity. In press, 10.1016/j.japb.2025.12.007

119. Madera BS. Population survey of marine turtles in Boracay Island, Philippines using a citizen science approach. Journal of Management and Development Studies. 2019; 8(2): 1–15.

120. Acuña D, Isagani N, Pitogo KME. Predation on a McClung's Philippine false coralsnake, *Hemibungarus mcclungi* (Weigmann 1835), by a giant spiny centipede, *Scolopendra spinosissima* Kraepelin 1903, on Luzon Island, The Philippines. Reptiles & Amphibians. 2021; 28(3): 417–419.

121. Sullivan BL, Aycrigg JL, Barry JH, Bonney RE, Bruns N, Cooper CB, et al. The eBird enterprise: An integrated approach to development and application of citizen science. Biological Conservation. 2014; 169: 31–40.

122. Barcelona JF, Nickrent DL, LaFrankie JV, Callado JRC, Pelser PB. Co's digital flora of the Philippines: Plant identification and conservation through cybertaxonomy. Philippine Journal of Science. 2013; 142: 57–67.

123.Pernat N, Canavan S, Golivets M, Hillaert J, Itescu Y, Jarić I, et al. Overcoming biodiversity blindness: Secondary data in primary citizen science observations. Ecological Solutions and Evidence. 2024; 5(1): e12295.

124. Binaday JWB, Amarga AKS, Barrameda ES, Bonagua BJM. Amphibians and reptiles in the vicinity of Bulusan lake, Bulusan Volcano natural park, Sorsogon, Philippines. Philippine Journal of Science. 2017; 146(3): 339–351.

125. Decena SCP, Macasait Jr DR, Arguelles MS. Species Accounts, assemblage, and microhabitats of amphibians and reptiles of Northeastern Leyte, Philippines. Philippine Journal of Science. 2023; 152(1).

126. Maglangit EPT, Nuñeza OM, Coritico FP, Medecilo-Guiang MMP, Mohagan AB, Patano Jr RR, Amoroso VB. Richness and distribution of reptiles and amphibians in the tropical lowland habitats of Mt. Agad-Agad, Iligan City, Southern Philippines. Reptiles & Amphibians. 2022; 29(1): 413–425.

127. Beck J, Ballesteros-Mejia L, Nagel P, Kitching IJ. Online solutions and the 'Wallacean shortfall': what does GBIF contribute to our knowledge of species' ranges?. Diversity and Distributions. 2013; 19(8): 1043–1050.

128. Cadotte MW, Beyene MS, Bowell M, Dalrymple SE, Dechoum MS, Dooner P, et al. Grey matters: Ensuring management information is a part of the permanent evidence base by creating open grey literature principles. Ecological Solutions and Evidence. 2025; 6(2).

129. Wieczorek J, Bloom D, Guralnick R, Blum S, Döring M, Giovanni R, et al. Darwin Core: an evolving community-developed biodiversity data standard. PloS one. 2012; 7(1): e29715.

130. Ball-Damerow JE, Brenskelle L, Barve N, Soltis PS, Sierwald P, Bieler R, et al. Research applications of primary biodiversity databases in the digital age. PloS one. 2019; 14(9): e0215794.

131. Marques N, de Melo Soares CD, de Melo Casali D, Guimarães EC, Fava FG, da Silva Abreu JM, et al. Retrieving biodiversity data from multiple sources: making secondary data standardised and accessible. Biodiversity Data Journal. 2024; 12: e133775.

132. Costello MJ, Wieczorek J. Best practice for biodiversity data management and publication. Biological Conservation. 2014; 173: 68–73.

133. Robinson JG, LaBruna D, O'Brien T, Clyne PJ, Dudley N, Andelman SJ, et al. Scaling up area-based conservation to implement the Global Biodiversity Framework's 30x30 target: The role of Nature's Strongholds. PLoS Biology. 2024; 22(5): e3002613.

134. Buckland ST, Johnston A. Monitoring the biodiversity of regions: Key principles and possible pitfalls. Biological Conservation. 2917; 214: 23–34.

135. Wenk E, Mesaglio T, Keith D, Cornwell W. Curating protected area-level species lists in an era of diverse and dynamic data sources. Ecological Informatics. 2024; 84: 102921.

136. Hoffmann S. Challenges and opportunities of area-based conservation in reaching biodiversity and sustainability goals. Biodiversity and Conservation. 2022; 31(2): 325–352.

137. Britz R, Hundsdörfer A, Fritz U. Funding, training, permits—the three big challenges of taxonomy. Megataxa. 2020; 1(1): 49–52.

138. Dawson NM, Coolsaet B, Bhardwaj A, Booker F, Brown D, Lliso B, et al. Is it just conservation? A typology of Indigenous peoples' and local communities' roles in conserving biodiversity. One Earth. 2024; 7(6): 1007–1021.

139. Abrahamse T, Andrade-Correa MG, Arida C, Galsim R, Häuser C, Price M, Sommerwerk N. The Global Taxonomy Initiative in Support of the Post-2020 Global Biodiversity Framework. CBD Technical Series No. 96. Secretariat of the Convention on Biological Diversity, Montreal, 103 pages; 2021.

140. Soberón J, Peterson AT. Biodiversity informatics: managing and applying primary biodiversity data. Philosophical Transactions of the Royal Society of London. Series B: Biological Sciences. 2004; 359(1444): 689–698.

141. Güntsch A, Overmann J, Ebert B, Bonn A, Le Bras Y, Engel T, et al. National biodiversity data infrastructures: ten essential functions for science, policy, and practice. BioScience. 2025; 75(2): 139–151.

142. Colella JP, Silvestri L, Súzan G, Weksler M, Cook JA, Lessa EP. Engaging with the Nagoya Protocol on Access and Benefit-Sharing: recommendations for noncommercial biodiversity researchers. Journal of Mammalogy. 2023; 104(3): 430–443.

143. DENR-BMB [Department of Environment and Natural Resources - Biodiversity Management Bureau]. Philippine Biodiversity Strategy and Action Plan (Abridged). Department of Environment and Natural Resources - Biodiversity Management Bureau, Quezon City, Metro Manila, 26 pp; 2025.

144. Schmeller DS, Böhm M, Arvanitidis C, Barber-Meyer S, Brummitt N, Chandler M, et al. Building capacity in biodiversity monitoring at the global scale. Biodiversity and Conservation. 2017; 26: 2765–2790.Carroll, S. R., Herczog, E., Hudson, M., Russell, K., & Stall, S. (2021). Operationalizing the CARE and FAIR Principles for Indigenous data futures. *Scientific Data*, 8(1): 108.

145. Carroll SR, Herczog E, Hudson M, Russell K, Stall S. Operationalizing the CARE and FAIR Principles for Indigenous data futures. Scientific Data. 2021; 8(1): 108.

References

1. Hortal J, De Bello F, Diniz-Filho JAF, Lewinsohn TM, Lobo JM, & Ladle RJ. Seven shortfalls that beset large-scale knowledge of biodiversity. Annual Review of Ecology, Evolution, and Systematics. 2015; 46(1): 523–549.
2. Hughes AC, Orr MC, Ma K, Costello MJ, Waller J, Provoost P, et al. Sampling biases shape our view of the natural world. Ecography. 2021; 44(9): 1259–1269.
3. Moura MR, Jetz W. Shortfalls and opportunities in terrestrial vertebrate species discovery. Nature Ecology & Evolution. 2021; 5(5): 631–639.
4. Ondo I, Dhanjal-Adams KL, Pironon S, Silvestro D, Colli-Silva M, Deklerck V, et al. Plant diversity darkspots for global collection priorities. New Phytologist. 2024; 244(2): 719–733.
5. Ball L, Rodríguez-Machado S, Paredes-Burneo D, Rutledge S, Boyd DA, Vander Pluym D, et al. What ‘unexplored’ means: mapping regions with digitized natural history records to look for ‘biodiversity blindspots’. PeerJ. 2025; 13: e18511.
6. Barber PH, Ablan-Lagman MCA, Berlinck RG, Cahyani D, Crandall ED, Ravago-Gotanco R, et al. Advancing biodiversity research in developing countries: the need for changing paradigms. Bulletin of Marine Science. 2014; 90(1): 187–210.

7. Zhang L, Yang L, Chapman CA, Peres CA, Lee TM, Fan PF. Growing disparity in global conservation research capacity and its impact on biodiversity conservation. One Earth. 2023; 6(2): 147–157.

8. Titley MA, Snaddon JL, Turner EC. Scientific research on animal biodiversity is systematically biased towards vertebrates and temperate regions. PloS one. 2017; 12(12): e0189577.

9. Troudet J, Grandcolas P, Blin A, Vignes-Lebbe R, Legendre F. Taxonomic bias in biodiversity data and societal preferences. Scientific Reports. 2017; 7(1): 9132.

10. Guénard B, Hughes AC, Lainé C, Cannicci S, Russell BD, Williams GA. Limited and biased global conservation funding means most threatened species remain unsupported. Proceedings of the National Academy of Sciences. 2025; 122(9): e2412479122.

11. Oliveira U, Paglia AP, Brescovit AD, de Carvalho CJ, Silva DP, Rezende DT, et al. The strong influence of collection bias on biodiversity knowledge shortfalls of Brazilian terrestrial biodiversity. Diversity and Distributions. 2016; 22(12): 1232–1244.

12. Mandeville CP, Nilsen EB, Finstad AG. Spatial distribution of biodiversity citizen science in a natural area depends on area accessibility and differs from other recreational area use. Ecological Solutions and Evidence. 2022; 3(4): e12185.

13. Penhacek M, Castro-Souza RA, Tessarolo G, Diniz-Filho JA, Sobral-Souza T, de Jesus Rodrigues D. Biases in amphibian sampling in the Amazon: Using infrastructure and accessibility data to identify sampling gaps. Biotropica. 2025; 57(5): e70079.

14. Sastre P, Lobo JM. Taxonomist survey biases and the unveiling of biodiversity patterns. Biological Conservation. 2009; 142(2): 462–467.

15. Mentges A, Blowes SA, Hodapp D, Hillebrand H, Chase JM. Effects of site-selection bias on estimates of biodiversity change. Conservation Biology. 2021; 35(2): 688–698.

16. Jetz W, McPherson JM, Guralnick RP. Integrating biodiversity distribution knowledge: toward a global map of life. Trends in Ecology & Evolution. 2012; 27(3): 151–159.

17. Engemann K, Enquist BJ, Sandel B, Boyle B, Jørgensen PM, Morueta-Holme N, et al. Limited sampling hampers "big data" estimation of species richness in a tropical biodiversity hotspot. Ecology and Evolution. 2015; 5(3): 807–820.

18. Santini L, Antão LH, Jung M, Benítez-López A, Rapacciuolo G, Di Marco M, et al. The interface between macroecology and conservation: existing links and untapped opportunities. Frontiers of Biogeography. 2021; 13(4): e53025.

19. Diniz-Filho JAF, Jardim L, Guedes JJ, Meyer L, Stropp J, Frateles LEF, et al. Macroecological links between the Linnean, Wallacean, and Darwinian shortfalls. Frontiers of Biogeography, 2023; 15(2): e59566.

20. Hughes AC, Dorey JB, Bossert S, Qiao H, Orr MC. Big data, big problems? How to circumvent problems in biodiversity mapping and ensure meaningful results. Ecography. 2024; 2024(8): e07115.

21. Grattarola F, Martínez-Lanfranco JA, Botto G, Naya DE, Maneyro R, Mai P, et al. Multiple forms of hotspots of tetrapod biodiversity and the challenges of open-access data scarcity. Scientific Reports. 2020; 10(1): 22045.

22. Perino A, Pereira HM, Felipe-Lucia M, Kim H, Kühl HS, Marselle MR, et al. Biodiversity post-2020: Closing the gap between global targets and national-level implementation. Conservation Letters. 2021; 15(2): e12848.

23. Soberón J. Biodiversity informatics for public policy. The case of CONABIO in Mexico. Biodiversity Informatics. 2022; 17.

24. Berba CMP, Matias AMA. State of biodiversity documentation in the Philippines: Metadata gaps, taxonomic biases, and spatial biases in the DNA barcode data of animal and plant taxa in the context of species occurrence data. PeerJ. 2022; 10: e13146.

25. Meneses CG, Pitogo KME, Supsup CE, Brown RM. Philippine herpetology (Amphibia, Reptilia), 20 years on: two decades of progress towards an increasingly collaborative, equitable, and inclusive approach to the study of the archipelago's amphibians and reptiles. ZooKeys. 2024; 1190, 213–257.

26. Pitogo KME. Gaps and biases in vertebrate wildlife genetics from a global biodiversity hotspot. Environmental Conservation. 2025; 52(3): 127–138.

27. Brown RM, Siler CD, Oliveros CH, Esselstyn JA, Diesmos AC, Hosner PA, et al. Evolutionary processes of diversification in a model island archipelago. Annual Review of Ecology, Evolution, and Systematics. 2013; 44(1): 411–435.

28. Huais PY, Osorio-Olvera L, Cordier JM, Tomba AN, Soberón J, Loyola R, Nori J. Rethinking global hotspots for threatened terrestrial vertebrates. Global Ecology and Biogeography. 2025; 34(1): e13942.

29. Soberón J, Peterson AT. Monitoring biodiversity loss with primary species-occurrence data: toward national-level indicators for the 2010 target of the convention on biological diversity. AMBIO: A Journal of the Human Environment. 2009; 38(1): 29–34.

30. CBD [Convention on Biological Diversity] [cited 01 August 2025]. Decision adopted by the Conference of the Parties to the Convention on Biological Diversity CBD/COP/DEC/15/4 Kunming-Montreal Global Biodiversity Framework. 2022;

CBD/COP/DEC/15/4. Available from: https://www.cbd.int/doc/decisions/cop-15/cop-15-dec-04-en.pdf
31. Orr MC, Hughes AC, Costello MJ, Qiao H. Biodiversity data synthesis is critical for realizing a functional post-2020 framework. Biological Conservation. 2022; 274: 109735.
32. ASEAN Centre for Biodiversity. ASEAN Biodiversity Outlook 3. Philippines, pp. 392; 2023.
33. Maxwell SL, Cazalis V, Dudley N, Hoffmann M, Rodrigues AS, Stolton S, et al. Area-based conservation in the twenty-first century. Nature. 2020; 586(7828): 217–227.
34. Mallari NAD, Collar NJ, McGowan PJ, Marsden SJ. Science-driven management of protected areas: a Philippine case study. Environmental Management. 2013; 51(6): 1236–1246.
35. Jetz W, McGeoch MA, Guralnick R, Ferrier S, Beck J, Costello MJ, et al. Essential biodiversity variables for mapping and monitoring species populations. Nature Ecology & Evolution. 2019; 3(4): 539–551.
36. Hochkirch A, Samways MJ, Gerlach J, Böhm M, Williams P, Cardoso P, et al. A strategy for the next decade to address data deficiency in neglected biodiversity. Conservation Biology. 2021; 35(2): 502–509.
37. Urbano F, Viterbi R, Pedrotti L, Vettorazzo E, Movalli C, Corlatti L. Enhancing biodiversity conservation and monitoring in protected areas through efficient data management. Environmental Monitoring and Assessment. 2024; 196(1): 12.
38. Brown RM, Diesmos AC, Alcala AC. The state of Philippine herpetology and the challenges for the next decade. Silliman Journal. 2001; 42(1): 18–87.

39. Heaney LR. A decade of research on Philippine mammals: progress and challenges. Silliman Journal. 2001; 42(1): 88–107.

40. Pelser PB, Barcelona JF, Nickrent DL . Co's Digital Flora of the Philippines. Available from: www. philippineplants.org.

41. Heaney LR, Balete DS, Rickart EA. The mammals of Luzon Island: biogeography and natural history of a Philippine fauna. JHU Press; 2016.

42. Tanalgo KC, Hughes AC. Bats of the Philippine Islands—A review of research directions and relevance to national-level priorities and targets. Mammalian Biology. 2018; 91(1): 46–56.

43. Allen D. Birds of the Philippines. Lynx and Birdlife International Field Guides, Lynx Edicions, Barcelona, Spain; 2020.

44. Gamalo LE, Sabanal B, Ang A. Three decades of Philippine nonhuman primate studies: research gaps and opportunities for Philippine primatology. Primates. 2021; 62(1): 233–239.

45. delos Angeles MD, Radbouchoom S, Ngarega BK, Lapuz RS, Schneider H. Spatial diversity and distribution of fern and lycophyte species in karst and non-karst landscapes towards conservation needs. Frontiers in Plant Science. 2025; 16: 1495796.

46. Fernandez DAP, Do Linh San E, Forget PM, Veron G. Carnivorans of the Philippines: current knowledge and research gaps. Journal of Mammalogy. 2025; gyaf020.

47. Balisco RAT, Liao TY. An annotated checklist of fish species described from the Philippines. ZooKeys. 2025; 1246: 1–287.

48. Diesmos AC, Watters JL, Huron NA, Davis DR, Alcala AC, Crombie RI, et al. Amphibians of the Philippines, part I: checklist of the species. Proceedings of the California Academy of Sciences. 2015; 62(20): 457–539.

49. Leviton AE, Siler CD, Weinell JL, Brown RM. Synopsis of the snakes of the Philippines. Proceedings of the California Academy of Sciences. 2018; 64(14): 399–568.

50. Fidelino JS, Constantino RRP, Duya MRM. Cross-taxon congruence of terrestrial vertebrates across Philippine Key Biodiversity Areas. Journal for Nature Conservation. 2025; 86: 126907.

51. Mallari NAD, Collar NJ, McGowan PJ, Marsden SJ. Philippine protected areas are not meeting the biodiversity coverage and management effectiveness requirements of Aichi Target 11. Ambio. 2016; 45(3): 313–322.

52. Struebig MJ, Lee JS, Deere NJ, Gevaña DT, Ingram DJ, Lwin N. Drivers and solutions to Southeast Asia's biodiversity crisis. Nature Reviews Biodiversity. 2025;1: 497–514.

53. QGIS Development Team. QGIS Geographic Information System. Open Source Geospatial Foundation Project; 2018. Available from: http://qgis.osgeo.org.

54. Roll U, Feldman A, Novosolov M, Allison A, Bauer AM, Bernard R, et al. The global distribution of tetrapods reveals a need for targeted reptile conservation. Nature Ecology & Evolution. 2017; 1(11): 1677–1682.

55. Caetano GHDO, Chapple DG, Grenyer R, Raz T, Rosenblatt J, Tingley R, et al. Automated assessment reveals that the extinction risk of reptiles is widely underestimated across space and phylogeny. PLoS Biology. 2022; 20(5): e3001544.

56. Brown RM, Siler CD, Richards SJ, Diesmos AC, Cannatella DC. Multilocus phylogeny and a new classification for Southeast Asian and Melanesian forest frogs (family Ceratobatrachidae). Zoological Journal of the Linnean Society. 2015; 174(1): 130–168.

57. Sanguila MB, Cobb KA, Siler CD, Diesmos AC, Alcala AC, Brown RM. The amphibians and reptiles of Mindanao Island, southern Philippines, II: the herpetofauna of northeast Mindanao and adjacent islands. ZooKeys. 2016; (624), 1–132.

58. Pitogo KME, Saavedra AJL, Afuang LE. Amphibians and reptiles of Mount Busa, Sarangani Province: a glimpse of the herpetological community of southern Mindanao, Philippines. Philippine Journal of Science. 2021; 150(5): 1279–1306.

59. Nuñez-Penichet C, Cobos ME, Soberón J, Gueta T, Barve N, Barve V, Navarro-Sigüenza AG, Peterson AT. Selection of sampling sites for biodiversity inventory: Effects of environmental and geographical considerations. Methods in Ecology and Evolution. 2022; 00:1–13.

60. Ong PS, Afuang LE, Rosell-Ambal RG. Philippine Biodiversity Conservation Priorities: A second iteration of the National Biodiversity Strategy and Action Plan. Department of Environment and Natural Resources-Protected Areas and Wildlife Bureau, Conservation International Philippines, Biodiversity Conservation Program-University of the Philippines Center for Integrative and Development Studies, and Foundation for the Philippine Environment. Quezon City, Philippines; 2002.

61. R Core Team (2022). R: A language and environment for statistical computing. R Foundation for Statistical Computing, Vienna, Austria. Available from: https://www.R-project.org/.

62. Hijmans R._terra: Spatial Data Analysis_. R package version 1.7-78; 2025. Available from: https://CRAN.R-project.org/package=terra.

63. Ricklefs RE, Lovette IJ. The roles of island area per se and habitat diversity in the species–area relationships of four Lesser Antillean faunal groups. Journal of Animal Ecology. 1999; 68(6): 1142–1160.

64. Gotelli NJ. A Primer of Ecology, 4th ed. Sunderland, MA: Sinauer Associates; 2008.

65. Venables WN, Ripley BD. Modern Applied Statistics with S. Fourth Edition. Springer, New York. ISBN 0-387-95457-0; 2002.

66. Brooks ME, Kristensen K, Van Benthem KJ, Magnusson A, Berg CW, Nielsen A, et al. glmmTMB balances speed and flexibility among packages for zero-inflated generalized linear mixed modeling. The R Journal. 2017; 9(2): 378–400.

67. Zuur AF, Ieno EN, Walker N, Saveliev AA, Smith GM, Zuur AF, et al. GLM and GAM for count data. Mixed effects models and extensions in ecology with R, 209–243; 2009.

68. Hartig F. _DHARMa: Residual Diagnostics for Hierarchical (Multi-Level / Mixed) Regression Models_. R package version 0.4.7; 2024. Available from: https://CRAN.R-project.org/package=DHARMa.

69. Lüdecke D, Ben-Shachar MS, Patil I, Waggoner P, Makowski D. performance: An R Package for Assessment, Comparison and Testing of Statistical Models. Journal of Open Source Software. 2021; 6(60): 3139.

70. H. Wickham. ggplot2: Elegant Graphics for Data Analysis. Springer-Verlag New York; 2016.

71. Nuñeza OM, Ates FB, Alicante AA. Distribution of endemic and threatened herpetofauna in Mt. Malindang, Mindanao, Philippines. Biodiversity and Conservation. 2010; 19, 503–518.

72. Gonzalez JCT, de Guia APO, Dimalibot JC, Pantua KV, Gustilo WO, Bantayan NC. Understorey to canopy vertebrate fauna of a lowland evergreen forest in Mt. Makiling Forest Reserve, Philippines. Biodiversity Data Journal. 72; 8: e56999.

73. Brown WC, Alcala AC. Populations of amphibians and reptiles in the submontane and montane forests of Cuernos de Negros, Philippine Islands. Ecology. 1961; 628–636.

74. Brown WC, Alcala AC. The zoogeography of the Philippine Islands, a fringing archipelago. Proceedings of the California Academy of Sciences. 1970; 38: 105–130.

75. Brown RM, McGuire JA, Ferner JW, Icarangal Jr N, Kennedy RS. Amphibians and reptiles of Luzon Island, II: preliminary report on the herpetofauna of Aurora Memorial National Park, Philippines. Hamadryad. 2000; 25:175–195

76. Siler CD, Welton LJ, Siler JM, Brown J, Bucol A, Diesmos AC, Brown RM. Amphibians and Reptiles, Luzon Island, Aurora Province and Aurora Memorial National Park, Northern Philippines: New island distribution records. Check List. 2011; 7(2): 182–195.

77. Brown RM. A new species of stream frog of the genus *Hylarana* from the mountains of southern Mindanao Island, Philippines. Herpetologica. 2015; 71(3): 223–233.

78. Siler CD, Welton LJ, Diesmos AC, Siler JM, Bucol A, Brown J, Brown RM. Amphibians and reptiles, Romblon Island Group, central Philippines: comprehensive herpetofaunal inventory. Check List. 2012; 8(3): 443–462.

79. Meneses CG, Siler CD, Alviola PA, Gonzalez JCT, Balatibat JB, Natividad CA, Brown RM. Amphibian and reptile diversity along a ridge-to-reef elevational gradient on a small isolated oceanic island of the central Philippines. Check List. 2012; 18(5): 941–948.

80. Plaza JL, Sanguila MB. Preliminary report on the anurans of Mount Hilong-hilong, Agusan del Norte, eastern Mindanao, Philippines. Asian Herpetological Research. 2015; 6(1): 18–33.

81. Gojo-Cruz PHP, Afuang LE, Gonzalez JCT, Gruezo WS. Amphibians and reptiles of Luzon Island, Philippines: the herpetofauna of Pantabangan-Carranglan watershed, Nueva Ecija Province, Caraballo Mountain range. Asian Herpetological Research. 2018; 9(4): 201–223.

82. Supsup CE, Asis AA, Carestia Jr UV, Diesmos AC, Mallari NAD, Brown RM. Variation in species richness, composition and herpetological community structure across a tropical habitat gradient of Palawan Island, Philippines. Herpetozoa. 2020; 33: 95–111.

83. Azovsky AI. Species–area and species–sampling effort relationships: disentangling the effects. Ecography. 2011; 34(1): 18–30.

84. Pitogo KME, Meneses CG, Flores ABA, Saavedra AJL, Amarga AKS, Angeles MD, et al. Caught in statistical noise: pitfalls of a unidimensional approach to understanding biodiversity-conflict relationships. npj Biodiversity. 2025; 4(1): 17.

85. Brown RM, Meneses CG, Wood Jr PL, Fernandez JB, Cuesta MA, Clores MA, et al. Unexpected discovery of another new species of Philippine false gecko (Gekkonidae; *Pseudogekko*) from the Bicol Peninsula of Luzon Island. Herpetologica. 2020; 76(3): 315–329.

86. Di Minin E, Toivonen T. Global protected area expansion: creating more than paper parks. BioScience. 2015; 65(7): 637–638.

87. Watson JE, Dudley N, Segan DB, Hockings M. The performance and potential of protected areas. Nature. 2014; 515(7525): 67–73.

88. Jonas HD, Ahmadia GN, Bingham HC, Briggs J, Butchart SHM, Carino J, Chassot O, Chaudhary S, et al. Equitable and effective areas-based conservation: Towards the conserved area paradigm. *PARKS*. 2021; 21: 71–84.

89. Eken G, Bennun L, Brooks TM, Darwall W, Fishpool LD, Foster M, et al. Key biodiversity areas as site conservation targets. BioScience. 2004; 54(12): 1110–1118.

90. Hoffmann S. Challenges and opportunities of area-based conservation in reaching biodiversity and sustainability goals. Biodiversity and Conservation. 2022; 31(2): 325–352.

91. Tenorio EA, Montoya P, Norden N, Rodríguez-Buriticá S, Salgado-Negret B, Gonzalez MA. Mountains exhibit a stronger latitudinal diversity gradient than lowland regions. Journal of Biogeography. 2023; 50(6): 1026–1036.

92. Pitogo KME, Saavedra AJ. Conservation assessment of the recently rediscovered Guttman's Stream Frog, *Pulchrana guttmani* (Brown, 2015), reveals the need for its uplisting into a higher threat category. Herpetology Notes. 2023; 16: 717–721.

93. Heaney LR. Conservation biogeography in oceanic archipelagoes. Frontiers of Biogeography, New Directions in the Geography of Nature. Sinauer Associates, Sunderland, MA, 345–360; 2004.

94. McGuire JA, Alcala AC. A taxonomic revision of the flying lizards (Iguania: Agamidae: *Draco*) of the Philippine Islands, with a description of a new species. Herpetological Monographs. 2000; 14: 81–138.

95. Allen D, Oliveros C, Española C, Broad G, Gonzalez JCT. A new species of *Galliralus* from Calayan Island, Philippines. Forktail. 2004; 20: 1–7.

96. Heaney LR, Tabaranza BR, Balete DS, Rigertas N. Synopsis and biogeography of the mammals of Camiguin Island, Philippines. Fieldiana Zoology. 2006; 2006(106): 28–48.

97. Brown RM, Siler CD, Oliveros CH, Diesmos AC, Alcala AC. A new *Gekko* from Sibuyan Island, central Philippines. Herpetologica. 2011; 67(4), 460–476.

98. Oliveros CH, Ota H, Crombie RI, Brown RM. The herpetofauna of the Babuyan group of islands, northern Philippines. Scientific Publications of the Natural History Museum of the University of Kansas. 2011; 43: 1–20.

99. Reyes MC, Ingicco T, Piper PJ, AmanoN, Pawlik AF. First fossil evidence of the extinct Philippine cloud rat *Crateromys paulus* (Muridae: Murinae: Phloeomyini) from Ilin Island, Mindoro, and insights into its Holocene abundance. Proceedings of the Biological Society of Washington. 2017; 130(1): 84–97.

100. Barley AJ, Diesmos AC, Siler CD, Martinez CM, Brown RM. Taxonomic revision of Philippine Sun Skinks (Reptilia: Squamata: Scincidae: *Eutropis*), and descriptions of eight new species. Herpetological Monographs. 2020; 34(1): 39–70.

101. Clores MA, Bautista JB, Fernandez JB, Cuesta MA, Brown RM. Diversity and distribution of amphibians and reptiles in the Caramoan Island Group, Maqueda Channel, southern Luzon, Philippines. Journal of Asia-Pacific Biodiversity. 2021; 14(1): 1–14.

102. Supsup CE, Asis AA, Carestia UV, Puna NM, Avanceña JB. Geographic distribution, population and conservation status of *Tragulus nigricans*, an endangered mouse deer endemic to western Philippines. Pacific Conservation Biology. 2021; 28(1): 90–96.

103. Russell JC, Kueffer C. Island biodiversity in the Anthropocene. Annual Review of Environment and Resources. 2019; 44(1): 31–60.

104. Borges PA, Cardoso P, Gabriel R, Ah-Peng C, Emerson BC. Challenges, advances and perspectives in Island Biogeography. Frontiers of Biogeography. 2016; 8(2).

105. Blades B, Ronquillo C, Hortal J. Mobilisation of data from natural history collections can increase the quality and coverage of biodiversity information. Ecology and Evolution. 2025; 15(4): e71139.

106. Heberling JM, Miller JT, Noesgaard D, Weingart SB, Schigel D. Data integration enables global biodiversity synthesis. Proceedings of the National Academy of Sciences. 2021; 118(6): e2018093118.

107. Amano T, Lamming JD, Sutherland WJ. Spatial gaps in global biodiversity information and the role of citizen science. Bioscience. 2016; 66(5): 393–400.

108. Della Rocca F, Musiani M, Galaverni M, Milanesi P. Improving online citizen science platforms for biodiversity monitoring. Journal of Biogeography. 108; 51(12): 2412–2423.

109. Mason BM, Mesaglio T, Barratt Heitmann J, Chandler M, Chowdhury S, Gorta SBZ, et al. iNaturalist accelerates biodiversity research. BioScience. 2025; biaf104.

110. Barve V. Discovering and developing primary biodiversity data from social networking sites: A novel approach. Ecological Informatics. 2014; 24: 194–199.

111.Chowdhury S, Aich U, Rokonuzzaman M, Alam S, Das P, Siddika A, et al. Increasing biodiversity knowledge through social media: A case study from tropical Bangladesh. BioScience. 2023; 73(6): 453–459.

112. Tabeta MSG, Bejar SGF. Improving our understanding of a cryptic primate, the Philippine Tarsier (*Carlito syrichta*), through social media. International Journal of Primatology. 2025; 1–26.

113. Madera BS. Population survey of marine turtles in Boracay Island, Philippines using a citizen science approach. Journal of Management and Development Studies. 2019; 8(2): 1–15.

114. Acuña D, Isagani N, Pitogo KME. Predation on a McClung's Philippine false coralsnake, *Hemibungarus mcclungi* (Weigmann 1835), by a giant spiny centipede, *Scolopendra spinosissima* Kraepelin 1903, on Luzon Island, The Philippines. Reptiles & Amphibians. 2021; 28(3): 417–419.

115. Sullivan BL, Aycrigg JL, Barry JH, Bonney RE, Bruns N, Cooper CB, et al. The eBird enterprise: An integrated approach to development and application of citizen science. Biological Conservation. 2014; 169: 31–40.

116. Pitogo KME, Jarvis F, Saavedra AJL, Senarillos TL, Concepcion CB, Simpson PM. Avian fauna of Mount Melibengoy, southern Mindanao, Philippines: conservation implications on a partially protected key biodiversity area under threat. Philippine Journal of Systematic Biology Online ISSN. 2024; 17: 29–40.

117. Barcelona JF, Nickrent DL, LaFrankie JV, Callado JRC, Pelser PB. Co's digital flora of the Philippines: Plant identification and conservation through cybertaxonomy. Philippine Journal of Science. 2013; 142: 57–67.

118.Pernat N, Canavan S, Golivets M, Hillaert J, Itescu Y, Jarić I, et al. Overcoming biodiversity blindness: Secondary data in primary citizen science observations. Ecological Solutions and Evidence. 2024; 5(1): e12295.

119. Binaday JWB, Amarga AKS, Barrameda ES, Bonagua BJM. Amphibians and reptiles in the vicinity of Bulusan lake, Bulusan Volcano natural park, Sorsogon, Philippines. Philippine Journal of Science. 2017; 146(3): 339–351.

120. Decena SCP, Macasait Jr DR, Arguelles MS. Species Accounts, assemblage, and microhabitats of amphibians and reptiles of Northeastern Leyte, Philippines. Philippine Journal of Science. 2023; 152(1).

121. Maglangit EPT, Nuñeza OM, Coritico FP, Medecilo-Guiang MMP, Mohagan AB, Patano Jr RR, Amoroso VB. Richness and distribution of reptiles and amphibians in the tropical lowland habitats of Mt. Agad-Agad, Iligan City, Southern Philippines. Reptiles & Amphibians. 2022; 29(1): 413–425.

122. Beck J, Ballesteros-Mejia L, Nagel P, Kitching IJ. Online solutions and the ‘Wallacean shortfall’: what does GBIF contribute to our knowledge of species' ranges?. Diversity and Distributions. 2013; 19(8): 1043–1050.

123. Cadotte MW, Beyene MS, Bowell M, Dalrymple SE, Dechoum MS, Dooner P, et al. Grey matters: Ensuring management information is a part of the permanent evidence base by creating open grey literature principles. Ecological Solutions and Evidence. 2025; 6(2).

124. Wieczorek J, Bloom D, Guralnick R, Blum S, Döring M, Giovanni R, et al. Darwin Core: an evolving community-developed biodiversity data standard. PloS one. 2012; 7(1): e29715.

125. Ball-Damerow JE, Brenskelle L, Barve N, Soltis PS, Sierwald P, Bieler R, et al. Research applications of primary biodiversity databases in the digital age. PloS one. 2019; 14(9): e0215794.

126. Marques N, de Melo Soares CD, de Melo Casali D, Guimarães EC, Fava FG, da Silva Abreu JM, et al. Retrieving biodiversity data from multiple sources: making secondary data standardised and accessible. Biodiversity Data Journal. 2024; 12: e133775.

127. Costello MJ, Wieczorek J. Best practice for biodiversity data management and publication. Biological Conservation. 2014; 173: 68–73.

128. Robinson JG, LaBruna D, O'Brien T, Clyne PJ, Dudley N, Andelman SJ, et al. Scaling up area-based conservation to implement the Global Biodiversity Framework's 30x30 target: The role of Nature's Strongholds. PLoS Biology. 2024; 22(5): e3002613.

129. Buckland ST, Johnston A. Monitoring the biodiversity of regions: Key principles and possible pitfalls. Biological Conservation. 2917; 214: 23–34.

130. Wenk E, Mesaglio T, Keith D, Cornwell W. Curating protected area-level species lists in an era of diverse and dynamic data sources. Ecological Informatics. 2024; 84: 102921.

131. Britz R, Hundsdörfer A, Fritz U. Funding, training, permits – the three big challenges of taxonomy. Megataxa. 2020; 1(1): 49–52.

132. Dawson NM, Coolsaet B, Bhardwaj A, Booker F, Brown D, Lliso B, et al. Is it just conservation? A typology of Indigenous peoples' and local communities' roles in conserving biodiversity. One Earth. 2024; 7(6): 1007–1021.

133. Abrahamse T, Andrade-Correa MG, Arida C, Galsim R, Häuser C, Price M, Sommerwerk N. The Global Taxonomy Initiative in Support of the Post-2020 Global

Biodiversity Framework. CBD Technical Series No. 96. Secretariat of the Convention on Biological Diversity, Montreal, 103 pages; 2021.

134. Soberón J, Peterson AT. Biodiversity informatics: managing and applying primary biodiversity data. Philosophical Transactions of the Royal Society of London. Series B: Biological Sciences. 2004; 359(1444): 689–698.

135. Güntsch A, Overmann J, Ebert B, Bonn A, Le Bras Y, Engel T, et al. National biodiversity data infrastructures: ten essential functions for science, policy, and practice. BioScience. 2025; 75(2): 139–151.

136. Colella JP, Silvestri L, Súzan G, Weksler M, Cook JA, Lessa EP. Engaging with the Nagoya Protocol on Access and Benefit-Sharing: recommendations for noncommercial biodiversity researchers. Journal of Mammalogy. 2023; 104(3): 430–443.

137. DENR-BMB [Department of Environment and Natural Resources – Biodiversity Management Bureau]. Philippine Biodiversity Strategy and Action Plan (Abridged). Department of Environment and Natural Resources – Biodiversity Management Bureau, Quezon City, Metro Manila, 26 pp; 2025.

138. Schmeller DS, Böhm M, Arvanitidis C, Barber-Meyer S, Brummitt N, Chandler M, et al. Building capacity in biodiversity monitoring at the global scale. Biodiversity and Conservation. 2017; 26: 2765–2790.Carroll, S. R., Herczog, E., Hudson, M., Russell, K., & Stall, S. (2021). Operationalizing the CARE and FAIR Principles for Indigenous data futures. *Scientific Data*, 8(1): 108.

139. Carroll SR, Herczog E, Hudson M, Russell K, Stall S. Operationalizing the CARE and FAIR Principles for Indigenous data futures. Scientific Data. 2021; 8(1): 108.

Supporting Information

S1 Fig. Model diagnostic results from DHARMa for the best-fit species diversity model for conserved areas (Species Diversity ~ log_Area + log_Occurrence + sqrt_Topo_Relief + log_Area:sqrt_Topo_Relief). Upper-right panel shows a Q–Q plot used to detect deviations from the expected distribution, along with tests for distribution (Kolmogorov–Smirnov), dispersion, and outliers. Additional panels show scaled residuals plotted against predicted values and each predictor. Simulation outliers, data points falling outside the range of simulated values, are highlighted as red stars. Red line indicates statistically significant deviations from model expectations. DHARMa zero-inflation test is also included.

S2 Fig. Model diagnostic results from DHARMa for the best-fit species diversity model for key biodiversity areas (Species Diversity ~ log_Area + log_Occurrence +

sqrt_Topo_Relief + log_Area:log_Occurrence + log_Area:sqrt_Topo_Relief + log_Occurrence:sqrt_Topo_Relief). Upper-right panel shows a Q–Q plot used to detect deviations from the expected distribution, along with tests for distribution (Kolmogorov–Smirnov), dispersion, and outliers. Additional panels show scaled residuals plotted against predicted values and each predictor. Simulation outliers, data points falling outside the range of simulated values, are highlighted as red stars. Red line indicates statistically significant deviations from model expectations. DHARMa zero-inflation test is also included.

S3 Fig. Model diagnostic results from DHARMa for the best-fit sampling effort model for conserved areas (No. of Occurrence Records ~ log_Area × sqrt_Topo_Relief + PAIC). Upper-right panel shows a Q–Q plot used to detect deviations from the expected distribution, along with tests for distribution (Kolmogorov–Smirnov), dispersion, and outliers. Additional panels show scaled residuals plotted against predicted values and each predictor. Simulation outliers, data points falling outside the range of simulated values, are highlighted as red stars. Red line indicates statistically significant deviations from model expectations. DHARMa zero-inflation test is also included.

S4 Fig. Model diagnostic results from DHARMa for the best-fit sampling effort model for key biodiversity areas (No. of Occurrence Records ~ log_Area × sqrt_Topo_Relief + PAIC). Upper-right panel shows a Q–Q plot used to detect deviations from the expected distribution, along with tests for distribution (Kolmogorov–Smirnov), dispersion, and outliers. Additional panels show scaled residuals plotted against predicted values and each predictor. Simulation outliers, data points falling outside the range of simulated values, are

highlighted as red stars. Red line indicates statistically significant deviations from model expectations. DHARMa zero-inflation test is also included.

S5 Fig. Map of the Philippines showing (A) overlap between forest cover, priority conservation areas (PCAs), and key biodiversity areas (KBAs), and (B) distribution of observed amphibian and squamate reptile diversity across all data types outside PCAs and KBAs, highlighting that many well-sampled sites with higher observed diversity lie beyond established conservation areas.

S1 Table. Stepwise model selection using AIC for species richness as a function of area, occurrence density, and topographic relief (species diversity model for conserved areas). The starting model included all main effects and their three-way interaction. The final model retained only two main effects and one two-way interaction.

S2 Table. Stepwise model selection using AIC for species richness as a function of area, occurrence density, and topographic relief (species diversity model for key biodiversity areas). The starting model included all main effects and their three-way interaction. The final model retained all main effects and two-way interactions, excluding the three-way interaction.

S3 Table. Stepwise model selection using AIC for occurrence count as a function of area, topographic relief, and biogeographic subregion or PAIC (sampling effort model

for conserved areas). The starting model included a two-way interaction between area and relief, and an additive effect of PAIC level.

S4 Table. Stepwise model selection using AIC for occurrence count as a function of area, topographic relief, and biogeographic subregion or PAIC (sampling effort model for key biodiversity areas). The starting model included a two-way interaction between area and relief, and an additive effect of PAIC level.

PONE-D-25-55790
Response to Reviewers

March 20, 2026

Juan C. Diaz-Ricaurte
Academic Editor, PLOS One

Dear Dr. Diaz-Ricaurte,

Thank you for the opportunity to submit a revised version of our manuscript, "*Sampling bias obscures biodiversity patterns, reveals data gaps in a megadiverse island archipelago hotspot*," for consideration in PLOS ONE. On behalf of my co-authors, I sincerely thank you and the reviewers for your thoughtful and constructive comments. These suggestions have substantially improved the clarity, coherence, and scientific rigor of the manuscript.

In the revised version, we have modified the title by replacing "priority conservation areas" with "megadiverse island archipelago hotspot" to ensure consistency with terminology used throughout the manuscript, as further explained in our responses below. We also removed "*a call for improved documentation*" to shorten title. We provide detailed, point-by-point responses to all comments following this letter for your review and consideration.

Sincerely,

Kier Mitchel E. Pitogo

EDITOR

Editor: A first overarching issue, raised primarily by Reviewer 1, concerns the alignment between research questions and analytical choices. Although the research questions are clearly stated, the links between each question, the statistical models used, and the corresponding results are not always explicit. I strongly suggest that the revised manuscript make these connections clear and systematic. Each main question should be directly associated with the analysis used to address it and with the relevant results, as this alignment is essential for transparency and interpretability.

Response: We have revised the phrasing of Questions 3 and 4 to better align with the analyses conducted. In the Methods section, we now state each specific objective before describing the corresponding analytical approach to clarify the connection between the questions and the analyses. The Results section also follow the same structure.

Editor: A major methodological concern, discussed in detail by Reviewer 1 and reinforced by Reviewer 2, relates to the framework used to compare different categories of conservation areas. Much of the manuscript's inference depends on comparing sampling effort and diversity patterns across conservation area types, yet the current analytical approach does not allow formal statistical testing of differences among these categories. I agree with both reviewers that this represents a substantive limitation. For the revision to be satisfactory, these comparisons should

be conducted within a unified modeling framework that allows direct statistical evaluation of differences in effects across conservation area types.

Response: We reanalyzed the dataset using a joint modeling framework in which species richness was modeled as a function of area, topographic relief, and occurrence density, including all pairwise interactions among these predictors and their interactions with PCA type (protected and conserved areas vs. key biodiversity areas). Biogeographic subregion was included as a random intercept to account for non-independence among PCAs due to shared biogeographic structure. Results were broadly consistent with our previous approach. However, we retain this unified model as it more directly addresses our specific questions, as recommended by the reviewers as well.

Editor: Reviewer 1 emphasizes the importance of retaining model components that are justified by the study design, particularly grouping or hierarchical variables included to account for non-independence among observations.

Response: In our new models, we retained the biogeographic subregion (Pleistocene Aggregate Island Complexes or PAIC) as a random effect. Variance explained for the random effect are also reported in the results section, alongside the coefficient estimates of the fixed predictors.

Editor: The spatial properties of the dataset constitute another key issue raised by both reviewers from complementary perspectives. While reviewer 1 focuses on spatial precision, including how coordinate uncertainty was handled and whether the chosen grid resolution is appropriate given the characteristics of the occurrence records, the reviewer 2 emphasizes the need to explicitly address spatial dependence among observations. Given the spatially explicit nature of the analyses, I agree that these issues cannot be treated as secondary. The revised manuscript should clearly justify the analytical scale, address positional uncertainty, and either incorporate spatial structure into the models or provide a clear and defensible rationale for not doing so.

Response: We thank both Reviewers for highlighting the inherent challenges of spatially explicit analyses. In response to Reviewer 1, we have clarified in the revised manuscript our rationale for the selected 10-km grid resolution (**lines 200–204**) and our treatment of coordinate uncertainty (**lines 184–190**). The choice of a 10-km grid resolution was deliberate and intended to provide an appropriate balance for visualizing broad-scale spatial patterns of observed species richness patterns across the Philippines.

Regarding spatial uncertainty, we retained coordinates that were consistent with general (provincial-level) locality information, as these adequately reflect broad geographic distribution patterns (country-wide assessment). Our sampling is broad enough and has a level of certainty where broad patterns will be discovered. Although minor spatial imprecision may influence species richness estimates within individual PCAs used in the modeling, any resulting bias is expected to be minimal because most PCAs are supported by numerous occurrence records, species are represented by multiple points within PCAs due to the integration of multiple data sources, and the overall sample size is large (n = 316 PCAs).

Editor: Both reviewers also identify limitations in the biological interpretation of the results, particularly regarding the joint treatment of amphibians and reptiles. These groups differ in ecology, detectability, sampling intensity, and conservation context. I suggest that the authors either explicitly assess whether these taxa exhibit similar or contrasting patterns, or clearly justify

their combined analysis. Without this clarification, the ecological interpretation of the results remains incomplete.

Response: We acknowledge that amphibians and reptiles differ in their ecology and diversity patterns. However, as stated in the Introduction, these groups were used in this study because, relative to other taxonomic groups in the Philippines, they are comparatively well-documented and thus provide a robust basis for assessing observed biodiversity patterns and sampling distribution across areas. Our objective was not to model amphibian and reptile diversity separately or to compare their biological drivers (e.g., fine-scale environmental variables), but rather to evaluate how observed species richness patterns relate to area, sampling effort, and topographic relief in the context of biodiversity documentation or conservation planning. Accordingly, our interpretation focuses on spatial patterns of documented diversity rather than taxon-specific ecological differences. In addition, amphibians and reptiles have historically and contemporarily been surveyed and analyzed jointly in the Philippines (see Meneses et al. 2024), making their combined treatment, at least within the context of this paper, consistent with established research practice.

Editor: Further clarification is also required regarding the conceptual framing of conservation areas. Reviewer 2 raises questions about how different conservation categories are defined and compared, while Reviewer 1 highlights the difficulty of interpreting area-based comparisons under strong and uneven sampling biases. I agree with both assessments and recommend that the revised manuscript explicitly state the assumptions underlying these comparisons and clearly articulate how results should be interpreted in light of known data limitations.

Response: We recognize that the conceptual framing and terminology of conservation areas required consistency in the original submission. Following the recommendation of the Reviewer, we adopted the lexicon proposed by Salafsky et al. (2024), distinguishing between protected and conserved areas (i.e., areas with formal management or governance) and key biodiversity areas (i.e., spatial prioritization tools without inherent governance or protection status). In the revised manuscript, we refer to both collectively as Philippine conservation-relevant areas (PCAs) to clarify the scope of our comparisons and to avoid confusion with the previous use of “priority conservation areas.” We limit acronym usage to PCA, while “protected and conserved areas” and “key biodiversity areas” are written out in full throughout the text for clarity and consistency.

Regarding concerns about interpretation under uneven sampling effort, we model observed species richness as a function of area, topographic relief, and occurrence density (as a proxy for sampling effort), with biogeographic subregion included as a random intercept to account for geographic clustering. This framework allows us to assess whether differences in observed richness among PCA types persist after accounting for variation in sampling intensity (and other predictors) and shared biogeographic structure. In addition, we modeled occurrence points as a function of area and topographic relief, again including biogeographic subregion as a random effect, to evaluate whether sampling effort itself varies systematically between PCA types (which we found no sufficient evidence). Finally, we clarify in the Discussion that our results reflect documented (observed) diversity patterns and should be interpreted in light of known data limitations.

Editor: Both reviewers note that several figures (particularly those illustrating spatial patterns) are difficult to interpret due to low resolution and small text. This may be related to how figures

are displayed in the submission system; however, I recommend you include all figures in the revised version at the highest resolution possible to facilitate careful evaluation by the reviewers.

Response: We will upload the highest-resolution version permitted by the submission system. The original figures were generated at high resolution; however, the conversion to PDF within the system may have reduced their clarity.

REVIEWER 1

Reviewer: I would suggest that the authors more explicitly connect each research question to the specific model(s) used. Clarifying which models and results correspond to which of the four main questions would enhance the transparency and coherence of the study design.

Response: We thank the Reviewer for raising this clarification. In the revised methods section, we now state each specific objective before describing the corresponding analytical approach to clarify the connection between the questions and the analyses. The Results section also follow the same structure.

Reviewer: Thank you for the detailed description of the data curation procedures. All the steps were important to guarantee the quality of the input data. However, I would like to raise a question regarding the spatial precision of the occurrence records used.

While the manuscript mentions the exclusion of records outside known distributions and the removal of generic coordinates, I did not find any reference to the use of the "coordinate uncertainty in meters" field (available in GBIF records) or a similar assessment of the dataset's spatial accuracy. Given that the study employs a 10-km grid resolution, I wonder whether this scale is appropriate considering the potential variability in coordinate precision, especially for historical museum records which can lack fine-scale georeferencing.

Could the authors clarify whether any filtering based on coordinate uncertainty was applied? If not, do the authors consider the 10-km resolution robust to such imprecision, and how might this potentially affect the spatial allocation of records and the resulting diversity patterns?

Response: We thank the reviewer for highlighting the issue of spatial precision. Because relatively few occurrence records include explicit uncertainty, we did not filter based on this metric. Instead, we retained records with geographic coordinates that correspond to general geographic location (provincial level). All records broadly reflect geographic patterns of each species distribution, and any minor imprecision is expected to have minimal impact on broad-scale, country-wide patterns. As stated above, although minor spatial imprecision may influence species richness estimates within individual PCAs used in the modeling, any resulting bias is expected to be minimal because most PCAs are supported by numerous occurrence records, species are represented by multiple points within PCAs due to the integration of multiple data sources, and the overall sample size is large (n = 316 PCAs).

The 10-km grid resolution was also deliberately chosen to visualize broad-scale distribution patterns across the Philippines. This scale provides an appropriate balance: smaller grids (e.g., <10 km) would be too fragmented on larger islands, while larger grids (e.g., 15–20 km) could average out meaningful spatial variation, particularly on smaller islands. We have included this justification in the revised manuscript (**Lines 200–204**).

Reviewer: The current analysis utilizes Negative Binomial GLMs fitted separately for Protected Areas (PAs) and Key Biodiversity Areas (KBAs). The methodological rationale for this decision is not fully clear in the manuscript. If this separation was a deliberate choice to address, for example, severe variance heterogeneity, it would be very helpful for the reader if this justification were made explicit.

My main concern with this approach is that a central objective of the study appears to be the comparison of predictor effects (e.g., area, topographic relief), and the comparison of richness and effort between PAs and KBAs. Adjusting separate models, while legitimate for describing the relationships within each group, impedes a formal statistical comparison of the coefficient estimates.

To formally test the hypothesis that a predictor's effect differs between PAs and KBAs, the standard and more robust statistical approach would be to analyze both groups within a single, unified model. This is typically achieved by including the group affiliation (PA vs. KBA) as a categorical predictor and, crucially, testing interaction terms between this group variable and the predictors of interest (e.g., PCA_Type * Area). The p-value for the interaction term provides the formal statistical test of the null hypothesis that there is no difference in the slopes between the groups.

It is plausible that adopting this unified model approach would yield substantive results similar to those already presented. However, its key advantage would be to make the comparative claims—which are central to the study's objectives—statistically explicit and defensible, thereby increasing the analysis's robustness and interpretive clarity.

Response: We thank the Reviewer for this thoughtful and constructive feedback. In the revised manuscript, we reanalyzed the dataset using a joint modeling framework in which species richness was modeled as a function of area, topographic relief, and occurrence density, including all pairwise interactions among these predictors and their interactions with PCA type. Biogeographic subregion was included as a random intercept to account for non-independence among PCAs due to shared biogeographic structure. Although the results are broadly consistent with our previous approach, as the Reviewer noted, this framework allows for a more direct comparison between PCA types and highlights additional patterns for discussion. Hence, we kept this unified modelling framework. We have revised the Methods (**lines 228–317**), Results (**lines 401–465**), and Discussion (**lines 522–578**) sections accordingly.

Reviewer: My final methodological point relates to the model selection for control effects. The authors state that the random effect variable "PAIC" was removed from one of the models because it "explained less than 1% of the variance and did not improve model performance." (Line 234).

A low number of levels (seven, in this case) can lead to imprecise variance estimates. However, the decision to include or exclude a structural random effect should be based on the study design, not on its statistical performance (such as variance explained or AIC).

As the 'PAIC' variable represents a grouping variable (e.g., island/region) dictated by the sampling design, its function in the model is not primarily to "explain variance" (as in a predictive model) , but rather to control for the non-independence of the data (i.e., pseudoreplication). Observations from the same PAIC group are likely to be more similar to each

other, violating the assumption of independence. The literature strongly advocates for retaining the random effects structure justified by the design. Removing this structural term, even if its variance is low, can lead to "overoptimistic" results, such as artificially reduced standard errors and an inflated Type I error rate. (Barr, D. J., Levy, R., Scheepers, C., & Tily, H. J. (2013). Random effects structure for confirmatory hypothesis testing: Keep it maximal. Journal of Memory and Language, 68(3), 255-278. https://doi.org/10.1016/j.jml.2012.11.001)

I would, therefore, recommend that the authors retain the 'PAIC' random effect in the final models to ensure that potential non-independence in the data is appropriately controlled for, thereby strengthening the validity of the model's inferences.

Response: We thank the Reviewer for this critical point. In our new models, we retained the biogeographic subregion (Pleistocene Aggregate Island Complexes or PAIC) as a random effect. Variance explained for the random effect are also reported in the results section, alongside the coefficient estimates of the fixed predictors.

Reviewer: Regarding the presentation of these results, I found many of the figures, both in the supplementary material and - more significantly - in the main manuscript, difficult to interpret: the resolution appears to be low, and the font size for labels and legends is very small. I would recommend the authors provide higher-resolution figures with more legible text to ensure readers can fully interpret the study's important spatial results.

Response: We will upload the highest-resolution version permitted by the submission system. The original figures were generated at high resolution; however, the conversion to PDF within the system may have reduced their clarity.

REVIEWER 2

Our responses address the specific comments provided in the annotated manuscript, which correspond closely to the summarized remarks. Accordingly, we have responded directly to those in-text comments.

Reviewer: Lines 84–85: You are mixing terms and categories. Key Biodiversity Areas (KBAs) are referred to in the manuscript as Priority Conservation Areas (PCAs), yet the models and figures still use the term “KBAs.” This inconsistency creates confusion for the reader.

Furthermore, it is not clear which criteria were used to spatially define the Priority Conservation Areas. Based on the description, it appears that these areas correspond to the officially recognized KBAs in the Philippines. If that is the case, then the PCAs in your study are essentially KBAs, and this should be explicitly stated to avoid misinterpretation.

Response: We thank the Reviewer for raising this point. We recognize that the conceptual framing and terminology of conservation areas required greater precision or consistency in the original submission. Following the recommendation, we adopted the lexicon proposed by Salafsky et al. (2024), distinguishing between protected and conserved areas (i.e., areas with formal management or governance) and key biodiversity areas (i.e., spatial prioritization tools without inherent governance or protection status). In the revised manuscript, we refer to both collectively as Philippine conservation-relevant areas (PCAs) to clarify the scope of our comparisons and to avoid confusion with the previous use of “priority conservation areas.” We

limit acronym usage to PCA, while "protected and conserved areas" and "key biodiversity areas" are written out in full throughout the text for clarity and consistency.

Reviewer: Lines 130–132: Why the criteria used to avoid duplication was time and not space? Did you drop data from that period indiscriminately without assessing the spatial distribution?

Response: The curated datasets used in Diesmos et al. (2015) and Leviton et al. (2018) were also sourced from GBIF. As a result, many records collected prior to 2015 and 2018 had already undergone rigorous curation, which allowed us to use our time efficiently during the manual data-cleaning process. The text was revised to read:

"To avoid overlap between GBIF records and these curated datasets (also sourced from GBIF), only GBIF records dated post-publication—2015 onwards for amphibians and 2018 onwards for snakes—were retained, since GBIF records for these groups collected before 2015 (for amphibains) and 2018 (for snakes) had already undergone rigorous curation. For lizards, all GBIF records were included since no curated datasets are available."

Reviewer: From Line 139, It is not clear how you warrant the quality of the citizen science data. For example, many records of iNaturalist are from enthusiast observers and not from specialists, leading in some cases to overestimation of records. Careful review of the records will be desired to discard those dubious observations. Please, explain the steps to follow to ensure data quality.

Response: We thank the Reviewer for raising this point. Only research-grade observations were included in the dataset. Although we did not manually curate each individual record, our species-by-species data curation process was designed to flag potentially dubious occurrences. This process relies on genetically confirmed species boundaries reported in the literature, expert-curated species ranges (e.g., GARD), and our own expertise in Philippine herpetofauna systematics. In addition, curated specimens from natural history museums constitute the majority of the dataset (92%).

Reviewer: From Lines 144–145, Please specify which source did you follow to define the current taxonomy nomenclature.

Response: We revised the text to read: "Occurrence records for each species were then manually curated using currently accepted taxonomic treatments and synonyms, following the AmphibiaWeb (AmphibiaWeb, 2025) for amphibians and The Reptile Database (Uetz et al. 2025) for reptiles"

Reviewer: From Line 146: How did you define the geographic range of the species? red list expert maps, national assessment, other?

Response: We have revised the text to read: "Records falling outside the known geographic range of a species were excluded, following genetically confirmed species boundaries from published literature summarized in Meneses et al. 2024 (*recent Philippine herpetology review*), and excluding records lacking verifiable documentation (e.g., curated vouchered specimens or occurrences within the known distribution reported in peer-reviewed literature)."

In addition, we compared our preliminary range estimates for lizards with the Global Assessment of Reptile Distributions (GARD), which is an expert-curated database. We avoided using IUCN Red List maps, as they can be broad and may not always accurately reflect species boundaries.

Reviewer: From Line 183, Definition related to terms like CAs and PCAs is needed. you previously refer to OEMCs and KBA, but this are one of many figures of area based conservation. Review Nick Salafsky lexicon https://conbio.onlinelibrary.wiley.com/doi/10.1111/cobi.14269.

Response: We thank the reviewer for sharing the reference. We followed the definition proposed by Salafsky et al. (2024), distinguishing between protected and conserved areas (i.e., areas with formal management or governance) and key biodiversity areas (i.e., spatial prioritization tools without inherent governance or protection status). In the revised manuscript, we refer to both collectively as Philippine conservation-relevant areas (PCAs) to clarify the scope of our comparisons and to avoid confusion with the previous use of "priority conservation areas." We limit acronym usage to PCA, while "protected and conserved areas" and "key biodiversity areas" are written out in full throughout the text for clarity and consistency.

Reviewer: From lines 217–218, I do not think this is the most appropriate approach. Species diversity is the outcome of multiple environmental and evolutionary factors, which you are not measuring here. Moreover, the manuscript itself argues that there are several sampling biases, which affect the observed patterns of species diversity.

I think that if you want to explore the relationship between species diversity and sampling effort, extrapolation and interpolation methods should be used. These approaches can indicate how well the current knowledge captures species diversity relative to the expected.

Response: We thank the Reviewer for this important clarification. We agree that species diversity arises from multiple environmental and evolutionary processes; for this reason, our focus is on the observed patterns rather than the underlying drivers. To avoid potential mischaracterization, we have used the term species diversity sparingly and consistently refer to counts of species as "observed species richness" throughout the manuscript. We also add a paragraph in the section "Predictors of observed species richness across PCAs":

"Species richness is shaped by a complex interplay of environmental and evolutionary processes. However, the richness values analyzed here reflect observed species counts derived from available occurrence records. Accordingly, our analysis focuses on variation in observed species richness across PCAs and the influence of spatial attributes, rather than attempting to infer the underlying processes that generate biodiversity per se. Specifically, we examined whether area, topographic relief, and sampling effort help account for variation in observed richness, and whether these relationships differ among PCA types. As a complementary analysis, we also tested whether sampling intensity is predicted by these same spatial attributes of PCAs, and whether these relationships vary across PCA types. Calculations for each variable are outlined below, followed by a description of our modeling framework."

Reviewer: From Lines 217–220: Does the model account for the non-independence of the records? Spatial data and regression analysis must deal with spatial patterns and positive autocorrelation in the records. I suggest you review the GLM and evaluate the possibility of

doing a Spatial GLM. An example: https://cran.r-project.org/web/packages/glmmfields/vignettes/spatial-glms.html

Response: We thank the Reviewer for this comment. In our modeling framework, we accounted for the non-independence of samples (306 Philippine conservation-relevant areas) by including biogeographic subregion (PAIC) as a random intercept, as PCAs may share similarity due to shared biogeographic structure. We included the variance explained by PAIC in our results section (which is negligible). We did not use individual occurrence points as model samples; instead, we used summary metrics (specifically, occurrence counts per km^2) as a predictor to serve as a proxy for sampling effort.

Reviewer: From Line 282, Even though amphibians and reptiles share certain traits that allow them to be grouped together, their spatial patterns and global research efforts differ considerably. I suggest splitting the dataset to examine each group separately. If they show different tendencies, you should present the analyses independently; otherwise, they can be grouped, at least for this section.

Response: We acknowledge that amphibians and reptiles differ in their ecology and diversity patterns. However, as stated in the Introduction, these groups were used in this study because, relative to other taxonomic groups in the Philippines, they are comparatively well-documented and thus provide a robust basis for assessing observed biodiversity patterns and sampling distribution across areas. Our objective was not to model amphibian and reptile diversity separately or to compare their biological drivers, but rather to evaluate how observed species richness patterns (across well-sampled vertebrate taxa) relate to area, sampling effort, and topographic relief in the context of biodiversity documentation or conservation planning. Accordingly, our interpretation focuses on spatial patterns of documented diversity rather than taxon-specific ecological differences. In addition, amphibians and reptiles have historically and contemporarily been surveyed and analyzed jointly in the Philippines (see Meneses et al. 2024), making their combined treatment, at least within the context of this paper, consistent with established research practice.

Reviewer: From Lines 312 –313: According to this figure, Conservation Areas (CAs) include both Protected Areas and OECMs. However, these categories differ substantially in their capacity, governance, and origin. Protected Areas are generally designated based on ecosystem representativeness and specialist criteria, whereas OECMs—which are highly diverse in governance and conservation contributions—may include Indigenous territories, private reserves, and other land designations that contribute to conservation objectives such as ecosystem service provision and species protection. Therefore, grouping all CAs together may be misleading, as one cannot assume comparable levels of sampling effort or management capacity across these distinct types.Moreover, the manuscript does not justify why KBAs are analyzed separately from OECMs. An OECM may also qualify as a KBA, and vice versa, so the rationale for treating them as mutually exclusive categories needs clarification. It is necessary to clearly define and categorize the CAs, and to structure the analyses according to these categories.

Response: We thank the Reviewer for this comment. As noted in our earlier response, we adopted the framework proposed by Salafsky et al. (2024), distinguishing between protected and conserved areas (i.e., areas with formal management or governance) and key biodiversity areas (i.e., spatial prioritization tools without formal management or protection). In the revised manuscript, we refer to both collectively as Philippine conservation-relevant areas (PCAs) to

clarify the scope of our comparisons and avoid confusion with the previous use of "priority conservation areas."

Within our dataset, protected and conserved areas include both PAs and OECMs, as both have some form of management or governance, though the mechanisms vary (even among PAs, at least in the Philippines). This distinction is meaningful relative to KBAs, which generally lack formal management and are primarily spatial planning tools. Some KBAs have subsequently been established as protected or conserved areas, although boundaries do not always coincide, due to land-use and socio-political considerations, which we discuss in the manuscript to support interpretation of our results (**see Lines 538–552**). In essence, our split distinguishes managed versus unmanaged conservation-relevant areas**,** while retaining formal terminology commonly used in policy and planning discussions (particularly in the Philippines)

Reviewer: From Line 343, Consider changing this title, as it may lead to misunderstandings. You are not attempting to predict diversity, but rather sampling effort success in terms of species richness.

Response: We revised the phrasing to refer to predictors of observed species richness across conservation areas to maintain consistency with the terminology used throughout the manuscript.

Reviewer: From Line 458, The relationship between sampling completeness and diversity cannot be reliably assessed using the proposed models. I recommend considering interpolation and extrapolation diversity estimators, such as Jackknife or Chao, which are more appropriate for evaluating diversity completeness.

Response: We note that our focus is not on assessing sampling completeness per se, but on evaluating the relationship between sampling effort and observed species richness. Our modeling framework addresses this by including occurrence density as a predictor, serving as a proxy for sampling effort. Accordingly, the resulting observed species richness is interpreted in relation to variation in sampling effort, rather than as a measure of completeness.

Reviewer: From Lines 539–540: Why are they so valuable? Because there are so few records, or because the identified species are threatened or/and endemic?

Response: We revised the text for clarity: "Although many regions remain underrepresented in biodiversity data, it is noteworthy that areas with limited contemporary surveys—as mentioned earlier, Sulu Archipelago, where fieldwork has been limited [25]—still hold valuable records preserved through historical collections, most of which are the only verifiable species records for this biogeographic subregion."

ADDITIONAL JOURNAL REQUIREMENTS

We moved this funding information to the funding statement:

"During the preparation of this manuscript, KMEP was partially supported by a Graduate Research Assistantship from the University of Kansas Department of Ecology and Evolutionary Biology; and RMB was supported by a Fulbright International Scholar Award administered by the U.S. Department of State, the Philippine-American Education Foundation (PAEF) and the Philippine Council for International Exchange of Scholars. Support for fieldwork that generated

many specimen-associated records was provided by the U.S. National Science Foundation (DEB 073199, 0743491, 1418895, 0910341, 0804115, 1654388, and 0334952 and EF-0334952 to RMB and KU-based students), the KU College of Liberal Arts and Sciences Graduate Assistantship in Support of Faculty Research Award, a KU Biodiversity Institute Research and Exploration Fund grant, the South, Southeast, and East Asia Fund of the KU Office of International Affairs, and the General Research Fund of the University of Kansas. The funders had no role in study design, data collection and analysis, decision to publish, or preparation of the manuscript."

Figures

Regarding the copyright of Figures 1, 2, and S5, we confirm that these maps were independently generated by the authors using open-access geospatial data. The foundational shapefiles for protected and conserved areas and key biodiversity areas were sourced from the National Integrated Protected Area System (NIPAS) dataset, while the national and administrative boundary shapefiles were provided by the National Mapping and Resource Information Auth ority (NAMRIA).

All layers were accessed via the Geoportal Philippines. According to the official Data Sharing Agreement managed by NAMRIA, the originating agencies (including DENR-BMB) authorize the public to 'freely download, reuse and share' this geospatial data (Clause f). Furthermore, the datasets' metadata indicates there are no access or use constraints. As the authors have used this unrestricted data to create original map visualizations, these figures are compatible with the CC BY 4.0 license and include proper attribution to the DENR-BMB and NAMRIA as the primary data sources.

(https://www.geoportal.gov.ph/resources/DataSharingAgreement.docx)

Data Review URL

Click here to download Data Review URL
http://zenodo.org/records/17344465
