# Supplementary material for: Sampling bias and uneven data coverage shape observed biodiversity patterns in a megadiverse island archipelago hotspot
Source: PLoS One. 2026 Jul 17;21(7):e0353393. doi: 10.1371/journal.pone.0353393 (PMC13379091; doi:10.1371/journal.pone.0353393)
Supplement: Attachment — Submitted filename: Response to Reviewers.docx [file pone.0353393.s008.docx]

PONE-D-25-55790
**Response to Reviewers**

**March 20, 2026**

**Juan C. Diaz-Ricaurte**

**Academic Editor, PLOS One**

Dear Dr. Diaz-Ricaurte,

Thank you for the opportunity to submit a revised version of our manuscript, “*Sampling bias obscures biodiversity patterns, reveals data gaps in a megadiverse island archipelago hotspot*,” for consideration in PLOS ONE. On behalf of my co-authors, I sincerely thank you and the reviewers for your thoughtful and constructive comments. These suggestions have substantially improved the clarity, coherence, and scientific rigor of the manuscript.

In the revised version, we have modified the title by replacing “priority conservation areas” with “megadiverse island archipelago hotspot” to ensure consistency with terminology used throughout the manuscript, as further explained in our responses below. We also removed “*a call for improved documentation*” to shorten title. We provide detailed, point-by-point responses to all comments following this letter for your review and consideration.

Sincerely,

Kier Mitchel E. Pitogo

**EDITOR**

**Editor:** A first overarching issue, raised primarily by Reviewer 1, concerns the alignment between research questions and analytical choices. Although the research questions are clearly stated, the links between each question, the statistical models used, and the corresponding results are not always explicit. I strongly suggest that the revised manuscript make these connections clear and systematic. Each main question should be directly associated with the analysis used to address it and with the relevant results, as this alignment is essential for transparency and interpretability.

**Response:** We have revised the phrasing of Questions 3 and 4 to better align with the analyses conducted. In the Methods section, we now state each specific objective before describing the corresponding analytical approach to clarify the connection between the questions and the analyses. The Results section also follow the same structure.

**Editor:** A major methodological concern, discussed in detail by Reviewer 1 and reinforced by Reviewer 2, relates to the framework used to compare different categories of conservation areas. Much of the manuscript’s inference depends on comparing sampling effort and diversity patterns across conservation area types, yet the current analytical approach does not allow formal statistical testing of differences among these categories. I agree with both reviewers that this represents a substantive limitation. For the revision to be satisfactory, these comparisons should be conducted within a unified modeling framework that allows direct statistical evaluation of differences in effects across conservation area types.

**Response:** We reanalyzed the dataset using a joint modeling framework in which species richness was modeled as a function of area, topographic relief, and occurrence density, including all pairwise interactions among these predictors and their interactions with PCA type (protected and conserved areas vs. key biodiversity areas). Biogeographic subregion was included as a random intercept to account for non-independence among PCAs due to shared biogeographic structure. Results were broadly consistent with our previous approach. However, we retain this unified model as it more directly addresses our specific questions, as recommended by the reviewers as well.

**Editor:** Reviewer 1 emphasizes the importance of retaining model components that are justified by the study design, particularly grouping or hierarchical variables included to account for non-independence among observations.

**Response:** In our new models, we retained the biogeographic subregion (Pleistocene Aggregate Island Complexes or PAIC) as a random effect. Variance explained for the random effect are also reported in the results section, alongside the coefficient estimates of the fixed predictors.

**Editor:** The spatial properties of the dataset constitute another key issue raised by both reviewers from complementary perspectives. While reviewer 1 focuses on spatial precision, including how coordinate uncertainty was handled and whether the chosen grid resolution is appropriate given the characteristics of the occurrence records, the reviewer 2 emphasizes the need to explicitly address spatial dependence among observations. Given the spatially explicit nature of the analyses, I agree that these issues cannot be treated as secondary. The revised manuscript should clearly justify the analytical scale, address positional uncertainty, and either incorporate spatial structure into the models or provide a clear and defensible rationale for not doing so.

**Response:** We thank both Reviewers for highlighting the inherent challenges of spatially explicit analyses. In response to Reviewer 1, we have clarified in the revised manuscript our rationale for the selected 10-km grid resolution (**lines 200–204**) and our treatment of coordinate uncertainty (**lines 184–190**). The choice of a 10-km grid resolution was deliberate and intended to provide an appropriate balance for visualizing broad-scale spatial patterns of observed species richness patterns across the Philippines.

Regarding spatial uncertainty, we retained coordinates that were consistent with general (provincial-level) locality information, as these adequately reflect broad geographic distribution patterns (country-wide assessment). Our sampling is broad enough and has a level of certainty where broad patterns will be discovered. Although minor spatial imprecision may influence species richness estimates within individual PCAs used in the modeling, any resulting bias is expected to be minimal because most PCAs are supported by numerous occurrence records, species are represented by multiple points within PCAs due to the integration of multiple data sources, and the overall sample size is large (n = 316 PCAs).

**Editor:** Both reviewers also identify limitations in the biological interpretation of the results, particularly regarding the joint treatment of amphibians and reptiles. These groups differ in ecology, detectability, sampling intensity, and conservation context. I suggest that the authors either explicitly assess whether these taxa exhibit similar or contrasting patterns, or clearly justify their combined analysis. Without this clarification, the ecological interpretation of the results remains incomplete.

**Response:** We acknowledge that amphibians and reptiles differ in their ecology and diversity patterns. However, as stated in the Introduction, these groups were used in this study because, relative to other taxonomic groups in the Philippines, they are comparatively well-documented and thus provide a robust basis for assessing observed biodiversity patterns and sampling distribution across areas. Our objective was not to model amphibian and reptile diversity separately or to compare their biological drivers (e.g., fine-scale environmental variables), but rather to evaluate how observed species richness patterns relate to area, sampling effort, and topographic relief in the context of biodiversity documentation or conservation planning. Accordingly, our interpretation focuses on spatial patterns of documented diversity rather than taxon-specific ecological differences. In addition, amphibians and reptiles have historically and contemporarily been surveyed and analyzed jointly in the Philippines (see Meneses et al. 2024), making their combined treatment, at least within the context of this paper, consistent with established research practice.

**Editor:** Further clarification is also required regarding the conceptual framing of conservation areas. Reviewer 2 raises questions about how different conservation categories are defined and compared, while Reviewer 1 highlights the difficulty of interpreting area-based comparisons under strong and uneven sampling biases. I agree with both assessments and recommend that the revised manuscript explicitly state the assumptions underlying these comparisons and clearly articulate how results should be interpreted in light of known data limitations.

**Response:** We recognize that the conceptual framing and terminology of conservation areas required consistency in the original submission. Following the recommendation of the Reviewer, we adopted the lexicon proposed by Salafsky et al. (2024), distinguishing between protected and conserved areas (i.e., areas with formal management or governance) and key biodiversity areas (i.e., spatial prioritization tools without inherent governance or protection status). In the revised manuscript, we refer to both collectively as Philippine conservation-relevant areas (PCAs) to clarify the scope of our comparisons and to avoid confusion with the previous use of “priority conservation areas.” We limit acronym usage to PCA, while “protected and conserved areas” and “key biodiversity areas” are written out in full throughout the text for clarity and consistency.

Regarding concerns about interpretation under uneven sampling effort, we model observed species richness as a function of area, topographic relief, and occurrence density (as a proxy for sampling effort), with biogeographic subregion included as a random intercept to account for geographic clustering. This framework allows us to assess whether differences in observed richness among PCA types persist after accounting for variation in sampling intensity (and other predictors) and shared biogeographic structure. In addition, we modeled occurrence points as a function of area and topographic relief, again including biogeographic subregion as a random effect, to evaluate whether sampling effort itself varies systematically between PCA types (which we found no sufficient evidence). Finally, we clarify in the Discussion that our results reflect documented (observed) diversity patterns and should be interpreted in light of known data limitations.

**Editor:** Both reviewers note that several figures (particularly those illustrating spatial patterns) are difficult to interpret due to low resolution and small text. This may be related to how figures are displayed in the submission system; however, I recommend you include all figures in the revised version at the highest resolution possible to facilitate careful evaluation by the reviewers.

**Response:** We will upload the highest-resolution version permitted by the submission system. The original figures were generated at high resolution; however, the conversion to PDF within the system may have reduced their clarity.

**REVIEWER 1**

**Reviewer:** I would suggest that the authors more explicitly connect each research question to the specific model(s) used. Clarifying which models and results correspond to which of the four main questions would enhance the transparency and coherence of the study design.

**Response:** We thank the Reviewer for raising this clarification. In the revised methods section, we now state each specific objective before describing the corresponding analytical approach to clarify the connection between the questions and the analyses. The Results section also follow the same structure.

**Reviewer:** Thank you for the detailed description of the data curation procedures. All the steps were important to guarantee the quality of the input data. However, I would like to raise a question regarding the spatial precision of the occurrence records used.

While the manuscript mentions the exclusion of records outside known distributions and the removal of generic coordinates, I did not find any reference to the use of the “coordinate uncertainty in meters” field (available in GBIF records) or a similar assessment of the dataset's spatial accuracy. Given that the study employs a 10-km grid resolution, I wonder whether this scale is appropriate considering the potential variability in coordinate precision, especially for historical museum records which can lack fine-scale georeferencing.

Could the authors clarify whether any filtering based on coordinate uncertainty was applied? If not, do the authors consider the 10-km resolution robust to such imprecision, and how might this potentially affect the spatial allocation of records and the resulting diversity patterns?

**Response:** We thank the reviewer for highlighting the issue of spatial precision. Because relatively few occurrence records include explicit uncertainty, we did not filter based on this metric. Instead, we retained records with geographic coordinates that correspond to general geographic location (provincial level). All records broadly reflect geographic patterns of each species distribution, and any minor imprecision is expected to have minimal impact on broad-scale, country-wide patterns. As stated above, although minor spatial imprecision may influence species richness estimates within individual PCAs used in the modeling, any resulting bias is expected to be minimal because most PCAs are supported by numerous occurrence records, species are represented by multiple points within PCAs due to the integration of multiple data sources, and the overall sample size is large (n = 316 PCAs).

The 10-km grid resolution was also deliberately chosen to visualize broad-scale distribution patterns across the Philippines. This scale provides an appropriate balance: smaller grids (e.g., <10 km) would be too fragmented on larger islands, while larger grids (e.g., 15–20 km) could average out meaningful spatial variation, particularly on smaller islands. We have included this justification in the revised manuscript (**Lines 200–204**).

**Reviewer:** The current analysis utilizes Negative Binomial GLMs fitted separately for Protected Areas (PAs) and Key Biodiversity Areas (KBAs). The methodological rationale for this decision is not fully clear in the manuscript. If this separation was a deliberate choice to address, for example, severe variance heterogeneity, it would be very helpful for the reader if this justification were made explicit.

My main concern with this approach is that a central objective of the study appears to be the comparison of predictor effects (e.g., area, topographic relief), and the comparison of richness and effort between PAs and KBAs. Adjusting separate models, while legitimate for describing the relationships within each group, impedes a formal statistical comparison of the coefficient estimates.

To formally test the hypothesis that a predictor's effect differs between PAs and KBAs, the standard and more robust statistical approach would be to analyze both groups within a single, unified model. This is typically achieved by including the group affiliation (PA vs. KBA) as a categorical predictor and, crucially, testing interaction terms between this group variable and the predictors of interest (e.g., PCA_Type * Area). The p-value for the interaction term provides the formal statistical test of the null hypothesis that there is no difference in the slopes between the groups.

It is plausible that adopting this unified model approach would yield substantive results similar to those already presented. However, its key advantage would be to make the comparative claims—which are central to the study's objectives—statistically explicit and defensible, thereby increasing the analysis's robustness and interpretive clarity.

**Response:** We thank the Reviewer for this thoughtful and constructive feedback. In the revised manuscript, we reanalyzed the dataset using a joint modeling framework in which species richness was modeled as a function of area, topographic relief, and occurrence density, including all pairwise interactions among these predictors and their interactions with PCA type. Biogeographic subregion was included as a random intercept to account for non-independence among PCAs due to shared biogeographic structure. Although the results are broadly consistent with our previous approach, as the Reviewer noted, this framework allows for a more direct comparison between PCA types and highlights additional patterns for discussion. Hence, we kept this unified modelling framework. We have revised the Methods (**lines 228–317**), Results (**lines 401–465**), and Discussion (**lines 522–578**) sections accordingly.

**Reviewer:** My final methodological point relates to the model selection for control effects. The authors state that the random effect variable "PAIC" was removed from one of the models because it "explained less than 1% of the variance and did not improve model performance.” (Line 234).

A low number of levels (seven, in this case) can lead to imprecise variance estimates. However, the decision to include or exclude a structural random effect should be based on the study design, not on its statistical performance (such as variance explained or AIC).

As the 'PAIC' variable represents a grouping variable (e.g., island/region) dictated by the sampling design, its function in the model is not primarily to "explain variance" (as in a predictive model) , but rather to control for the non-independence of the data (i.e., pseudoreplication). Observations from the same PAIC group are likely to be more similar to each other, violating the assumption of independence. The literature strongly advocates for retaining the random effects structure justified by the design. Removing this structural term, even if its variance is low, can lead to "overoptimistic" results, such as artificially reduced standard errors and an inflated Type I error rate. (Barr, D. J., Levy, R., Scheepers, C., & Tily, H. J. (2013). Random effects structure for confirmatory hypothesis testing: Keep it maximal. Journal of Memory and Language, 68(3), 255-278. <https://doi.org/10.1016/j.jml.2012.11.001>)

I would, therefore, recommend that the authors retain the 'PAIC' random effect in the final models to ensure that potential non-independence in the data is appropriately controlled for, thereby strengthening the validity of the model's inferences.

**Response:** We thank the Reviewer for this critical point. In our new models, we retained the biogeographic subregion (Pleistocene Aggregate Island Complexes or PAIC) as a random effect. Variance explained for the random effect are also reported in the results section, alongside the coefficient estimates of the fixed predictors.

**Reviewer:** Regarding the presentation of these results, I found many of the figures, both in the supplementary material and - more significantly - in the main manuscript, difficult to interpret: the resolution appears to be low, and the font size for labels and legends is very small. I would recommend the authors provide higher-resolution figures with more legible text to ensure readers can fully interpret the study’s important spatial results.

**Response:** We will upload the highest-resolution version permitted by the submission system. The original figures were generated at high resolution; however, the conversion to PDF within the system may have reduced their clarity.

**REVIEWER 2**

Our responses address the specific comments provided in the annotated manuscript, which correspond closely to the summarized remarks. Accordingly, we have responded directly to those in-text comments.

**Reviewer:** Lines 84–85: You are mixing terms and categories. Key Biodiversity Areas (KBAs) are referred to in the manuscript as Priority Conservation Areas (PCAs), yet the models and figures still use the term “KBAs.” This inconsistency creates confusion for the reader.

Furthermore, it is not clear which criteria were used to spatially define the Priority Conservation Areas. Based on the description, it appears that these areas correspond to the officially recognized KBAs in the Philippines. If that is the case, then the PCAs in your study are essentially KBAs, and this should be explicitly stated to avoid misinterpretation.

**Response:** We thank the Reviewer for raising this point. We recognize that the conceptual framing and terminology of conservation areas required greater precision or consistency in the original submission. Following the recommendation, we adopted the lexicon proposed by Salafsky et al. (2024), distinguishing between protected and conserved areas (i.e., areas with formal management or governance) and key biodiversity areas (i.e., spatial prioritization tools without inherent governance or protection status). In the revised manuscript, we refer to both collectively as Philippine conservation-relevant areas (PCAs) to clarify the scope of our comparisons and to avoid confusion with the previous use of “priority conservation areas.” We limit acronym usage to PCA, while “protected and conserved areas” and “key biodiversity areas” are written out in full throughout the text for clarity and consistency.

**Reviewer:** Lines 130–132: Why the criteria used to avoid duplication was time and not space? Did you drop data from that period indiscriminately without assessing the spatial distribution?

**Response:** The curated datasets used in Diesmos et al. (2015) and Leviton et al. (2018) were also sourced from GBIF. As a result, many records collected prior to 2015 and 2018 had already undergone rigorous curation, which allowed us to use our time efficiently during the manual data-cleaning process. The text was revised to read:

“To avoid overlap between GBIF records and these curated datasets (also sourced from GBIF), only GBIF records dated post-publication—2015 onwards for amphibians and 2018 onwards for snakes—were retained, since GBIF records for these groups collected before 2015 (for amphibains) and 2018 (for snakes) had already undergone rigorous curation. For lizards, all GBIF records were included since no curated datasets are available.”

**Reviewer:** From Line 139, It is not clear how you warrant the quality of the citizen science data. For example, many records of iNaturalist are from enthusiast observers and not from specialists, leading in some cases to overestimation of records. Careful review of the records will be desired to discard those dubious observations. Please, explain the steps to follow to ensure data quality.

**Response:** We thank the Reviewer for raising this point. Only research-grade observations were included in the dataset. Although we did not manually curate each individual record, our species-by-species data curation process was designed to flag potentially dubious occurrences. This process relies on genetically confirmed species boundaries reported in the literature, expert-curated species ranges (e.g., GARD), and our own expertise in Philippine herpetofauna systematics. In addition, curated specimens from natural history museums constitute the majority of the dataset (92%).

**Reviewer:** From Lines 144–145, Please specify which source did you follow to define the current taxonomy nomenclature.

**Response:** We revised the text to read: “Occurrence records for each species were then manually curated using currently accepted taxonomic treatments and synonyms, following the AmphibiaWeb (AmphibiaWeb, 2025) for amphibians and The Reptile Database (Uetz et al. 2025) for reptiles”

**Reviewer**: From Line 146: How did you define the geographic range of the species? red list expert maps, national assessment, other?

**Response:** We have revised the text to read: “Records falling outside the known geographic range of a species were excluded, following genetically confirmed species boundaries from published literature summarized in Meneses et al. 2024 (*recent Philippine herpetology review*), and excluding records lacking verifiable documentation (e.g., curated vouchered specimens or occurrences within the known distribution reported in peer-reviewed literature).”

In addition, we compared our preliminary range estimates for lizards with the Global Assessment of Reptile Distributions (GARD), which is an expert-curated database. We avoided using IUCN Red List maps, as they can be broad and may not always accurately reflect species boundaries.

**Reviewer:** From Line 183, Definition related to terms like CAs and PCAs is needed. you previously refer to OEMCs and KBA, but this are one of many figures of area based conservation. Review Nick Salafsky lexicon <https://conbio.onlinelibrary.wiley.com/doi/10.1111/cobi.14269>.

**Response:** We thank the reviewer for sharing the reference. We followed the definition proposed by Salafsky et al. (2024), distinguishing between protected and conserved areas (i.e., areas with formal management or governance) and key biodiversity areas (i.e., spatial prioritization tools without inherent governance or protection status). In the revised manuscript, we refer to both collectively as Philippine conservation-relevant areas (PCAs) to clarify the scope of our comparisons and to avoid confusion with the previous use of “priority conservation areas.” We limit acronym usage to PCA, while “protected and conserved areas” and “key biodiversity areas” are written out in full throughout the text for clarity and consistency.

**Reviewer:** From lines 217–218, I do not think this is the most appropriate approach. Species diversity is the outcome of multiple environmental and evolutionary factors, which you are not measuring here. Moreover, the manuscript itself argues that there are several sampling biases, which affect the observed patterns of species diversity.

I think that if you want to explore the relationship between species diversity and sampling effort, extrapolation and interpolation methods should be used. These approaches can indicate how well the current knowledge captures species diversity relative to the expected.

**Response:** We thank the Reviewer for this important clarification. We agree that species diversity arises from multiple environmental and evolutionary processes; for this reason, our focus is on the observed patterns rather than the underlying drivers. To avoid potential mischaracterization, we have used the term species diversity sparingly and consistently refer to counts of species as “observed species richness” throughout the manuscript. We also add a paragraph in the section “Predictors of observed species richness across PCAs”:

“Species richness is shaped by a complex interplay of environmental and evolutionary processes. However, the richness values analyzed here reflect observed species counts derived from available occurrence records. Accordingly, our analysis focuses on variation in observed species richness across PCAs and the influence of spatial attributes, rather than attempting to infer the underlying processes that generate biodiversity per se. Specifically, we examined whether area, topographic relief, and sampling effort help account for variation in observed richness, and whether these relationships differ among PCA types. As a complementary analysis, we also tested whether sampling intensity is predicted by these same spatial attributes of PCAs, and whether these relationships vary across PCA types. Calculations for each variable are outlined below, followed by a description of our modeling framework.”

Reviewer: From Lines 217–220: Does the model account for the non-independence of the records? Spatial data and regression analysis must deal with spatial patterns and positive autocorrelation in the records. I suggest you review the GLM and evaluate the possibility of doing a Spatial GLM. An example: <https://cran.r-project.org/web/packages/glmmfields/vignettes/spatial-glms.html>

**Response:** We thank the Reviewer for this comment. In our modeling framework, we accounted for the non-independence of samples (306 Philippine conservation-relevant areas) by including biogeographic subregion (PAIC) as a random intercept, as PCAs may share similarity due to shared biogeographic structure. We included the variance explained by PAIC in our results section (which is negligible). We did not use individual occurrence points as model samples; instead, we used summary metrics (specifically, occurrence counts per km²) as a predictor to serve as a proxy for sampling effort.

Reviewer: From Line 282, Even though amphibians and reptiles share certain traits that allow them to be grouped together, their spatial patterns and global research efforts differ considerably. I suggest splitting the dataset to examine each group separately. If they show different tendencies, you should present the analyses independently; otherwise, they can be grouped, at least for this section.

**Response:** We acknowledge that amphibians and reptiles differ in their ecology and diversity patterns. However, as stated in the Introduction, these groups were used in this study because, relative to other taxonomic groups in the Philippines, they are comparatively well-documented and thus provide a robust basis for assessing observed biodiversity patterns and sampling distribution across areas. Our objective was not to model amphibian and reptile diversity separately or to compare their biological drivers, but rather to evaluate how observed species richness patterns (across well-sampled vertebrate taxa) relate to area, sampling effort, and topographic relief in the context of biodiversity documentation or conservation planning. Accordingly, our interpretation focuses on spatial patterns of documented diversity rather than taxon-specific ecological differences. In addition, amphibians and reptiles have historically and contemporarily been surveyed and analyzed jointly in the Philippines (see Meneses et al. 2024), making their combined treatment, at least within the context of this paper, consistent with established research practice.

**Reviewer:** From Lines 312 –313: According to this figure, Conservation Areas (CAs) include both Protected Areas and OECMs. However, these categories differ substantially in their capacity, governance, and origin. Protected Areas are generally designated based on ecosystem representativeness and specialist criteria, whereas OECMs—which are highly diverse in governance and conservation contributions—may include Indigenous territories, private reserves, and other land designations that contribute to conservation objectives such as ecosystem service provision and species protection. Therefore, grouping all CAs together may be misleading, as one cannot assume comparable levels of sampling effort or management capacity across these distinct types.Moreover, the manuscript does not justify why KBAs are analyzed separately from OECMs. An OECM may also qualify as a KBA, and vice versa, so the rationale for treating them as mutually exclusive categories needs clarification. It is necessary to clearly define and categorize the CAs, and to structure the analyses according to these categories.

**Response:** We thank the Reviewer for this comment. As noted in our earlier response, we adopted the framework proposed by Salafsky et al. (2024), distinguishing between **protected and conserved areas** (i.e., areas with formal management or governance) and **key biodiversity areas** (i.e., spatial prioritization tools without formal management or protection). In the revised manuscript, we refer to both collectively as **Philippine conservation-relevant areas (PCAs)** to clarify the scope of our comparisons and avoid confusion with the previous use of “priority conservation areas.”

Within our dataset, protected and conserved areas include both PAs and OECMs, as both have some form of management or governance, though the mechanisms vary (even among PAs, at least in the Philippines). This distinction is meaningful relative to KBAs, which generally lack formal management and are primarily spatial planning tools. Some KBAs have subsequently been established as protected or conserved areas, although boundaries do not always coincide, due to land-use and socio-political considerations, which we discuss in the manuscript to support interpretation of our results (**see Lines 538–552**). In essence, our split distinguishes **managed versus unmanaged conservation-relevant areas,** while retaining formal terminology commonly used in policy and planning discussions (particularly in the Philippines)

**Reviewer:** From Line 343, Consider changing this title, as it may lead to misunderstandings. You are not attempting to predict diversity, but rather sampling effort success in terms of species richness.

**Response:** We revised the phrasing to refer to predictors of observed species richness across conservation areas to maintain consistency with the terminology used throughout the manuscript.

**Reviewer:** From Line 458, The relationship between sampling completeness and diversity cannot be reliably assessed using the proposed models. I recommend considering interpolation and extrapolation diversity estimators, such as Jackknife or Chao, which are more appropriate for evaluating diversity completeness.

**Response:** We note that our focus is not on assessing sampling completeness per se, but on evaluating the relationship between sampling effort and observed species richness. Our modeling framework addresses this by including occurrence density as a predictor, serving as a proxy for sampling effort. Accordingly, the resulting observed species richness is interpreted in relation to variation in sampling effort, rather than as a measure of completeness.

**Reviewer:** From Lines 539–540: Why are they so valuable? Because there are so few records, or because the identified species are threatened or/and endemic?

**Response:** We revised the text for clarity: “Although many regions remain underrepresented in biodiversity data, it is noteworthy that areas with limited contemporary surveys—as mentioned earlier, Sulu Archipelago, where fieldwork has been limited [25]—still hold valuable records preserved through historical collections, most of which are the only verifiable species records for this biogeographic subregion.”

**ADDITIONAL JOURNAL REQUIREMENTS**

We moved this funding information to the funding statement:

“During the preparation of this manuscript, KMEP was partially supported by a Graduate Research Assistantship from the University of Kansas Department of Ecology and Evolutionary Biology; and RMB was supported by a Fulbright International Scholar Award administered by the U.S. Department of State, the Philippine-American Education Foundation (PAEF) and the Philippine Council for International Exchange of Scholars. Support for fieldwork that generated many specimen-associated records was provided by the U.S. National Science Foundation (DEB 073199, 0743491, 1418895, 0910341, 0804115, 1654388, and 0334952 and EF-0334952 to RMB and KU-based students), the KU College of Liberal Arts and Sciences Graduate Assistantship in Support of Faculty Research Award, a KU Biodiversity Institute Research and Exploration Fund grant, the South, Southeast, and East Asia Fund of the KU Office of International Affairs, and the General Research Fund of the University of Kansas. The funders had no role in study design, data collection and analysis, decision to publish, or preparation of the manuscript.”

**Figures**

Regarding the copyright of Figures 1, 2, and S5, we confirm that these maps were independently generated by the authors using open-access geospatial data. The foundational shapefiles for protected and conserved areas and key biodiversity areas were sourced from the National Integrated Protected Area System (NIPAS) dataset, while the national and administrative boundary shapefiles were provided by the National Mapping and Resource Information Auth ority (NAMRIA).

All layers were accessed via the Geoportal Philippines. According to the official Data Sharing Agreement managed by NAMRIA, the originating agencies (including DENR-BMB) authorize the public to 'freely download, reuse and share' this geospatial data (Clause f). Furthermore, the datasets' metadata indicates there are no access or use constraints. As the authors have used this unrestricted data to create original map visualizations, these figures are compatible with the CC BY 4.0 license and include proper attribution to the DENR-BMB and NAMRIA as the primary data sources.

(https://www.geoportal.gov.ph/resources/DataSharingAgreement.docx)
