# Supplementary material for: Sampling bias and uneven data coverage shape observed biodiversity patterns in a megadiverse island archipelago hotspot
Source: PLoS One. 2026 Jul 17;21(7):e0353393. doi: 10.1371/journal.pone.0353393 (PMC13379091; doi:10.1371/journal.pone.0353393)
Supplement: Attachment — Submitted filename: Response_to_Reviewers_auresp_2.docx [file pone.0353393.s009.docx]

PONE-D-25-55790

**May 16, 2026**

**Juan C. Diaz-Ricaurte**

**Academic Editor, PLOS One**

Dear Dr. Diaz-Ricaurte,

Thank you for your careful evaluation of our revised manuscript and for the continued feedback from both reviewers. We appreciate the opportunity to further clarify the analytical framework, model structure, and inferential scope of the study.

In this revision, we improved internal consistency in how we interpret observed species richness patterns, sampling heterogeneity, and the role of occurrence records as a proxy for sampling effort. We clarified throughout the manuscript that our analyses focus on observed biodiversity patterns derived from heterogeneous, digitally accessible occurrence datasets, rather than attempting to infer unbiased estimates of underlying ecological richness. This clarification is now consistently reflected across the manuscript.

We also addressed Reviewer 1’s concerns by implementing an explicit AICc-based model selection procedure. This confirmed the retained full interaction model as the best-supported among the candidate set, and we now report this procedure in the Methods and Supplementary Materials. We additionally clarified the rationale for retaining interaction terms based on the study objectives, which focus on testing whether relationships between predictors and observed richness differ between PCA types. Occurrence density is now more explicitly framed as a proxy for sampling intensity that may also reflect biological signal.

Regarding Reviewer 2’s concern on spatial dependence, we clarify that the analytical unit of inference in this study is the Philippine conservation-relevant area (PCA), which represents irregular areal units defined by conservation designations rather than point-referenced sampling locations. Within this framework, spatial structure is already partially accounted for through a PAIC-level random intercept, which captures broad-scale biogeographic non-independence among island groups across the archipelago. We acknowledge that Reviewer 2 raises an important point regarding spatial autocorrelation in the sense of distance-based or continuous spatial dependence among nearby observations. However, such structures are not directly identifiable at the level of aggregated spatial polygons used here, where observations are already summarized within heterogeneous conservation units. Modeling continuous spatial covariance (e.g., spatial random fields or distance-based autocorrelation structures) would require a fundamentally different data structure based on point-referenced sampling locations, which is not the scale of inference used in this study. We believe we have clarified in the manuscript that our inference is explicitly conducted at the PCA level, and that PAIC captures the relevant broad-scale spatial structure appropriate to this analytical scale.

Regarding Reviewer 2’s concern about the joint analysis of amphibians and reptiles, we emphasize that the objective of this study is to evaluate spatial structure in observed biodiversity data, rather than to infer taxon-specific ecological processes. Both the Introduction and Discussion are explicitly framed around biodiversity documentation, sampling coverage, and spatial data gaps. Within this context, amphibians and reptiles are included as two of the most consistently and extensively sampled vertebrate groups in the Philippines and are therefore treated jointly as a composite dataset for assessing patterns in biodiversity data across conservation-relevant areas. We further note that separating these groups would shift the analytical and interpretive focus toward taxon-specific comparisons, which would diverge from the current narrative and scope of the manuscript. For this reason, we retain the original combined framework to maintain consistency with the study’s stated objectives, analytical design, and scope of inference.

We believe these revisions improve clarity regarding the study’s scope, analytical structure, and interpretive limits. We sincerely appreciate the time and effort invested by you and the reviewers.

Sincerely,
Kier Mitchel Pitogo and co-authors

**REVIEWER 1**

**On model complexity**

We thank Reviewer 1 for this helpful suggestion. We agree that the previous revision included a comparatively complex interaction structure that required clearer justification and interpretation. In response, we simplified the model by retaining area, topographic relief, occurrence density, all pairwise interactions among these predictors, PCA type, and interactions between PCA type and each spatial predictor (PAIC as random effect). We additionally implemented a model selection procedure using AICc-based comparison among candidate models. This procedure confirmed the retained interaction structure as the best-supported model, and we now explicitly describe the model selection approach in the Methods and report the top-supported candidate models in the Supplementary Material (Table S1).

Lines 292 –294:

“*Model selection supported retention of the hypothesized full interaction structure and the random intercept, which had the lowest AICc among candidate models and was therefore used for inference (S1 Table)*.”

Importantly, the retained interaction structure was biologically and analytically motivated because one of the primary objectives of the study was to determine whether the relationships between observed species richness and spatial predictors differ between PCA types. We clarified this rationale in the revised manuscript.

**On occurrence density as sampling effort**

We agree that total occurrence records are an imperfect proxy for sampling effort and may partly reflect biological signal, detectability, or true variation in abundance rather than observer effort alone. Because standardized sampling-effort metrics are not available consistently across the compiled datasets, occurrence density represented the only broadly comparable metric available at the national scale. To address this concern, we revised the manuscript to treat this variable more cautiously throughout. Specifically, we now explicitly acknowledge that occurrence density may reflect both sampling-related and biological processes, and we avoid interpreting it as a pure measure of observer effort. From lines 274–277, it reads:

“*Occurrence density was included as a proxy for sampling effort, while acknowledging that it may also reflect underlying biological variation in abundance and detectability. As such, it represents a composite measure rather than a purely independent estimate of survey effort, and results involving this variable are interpreted with this limitation in mind.*”

We also revised several sections of the Results and Discussion to clarify that the analyses evaluate relationships involving observed richness patterns rather than definitive ecological richness estimates (see Manuscript with tracked changes).

**On apparent contradiction regarding sampling bias**

We appreciate this important observation and agree that the previous revision did not sufficiently distinguish between broad-scale geographic sampling heterogeneity across the Philippines and relationships evaluated within the PCA-level modeling framework.

At the national scale, our analyses demonstrate strong geographic heterogeneity in biodiversity documentation and digitally accessible occurrence records, indicating uneven spatial coverage across the archipelago. In this context, observed richness patterns likely reflect, in part, where biodiversity sampling has historically been concentrated.

In contrast, the PCA-level analyses address a different question: whether variation in observed species richness among conservation units is associated with area, occurrence density, topographic relief, and PCA type after accounting for these variables simultaneously within a unified modeling framework. Within these models, we found no strong evidence that baseline differences between PCA types are explained solely by occurrence density or systematic differences in sampling intensity.

We revised the Discussion throughout to more consistently frame the results in terms of observed or documented richness patterns, while explicitly recognizing that both sampling structure and biological variation likely contribute to the relationships recovered in the analyses.

**On figure readability**

We thank the reviewer for this suggestion. Figure readability, including font size, was carefully revised during the previous round of revision, and we would prefer to retain the current formatting in the present version. Because several figures contain multiple map layers, panels, legends, and annotations within limited space, further increasing font sizes disrupts the visual balance of the figures and compresses other important graphical elements.

We carefully explored different formatting arrangements through multiple rounds of adjustment and found that the current layout provides the best compromise between readability, spatial clarity, and overall figure composition. We therefore respectfully maintain the present figure formatting. We thank you for your understanding.

**REVIEWER 2**

**On spatial autocorrelation**

We appreciate the reviewer’s concern regarding spatial autocorrelation. In our study, the sampling units are Philippine conservation-relevant areas (PCAs), which are irregular spatial polygons that vary substantially in size and shape. The response variable is therefore aggregated species richness at the level of these conservation units rather than point-based occurrence observations. Within this framework, the relevant source of non-independence is broad-scale biogeographic similarity among PCAs rather than fine-scale spatial covariance among individual occurrence records.

To account for this structure, we included Pleistocene Aggregate Island Complex (PAIC) as a random intercept, which captures major regional biogeographic partitioning across the Philippine archipelago at the analytical scale of inference. We further note that commonly used spatial autocorrelation approaches (e.g., distance-based covariance structures or spatial random fields) generally assume comparable spatial units represented by fixed coordinates or adjacency structures. Applying such methods to highly heterogeneous conservation polygons would require additional centroid- or adjacency-based simplifications that are not directly supported by the structure of the dataset.

Lines 287 –291 now reads:

“*To account for potential spatial non-independence among PCAs within biogeographic regions (i.e., PCAs share more similarity in species composition by virtue of historical biogeographic structuring),* ***we included the Pleistocene Aggregate Island Complex (PAIC; see [27]) as a random intercept*** *in all models.* ***This structure reflects the analytical scale of inference, which is defined at the level of heterogenous PCA polygons rather than individual occurrence points****.*”

**On combining amphibians and reptiles**

We respectfully maintain the joint analysis of amphibians and reptiles because separating these groups would substantially alter the scope and objective of the study. The manuscript does not aim to infer taxon-specific ecological drivers of diversity or compare amphibian and reptile ecology. Rather, the primary objective is to evaluate broad spatial patterns in biodiversity documentation and observed richness across conservation-relevant areas using comparatively well-documented vertebrate taxa in the Philippines.

We now clarify this rationale in the revised Introduction. Amphibians and squamate reptiles were intentionally analyzed together because both groups represent some of the most comprehensively documented terrestrial vertebrates in the Philippines, with extensive specimen representation, taxonomic work, and digitally accessible occurrence data spanning more than a century. Their combined treatment therefore provides a suitable composite dataset for evaluating spatial structure in biodiversity records and conservation-relevant documentation gaps at a national scale. From Lines 93 –97, it reads:

“*As two of the most consistently documented vertebrate taxa in the country, herpetofauna provide a uniquely comprehensive dataset for assessing spatial documentation patterns. While acknowledging their distinct ecological niches, we integrate these groups as a singular, informative lens to evaluate biodiversity records across varying sampling landscapes*.”

To further address this concern, we revised the manuscript to avoid generalizing ecological interpretations across vertebrate groups and instead frame the results explicitly in terms of observed biodiversity documentation patterns.

**On specific comment in the manuscript about uneven sampling limiting the ability to compare observed richness between managed and unmanaged areas:**

We thank the reviewer for raising concerns regarding sampling bias, sampling completeness, and the interpretation of observed richness patterns. We agree that biodiversity occurrence data are inherently uneven and that observed richness does not directly represent true species richness. For this reason, our analytical framework explicitly incorporates sampling effort (occurrence density) as a covariate in all models, allowing us to account for variation in sampling effort when estimating relationships between species richness and environmental predictors. In addition, we tested whether sampling intensity differs systematically between PCA types, and found no strong evidence of baseline differences or PCA-type-dependent variation in sampling relationships. We also explicitly acknowledged in the revised manuscript that the number of occurrence points is an imperfect proxy for sampling effort and may reflect biological variation.

Importantly, our inference is explicitly restricted to observed species richness, and we have revised the manuscript to ensure that interpretations were tempered and do not conflate observed patterns with underlying ecological richness. In this context, variation in sampling completeness is not treated as a confounding error to be fully removed, but as an inherent property of biodiversity data that is directly modeled and explicitly quantified.

Regarding the suggestion that analyses should be restricted only to well-sampled units, we respectfully note that this would substantially reduce spatial coverage and disproportionately exclude precisely the under-sampled regions that are central to the objectives of this study. Instead, we adopt a modelling framework that retains all PCAs while accounting for sampling intensity continuously, which we believe is an appropriate approach in large-scale biodiversity syntheses where complete sampling equivalence is not achievable.

Finally, we have revised the Discussion to ensure clearer separation between (i) patterns in observed biodiversity data, (ii) potential ecological interpretation, and (iii) limitations imposed by uneven sampling coverage.
