# Supplementary material for: Sampling bias and uneven data coverage shape observed biodiversity patterns in a megadiverse island archipelago hotspot
Source: PLoS One. 2026 Jul 17;21(7):e0353393. doi: 10.1371/journal.pone.0353393 (PMC13379091; doi:10.1371/journal.pone.0353393)
Supplement: Peer review history

# Peer review: Decision Letter 0

**DOI:** 10.1371/journal.pone.0353393.r001

**Authors:** Juan Diaz-Ricaurte

16 Jan 2026

PONE-D-25-55790

Sampling bias obscures biodiversity patterns, reveals data gaps in priority conservation areas: a call for improved documentation

PLOS One

Dear Dr. Pitogo,

Thank you for submitting your manuscript to PLOS ONE. After careful consideration, we feel that it has merit but does not fully meet PLOS ONE’s publication criteria as it currently stands. Therefore, we invite you to submit a revised version of the manuscript that addresses the points raised during the review process.

Please submit your revised manuscript by Mar 02 2026 11:59PM. If you will need more time than this to complete your revisions, please reply to this message or contact the journal office at plosone@plos.org. When you're ready to submit your revision, log on to https://www.editorialmanager.com/pone/ and select the 'Submissions Needing Revision' folder to locate your manuscript file.

Please include the following items when submitting your revised manuscript:

- A letter that responds to each point raised by the academic editor and reviewer(s). You should upload this letter as a separate file labeled 'Response to Reviewers'.
- A marked-up copy of your manuscript that highlights changes made to the original version. You should upload this as a separate file labeled 'Revised Manuscript with Track Changes'.
- An unmarked version of your revised paper without tracked changes. You should upload this as a separate file labeled 'Manuscript'.

If you would like to make changes to your financial disclosure, please include your updated statement in your cover letter. Guidelines for resubmitting your figure files are available below the reviewer comments at the end of this letter.

If applicable, we recommend that you deposit your laboratory protocols in protocols.io to enhance the reproducibility of your results. Protocols.io assigns your protocol its own identifier (DOI) so that it can be cited independently in the future. For instructions see: https://journals.plos.org/plosone/s/submission-guidelines#loc-laboratory-protocols. Additionally, PLOS ONE offers an option for publishing peer-reviewed Lab Protocol articles, which describe protocols hosted on protocols.io. Read more information on sharing protocols at https://plos.org/protocols?utm_medium=editorial-email&utm_source=authorletters&utm_campaign=protocols.

Sincerely,

Juan C. Diaz-Ricaurte, PhD

Academic Editor

PLOS ONE

Journal requirements:

When submitting your revision, we need you to address these additional requirements.

1. Please ensure that your manuscript meets PLOS ONE's style requirements, including those for file naming. The PLOS ONE style templates can be found at

https://journals.plos.org/plosone/s/file?id=wjVg/PLOSOne_formatting_sample_main_body.pdf and

https://journals.plos.org/plosone/s/file?id=ba62/PLOSOne_formatting_sample_title_authors_affiliations.pdf

2. Thank you for stating the following in the Acknowledgments Section of your manuscript:

“During the preparation of this manuscript, KMEP was partially supported by a Graduate Research Assistantship from the University of Kansas Department of Ecology and Evolutionary Biology; and RMB was supported by a Fulbright International Scholar Award administered by the U.S. Department of State, the Philippine-American Education Foundation (PAEF) and the Philippine Council for International Exchange of Scholars. Support for fieldwork that generated many specimen-associated records was provided by the U.S. National Science Foundation (DEB 073199, 0743491, 1418895, 0910341, 0804115, 1654388, and 0334952 and EF-0334952 to RMB and KU-based students), the KU College of Liberal Arts and Sciences Graduate Assistantship in Support of Faculty Research Award, a KU Biodiversity Institute Research and Exploration Fund grant, the South, Southeast, and East Asia Fund of the KU Office of International Affairs, and the General Research Fund of the University of Kansas.”

We note that you have provided funding information that is not currently declared in your Funding Statement. However, funding information should not appear in the Acknowledgments section or other areas of your manuscript. We will only publish funding information present in the Funding Statement section of the online submission form.

Please remove any funding-related text from the manuscript and let us know how you would like to update your Funding Statement. Currently, your Funding Statement reads as follows:

“The author(s) received no specific funding for this work.”

Please include your amended statements within your cover letter; we will change the online submission form on your behalf.

3. Thank you for stating the following financial disclosure:

“During the preparation of this manuscript, KMEP was partially supported by a Graduate Research Assistantship from the University of Kansas Department of Ecology and Evolutionary Biology; and RMB was supported by a Fulbright International Scholar Award administered by the U.S. Department of State, the Philippine-American Education Foundation (PAEF) and the Philippine Council for International Exchange of Scholars. Support for fieldwork that generated many specimen-associated records was provided by the U.S. National Science Foundation (DEB 073199, 0743491, 1418895, 0910341, 0804115, 1654388, and 0334952 and EF-0334952 to RMB and KU-based students), the KU College of Liberal Arts and Sciences Graduate Assistantship in Support of Faculty Research Award, a KU Biodiversity Institute Research and Exploration Fund grant, the South, Southeast, and East Asia Fund of the KU Office of International Affairs, and the General Research Fund of the University of Kansas.”

At this time, please address the following queries:

a) Please clarify the sources of funding (financial or material support) for your study. List the grants or organizations that supported your study, including funding received from your institution.

b) State what role the funders took in the study. If the funders had no role in your study, please state: “The funders had no role in study design, data collection and analysis, decision to publish, or preparation of the manuscript.”

c) If any authors received a salary from any of your funders, please state which authors and which funders.

d) If you did not receive any funding for this study, please state: “The authors received no specific funding for this work.”

4. Thank you for stating in your Funding Statement:

“During the preparation of this manuscript, KMEP was partially supported by a Graduate Research Assistantship from the University of Kansas Department of Ecology and Evolutionary Biology”

Please provide an amended statement that declares *all* the funding or sources of support (whether external or internal to your organization) received during this study, as detailed online in our guide for authors at http://journals.plos.org/plosone/s/submit-now.  Please also include the statement “There was no additional external funding received for this study.” in your updated Funding Statement.

Please include your amended Funding Statement within your cover letter. We will change the online submission form on your behalf.

5. We note that Figures 1, 2 and S5 in your submission contain map images which may be copyrighted. All PLOS content is published under the Creative Commons Attribution License (CC BY 4.0), which means that the manuscript, images, and Supporting Information files will be freely available online, and any third party is permitted to access, download, copy, distribute, and use these materials in any way, even commercially, with proper attribution. For these reasons, we cannot publish previously copyrighted maps or satellite images created using proprietary data, such as Google software (Google Maps, Street View, and Earth). For more information, see our copyright guidelines: http://journals.plos.org/plosone/s/licenses-and-copyright.

We require you to either (1) present written permission from the copyright holder to publish these figures specifically under the CC BY 4.0 license, or (2) remove the figures from your submission:

1. You may seek permission from the original copyright holder of Figure(s) [#] to publish the content specifically under the CC BY 4.0 license.

We recommend that you contact the original copyright holder with the Content Permission Form (http://journals.plos.org/plosone/s/file?id=7c09/content-permission-form.pdf) and the following text:

“I request permission for the open-access journal PLOS ONE to publish XXX under the Creative Commons Attribution License (CCAL) CC BY 4.0 (http://creativecommons.org/licenses/by/4.0/). Please be aware that this license allows unrestricted use and distribution, even commercially, by third parties. Please reply and provide explicit written permission to publish XXX under a CC BY license and complete the attached form.”

Please upload the completed Content Permission Form or other proof of granted permissions as an "Other" file with your submission.

In the figure caption of the copyrighted figure, please include the following text: “Reprinted from [ref] under a CC BY license, with permission from [name of publisher], original copyright [original copyright year].”

2. If you are unable to obtain permission from the original copyright holder to publish these figures under the CC BY 4.0 license or if the copyright holder’s requirements are incompatible with the CC BY 4.0 license, please either i) remove the figure or ii) supply a replacement figure that complies with the CC BY 4.0 license. Please check copyright information on all replacement figures and update the figure caption with source information. If applicable, please specify in the figure caption text when a figure is similar but not identical to the original image and is therefore for illustrative purposes only.

The following resources for replacing copyrighted map figures may be helpful:

USGS National Map Viewer (public domain): http://viewer.nationalmap.gov/viewer/

The Gateway to Astronaut Photography of Earth (public domain): http://eol.jsc.nasa.gov/sseop/clickmap/

Maps at the CIA (public domain): https://www.cia.gov/library/publications/the-world-factbook/index.html and https://www.cia.gov/library/publications/cia-maps-publications/index.html

NASA Earth Observatory (public domain): http://earthobservatory.nasa.gov/

Landsat: http://landsat.visibleearth.nasa.gov/

USGS EROS (Earth Resources Observatory and Science (EROS) Center) (public domain): http://eros.usgs.gov/#

Natural Earth (public domain): http://www.naturalearthdata.com/

Additional Editor Comments:

Decision: Major Revision

Manuscript: Sampling bias obscures biodiversity patterns, reveals data gaps in priority conservation areas: a call for improved documentation

Dear Dr. Pitogo,

I have now received two reviewer comments on your manuscript. Both reviewers recognize the study as well written, timely, and highly relevant for biodiversity research and conservation planning. The reviewers and I agree that the manuscript addresses an important and well-defined problem, and that the assembled dataset represents a valuable contribution. However, in its current form, the manuscript cannot be considered for publication.

Both reviewers identify a number of analytical and methodological issues that are central to the study’s main conclusions. I agree with the reviewers that these issues must be addressed before the manuscript can be evaluated further. Below, I synthesize their comments, indicating where each reviewer places particular emphasis, and outline the key expectations for revision.

A first overarching issue, raised primarily by Reviewer 1, concerns the alignment between research questions and analytical choices. Although the research questions are clearly stated, the links between each question, the statistical models used, and the corresponding results are not always explicit. I strongly suggest that the revised manuscript make these connections clear and systematic. Each main question should be directly associated with the analysis used to address it and with the relevant results, as this alignment is essential for transparency and interpretability.

A major methodological concern, discussed in detail by Reviewer 1 and reinforced by Reviewer 2, relates to the framework used to compare different categories of conservation areas. Much of the manuscript’s inference depends on comparing sampling effort and diversity patterns across conservation area types, yet the current analytical approach does not allow formal statistical testing of differences among these categories. I agree with both reviewers that this represents a substantive limitation. For the revision to be satisfactory, these comparisons should be conducted within a unified modeling framework that allows direct statistical evaluation of differences in effects across conservation area types.

Closely linked to this issue, Reviewer 1 emphasizes the importance of retaining model components that are justified by the study design, particularly grouping or hierarchical variables included to account for non-independence among observations. I concur with this point. Structural terms that reflect the sampling design should not be removed based solely on variance explained or model fit. I therefore recommend revisiting the model structure to ensure that non-independence is appropriately controlled, as this is critical for the validity of the inferences.

The spatial properties of the dataset constitute another key issue raised by both reviewers from complementary perspectives. While reviewer 1 focuses on spatial precision, including how coordinate uncertainty was handled and whether the chosen grid resolution is appropriate given the characteristics of the occurrence records, the reviewer 2 emphasizes the need to explicitly address spatial dependence among observations. Given the spatially explicit nature of the analyses, I agree that these issues cannot be treated as secondary. The revised manuscript should clearly justify the analytical scale, address positional uncertainty, and either incorporate spatial structure into the models or provide a clear and defensible rationale for not doing so.

Both reviewers also identify limitations in the biological interpretation of the results, particularly regarding the joint treatment of amphibians and reptiles. These groups differ in ecology, detectability, sampling intensity, and conservation context. I suggest that the authors either explicitly assess whether these taxa exhibit similar or contrasting patterns, or clearly justify their combined analysis. Without this clarification, the ecological interpretation of the results remains incomplete.

Further clarification is also required regarding the conceptual framing of conservation areas. Reviewer 2 raises questions about how different conservation categories are defined and compared, while Reviewer 1 highlights the difficulty of interpreting area-based comparisons under strong and uneven sampling biases. I agree with both assessments and recommend that the revised manuscript explicitly state the assumptions underlying these comparisons and clearly articulate how results should be interpreted in light of known data limitations.

Finally, both reviewers note that several figures (particularly those illustrating spatial patterns) are difficult to interpret due to low resolution and small text. This may be related to how figures are displayed in the submission system; however, I recommend you include all figures in the revised version at the highest resolution possible to facilitate careful evaluation by the reviewers. Thank you in advance for their attention to this point.

In light of these points, my editorial decision is Major Revision. Addressing them will require substantive re-analysis and careful revision of both methods and interpretation. Nevertheless, the reviewers and I agree that a thoroughly revised manuscript has strong potential, and we encourage you to engage fully with these comments as you prepare your revision.

We look forward to receiving a revised version of your manuscript.

Sincerely,

Juan C. Diaz-Ricaurte, PhD

Academic Editor

PLOS ONE

[Note: HTML markup is below. Please do not edit.]

Reviewers' comments:

Reviewer's Responses to Questions

**Comments to the Author**

1. Is the manuscript technically sound, and do the data support the conclusions?

The manuscript must describe a technically sound piece of scientific research with data that supports the conclusions. Experiments must have been conducted rigorously, with appropriate controls, replication, and sample sizes. The conclusions must be drawn appropriately based on the data presented.

Reviewer #1: Yes

Reviewer #2: Yes

**********

2. Has the statistical analysis been performed appropriately and rigorously?

Reviewer #1: I Don't Know

Reviewer #2: No

**********

3. Have the authors made all data underlying the findings in their manuscript fully available?

The PLOS Data policy requires authors to make all data underlying the findings described in their manuscript fully available without restriction, with rare exception (please refer to the Data Availability Statement in the manuscript PDF file). The data should be provided as part of the manuscript or its supporting information, or deposited to a public repository. For example, in addition to summary statistics, the data points behind means, medians and variance measures should be available. If there are restrictions on publicly sharing data—e.g. participant privacy or use of data from a third party—those must be specified.

Reviewer #1: Yes

Reviewer #2: Yes

**********

4. Is the manuscript presented in an intelligible fashion and written in standard English?

PLOS ONE does not copyedit accepted manuscripts, so the language in submitted articles must be clear, correct, and unambiguous. Any typographical or grammatical errors should be corrected at revision, so please note any specific errors here.

Reviewer #1: Yes

Reviewer #2: Yes

**********

5. Review Comments to the Author

Please use the space provided to explain your answers to the questions above. You may also include additional comments for the author, including concerns about dual publication, research ethics, or publication ethics. (Please upload your review as an attachment if it exceeds 20,000 characters)

Reviewer #1: Dear Authors,

The study is well written and provides a comprehensive overview of the state of herpetofaunal knowledge in the Philippines, addressing important questions for species and territorial conservation.

The manuscript investigates a critical challenge in conservation biology: how sampling biases affect our understanding of biodiversity patterns, particularly within priority conservation areas (PCAs) in the Philippines.

This central question is clearly articulated through four specific inquiries:

1. How is observed herpetofaunal diversity (amphibians and squamate reptiles) spatially distributed across the Philippines?

2. How do these patterns differ across datasets from museum collections, citizen-science platforms, and peer-reviewed literature?

3. How well do observed diversity patterns coincide with priority conservation areas (PCAs)?

4. Which PCAs, and which types, are more thoroughly sampled?

To address these questions, a data-driven spatial analysis approach was adopted, compiling an impressive dataset of occurrence records from museum collections, citizen science, and literature. You then apply grid-based mapping, statistical modeling, and model selection procedures to explore species richness and sampling effort.

The overall approach is adequate. However, I would suggest that the authors more explicitly connect each research question to the specific model(s) used. Clarifying which models and results correspond to which of the four main questions would enhance the transparency and coherence of the study design.

Beyond this general comment, my primary concerns are methodological, and I believe addressing them will strengthen the statistical rigor and interpretability of this important work.

Thank you for the detailed description of the data curation procedures. All the steps were important to guarantee the quality of the input data. However, I would like to raise a question regarding the spatial precision of the occurrence records used.

While the manuscript mentions the exclusion of records outside known distributions and the removal of generic coordinates, I did not find any reference to the use of the “coordinate uncertainty in meters” field (available in GBIF records) or a similar assessment of the dataset's spatial accuracy. Given that the study employs a 10-km grid resolution, I wonder whether this scale is appropriate considering the potential variability in coordinate precision, especially for historical museum records which can lack fine-scale georeferencing.

Could the authors clarify whether any filtering based on coordinate uncertainty was applied? If not, do the authors consider the 10-km resolution robust to such imprecision, and how might this potentially affect the spatial allocation of records and the resulting diversity patterns?

The current analysis utilizes Negative Binomial GLMs fitted separately for Protected Areas (PAs) and Key Biodiversity Areas (KBAs). The methodological rationale for this decision is not fully clear in the manuscript. If this separation was a deliberate choice to address, for example, severe variance heterogeneity, it would be very helpful for the reader if this justification were made explicit.

My main concern with this approach is that a central objective of the study appears to be the comparison of predictor effects (e.g., area, topographic relief), and the comparison of richness and effort between PAs and KBAs. Adjusting separate models, while legitimate for describing the relationships within each group, impedes a formal statistical comparison of the coefficient estimates.

To formally test the hypothesis that a predictor's effect differs between PAs and KBAs, the standard and more robust statistical approach would be to analyze both groups within a single, unified model. This is typically achieved by including the group affiliation (PA vs. KBA) as a categorical predictor and, crucially, testing interaction terms between this group variable and the predictors of interest (e.g., PCA_Type * Area). The p-value for the interaction term provides the formal statistical test of the null hypothesis that there is no difference in the slopes between the groups.

It is plausible that adopting this unified model approach would yield substantive results similar to those already presented. However, its key advantage would be to make the comparative claims—which are central to the study's objectives—statistically explicit and defensible, thereby increasing the analysis's robustness and interpretive clarity.

My final methodological point relates to the model selection for control effects. The authors state that the random effect variable "PAIC" was removed from one of the models because it "explained less than 1% of the variance and did not improve model performance.” (Line 234).

A low number of levels (seven, in this case) can lead to imprecise variance estimates. However, the decision to include or exclude a structural random effect should be based on the study design, not on its statistical performance (such as variance explained or AIC).

As the 'PAIC' variable represents a grouping variable (e.g., island/region) dictated by the sampling design, its function in the model is not primarily to "explain variance" (as in a predictive model) , but rather to control for the non-independence of the data (i.e., pseudoreplication). Observations from the same PAIC group are likely to be more similar to each other, violating the assumption of independence. The literature strongly advocates for retaining the random effects structure justified by the design. Removing this structural term, even if its variance is low, can lead to "overoptimistic" results, such as artificially reduced standard errors and an inflated Type I error rate. (Barr, D. J., Levy, R., Scheepers, C., & Tily, H. J. (2013). Random effects structure for confirmatory hypothesis testing: Keep it maximal. Journal of Memory and Language, 68(3), 255-278. https://doi.org/10.1016/j.jml.2012.11.001)

I would, therefore, recommend that the authors retain the 'PAIC' random effect in the final models to ensure that potential non-independence in the data is appropriately controlled for, thereby strengthening the validity of the model's inferences.

The study is situated within a well-established body of literature that highlights the challenges posed by sampling bias in biodiversity data and its implications for conservation planning. By focusing on the Philippines—a recognized biodiversity hotspot—the authors contribute valuable insights to this broader discourse. Their integration of museum records, citizen science, and literature-based data reflects current best practices in biodiversity informatics.

The study's main findings and conclusions are clear and relevant. You found that biodiversity data is unevenly distributed, with most records concentrated in a few well-studied areas. A key conclusion is that many PCAs, especially in mountainous regions, lack sufficient documentation. Furthermore, the analysis suggests that some of the most species-rich areas may lie outside current PCAs, highlighting a potential need to expand conservation boundaries. I believe this discussion could be further strengthened by evidence following the adjustments to the model selection steps, as suggested above. The authors then conclude that integrating diverse data sources and improving documentation practices are essential to address these critical data gaps and enhance future conservation planning.

Regarding the presentation of these results, I found many of the figures, both in the supplementary material and - more significantly - in the main manuscript, difficult to interpret: the resolution appears to be low, and the font size for labels and legends is very small. I would recommend the authors provide higher-resolution figures with more legible text to ensure readers can fully interpret the study’s important spatial results.

Thank you for the opportunity to review this manuscript. I truly enjoyed reading it and found the research to be a very valuable contribution to conservation. I hope my comments are clear and provide a constructive path for improving the analysis and impact of this important work.

Kind Regards

Reviewer #2: Dear Authors

The manuscript is well-written, and the findings are important contributions to conservation planning in the Philippines. Nevertheless, there are some drawbacks in the MS. 1. There is confusion about conservation based on area figures, requiring justification for why to analyze KBA separately from OECM. 2. Models don´t account for spatial correlation, and since you’re using georeferenced records, it´s necessary to include the effect of dependence between observations. 3 The model relating species diversity to potential biases from topography and conservation area size is not appropriate, given the acknowledged data biases. 4. It’s not clear whether amphibians follow similar patterns of reptile species records. Both classes have differences in distribution, sampling, conservation and research effort. Therefore, presenting data analysis that discriminates between the two classes would be helpful to unveil shortfalls at a finer resolution.

**********

6. PLOS authors have the option to publish the peer review history of their article (what does this mean?). If published, this will include your full peer review and any attached files.

If you choose “no”, your identity will remain anonymous but your review may still be made public.

**Do you want your identity to be public for this peer review?**  For information about this choice, including consent withdrawal, please see our Privacy Policy.

Reviewer #1: No

Reviewer #2: No

**********

[NOTE: If reviewer comments were submitted as an attachment file, they will be attached to this email and accessible via the submission site. Please log into your account, locate the manuscript record, and check for the action link "View Attachments". If this link does not appear, there are no attachment files.]

To ensure your figures meet our technical requirements, please review our figure guidelines: https://journals.plos.org/plosone/s/figures

You may also use PLOS’s free figure tool, NAAS, to help you prepare publication quality figures: https://journals.plos.org/plosone/s/figures#loc-tools-for-figure-preparation.

NAAS will assess whether your figures meet our technical requirements by comparing each figure against our figure specifications.

Attachment Submitted filename: PONE-D-25-55790.pdf

# Author comment: Author response to Decision Letter 1

**DOI:** 10.1371/journal.pone.0353393.r002

20 Mar 2026

EDITOR

Editor: A first overarching issue, raised primarily by Reviewer 1, concerns the alignment between research questions and analytical choices. Although the research questions are clearly stated, the links between each question, the statistical models used, and the corresponding results are not always explicit. I strongly suggest that the revised manuscript make these connections clear and systematic. Each main question should be directly associated with the analysis used to address it and with the relevant results, as this alignment is essential for transparency and interpretability.

Response: We have revised the phrasing of Questions 3 and 4 to better align with the analyses conducted. In the Methods section, we now state each specific objective before describing the corresponding analytical approach to clarify the connection between the questions and the analyses. The Results section also follow the same structure.

Editor: A major methodological concern, discussed in detail by Reviewer 1 and reinforced by Reviewer 2, relates to the framework used to compare different categories of conservation areas. Much of the manuscript’s inference depends on comparing sampling effort and diversity patterns across conservation area types, yet the current analytical approach does not allow formal statistical testing of differences among these categories. I agree with both reviewers that this represents a substantive limitation. For the revision to be satisfactory, these comparisons should be conducted within a unified modeling framework that allows direct statistical evaluation of differences in effects across conservation area types.

Response: We reanalyzed the dataset using a joint modeling framework in which species richness was modeled as a function of area, topographic relief, and occurrence density, including all pairwise interactions among these predictors and their interactions with PCA type (protected and conserved areas vs. key biodiversity areas). Biogeographic subregion was included as a random intercept to account for non-independence among PCAs due to shared biogeographic structure. Results were broadly consistent with our previous approach. However, we retain this unified model as it more directly addresses our specific questions, as recommended by the reviewers as well.

Editor: Reviewer 1 emphasizes the importance of retaining model components that are justified by the study design, particularly grouping or hierarchical variables included to account for non-independence among observations.

Response: In our new models, we retained the biogeographic subregion (Pleistocene Aggregate Island Complexes or PAIC) as a random effect. Variance explained for the random effect are also reported in the results section, alongside the coefficient estimates of the fixed predictors.

Editor: The spatial properties of the dataset constitute another key issue raised by both reviewers from complementary perspectives. While reviewer 1 focuses on spatial precision, including how coordinate uncertainty was handled and whether the chosen grid resolution is appropriate given the characteristics of the occurrence records, the reviewer 2 emphasizes the need to explicitly address spatial dependence among observations. Given the spatially explicit nature of the analyses, I agree that these issues cannot be treated as secondary. The revised manuscript should clearly justify the analytical scale, address positional uncertainty, and either incorporate spatial structure into the models or provide a clear and defensible rationale for not doing so.

Response: We thank both Reviewers for highlighting the inherent challenges of spatially explicit analyses. In response to Reviewer 1, we have clarified in the revised manuscript our rationale for the selected 10-km grid resolution (lines 200–204) and our treatment of coordinate uncertainty (lines 184–190). The choice of a 10-km grid resolution was deliberate and intended to provide an appropriate balance for visualizing broad-scale spatial patterns of observed species richness patterns across the Philippines.

Regarding spatial uncertainty, we retained coordinates that were consistent with general (provincial-level) locality information, as these adequately reflect broad geographic distribution patterns (country-wide assessment). Our sampling is broad enough and has a level of certainty where broad patterns will be discovered. Although minor spatial imprecision may influence species richness estimates within individual PCAs used in the modeling, any resulting bias is expected to be minimal because most PCAs are supported by numerous occurrence records, species are represented by multiple points within PCAs due to the integration of multiple data sources, and the overall sample size is large (n = 316 PCAs).

Editor: Both reviewers also identify limitations in the biological interpretation of the results, particularly regarding the joint treatment of amphibians and reptiles. These groups differ in ecology, detectability, sampling intensity, and conservation context. I suggest that the authors either explicitly assess whether these taxa exhibit similar or contrasting patterns, or clearly justify their combined analysis. Without this clarification, the ecological interpretation of the results remains incomplete.

Response: We acknowledge that amphibians and reptiles differ in their ecology and diversity patterns. However, as stated in the Introduction, these groups were used in this study because, relative to other taxonomic groups in the Philippines, they are comparatively well-documented and thus provide a robust basis for assessing observed biodiversity patterns and sampling distribution across areas. Our objective was not to model amphibian and reptile diversity separately or to compare their biological drivers (e.g., fine-scale environmental variables), but rather to evaluate how observed species richness patterns relate to area, sampling effort, and topographic relief in the context of biodiversity documentation or conservation planning. Accordingly, our interpretation focuses on spatial patterns of documented diversity rather than taxon-specific ecological differences. In addition, amphibians and reptiles have historically and contemporarily been surveyed and analyzed jointly in the Philippines (see Meneses et al. 2024), making their combined treatment, at least within the context of this paper, consistent with established research practice.

Editor: Further clarification is also required regarding the conceptual framing of conservation areas. Reviewer 2 raises questions about how different conservation categories are defined and compared, while Reviewer 1 highlights the difficulty of interpreting area-based comparisons under strong and uneven sampling biases. I agree with both assessments and recommend that the revised manuscript explicitly state the assumptions underlying these comparisons and clearly articulate how results should be interpreted in light of known data limitations.

Response: We recognize that the conceptual framing and terminology of conservation areas required consistency in the original submission. Following the recommendation of the Reviewer, we adopted the lexicon proposed by Salafsky et al. (2024), distinguishing between protected and conserved areas (i.e., areas with formal management or governance) and key biodiversity areas (i.e., spatial prioritization tools without inherent governance or protection status). In the revised manuscript, we refer to both collectively as Philippine conservation-relevant areas (PCAs) to clarify the scope of our comparisons and to avoid confusion with the previous use of “priority conservation areas.” We limit acronym usage to PCA, while “protected and conserved areas” and “key biodiversity areas” are written out in full throughout the text for clarity and consistency.

Regarding concerns about interpretation under uneven sampling effort, we model observed species richness as a function of area, topographic relief, and occurrence density (as a proxy for sampling effort), with biogeographic subregion included as a random intercept to account for geographic clustering. This framework allows us to assess whether differences in observed richness among PCA types persist after accounting for variation in sampling intensity (and other predictors) and shared biogeographic structure. In addition, we modeled occurrence points as a function of area and topographic relief, again including biogeographic subregion as a random effect, to evaluate whether sampling effort itself varies systematically between PCA types (which we found no sufficient evidence). Finally, we clarify in the Discussion that our results reflect documented (observed) diversity patterns and should be interpreted in light of known data limitations.

Editor: Both reviewers note that several figures (particularly those illustrating spatial patterns) are difficult to interpret due to low resolution and small text. This may be related to how figures are displayed in the submission system; however, I recommend you include all figures in the revised version at the highest resolution possible to facilitate careful evaluation by the reviewers.

Response: We will upload the highest-resolution version permitted by the submission system. The original figures were generated at high resolution; however, the conversion to PDF within the system may have reduced their clarity.

REVIEWER 1

Reviewer: I would suggest that the authors more explicitly connect each research question to the specific model(s) used. Clarifying which models and results correspond to which of the four main questions would enhance the transparency and coherence of the study design.

Response: We thank the Reviewer for raising this clarification. In the revised methods section, we now state each specific objective before describing the corresponding analytical approach to clarify the connection between the questions and the analyses. The Results section also follow the same structure.

Reviewer: Thank you for the detailed description of the data curation procedures. All the steps were important to guarantee the quality of the input data. However, I would like to raise a question regarding the spatial precision of the occurrence records used.

While the manuscript mentions the exclusion of records outside known distributions and the removal of generic coordinates, I did not find any reference to the use of the “coordinate uncertainty in meters” field (available in GBIF records) or a similar assessment of the dataset's spatial accuracy. Given that the study employs a 10-km grid resolution, I wonder whether this scale is appropriate considering the potential variability in coordinate precision, especially for historical museum records which can lack fine-scale georeferencing.

Could the authors clarify whether any filtering based on coordinate uncertainty was applied? If not, do the authors consider the 10-km resolution robust to such imprecision, and how might this potentially affect the spatial allocation of records and the resulting diversity patterns?

Response: We thank the reviewer for highlighting the issue of spatial precision. Because relatively few occurrence records include explicit uncertainty, we did not filter based on this metric. Instead, we retained records with geographic coordinates that correspond to general geographic location (provincial level). All records broadly reflect geographic patterns of each species distribution, and any minor imprecision is expected to have minimal impact on broad-scale, country-wide patterns. As stated above, although minor spatial imprecision may influence species richness estimates within individual PCAs used in the modeling, any resulting bias is expected to be minimal because most PCAs are supported by numerous occurrence records, species are represented by multiple points within PCAs due to the integration of multiple data sources, and the overall sample size is large (n = 316 PCAs).

The 10-km grid resolution was also deliberately chosen to visualize broad-scale distribution patterns across the Philippines. This scale provides an appropriate balance: smaller grids (e.g., <10 km) would be too fragmented on larger islands, while larger grids (e.g., 15–20 km) could average out meaningful spatial variation, particularly on smaller islands. We have included this justification in the revised manuscript (Lines 200–204).

Reviewer: The current analysis utilizes Negative Binomial GLMs fitted separately for Protected Areas (PAs) and Key Biodiversity Areas (KBAs). The methodological rationale for this decision is not fully clear in the manuscript. If this separation was a deliberate choice to address, for example, severe variance heterogeneity, it would be very helpful for the reader if this justification were made explicit.

My main concern with this approach is that a central objective of the study appears to be the comparison of predictor effects (e.g., area, topographic relief), and the comparison of richness and effort between PAs and KBAs. Adjusting separate models, while legitimate for describing the relationships within each group, impedes a formal statistical comparison of the coefficient estimates.

To formally test the hypothesis that a predictor's effect differs between PAs and KBAs, the standard and more robust statistical approach would be to analyze both groups within a single, unified model. This is typically achieved by including the group affiliation (PA vs. KBA) as a categorical predictor and, crucially, testing interaction terms between this group variable and the predictors of interest (e.g., PCA_Type * Area). The p-value for the interaction term provides the formal statistical test of the null hypothesis that there is no difference in the slopes between the groups.

It is plausible that adopting this unified model approach would yield substantive results similar to those already presented. However, its key advantage would be to make the comparative claims—which are central to the study's objectives—statistically explicit and defensible, thereby increasing the analysis's robustness and interpretive clarity.

Response: We thank the Reviewer for this thoughtful and constructive feedback. In the revised manuscript, we reanalyzed the dataset using a joint modeling framework in which species richness was modeled as a function of area, topographic relief, and occurrence density, including all pairwise interactions among these predictors and their interactions with PCA type. Biogeographic subregion was included as a random intercept to account for non-independence among PCAs due to shared biogeographic structure. Although the results are broadly consistent with our previous approach, as the Reviewer noted, this framework allows for a more direct comparison between PCA types and highlights additional patterns for discussion. Hence, we kept this unified modelling framework. We have revised the Methods (lines 228–317), Results (lines 401–465), and Discussion (lines 522–578) sections accordingly.

Reviewer: My final methodological point relates to the model selection for control effects. The authors state that the random effect variable "PAIC" was removed from one of the models because it "explained less than 1% of the variance and did not improve model performance.” (Line 234).

A low number of levels (seven, in this case) can lead to imprecise variance estimates. However, the decision to include or exclude a structural random effect should be based on the study design, not on its statistical performance (such as variance explained or AIC).

As the 'PAIC' variable represents a grouping variable (e.g., island/region) dictated by the sampling design, its function in the model is not primarily to "explain variance" (as in a predictive model) , but rather to control for the non-independence of the data (i.e., pseudoreplication). Observations from the same PAIC group are likely to be more similar to each other, violating the assumption of independence. The literature strongly advocates for retaining the random effects structure justified by the design. Removing this structural term, even if its variance is low, can lead to "overoptimistic" results, such as artificiall

Attachment Submitted filename: Response to Reviewers.docx

# Peer review: Decision Letter 1

**DOI:** 10.1371/journal.pone.0353393.r003

**Authors:** Juan Diaz-Ricaurte

12 May 2026

PONE-D-25-55790R1

Sampling bias obscures biodiversity patterns, reveals data gaps in a megadiverse island archipelago hotspot

PLOS One

Dear Dr. Pitogo,

Thank you for submitting your manuscript to PLOS ONE. After careful consideration, we feel that it has merit but does not fully meet PLOS ONE’s publication criteria as it currently stands. Therefore, we invite you to submit a revised version of the manuscript that addresses the points raised during the review process.

Thank you for submitting the revised version of your manuscript. I have now received the reviewers’ comments and have carefully considered them together with the revised manuscript.

First, I would like to acknowledge the work that has gone into this revision. Both reviewers recognize that the manuscript is clearer and that several of the previous concerns have been addressed. I also think that the study has considerable potential. The topic is relevant, the dataset is valuable, and the manuscript addresses questions that are important for understanding biodiversity patterns and sampling limitations.

However, there are still some important issues that need to be resolved before the manuscript can be considered further. For this reason, my decision is to invite a further revision.

At this stage, I would like to be very clear about the main editorial concern. The remaining issues are not simply matters of wording or presentation. They affect how the results are interpreted and how strongly the manuscript can support its conclusions. In particular, the reviewers continue to raise concerns about the treatment of sampling bias, the structure of the statistical models, spatial dependence, and the interpretation of observed richness patterns.

Please pay particular attention to the comments from Reviewer 1. Their points regarding model complexity, the use of occurrence records as a proxy for sampling effort, and the apparent inconsistency in the interpretation of sampling bias are especially important. These issues are central to the manuscript’s argument and should be addressed directly in the revised version.

In particular, the revised manuscript should clarify whether the current model structure is the most appropriate one for the questions being asked. The inclusion of several variables and interaction terms may make it difficult to identify the main patterns and may obscure the role of unequal sampling effort, which is one of the key issues the manuscript aims to address. If the authors retain the current model structure, a clearer justification is needed. Alternatively, some form of model simplification, model comparison, or sensitivity analysis may help make the results easier to interpret.

The use of total occurrence records as a proxy for sampling effort also needs to be treated with more caution. As noted by the reviewers, this variable may partly reflect observer effort, but it may also reflect true biological abundance or detectability. This creates a potential circularity that should either be addressed analytically, if possible, or discussed more explicitly as a limitation. The interpretation of the results should be adjusted accordingly.

There is also an apparent inconsistency in the way the manuscript discusses sampling bias. In some sections, the text suggests that sampling effort does not drive the observed patterns, whereas in others it suggests that spatial sampling bias is a major driver of observed richness. This point should be resolved carefully, because it is central to the manuscript. The revised version should present a clear and internally consistent interpretation of what the analyses can and cannot show.

I also ask the authors to address Reviewer 2’s concerns regarding spatial dependence and the combined analysis of amphibians and reptiles. If spatial autocorrelation cannot be explicitly modeled or tested, this should be clearly justified and incorporated into the limitations of the study. Similarly, the decision to analyze amphibians and reptiles within a single framework should be better justified, or the interpretation should be made more cautious and, where appropriate, group-specific.

Finally, please revise the Discussion to make it more focused and less repetitive. The manuscript would benefit from a clearer distinction between ecological signal, sampling artifacts, and the limitations imposed by the available data. Please also improve figure readability, especially the font size, as noted by Reviewer 1.

Because this is already a further round of revision, I strongly encourage the authors to address the reviewers’ comments as fully and carefully as possible. From an editorial perspective, the next version should allow a clear final assessment of whether the main concerns have been resolved. In that sense, this may be the last opportunity for substantive revision before a final decision is made.

I remain interested in the manuscript and believe it could make a valuable contribution if these issues are addressed convincingly. Please submit a detailed response letter explaining how each point raised by the reviewers has been handled. Where you decide not to make a suggested change, please provide a clear and specific justification.

I look forward to receiving your revised manuscript.

Kind regards,

Juan C. Diaz-Ricaurte, Ph.D

Academic Editor

PLOS One

Please submit your revised manuscript by Jun 26 2026 11:59PM. If you will need more time than this to complete your revisions, please reply to this message or contact the journal office at plosone@plos.org. When you're ready to submit your revision, log on to https://www.editorialmanager.com/pone/ and select the 'Submissions Needing Revision' folder to locate your manuscript file.

Please include the following items when submitting your revised manuscript:

- A letter that responds to each point raised by the academic editor and reviewer(s). You should upload this letter as a separate file labeled 'Response to Reviewers'.
- A marked-up copy of your manuscript that highlights changes made to the original version. You should upload this as a separate file labeled 'Revised Manuscript with Track Changes'.
- An unmarked version of your revised paper without tracked changes. You should upload this as a separate file labeled 'Manuscript'.

If you would like to make changes to your financial disclosure, please include your updated statement in your cover letter. Guidelines for resubmitting your figure files are available below the reviewer comments at the end of this letter.

If applicable, we recommend that you deposit your laboratory protocols in protocols.io to enhance the reproducibility of your results. Protocols.io assigns your protocol its own identifier (DOI) so that it can be cited independently in the future. For instructions see: https://journals.plos.org/plosone/s/submission-guidelines#loc-laboratory-protocols. Additionally, PLOS ONE offers an option for publishing peer-reviewed Lab Protocol articles, which describe protocols hosted on protocols.io. Read more information on sharing protocols at https://plos.org/protocols?utm_medium=editorial-email&utm_source=authorletters&utm_campaign=protocols.

As the corresponding author, your ORCID iD is verified in the submission system and will appear in the published article. PLOS supports the use of ORCID, and we encourage all coauthors to register for an ORCID iD and use it as well. Please encourage your coauthors to verify their ORCID iD within the submission system before final acceptance, as unverified ORCID iDs will not appear in the published article. *Only*  the individual author can complete the verification step; PLOS staff *cannot*  verify ORCID iDs on behalf of authors.

Journal Requirements:

If the reviewer comments include a recommendation to cite specific previously published works, please review and evaluate these publications to determine whether they are relevant and should be cited. There is no requirement to cite these works unless the editor has indicated otherwise.

[Note: HTML markup is below. Please do not edit.]

Reviewers' comments:

Reviewer's Responses to Questions

**Comments to the Author**

1. If the authors have adequately addressed your comments raised in a previous round of review and you feel that this manuscript is now acceptable for publication, you may indicate that here to bypass the “Comments to the Author” section, enter your conflict of interest statement in the “Confidential to Editor” section, and submit your "Accept" recommendation.

Reviewer #1: All comments have been addressed

Reviewer #2: (No Response)

**********

2. Is the manuscript technically sound, and do the data support the conclusions?

The manuscript must describe a technically sound piece of scientific research with data that supports the conclusions. Experiments must have been conducted rigorously, with appropriate controls, replication, and sample sizes. The conclusions must be drawn appropriately based on the data presented.

Reviewer #1: Partly

Reviewer #2: Partly

**********

3. Has the statistical analysis been performed appropriately and rigorously?

Reviewer #1: I Don't Know

Reviewer #2: No

**********

4. Have the authors made all data underlying the findings in their manuscript fully available?

The PLOS Data policy requires authors to make all data underlying the findings described in their manuscript fully available without restriction, with rare exception (please refer to the Data Availability Statement in the manuscript PDF file). The data should be provided as part of the manuscript or its supporting information, or deposited to a public repository. For example, in addition to summary statistics, the data points behind means, medians and variance measures should be available. If there are restrictions on publicly sharing data—e.g. participant privacy or use of data from a third party—those must be specified.

Reviewer #1: Yes

Reviewer #2: Yes

**********

5. Is the manuscript presented in an intelligible fashion and written in standard English?

PLOS ONE does not copyedit accepted manuscripts, so the language in submitted articles must be clear, correct, and unambiguous. Any typographical or grammatical errors should be corrected at revision, so please note any specific errors here.

Reviewer #1: Yes

Reviewer #2: Yes

**********

6. Review Comments to the Author

Please use the space provided to explain your answers to the questions above. You may also include additional comments for the author, including concerns about dual publication, research ethics, or publication ethics. (Please upload your review as an attachment if it exceeds 20,000 characters)

Reviewer #1: The manuscript is noticeably clearer and have addressed all previous questions. That said, I still have very few concerns I would appreciate clarification.

The previous manuscript version perhaps removed too many variables; this one may have went in the opposite direction. A model with many interaction terms can make it harder to identify the main effects—and might inadvertently blur the very patterns related to unequal sampling effort that the paper aims to highlight. Some form of model selection or simplification could help. The previous comments were about retaining the random variable in order to control the dependency among the occurrences. This version do not mentioned any model selection steps that could help interpretation.

I am a bit uncertain about using total occurrence records as a proxy for sampling effort. There's a risk of circularity here: high record counts could reflect genuine biological abundance rather than observer effort, which would make it difficult to isolate sampling bias as the driver. Would it be possible to use a more independent effort metric, or at least discuss this limitation more explicitly?

An internal contradiction regarding sampling bias seems to arise from the issues mentioned above. In some places, the text argues that sampling effort does not drive the observed patterns (e.g., Lines 455–458 and 529–534), while in others it suggests that spatial sampling biases are the main driver of observed richness (e.g., Lines 476–482 and 489–491). Since this point is central to the paper's argument, I think it is worth addressing explicitly.

The images are much better, but as they might go in the middle of the text I would recommend increasing the font size. It was possible to read the text only in full size screen.

I appreciate the work already put into the revision.

Reviewer #2: I appreciate the authors’ efforts to revise the manuscript and address previous comments, the manuscript has substantially improved. However, some key conceptual and methodological issues remain unresolved, which continue to limit the robustness of the analyses and the strength of the conclusions.

In particular, important concerns related to sampling bias, spatial dependence, and the interpretation of richness patterns are still not adequately addressed. Although the manuscript acknowledges data gaps and uneven sampling, the interpretation of analyses could lead to confusion over observed richness patterns as output of the ecological process.

Additionally, while the inclusion of biogeographic regions (PAIC) as a random effect is a useful step to account for broad-scale structure, spatial autocorrelation among observations is not explicitly modeled or tested. This limitation may affect statistical inference, as spatial dependence is expected in ecological datasets and can bias parameter estimates and significance tests.

The manuscript also continues to combine amphibians and reptiles within a single analytical framework without providing sufficient evidence that both groups share comparable spatial or ecological patterns. Given their known ecological differences, this approach may introduce bias and obscure meaningful patterns.

Moreover, the Discussion section would benefit from revision, as it is currently too long and somewhat repetitive, which reduces clarity and focus.

Overall, although the revision improves some aspects of the manuscript, the remaining issues are structural and affect the validity of the results and conclusions. Additional work would be required to adequately address these concerns.

**********

7. PLOS authors have the option to publish the peer review history of their article (what does this mean?). If published, this will include your full peer review and any attached files.

If you choose “no”, your identity will remain anonymous but your review may still be made public.

**Do you want your identity to be public for this peer review?**  For information about this choice, including consent withdrawal, please see our Privacy Policy.

Reviewer #1: No

Reviewer #2: No

**********

[NOTE: If reviewer comments were submitted as an attachment file, they will be attached to this email and accessible via the submission site. Please log into your account, locate the manuscript record, and check for the action link "View Attachments". If this link does not appear, there are no attachment files.]

To ensure your figures meet our technical requirements, please review our figure guidelines: https://journals.plos.org/plosone/s/figures

You may also use PLOS’s free figure tool, NAAS, to help you prepare publication quality figures: https://journals.plos.org/plosone/s/figures#loc-tools-for-figure-preparation.

NAAS will assess whether your figures meet our technical requirements by comparing each figure against our figure specifications.

Attachment Submitted filename: PONE-D-25-55790_R1.pdf

# Author comment: Author response to Decision Letter 2

**DOI:** 10.1371/journal.pone.0353393.r004

16 May 2026

REVIEWER 1

On model complexity

We thank Reviewer 1 for this helpful suggestion. We agree that the previous revision included a comparatively complex interaction structure that required clearer justification and interpretation. In response, we simplified the model by retaining area, topographic relief, occurrence density, all pairwise interactions among these predictors, PCA type, and interactions between PCA type and each spatial predictor (PAIC as random effect). We additionally implemented a model selection procedure using AICc-based comparison among candidate models. This procedure confirmed the retained interaction structure as the best-supported model, and we now explicitly describe the model selection approach in the Methods and report the top-supported candidate models in the Supplementary Material (Table S1).

Lines 292 –294:

“Model selection supported retention of the hypothesized full interaction structure and the random intercept, which had the lowest AICc among candidate models and was therefore used for inference (S1 Table).”

Importantly, the retained interaction structure was biologically and analytically motivated because one of the primary objectives of the study was to determine whether the relationships between observed species richness and spatial predictors differ between PCA types. We clarified this rationale in the revised manuscript.

On occurrence density as sampling effort

We agree that total occurrence records are an imperfect proxy for sampling effort and may partly reflect biological signal, detectability, or true variation in abundance rather than observer effort alone. Because standardized sampling-effort metrics are not available consistently across the compiled datasets, occurrence density represented the only broadly comparable metric available at the national scale. To address this concern, we revised the manuscript to treat this variable more cautiously throughout. Specifically, we now explicitly acknowledge that occurrence density may reflect both sampling-related and biological processes, and we avoid interpreting it as a pure measure of observer effort. From lines 274–277, it reads:

“Occurrence density was included as a proxy for sampling effort, while acknowledging that it may also reflect underlying biological variation in abundance and detectability. As such, it represents a composite measure rather than a purely independent estimate of survey effort, and results involving this variable are interpreted with this limitation in mind.”

We also revised several sections of the Results and Discussion to clarify that the analyses evaluate relationships involving observed richness patterns rather than definitive ecological richness estimates (see Manuscript with tracked changes).

On apparent contradiction regarding sampling bias

We appreciate this important observation and agree that the previous revision did not sufficiently distinguish between broad-scale geographic sampling heterogeneity across the Philippines and relationships evaluated within the PCA-level modeling framework.

At the national scale, our analyses demonstrate strong geographic heterogeneity in biodiversity documentation and digitally accessible occurrence records, indicating uneven spatial coverage across the archipelago. In this context, observed richness patterns likely reflect, in part, where biodiversity sampling has historically been concentrated.

In contrast, the PCA-level analyses address a different question: whether variation in observed species richness among conservation units is associated with area, occurrence density, topographic relief, and PCA type after accounting for these variables simultaneously within a unified modeling framework. Within these models, we found no strong evidence that baseline differences between PCA types are explained solely by occurrence density or systematic differences in sampling intensity.

We revised the Discussion throughout to more consistently frame the results in terms of observed or documented richness patterns, while explicitly recognizing that both sampling structure and biological variation likely contribute to the relationships recovered in the analyses.

On figure readability

We thank the reviewer for this suggestion. Figure readability, including font size, was carefully revised during the previous round of revision, and we would prefer to retain the current formatting in the present version. Because several figures contain multiple map layers, panels, legends, and annotations within limited space, further increasing font sizes disrupts the visual balance of the figures and compresses other important graphical elements.

We carefully explored different formatting arrangements through multiple rounds of adjustment and found that the current layout provides the best compromise between readability, spatial clarity, and overall figure composition. We therefore respectfully maintain the present figure formatting. We thank you for your understanding.

REVIEWER 2

On spatial autocorrelation

We appreciate the reviewer’s concern regarding spatial autocorrelation. In our study, the sampling units are Philippine conservation-relevant areas (PCAs), which are irregular spatial polygons that vary substantially in size and shape. The response variable is therefore aggregated species richness at the level of these conservation units rather than point-based occurrence observations. Within this framework, the relevant source of non-independence is broad-scale biogeographic similarity among PCAs rather than fine-scale spatial covariance among individual occurrence records.

To account for this structure, we included Pleistocene Aggregate Island Complex (PAIC) as a random intercept, which captures major regional biogeographic partitioning across the Philippine archipelago at the analytical scale of inference. We further note that commonly used spatial autocorrelation approaches (e.g., distance-based covariance structures or spatial random fields) generally assume comparable spatial units represented by fixed coordinates or adjacency structures. Applying such methods to highly heterogeneous conservation polygons would require additional centroid- or adjacency-based simplifications that are not directly supported by the structure of the dataset.

Lines 287 –291 now reads:

“To account for potential spatial non-independence among PCAs within biogeographic regions (i.e., PCAs share more similarity in species composition by virtue of historical biogeographic structuring), we included the Pleistocene Aggregate Island Complex (PAIC; see [27]) as a random intercept in all models. This structure reflects the analytical scale of inference, which is defined at the level of heterogenous PCA polygons rather than individual occurrence points.”

On combining amphibians and reptiles

We respectfully maintain the joint analysis of amphibians and reptiles because separating these groups would substantially alter the scope and objective of the study. The manuscript does not aim to infer taxon-specific ecological drivers of diversity or compare amphibian and reptile ecology. Rather, the primary objective is to evaluate broad spatial patterns in biodiversity documentation and observed richness across conservation-relevant areas using comparatively well-documented vertebrate taxa in the Philippines.

We now clarify this rationale in the revised Introduction. Amphibians and squamate reptiles were intentionally analyzed together because both groups represent some of the most comprehensively documented terrestrial vertebrates in the Philippines, with extensive specimen representation, taxonomic work, and digitally accessible occurrence data spanning more than a century. Their combined treatment therefore provides a suitable composite dataset for evaluating spatial structure in biodiversity records and conservation-relevant documentation gaps at a national scale. From Lines 93 –97, it reads:

“As two of the most consistently documented vertebrate taxa in the country, herpetofauna provide a uniquely comprehensive dataset for assessing spatial documentation patterns. While acknowledging their distinct ecological niches, we integrate these groups as a singular, informative lens to evaluate biodiversity records across varying sampling landscapes.”

To further address this concern, we revised the manuscript to avoid generalizing ecological interpretations across vertebrate groups and instead frame the results explicitly in terms of observed biodiversity documentation patterns.

On specific comment in the manuscript about uneven sampling limiting the ability to compare observed richness between managed and unmanaged areas:

We thank the reviewer for raising concerns regarding sampling bias, sampling completeness, and the interpretation of observed richness patterns. We agree that biodiversity occurrence data are inherently uneven and that observed richness does not directly represent true species richness. For this reason, our analytical framework explicitly incorporates sampling effort (occurrence density) as a covariate in all models, allowing us to account for variation in sampling effort when estimating relationships between species richness and environmental predictors. In addition, we tested whether sampling intensity differs systematically between PCA types, and found no strong evidence of baseline differences or PCA-type-dependent variation in sampling relationships. We also explicitly acknowledged in the revised manuscript that the number of occurrence points is an imperfect proxy for sampling effort and may reflect biological variation.

Importantly, our inference is explicitly restricted to observed species richness, and we have revised the manuscript to ensure that interpretations were tempered and do not conflate observed patterns with underlying ecological richness. In this context, variation in sampling completeness is not treated as a confounding error to be fully removed, but as an inherent property of biodiversity data that is directly modeled and explicitly quantified.

Regarding the suggestion that analyses should be restricted only to well-sampled units, we respectfully note that this would substantially reduce spatial coverage and disproportionately exclude precisely the under-sampled regions that are central to the objectives of this study. Instead, we adopt a modelling framework that retains all PCAs while accounting for sampling intensity continuously, which we believe is an appropriate approach in large-scale biodiversity syntheses where complete sampling equivalence is not achievable.

Finally, we have revised the Discussion to ensure clearer separation between (i) patterns in observed biodiversity data, (ii) potential ecological interpretation, and (iii) limitations imposed by uneven sampling coverage.

Attachment Submitted filename: Response_to_Reviewers_auresp_2.docx

# Peer review: Decision Letter 2

**DOI:** 10.1371/journal.pone.0353393.r005

**Authors:** Juan Diaz-Ricaurte

23 Jun 2026

Sampling bias and uneven data coverage shape observed biodiversity patterns in a megadiverse island archipelago hotspot

PONE-D-25-55790R2

Dear Dr. Pitogo,

Thank you for submitting the revised version of your manuscript, “Sampling bias and uneven data coverage shape observed biodiversity patterns in a megadiverse island archipelago hotspot,” to PLOS ONE.

I am pleased to let you know that, from an editorial and scientific perspective, your manuscript is now suitable for publication. The journal office may still request technical or formatting adjustments before formal acceptance and production, but no further scientific revision is required from my side.

Before production, I would only encourage you to take one final look at the figure files, particularly the maps, to ensure that key elements such as axis labels, titles, legends, and other visual information remain legible in the final PDF and online versions.

Congratulations to you and your co-authors on reaching this stage.

Kind regards,

Juan Diaz-Ricaurte, Ph.D

Academic Editor

PLOS One

_____

An invoice will be generated when your article is formally accepted. Please note, if your institution has a publishing partnership with PLOS and your article meets the relevant criteria, all or part of your publication costs will be covered. Please make sure your user information is up-to-date by logging into Editorial Manager at Editorial Manager® and clicking the ‘Update My Information' link at the top of the page. For questions related to billing, please contact billing support.

If your institution or institutions have a press office, please notify them about your upcoming paper to help maximize its impact. If they’ll be preparing press materials, please inform our press team as soon as possible -- no later than 48 hours after receiving the formal acceptance. Your manuscript will remain under strict press embargo until 2 pm Eastern Time on the date of publication. For more information, please contact onepress@plos.org.

Reviewers' comments:

Reviewer's Responses to Questions

**Comments to the Author**

1. If the authors have adequately addressed your comments raised in a previous round of review and you feel that this manuscript is now acceptable for publication, you may indicate that here to bypass the “Comments to the Author” section, enter your conflict of interest statement in the “Confidential to Editor” section, and submit your "Accept" recommendation.

Reviewer #1: All comments have been addressed

Reviewer #2: All comments have been addressed

**********

2. Is the manuscript technically sound, and do the data support the conclusions?

The manuscript must describe a technically sound piece of scientific research with data that supports the conclusions. Experiments must have been conducted rigorously, with appropriate controls, replication, and sample sizes. The conclusions must be drawn appropriately based on the data presented.

Reviewer #1: Yes

Reviewer #2: Yes

**********

3. Has the statistical analysis been performed appropriately and rigorously?

Reviewer #1: Yes

Reviewer #2: Yes

**********

4. Have the authors made all data underlying the findings in their manuscript fully available?

The PLOS Data policy requires authors to make all data underlying the findings described in their manuscript fully available without restriction, with rare exception (please refer to the Data Availability Statement in the manuscript PDF file). The data should be provided as part of the manuscript or its supporting information, or deposited to a public repository. For example, in addition to summary statistics, the data points behind means, medians and variance measures should be available. If there are restrictions on publicly sharing data—e.g. participant privacy or use of data from a third party—those must be specified.

Reviewer #1: Yes

Reviewer #2: Yes

**********

5. Is the manuscript presented in an intelligible fashion and written in standard English?

PLOS ONE does not copyedit accepted manuscripts, so the language in submitted articles must be clear, correct, and unambiguous. Any typographical or grammatical errors should be corrected at revision, so please note any specific errors here.

Reviewer #1: Yes

Reviewer #2: Yes

**********

6. Review Comments to the Author

Please use the space provided to explain your answers to the questions above. You may also include additional comments for the author, including concerns about dual publication, research ethics, or publication ethics. (Please upload your review as an attachment if it exceeds 20,000 characters)

Reviewer #1: The authors have properly addressed my comments and suggestions from the previous review rounds. Concerns regarding the modeling steps and the authors' decisions are now well described in the text and supplementary material. Additionally, the previously highlighted contradictions have been corrected.

The maps are readable in high-resolution, and the authors stated that they prefer to keep them as they are. However, I consider that images should be legible on small screens (like tablets), in printed PDFs, and directly on the PLOS ONE website. Therefore, at least important information such as chart axis labels and titles might be readable for comparing even when zoomed-out.

Despite this, I would recommend that my revision finishes here and the work is ready to publication.

Reviewer #2: (No Response)

**********

7. PLOS authors have the option to publish the peer review history of their article (what does this mean?). If published, this will include your full peer review and any attached files.

If you choose “no”, your identity will remain anonymous but your review may still be made public.

**Do you want your identity to be public for this peer review?**  For information about this choice, including consent withdrawal, please see our Privacy Policy.

Reviewer #1: No

Reviewer #2: No

**********

# Editor report: Acceptance letter

**DOI:** 10.1371/journal.pone.0353393.r006

**Authors:** Juan Diaz-Ricaurte

PONE-D-25-55790R2

PLOS One

Dear Dr. Pitogo,

I'm pleased to inform you that your manuscript has been deemed suitable for publication in PLOS One. Congratulations! Your manuscript is now being handed over to our production team.

At this stage, our production department will prepare your paper for publication. This includes ensuring the following:

* All references, tables, and figures are properly cited

* All relevant supporting information is included in the manuscript submission,

* There are no issues that prevent the paper from being properly typeset

You will receive further instructions from the production team, including instructions on how to review your proof when it is ready. Please keep in mind that we are working through a large volume of accepted articles, so please give us a few days to review your paper and let you know the next and final steps.

Lastly, if your institution or institutions have a press office, please let them know about your upcoming paper now to help maximize its impact. If they'll be preparing press materials, please inform our press team within the next 48 hours. Your manuscript will remain under strict press embargo until 2 pm Eastern Time on the date of publication. For more information, please contact onepress@plos.org.

You will receive an invoice from PLOS for your publication fee after your manuscript has reached the completed accept phase. If you receive an email requesting payment before acceptance or for any other service, this may be a phishing scheme. Learn how to identify phishing emails and protect your accounts at https://explore.plos.org/phishing.

If we can help with anything else, please email us at customercare@plos.org.

Thank you for submitting your work to PLOS ONE and supporting open access.

Kind regards,

PLOS ONE Editorial Office Staff

on behalf of

Dr. Juan Diaz-Ricaurte

Academic Editor

PLOS One
